# Multipoint Coordinative Capture of Olefinic Substrates Utilizing Metal‐Carbon Bonds by Macrocyclic Complex

**DOI:** 10.1002/anie.202505734

**Published:** 2025-07-17

**Authors:** Kohtaro Sugawara, Kenichiro Omoto, Takashi Nakamura

**Affiliations:** ^1^ Degree Programs in Pure and Applied Sciences, Graduate School of Science and Technology University of Tsukuba 1‐1‐1 Tennodai Tsukuba Ibaraki 305–8571 Japan; ^2^ Division of Chemistry and Materials Science Graduate School of Integrated Science and Technology Nagasaki University, Bunkyo‐machi Nagasaki Nagasaki 852–8521 Japan; ^3^ Institute of Pure and Applied Sciences University of Tsukuba 1‐1‐1 Tennodai Tsukuba Ibaraki 305–8571 Japan

**Keywords:** Alkene ligands, Fatty acids, Host‐guest systems, Macrocycles, Terpenoids

## Abstract

Terpenoids and unsaturated fatty acids containing C═C double bonds are biologically and synthetically important compounds. The transformation of these substrates catalyzed by proteins relies on precise molecular capture. However, achieving precise molecular recognition of C═C double bonds using synthetically designed hosts remains a significant challenge. We now report a macrocyclic palladium tetranuclear complex that functions as a synthetic receptor featuring coordination sites within its inner cavity. This receptor selectively binds specific double bonds of unsaturated hydrocarbon substrates. Squalene (C_30_H_50_), a linear triterpene with chemically similar six isoprenoid units, was captured by the macrocyclic complex through the coordination of four out of the six C═C double bonds, leading to a unique host‐guest complex with a folded‐squalene structure. Furthermore, the macrocyclic complex exhibited stronger binding affinity for methyl linolenate compared to methyl oleate or methyl linoleate through multipoint coordinative capture. This unprecedented approach to multipoint coordination of specific unsaturated bonds contrasts sharply with biological and traditional artificial receptors, which typically rely on intermolecular interactions such as hydrogen bonding or hydrophobic effects. By achieving precise capture and folding of flexible olefinic substrates, this study establishes a new paradigm for the design of artificial host molecules and a novel platform for enzyme‐like reaction vessels.

## Graphical abstract

We have developed a macrocyclic tetranuclear palladium complex, which has four coordination sites within its inner cavity as a host molecule. The complex captured squalene (C_30_H_50_), a linear triterpene with six C═C double bonds, through multipoint palladium‐carbon bonds, and formed a unique host‐guest complex with a folded‐squalene structure. The host also selectively captured methyl α‐linolenate over methyl oleate and methyl linoleate.

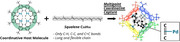

1

The development of artificial host molecules capable of precise recognition is a fundamental pursuit in supramolecular chemistry. Such host molecules hold the potential to enable regio‐ and stereo‐selective reactions, making them valuable tools for sophisticated substrate modification.^[^ ^1^, ^2^, ^3^, ^4^, ^5^, ^6^ ^]^ Biological and most artificial receptors utilize intermolecular interactions,^[^ ^7^, ^8^ ^]^ such as hydrogen bonds^[^ ^9^, ^10^, ^11^, ^12^, ^13^ ^]^ and van der Waals interactions (π–π, dipole etc.),^[^ ^14^, ^15^ ^]^ as well as solvophobic effects represented by the hydrophobic effects^[^ ^16^, ^17^, ^18^, ^19^, ^20^ ^]^ (Figure 1a,b). The hydrogen bondings enable the capture of various polar guests, anions and cations, while the hydrophobic effects lead to the encapsulation of a wide range of hydrophobic substrates. In this context, metal‐ligand coordination offers distinct features, providing not only a thermodynamic stability through strong bonding energy but also reversibility and directionality via the d‐orbitals of the transition metals. As a result, coordination bonds can serve as powerful tools for selective molecular capture. Although artificial hosts capable of binding substrates with strong coordinating groups, such as amines and carboxylates, have been developed (Figure 1b),^[^ ^21^, ^22^, ^23^, ^24^, ^25^, ^26^, ^27^ ^]^ relatively few examples demonstrate a multipoint coordination recognition. This scarcity is due to the difficulty of designing rigid frameworks that position metal coordination sites at regular intervals.^[^ ^27^, ^28^, ^29^, ^30^, ^31^ ^]^

**Figure 1:**
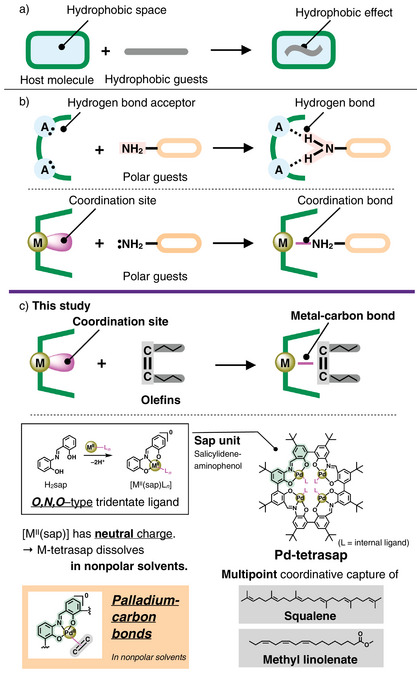
a) Capture of hydrophobic guests by the hydrophobic effect. b) Capture of polar guests by hydrogen bonds or coordination bonds. c) Capture of olefins by coordination bonds and structure of cyclic tetramer of sap (salicylidene‐aminophenol), tetrasap, used in this study. The Pd^II^ complex is electrostatically neutral, facilitating guest capture with less competition from the solvent coordination.

C═C double bonds, unlike amines or carboxylates, are difficult to capture through hydrogen bonding or electrostatic interactions, making them a less commonly targeted functional group in host‐guest chemistry. However, it is well‐established that C═C double bonds readily form organometallic complexes and can be transformed into various functional groups (for instance, hydrogenation, hydrofunctionalization, oxidative functionalization, C─C coupling, metathesis, and polymerization).^[^ ^32^ ^]^ Therefore, the creation of artificial receptors capable of selectively recognizing substrates with C═C double bonds through multipoint coordination would represent an advancement toward “artificial enzymes” that can realize precise, site‐selective molecular transformations.^[^ ^33^, ^34^, ^35^, ^36^, ^37^, ^38^, ^39^, ^40^ ^]^

Our group has been focusing on the design of macrocycles^[^ ^41^ ^]^ through the assembly of chelate complex units,^[^ ^42^, ^43^, ^44^ ^]^ employing them to capture substrates by coordination bonds. For example, we have developed a cyclic hexamer of the pap (pyridylidene‐aminophenol) ligand,^[^ ^45^ ^]^ hexapap, and its hexanuclear complex M‐hexapap (M = Zn^2+^, Ag^+^, Pd^2+^),^[^ ^22^, ^27^, ^46^, ^47^ ^]^ Zn‐hexapap and Pd‐hexapap recognize specific lengths of dicarboxylic acids and diamines, respectively.^[^ ^22^, ^27^ ^]^ However, due to the monovalent negative charge of the deprotonated pap unit and the resulting positive charge of complexes with divalent metal ions, M^II^‐hexapap is only soluble in polar solvents, as with many other supramolecular metal complexes. This solubility restricts its ability to capture guest molecules with weakly coordinating groups, such as the C═C double bond, as competition with solvent molecules for coordination sites becomes significant.

In this study, we focused on the sap (salicylidene‐aminophenol) ligand,^[^ ^48^ ^]^ which forms electrostatically‐neutral complexes with divalent metal ions, and developed a cyclic tetramer of sap, tetrasap (Figure 1c). The cyclic Pd^II^ tetranuclear complex, Pd‐tetrasap, was found to dissolve in non‐coordinating solvents, such as chloroform and benzene, and to capture olefinic guests by palladium‐carbon bonds. We found to selectively capture four out of the six C═C double bonds of squalene in the cavity of Pd‐tetrasap. Squalene lacks characteristic heteroatom functional groups, making its precise capture challenging. Moreover, as the six C═C double bonds are nearly chemically identical, achieving selective recognition is even more difficult. Furthermore, selective capture of two out of the three C═C double bonds of methyl linolenate was realized, and preferential recognition of unsaturated fatty acids depending on the number of unsaturated bonds has been demonstrated.

Tetrasap H_8_ **C4** has been developed as a macrocyclic ligand for a tetranuclear complex that functions as a host molecule. To achieve this, a bifunctional monomer **1** has been designed, whose reduced form can form the sap units through Schiff base formation while undergoing condensation oligomerization. **1** features a nitro group at the 3 position of 2,2´‐biphenol and an acetal‐protected formyl group at the 3´ position (See Figures S1–S10 for the synthesis of **1**). The monomer **1** was subjected to hydrogen reduction and subsequent cyclization with an acid catalyst in chloroform to form tetrasap H_8_ **C4** as a precipitate (Figure 2a). Repeated heating of the filtrate and filtration of the precipitate could yield additional pure H_8_ **C4**, and H_8_ **C4** was obtained as an orange solid in the overall yield of 43%. H_8_ **C4** was characterized by ^1^H NMR, MALDI‐TOF mass, and element analysis (Figures S11–S13).

**Figure 2:**
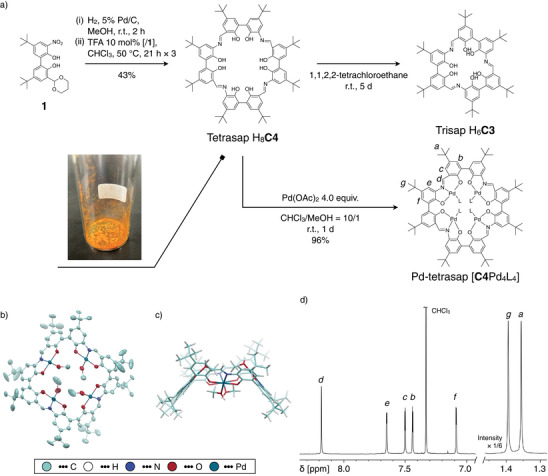
a) Synthesis of tetrasap ligand H_8_ **C4**, conversion to trisap H_6_ **C3**, and complexation to obtain Pd‐tetrasap [**C4**Pd_4_L_4_]. b), c) Structure of [**C4**Pd_4_(MeOH)_4_] determined by X‐ray crystallography. Solvents and disorders are omitted for clarity. b) Top view. An ellipsoidal model (50% probability). Hydrogens are not shown. c) Side view. A capped‐sticks model. d) ^1^H NMR spectrum of [**C4**Pd_4_L_4_] (600 MHz, CDCl_3_/CD_3_OD = 10/1).

Considering that the internal angle between the amino group and the formyl group in the biphenyl monomeric unit is approximately 60 degrees, which is typically favorable for the formation of a cyclic trimer,^[^ ^49^ ^]^ it is noteworthy that the tetramer H_8_ **C4** was obtained as a pure precipitate from a CHCl_3_ solution. Interestingly, when H_8_ **C4** was placed in 1,1,2,2‐tetrachloroethane at room temperature, the sample's appearance changed from an orange suspension to an orange solution. Both ^1^H NMR and MALDI‐TOF mass measurements revealed that tetrasap H_8_ **C4** was almost completely converted to a cyclic trimer of sap, trisap H_6_ **C3** (Figures 2a and S14–S21). This result indicated that the formation of H_8_ **C4** was driven by its limited solubility in chloroform, while H_6_ **C3** represents the thermodynamically‐stable species in solution. This study thus provides an intriguing example of utilizing the dynamic covalent bonding properties of imines to selectively produce distinct cyclic oligomers under specific conditions.^[^ ^50^ ^]^

The reaction of H_8_ **C4** with Pd(OAc)_2_ resulted in the formation of Pd‐tetrasap [**C4**Pd_4_L_4_] (Figure 2a) (L = exchangeable ligands such as MeOH, H_2_O, EtOH, AcOH, see the supporting information for detailed characterization, Figures S24–S30). The structure of [**C4**Pd_4_(MeOH)_4_] was determined by X‐ray crystallography (Figures 2b,c and S33).^[^ ^51^ ^]^ The Pd^II^ centers adopt a four‐coordinate square planar geometry, featuring *O,N,O*‐tridentate chelation by the sap units and the *O*‐monodentate coordination of methanol. The overall structure of the Pd‐tetrasap molecule is saddle‐shaped with methanol‐coordination sites arranged in an alternating up–down–up–down conformation (*S*_4_ symmetry). ^1^H NMR spectrum of [**C4**Pd_4_L_4_] in CDCl_3_/CD_3_OD  =  10/1 (Figures 2d and S32) also supported a time‐averaged *S*_4_ symmetric complex, in which four sap units coordinated by CD_3_OD are equivalent.

In terms of solubility, the Pd‐tetrasap [**C4**Pd_4_L_4_] exhibits a good solubility in non‐polar solvents, such as chloroform and benzene, in contrast to our previous reported Pd‐hexapap complex and other positively charged multinuclear complexes, which are typically not soluble in non‐polar solvents. This solubility character is attributed to the electroneutrality of the Pd‐tetrasap.

The olefin‐binding ability of Pd‐tetrasap [**C4**Pd_4_L_4_] was first discovered with amylene (2‐methyl‐2‐butene), a structural unit of squalene and a compound commonly used as a stabilizer in chloroform solvents. In an attempt to align the internal ligands L to water, after a chloroform (containing amylene) solution of [**C4**Pd_4_L_4_] was washed with water and the organic layer was evaporated, the amylene signal was observed in a ^1^H NMR measurement of the obtained complex redissolved in CDCl_3_ or CDCl_3_/CD_3_OD  =  10/1 (Figures S34 and S35). Since the boiling point of amylene (39 °C) is lower than that of chloroform (61 °C), amylene does not usually remain when the chloroform solution is evaporated. This result suggested that Pd‐tetrasap has the ability to capture C═C double bonds. In a separate titration experiment, when amylene was incrementally added to the CDCl_3_ (amylene‐free) solution of [**C4**Pd_4_L_4_], [**C4**Pd_4_(amylene)_4_], in which four amylene molecules were coordinated to palladium, was formed. The olefin ^1^H NMR signals of the complex appeared at 5.41–5.25 ppm, shifted downfield from the original signal at 5.18 ppm (Figures S36 and S37). This observation confirmed that Pd‐tetrasap made bonds with amylene via coordination.

Next, squalene, a linear C30 triterpene with six C═C double bonds, was investigated as a guest molecule. The ^1^H NMR spectrum obtained after adding one equivalent of squalene to [**C4**Pd_4_L_4_] in C_6_D_6_ is shown in Figure 3a,b. The spectrum indicates the formation of a single host‐guest complex, and the integration values confirmed the formation of a one‐to‐one complex, [**C4**Pd_4_(squalene)]. The host‐guest complex was also observed in ESI‐TOF and MALDI‐TOF mass measurements (Figures S45 and S46). In the ^1^H NMR spectrum of [**C4**Pd_4_(squalene)], it was indicated that all four sap units of Pd‐tetrasap were in different chemical environments. For instance, without a guest molecule, only one imine proton signal was observed, whereas four distinct imine proton signals appeared in the spectrum of [**C4**Pd_4_(squalene)]. Also, focusing on the guest molecule, the six olefinic protons of squalene exhibited different chemical shifts in the presence of Pd‐tetrasap. In free squalene, these protons showed nearly identical chemical shifts at δ 5.35–5.21 ppm. However, upon complexation, four protons shifted downfield to 6.31, 6.24, 6.00, and 5.95 ppm, while two protons shifted upfield to 4.66 and 4.59 ppm. Considering the downfield shift of the olefinic proton of amylene upon coordination, these shifts suggest that four out of the six C═C double bonds in squalene were coordinated by Pd‐tetrasap (see Figures S39–S44 for further details).

**Figure 3:**
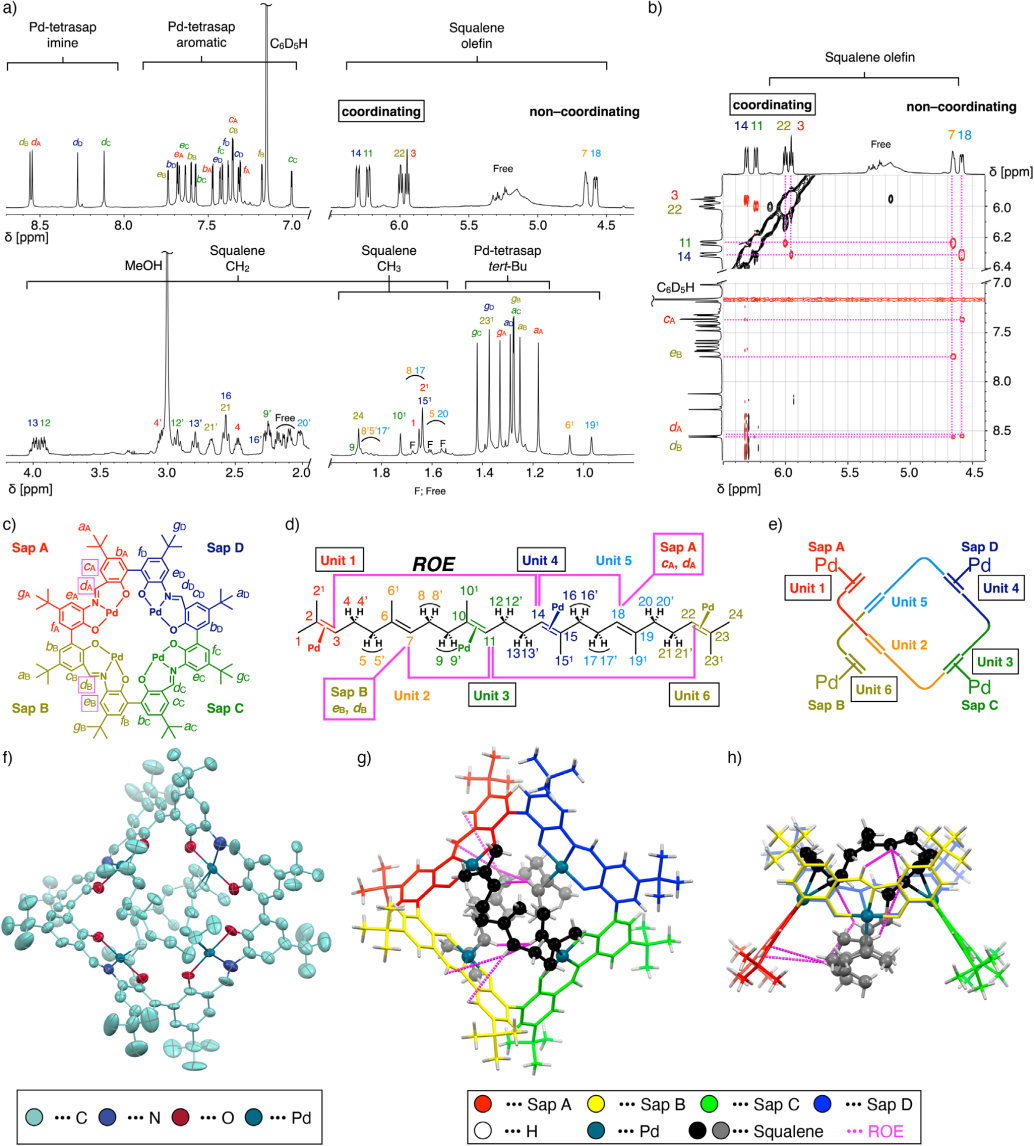
a) ^1^H NMR spectrum of [**C4**Pd_4_(squalene)] (600 MHz, C_6_D_6_). b) ^1^H–^1^H ROESY NMR spectrum of [**C4**Pd_4_(squalene)] (600 MHz, C_6_D_6_). c), d) Assignments of ^1^H NMR signals of [**C4**Pd_4_(squalene)]. c) Pd‐tetrasap part. d) Squalene part. e) An overview structure of [**C4**Pd_4_(squalene)]. (f–h) Structure of [**C4**Pd_4_(squalene)] determined by X‐ray crystallography. Disordered parts are omitted for clarity. f) Top view. An ellipsoidal model (30% probability). Hydrogens are not shown for clarity. (g,h) A ball‐and‐sticks model. g) Top view. h) Side view.

A single crystal of [**C4**Pd_4_(squalene)] was obtained and its structure was successfully determined by X‐ray crystallography (Figures 3f–h and S64–S67).^[^ ^51^ ^]^ As anticipated from the NMR analysis, squalene was captured by the Pd‐tetrasap through coordination with four of its C═C double bonds. Specifically, the double bonds at positions 2,3, 10,11, 13,14, and 22,23 (counting from the terminal as the 1st, 3rd, 4th, and 6th double bonds) formed coordination bonds with palladium. Similar to the structure of the methanol‐coordinated Pd‐tetrasap (Figure 2b,c), the macrocyclic framework of Pd‐tetrasap in [**C4**Pd_4_(squalene)] displayed an alternating up–down–up–down arrangement of its Pd coordination sites. Within the cavity of the Pd‐tetrasap, the linear squalene molecule adopted a folded conformation, coordinating to Pd centers in an up–up–down–down sequence. The remaining two C═C double bonds at positions 6,7 and 18,19 were not coordinated to Pd and remained free, located at the outermost upper and lower regions in the cavity.

The ^1^H NMR assignment of [**C4**Pd_4_(squalene)], performed using ^1^H–^1^H COSY, ^1^H–^1^H TOCSY, ^1^H–^1^H ROESY, ^11^H–^13^C HSQC, and ^1^H–^13^C HMBC NMR techniques, confirmed that the structure observed in the crystal was also present in solution (Figures S47–S63). In this assignment, the sap units of Pd‐tetrasap were labeled counterclockwise as sap A, sap B, sap C, and sap D, and the alkene units of squalene were sequentially numbered from the terminal as unit 1, unit 2, …, unit 6 (Figure 3c,d). The assignment of the NMR signals for these units was unambiguously determined by sequentially tracing the relationships between them using COSY, TOCSY, ROESY, HSQC, and HMBC measurements. Regarding the olefin protons of squalene, the four protons showing downfield shifts were assigned to the olefinic protons of units 1, 3, 4, and 6 (Figure 3e), consistent with their coordination to palladium as demonstrated by X‐ray crystallography. ^1^H–^1^H ROESY NMR revealed ROE correlations between the non‐coordinated olefinic protons of units 2 and 5 of the squalene and the imine protons of sap B and sap A of the Pd‐tetrasap, respectively (Figure 3b). These pairs of protons were found to be in close proximity in the structure observed by X‐ray crystallography (Figure 3g,h). Furthermore, the ROE correlations observed between the olefinic protons of squalene (3–14, 14–18, 7–11, and 11–22) matched with its folded structure within the cavity (Figure 3b,d,g,h), further reinforcing the structural consistency between the crystalline and solution states.

Isoprenoids other than squalene (geraniol, farnesol, solanesol, coenzyme Q_10_, and 2,3‐oxidosqualene) were also shown to be captured by Pd‐tetrasap through olefin coordination (Figures S71–S77). Unlike the squalene complex, the ^1^H NMR spectra of the host‐guest complexes with these substrates were more complex, suggesting the presence of multiple binding modes. Competitive binding experiments were conducted to assess whether squalene (1 equiv.) or amylene (40 equiv.), or the other isoprenoids (10 equiv.), binds more strongly to Pd‐tetrasap (Figure 4a). ^1^H NMR analysis revealed that, in all cases, the signals for the squalene complex were predominantly observed, while signals for the competitive guest complexes were either significantly weaker or below the S/N ratio in their NMR spectra. Specifically, the ratio of the host‐guest complex of squalene and that of the competitive guests was at least larger than 6 for amylene, 22 for geraniol, and 3 for solanesol (Figures S88–S95 and Tables S2–S5). These results demonstrate the ability of Pd‐tetrasap to strongly capture olefinic substrates that possess double bonds at intervals that match the spatial arrangement of the Pd coordination sites through multi‐point recognition.

**Figure 4:**
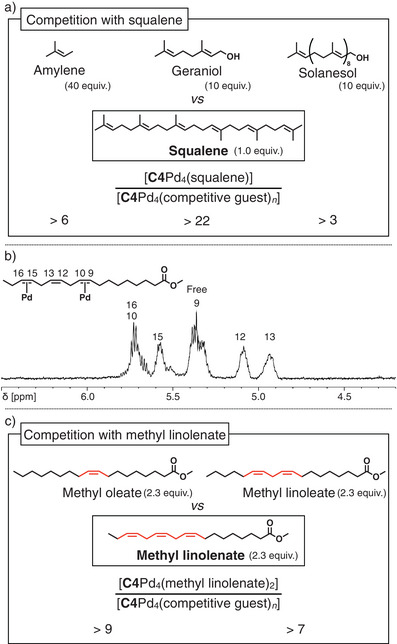
a) Competitive binding experiments of squalene and amylene or other isoprenoids (^1^H NMR, CDCl_3_, 298 K). b) ^1^H NMR spectrum of [**C4**Pd_4_(methyl linolenate)_2_] (600 MHz, CDCl_3_). c) Competitive binding experiments of methyl linolenate and other unsaturated fatty acid methyl esters (^1^H NMR, CDCl_3_, 298 K).

Furthermore, the coordinative capture of unsaturated fatty acid methyl esters (methyl oleate, methyl linoleate, and methyl α‐linolenate) as olefinic substrates was investigated. All of these fatty acid esters were captured by Pd‐tetrasap, with methyl linolenate displaying sharper ^1^H NMR signals than the other unsaturated fatty acid esters (Figures 4b and S78–S81). Analysis using 2D NMR spectroscopy (Figures S82–S86) revealed that, among the three C═C double bonds of the methyl linolenate, the two double bonds at 9,10 and 15,16 positions coordinated to palladium to form [**C4**Pd_4_(methyl linolenate)_2_]. Competition experiments demonstrated that the ratio of the host‐guest complex of methyl linolenate and that of the other methyl esters was at least larger than 9 for methyl oleate, and 7 for methyl linoleate (Figures 4c, S98–S101, and Tables S7 and S8). This selectivity to methyl linolenate highlights the effectiveness of multipoint capture.

In summary, we have synthesized a novel macrocyclic ligand, tetrasap H_8_ **C4**, and its Pd tetranuclear complex, Pd‐tetrasap [**C4**Pd_4_L_4_], which features four coordination sites within its inner cavity. Pd‐tetrasap demonstrated the remarkable ability to capture squalene—a molecule composed solely of carbon and hydrogen—through coordination of its specific C═C double bonds, forming the unique host‐guest complex [**C4**Pd_4_(squalene)]. Notably, the multipoint coordination enabled atomic‐level precision in the folding and fixing of the linear molecular scaffold, holding it in a well‐defined conformation in solution as revealed by NMR spectroscopy. Moreover, Pd‐tetrasap selectively captured methyl α‐linolenate over methyl oleate or methyl linoleate utilizing the multipoint metal‐carbon coordination. These findings, achieved through the integration of metal coordination sites in the macrocycle, represent a novel approach to the design of artificial hosts, introducing a recognition strategy distinct from typically‐employed intermolecular interactions such as hydrogen bonding and Coulomb forces.

## Supporting Information

The authors have cited additional references within the Supporting Information.^[^ ^52^, ^53^, ^54^, ^55^, ^56^, ^57^, ^58^, ^59^, ^60^, ^61^, ^62^, ^63^, ^64^, ^65^ ^]^

## Conflict of Interests

The authors declare no conflict of interest.

## Supporting information



Supporting Information

## Data Availability

The data that support the findings of this study are available in the supplementary material of this article.
