# Supplementary material for: Multipoint Coordinative Capture of Olefinic Substrates Utilizing Metal‐Carbon Bonds by Macrocyclic Complex
Source: Angew Chem Int Ed Engl. 2025 Jul 17;64(35):e202505734. doi: 10.1002/anie.202505734 (PMC12377451; doi:10.1002/anie.202505734)
Supplement: Supplementary file 1 — Supporting Information [file ANIE-64-e202505734-s001.pdf]

The Supporting Information for

Multipoint Coordinative Capture of Olefinic Substrates Utilizing Metal-Carbon Bonds by Macrocyclic Complex

Kohtaro Sugawara[1], Kenichiro Omoto[2] & Takashi Nakamura[3,*]

[1]Degree Programs in Pure and Applied Sciences, Graduate School of Science and Technology, University of Tsukuba, 1-1-1 Tennodai, Tsukuba, Ibaraki 305-8571, Japan.

[2]Division of Chemistry and Materials Science, Graduate School of Integrated Science and Technology, Nagasaki University, Bunkyo-machi, Nagasaki, Nagasaki 852-8521, Japan.

[3]Institute of Pure and Applied Sciences, University of Tsukuba, 1-1-1 Tennodai, Tsukuba, Ibaraki 305-8571, Japan.

*Corresponding Author. E-mail: nakamura@chem.tsukuba.ac.jp

Contents

1. Materials and methods ... 3

2. Synthesis and characterization of the compounds ... 4

2-1. Synthesis of **1** ... 4

2-2. Synthesis of tetrasap H_8**C4** ... 11

3. Conversion from tetrasap H_8C4 to trisap H_6C3 ... 14

3-1. Conversion from tetrasap H_8**C4** to trisap H_6**C3** by 1,1,2,2-tetrachloroethane ... 14

3-2. Behavior of tetrasap H_8**C4** in $CDCl_3$ ... 21

4. Formation of tetranuclear palladium complex [C4Pd_4L_4] ... 22

4-1. Synthesis of [**C4**Pd_4L_4] ... 22

4-2. NMR assignment of [**C4**Pd_4L_4] ... 24

4-3. Comparison of ^{1}H NMR spectra in different solvents ... 29

4-4. Comparison of ^{1}H NMR spectra for different coordination solvents L ... 30

4-5. X-ray diffraction analysis of [**C4**Pd_4(MeOH)$_4$] ... 31

5. Coordination binding experiments of amylene ... 32

5-1. Coordination binding of amylene as a stabilizer in chloroform ... 32

5-2. Titration of amylene against [**C4**Pd_4L_4] ... 34

6. Coordination binding experiments of squalene ... 36

6-1. Titration of squalene against [**C4**Pd_4L_4] ... 36

6-2. NMR assignment of [**C4**Pd_4(squalene)] ... 42

6-3. X-ray diffraction analysis of [**C4**Pd_4(squalene)] ... 54

6-4. Addition of squalene against Pd-hexapap ... 57

7. Coordination binding experiments of other isoprenoids ... 59

7-1. Geraniol ... 59

7-2. Farnesol (mixture of isomers) ... 61

7-3. Solanesol ... 62

7-4. Coenzyme Q_{10} (ubiquinone 10) ... 63

7-5. 2,3-Oxidosqualene ... 64

8. Coordination binding experiments of unsaturated fatty acid methyl esters ... 66

8-1. Methyl oleate ... 66

8-2. Methyl linoleate ... 68

8-3. Methyl linolenate ... 69

9. Ratio of guest molecules captured by Pd-tetrasap ... 76

10. Competition experiments of guest molecules ... 77

10-1. Competition of squalene and amylene ... 77

10-2. Competition of squalene and geraniol or solanesol ... 84

10-3. Competition of squalene and methanol ... 87

10-4. Competition of methyl linolenate and methyl oleate or methyl linoleate ... 89

1. Materials and methods

Unless otherwise noted, the solvents and reagents were purchased from TCI Co., Ltd., FUJIFILM Wako Pure Chemical Industries, Ltd., Kanto Chemical Co., Inc., Nacalai Tesque, Inc., Sigma-Aldrich Co., Angene Int., Ltd., BLD Pharmatech Ltd., Eurisotop or Cambridge Isotope Laboratories, Inc., and used without further purification. Dry THF was purified by Glass Contour Ultimate Solvent System. Silica gel for column chromatography was purchased from Kanto Chemical Co. Inc. (Silica Gel 60 N, spherical, 63–210 µm or Silica Gel 60, spherical, 40–50 µm). Automated column purification was performed by Biotage Isolera One (column: Sfär Silica HC Duo 20 µm).

Measurements were performed at r.t. unless otherwise noted. ^{1}H, ^{13}C, and other 2D NMR spectra were recorded by a Bruker AVANCE III-600 (600 MHz) spectrometer, a Bruker AVANCE II-500 (500 MHz) spectrometer or a Bruker AVANCE III-400 (400 MHz) spectrometer. The assignments of ^{1}H and ^{13}C signals were based on ^{1}H-^{1}H COSY, ^{1}H-^{1}H TOCSY, ^{1}H-^{1}H NOESY, ^{1}H-^{1}H ROESY, ^{1}H-^{13}C HSQC, and ^{1}H-^{13}C HMBC measurements. Tetramethylsilane was used as the internal standard (δ 0.00 ppm) for the ^{1}H and ^{13}C NMR measurements when $CDCl_3$ or a mixed solvent with $CDCl_3$ was used as the solvent. Solvent residual signals were used as a standard for ^{1}H and ^{13}C NMR measurements when solvents other than $CDCl_3$ were used[52]. For ^{1}H and ^{13}C NMR measurements in 1,1,2,2-tetrachloroethane-d_2, the solvent residual signals were set to 6.00 ppm and 73.78 ppm, respectively.

The MALDI-TOF mass data were recorded by an AB SCIEX TOF/TOF 5800 system or a Bruker autoflexmaX TOF/TOF system. The ESI-TOF mass data were recorded by an AB SCIEX TripleTOF 4600 system or a Bruker compact QTOF system. Melting points were measured by Yanaco LID Co., Ltd. MP-J3 instrument.

Single-crystal X-ray crystallographic measurements were performed using Bruker APEX II ULTRA with Mo *K*α radiation at 100 K. Obtained data were collected using Bruker APEX2[53] and processed using Bruker APEX3[54] and Yadokari-XG[55],[56] crystallographic software package. The initial structures were solved using SHELXT-2018[57] or SHELXD-2013[58] and refined using SHELXL-2018[59].

Elemental analysis was performed on a Yanaco MT-6 analyzer with tin boats purchased from Elementar. We appreciate Mr. Masao Sasaki (Univ. of Tsukuba) for the measurements.

We appreciate the Chemical Analysis Division, Research Facility Center for Science and Technology (Univ. of Tsukuba) for the MALDI-TOF mass measurements. We appreciate the Organization of Open Facility Initiatives (Univ. of Tsukuba) for the NMR, MALDI-TOF mass and ESI-TOF mass measurements.

2. Synthesis and characterization of the compounds

2-1. Synthesis of **1**

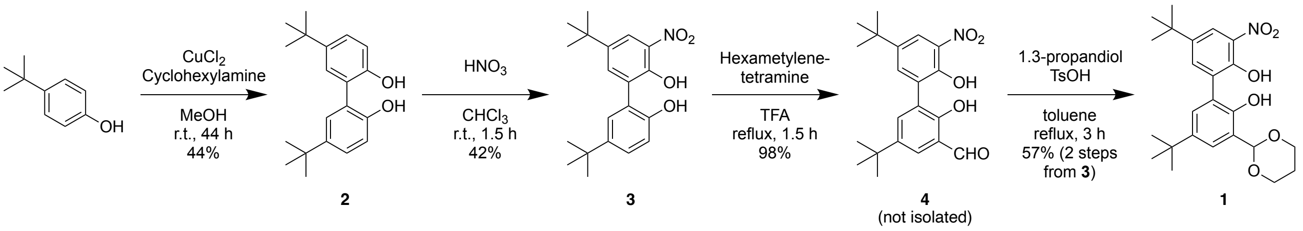

Synthesis of biphenol **2**

2 was synthesized based on a previously reported procedure for biphenols.[60] In a 2 L three-necked round bottom flask, a mixture of 4-*tert*-butylphenol (25.35 g, 168.7 mmol, 1.0 equiv.), cyclohexylamine (46.0 mL, 405 mmol, 2.4 equiv.) and $CuCl_2 \cdot 2H_2O$ (28.91 g, 169.6 mmol, 1.0 equiv.) in MeOH (300 mL) was stirred at r.t. for 44 h with bubbling of the air into the solution for 15 h (MeOH was added to compensate for the evaporation during the reaction). The reaction mixture was filtered, and to the obtained solid were added 2 M HCl aq. (200 mL) and AcOEt (300 mL), then stirred at r.t. for 1 h. The organic layer was separated and washed with 1 M HCl aq. (200 mL) and water (200 mL). The organic layer was dried over Na_2SO_4, filtered, and concentrated in vacuo. The residue was recrystallized from toluene twice and hexane/toluene = 2/1 to give **2**[61] as a pale pink solid (11.18 g, 37.46 mmol, 44%).

^{1}H NMR (400 MHz, $CDCl_3$): δ 7.36 (dd, J = 8.5, 2.4 Hz, 2H), 7.27 (d, J = 2.4 Hz, 2H), 6.98 (d, J = 8.5 Hz, 2H), 5.32 (br, 2H), 1.33 (s, 18H);

HRMS (ESI): m/z calcd for $C_{20}H_{26}O_2Na$ ([**2**+Na]$^+$): 321.1825; found: 321.1845.

Synthesis of nitrophenol **3**

In a 10 mL volumetric flask, 97% HNO_3 aq. (3.517 g) was diluted with $CHCl_3$ (solution A). In a 1 L eggplant flask, to a solution of **2** (11.00 g, 36.86 mmol, 1.0 equiv.) in $CHCl_3$ (280 mL) was added the solution A (6.80 mL, HNO_3: 2.32 g, 36.8 mmol, 1.0 equiv.), and then the mixture was stirred at r.t. for 1.5 h. The reaction was quenched by adding water (300 mL) to the reaction mixture, and the aqueous layer was extracted with $CHCl_3$ (300 mL × 3). The organic layer was dried over Na_2SO_4, filtered, and concentrated in vacuo. The residue was purified by column chromatography on silica gel (63–210 µm, *n*-hexane/$CHCl_3$ = 1/1–1/2 and 40–50 µm, *n*-hexane/$CHCl_3$ = 2/1) and dried in vacuo to give **3** as a red oil (5.326 g, 15.51 mmol, 42%).

^{1}H NMR (400 MHz, $CDCl_3$): δ 11.30 (br, 1H), 8.17 (d, J = 2.4 Hz, 1H), 7.71 (d, J = 2.4 Hz, 1H), 7.39 (dd, J = 8.5, 2.4 Hz, 1H), 7.22 (d, J = 2.4 Hz, 1H), 7.00 (d, J = 8.5 Hz, 1H), 5.55 (br, 1H), 1.37 (s, 9H), 1.34 (s, 9H);

^{13}C NMR (101 MHz, $CDCl_3$): δ 151.1, 149.5, 144.2, 144.1, 138.0, 133.6, 129.6, 128.0, 127.2, 123.5, 120.9, 117.3, 34.6, 34.2, 31.5, 31.1;

HRMS (ESI): m/z calcd for $C_{20}H_{25}NO_4Na$ ([**3**+Na]$^+$): 366.1676; found: 366.1681.

Synthesis of salicylaldehyde **4**

In a 300 mL eggplant flask, a solution of **3** (5.033 g, 14.66 mmol, 1.0 equiv.) and hexamethylenetetramine (2.057 g, 14.67 mmol, 1.0 equiv.) in TFA (30 mL) was refluxed (85–90 ºC, bath temp.) for 1.5 h under an Ar atmosphere. The reaction was quenched by adding water (150 mL) after the reaction mixture was cooled to r.t., then the aqueous layer was extracted with $CHCl_3$ (200 mL × 3). The organic layer was dried over Na_2SO_4, filtered, and concentrated in vacuo to give crude **4** as a dark yellow oil (5.354 g, 14.41 mmol, 98% if pure). The crude was used next reaction without further purification.

^{1}H NMR (600 MHz, $CDCl_3$): δ 11.25 (br, 1H), 10.87 (br, 1H), 9.96 (s, 1H), 8.14 (d, J = 2.5 Hz, 1H), 7.73 (d, J = 2.5 Hz, 1H), 7.67 (d, J = 2.5 Hz, 1H), 7.61 (d, J = 2.5 Hz, 1H), 1.38 (s, 9H), 1.36 (s, 9H);

^{13}C NMR (151 MHz, $CDCl_3$): δ 196.9, 157.1, 151.2, 142.9, 142.6, 137.8, 136.8, 133.8, 130.4, 127.5, 124.7, 121.0, 120.6, 34.6, 34.4, 31.4, 31.3;

HRMS (ESI): *m/z* calcd for $C_{21}H_{25}NO_5Na$ ([**4**+Na]$^+$): 394.1625; found: 394.1633.

(Pure **4** was obtained during purification of **1**.)

Synthesis of acetal protected salicylaldehyde **1**

In a 100 mL eggplant flask, a solution of the crude **4** (5.079 g, 13.68 mmol, 1.0 equiv. if pure), 1,3-propanediol (3.5 mL, 3.68 g, 48.3 mmol, 3.5 equiv.) and TsOH·H_2O (0.2657 g, 1.397 mmol, 10 mol%) in dry toluene (50 mL) was refluxed (145 ºC, bath temp.) for 3 h under an Ar atmosphere. The reaction mixture was concentrated in vacuo. AcOEt (100 mL) was added to the crude, and the solution was washed with sat. $NaHCO_3$ aq. (100 mL × 2). The organic layer was dried over Na_2SO_4, filtered, and concentrated in vacuo, and was purified by automated column chromatography on silica gel (*n*-hexane/$CHCl_3$ = 50/50–0/100) to give **1** as a dark yellowish green oil (3.414 g, 7.949 mmol, 57% (two steps from **3**)).

^{1}H NMR (600 MHz, $CDCl_3$): δ 10.85 (br, 1H), 8.09 (d, J = 2.5 Hz, 1H), 7.88 (br, 1H), 7.67 (d, J = 2.5 Hz, 1H), 7.28 (d, J = 2.5 Hz, 1H), 7.26 (d, J = 2.5 Hz, 1H), 5.72 (s, 1H), 4.33–4.30 (m, 2H), 4.06–4.01 (m, 2H), 2.30–2.22 (m, 1H), 1.53–1.50 (m, 1H), 1.35 (s, 9H), 1.32 (s, 9H);

^{13}C NMR (151 MHz, $CDCl_3$): δ 151.2, 150.3, 142.6, 142.2, 137.8, 133.4, 129.8, 129.3, 125.0, 124.1, 122.0, 120.2, 103.3, 67.5, 34.4, 34.2, 31.5, 31.1, 25.7;

HRMS (ESI): *m/z* calcd for $C_{24}H_{31}NO_6Na$ ([**1**+Na]$^+$): 452.2044; found: 452.2060.

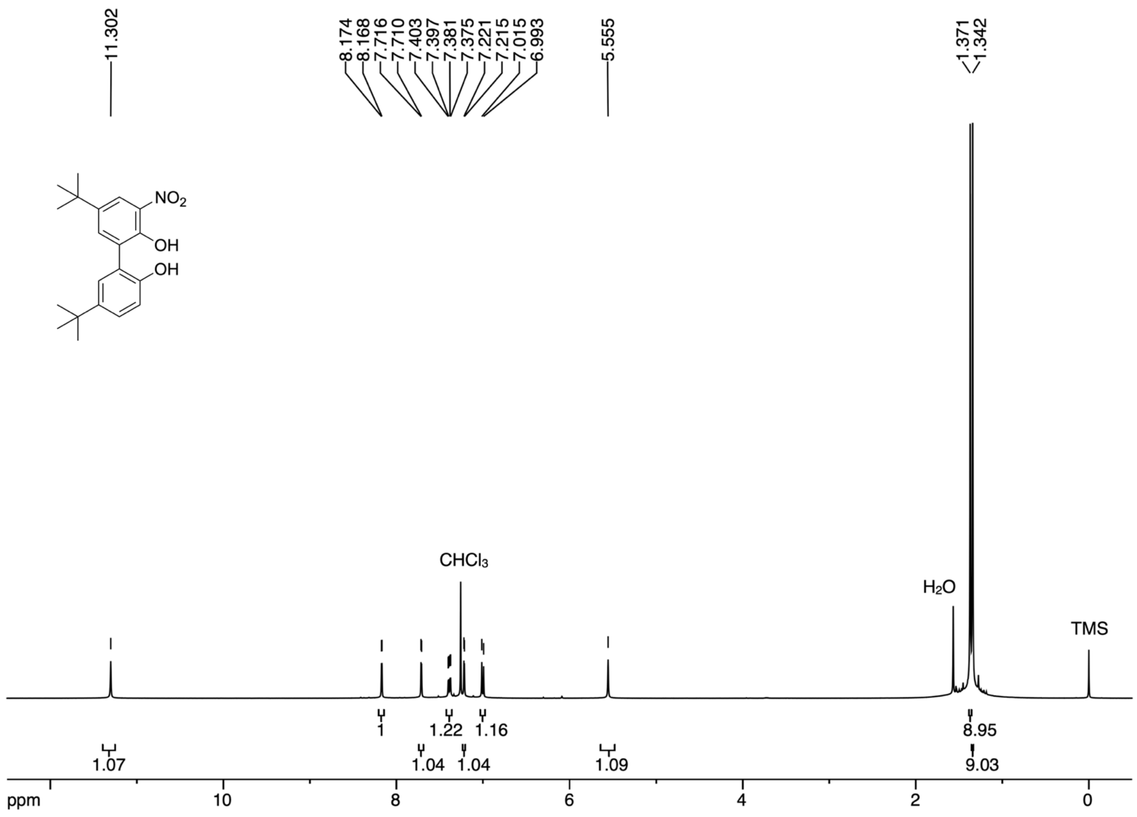

Figure S1. ^{1}H NMR spectrum of **3** (400 MHz, $CDCl_3$).

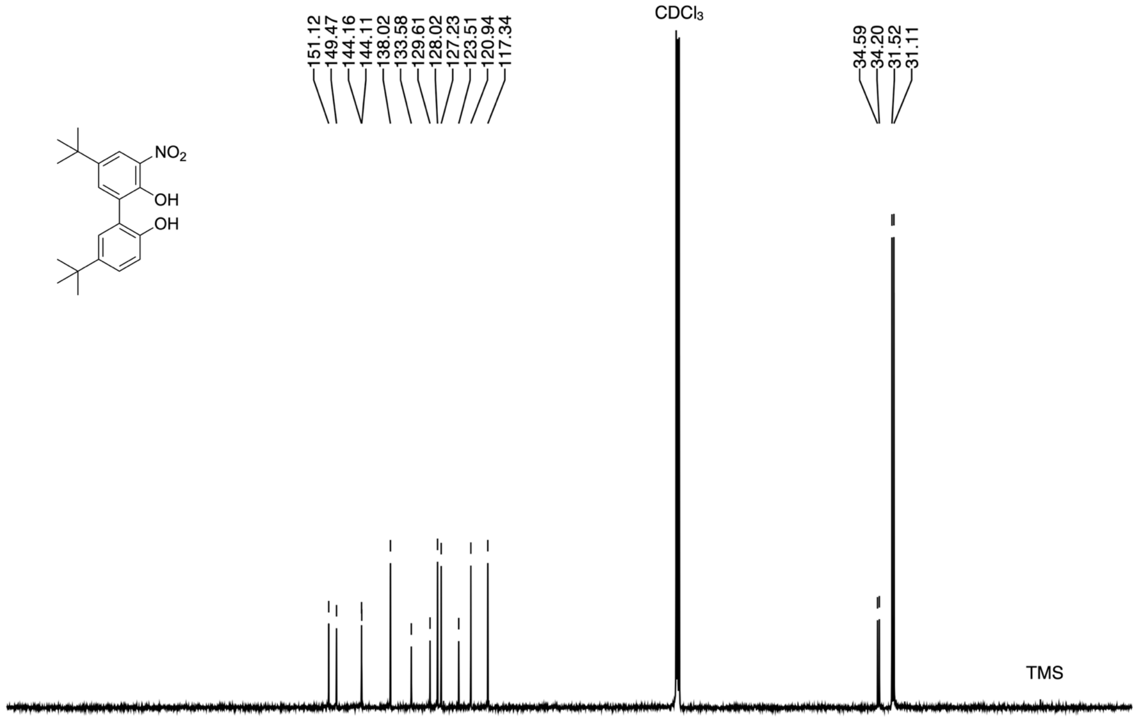

Figure S2. ^{13}C NMR spectrum of **3** (101 MHz, $CDCl_3$).

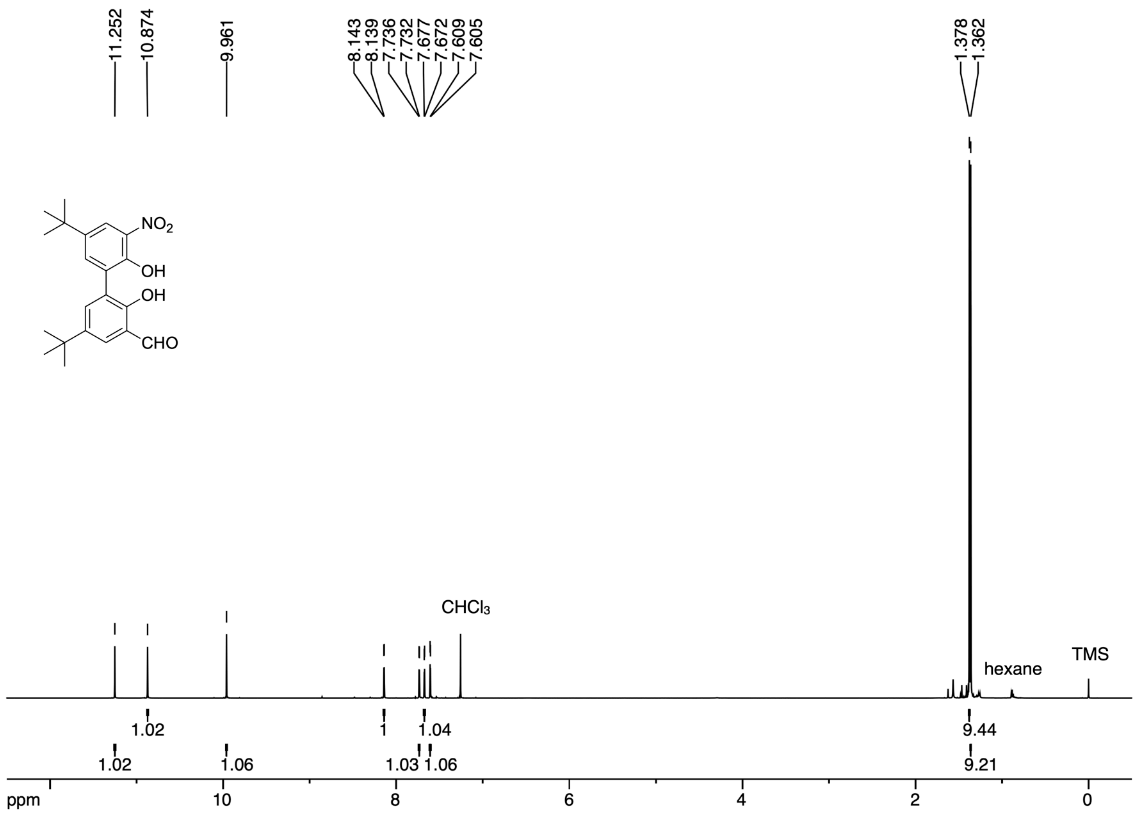

Figure S3. ^{1}H NMR spectrum of **4** (600 MHz, $CDCl_3$).

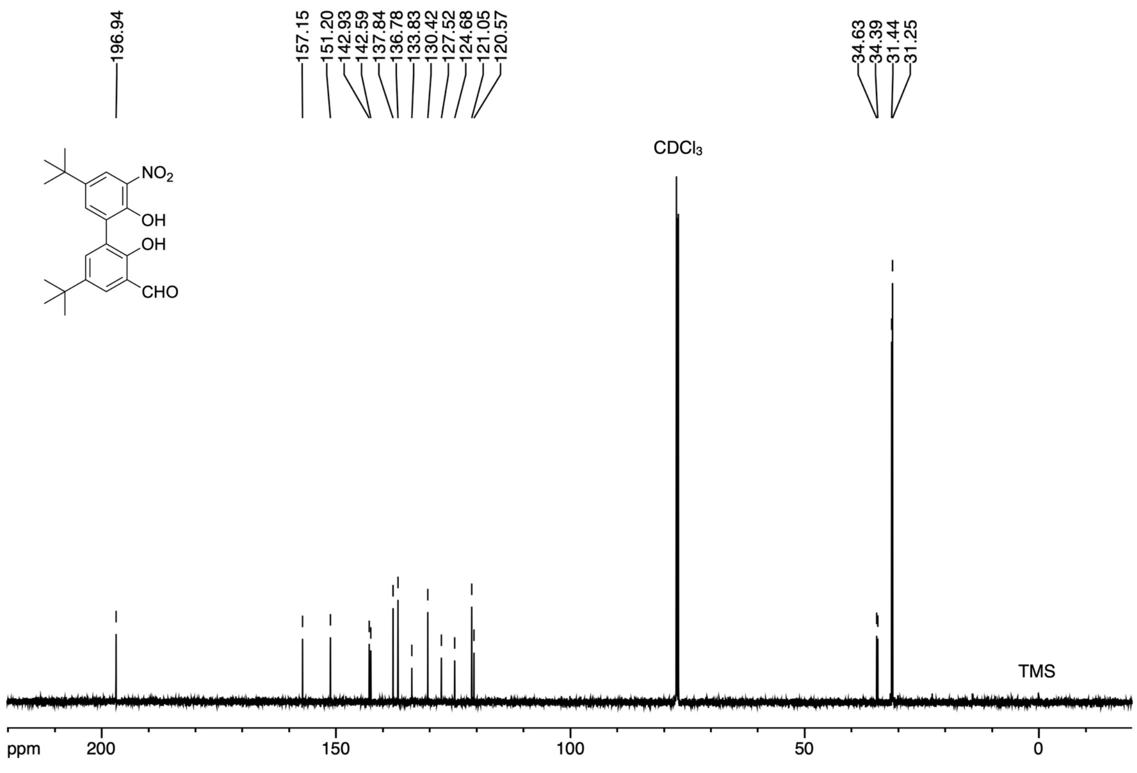

Figure S4. ^{13}C NMR spectrum of **4** (151 MHz, $CDCl_3$).

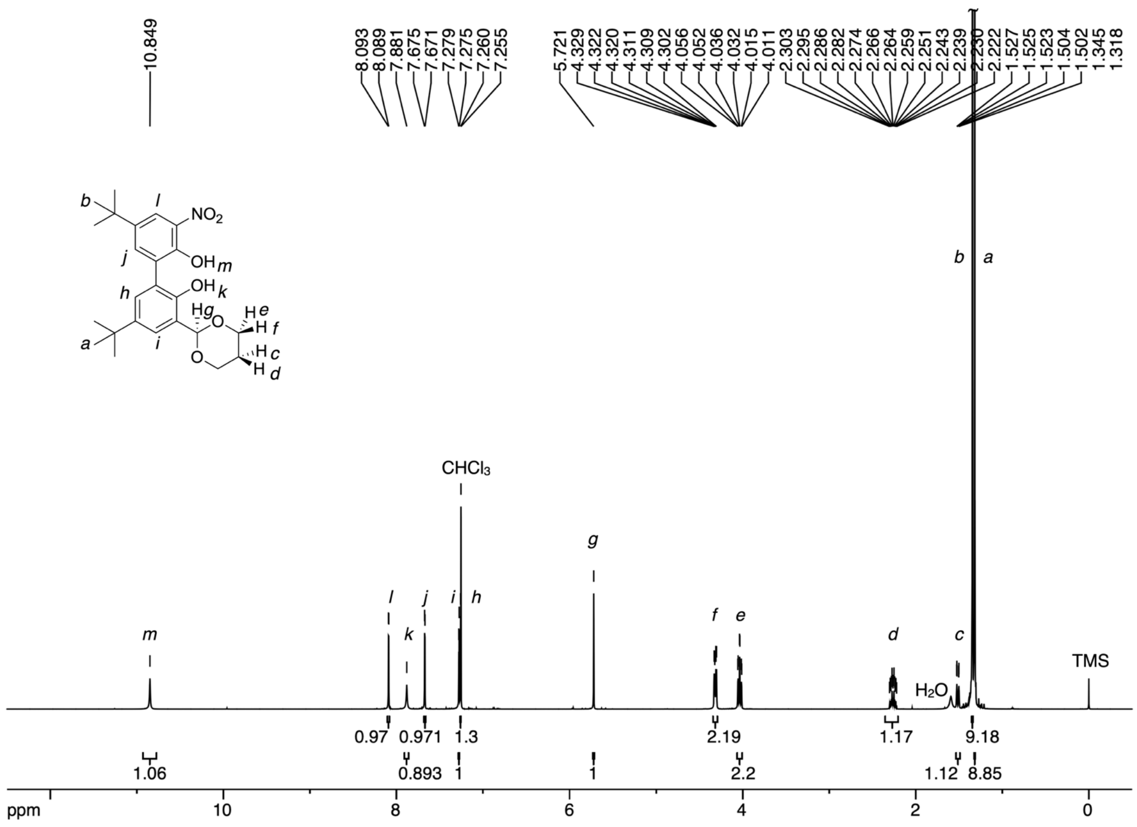

Figure S5. ^{1}H NMR spectrum of **1** (600 MHz, $CDCl_3$).

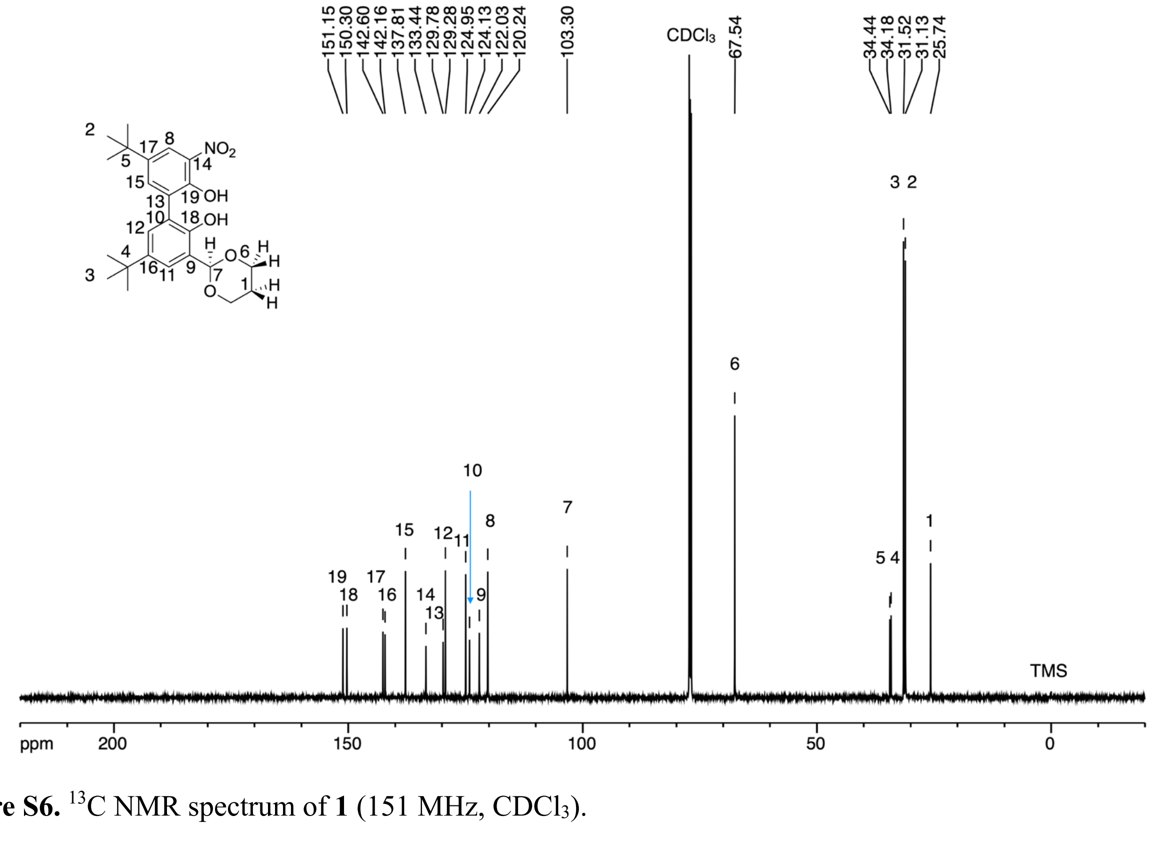

Figure S6. ^{13}C NMR spectrum of **1** (151 MHz, $CDCl_3$).

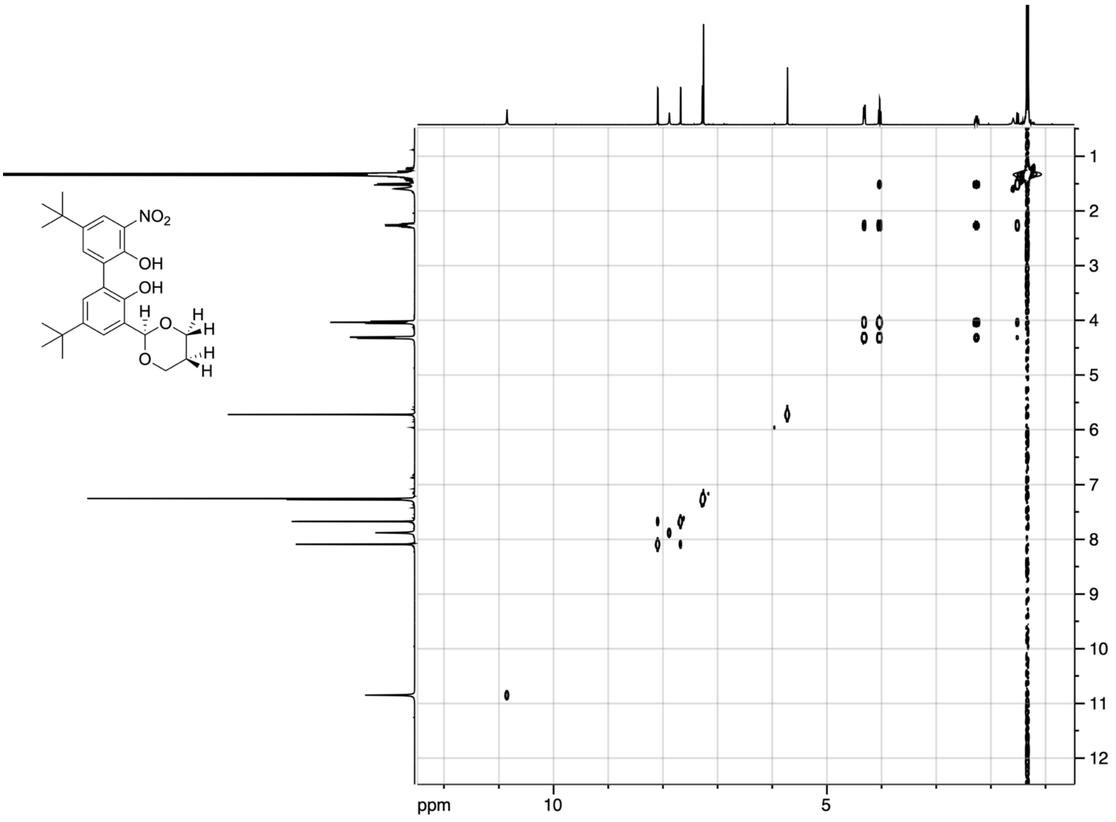

Figure S7. ^{1}H–^{1}H COSY spectrum of **1** (600 MHz, $CDCl_3$).

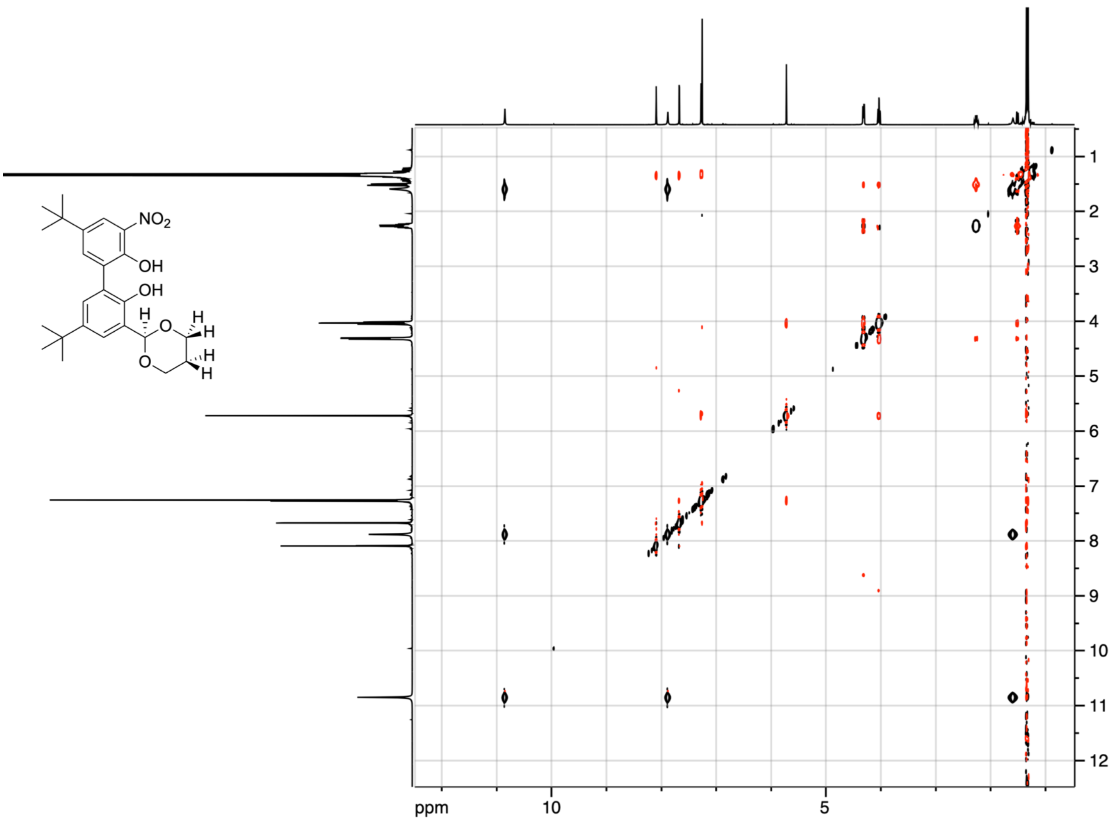

Figure S8. ^{1}H–^{1}H NOESY spectrum of **1** (600 MHz, $CDCl_3$).

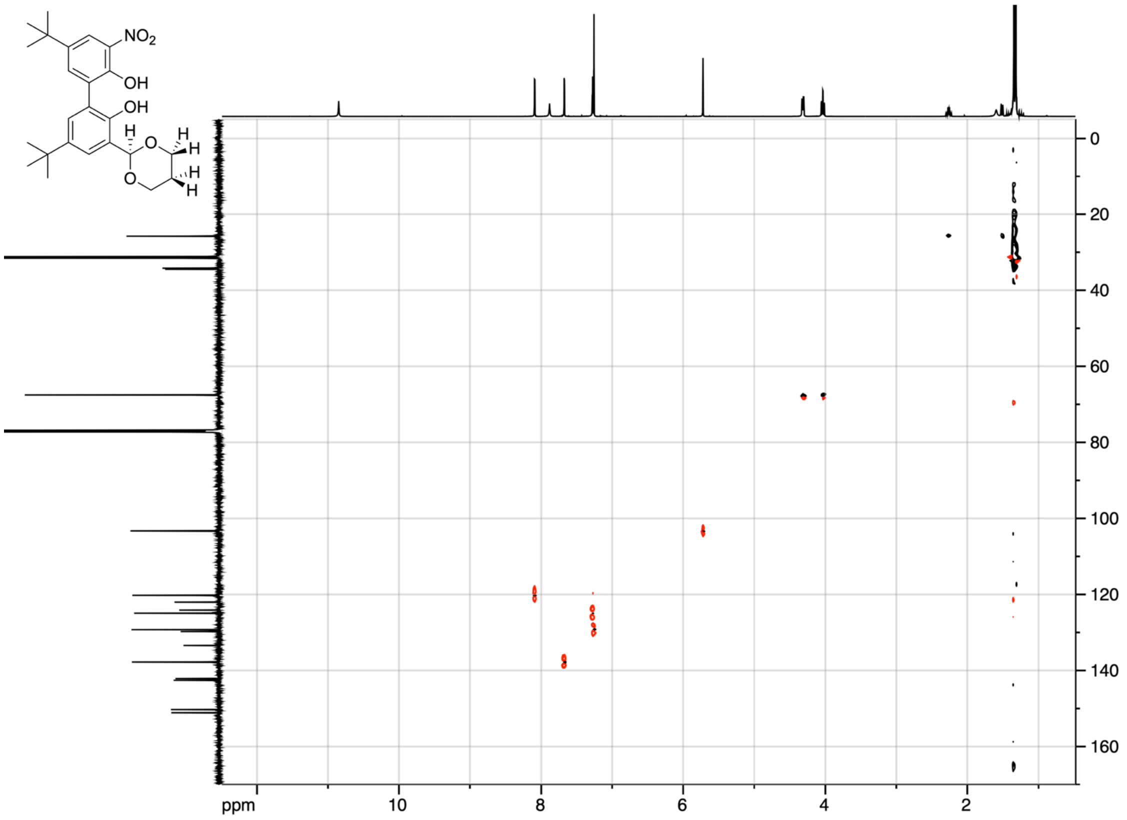

Figure S9. ^{1}H–^{13}C HSQC spectrum of **1** (600 MHz, $CDCl_3$).

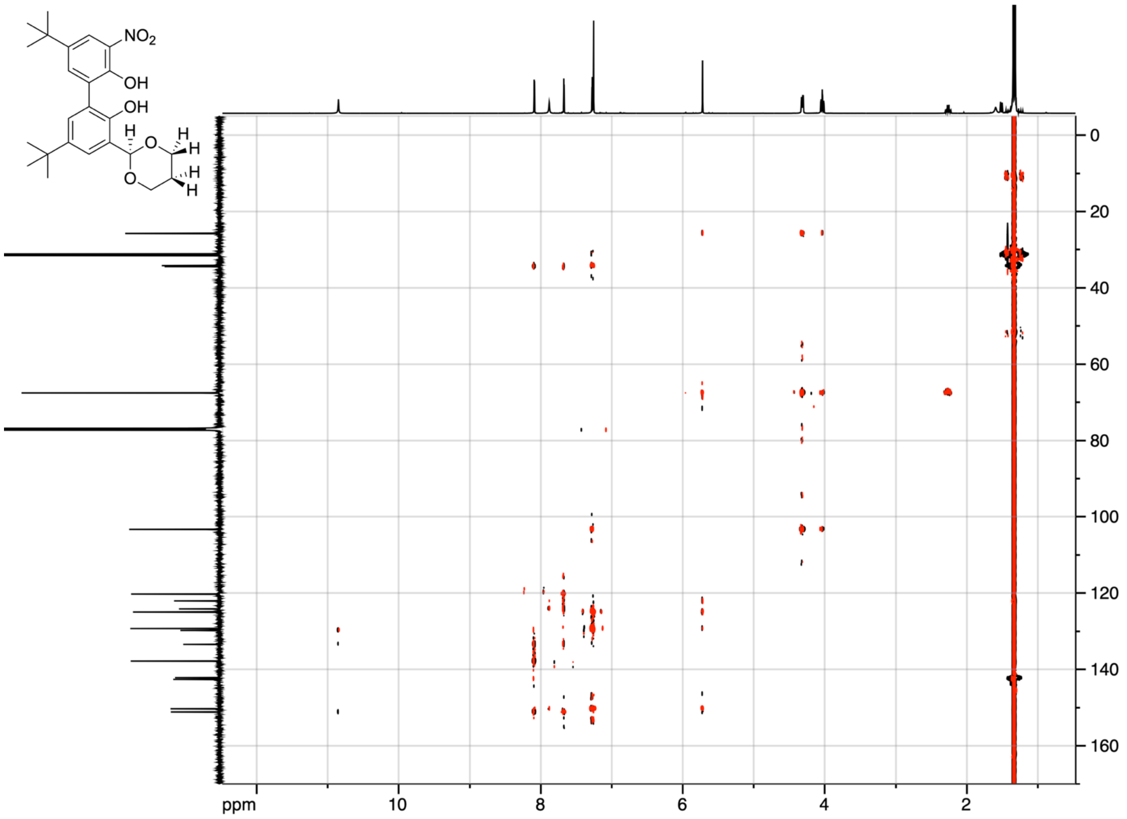

Figure S10. ^{1}H–^{13}C HMBC spectrum of **1** (600 MHz, $CDCl_3$).

2-2. Synthesis of tetrasap H_8**C4**

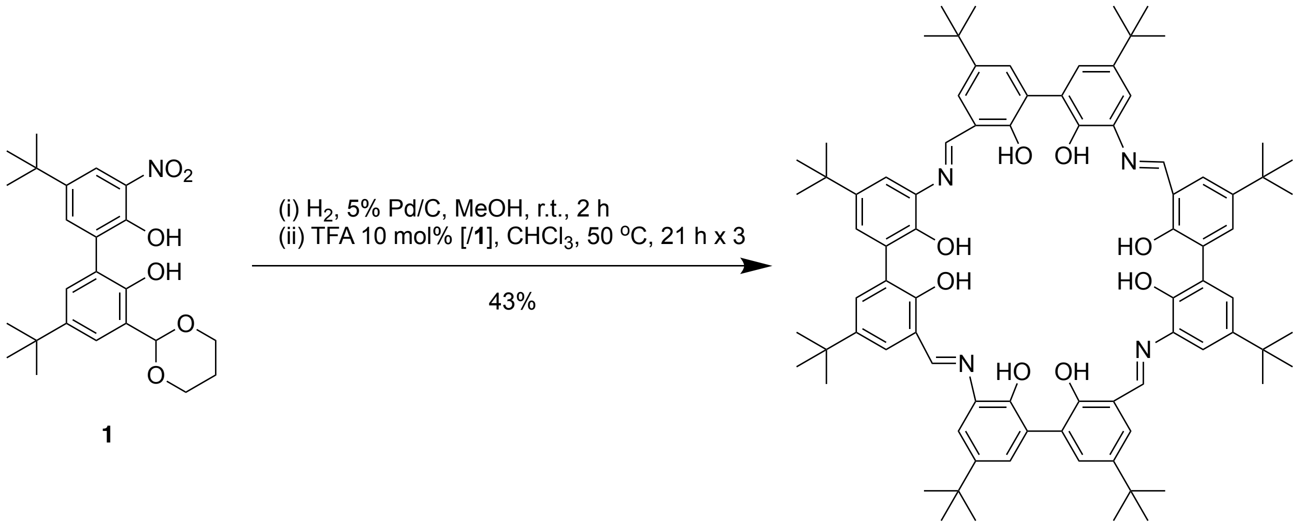

Tetrasap H_8**C4**

Synthesis of tetrasap H_8**C4**

In a 300 mL eggplant flask, a mixture of **1** (0.9637 g, 2.244 mmol, 1.0 equiv.) and 5% Pd/C (0.1937 g) in dry MeOH (100 mL) was stirred at r.t. for 2 h under a H_2 atmosphere. The reaction mixture was filtered and the filtrate was concentrated in vacuo. During the reduction of **1** (step (i)), the acetal group of **1** was partially deprotected and oligomers were produced (^{1}H NMR spectrum of the crude suggested that the cyclic trimer H_6**C3** was the major product). In a 50 mL Schlenk tube, to a mixture of the crude in $CHCl_3$ (9.0 mL) was added solution A (prepared by diluting TFA 75.7 mg in a 2 mL volumetric flask with $CHCl_3$) (675 μL, TFA 25.5 mg, 0.224 mmol, 10 mol%), and the mixture was stirred at 50 °C for 21 h under an Ar atmosphere. The reaction mixture was filtered to give H_8**C4** as an orange solid (0.1940 g). The filtrate was concentrated in vacuo, and the residue was added to $CHCl_3$ (5.0 mL) in a 50 mL Schlenk tube, then the mixture was stirred at 50 °C for 21 h under an Ar atmosphere. The reaction mixture was filtered to give H_8**C4** as an orange solid (0.0255 g). This operation was carried out again to give H_8**C4** as an orange solid (0.0925 g) (total: 0.3120 g, 0.2412 mmol, 43%).

^{1}H NMR (600 MHz, $CDCl_3$): δ 16.00 (d, J = 9.5 Hz, 4H), 10.63 (br, 4H), 8.49 (d, J = 9.5 Hz, 4H), 7.59 (d, J = 2.5 Hz, 4H), 7.35 (d, J = 2.2 Hz, 4H), 7.33 (d, J = 2.2 Hz, 4H), 7.31 (d, J = 2.5 Hz, 4H), 1.40 (s, 36H), 1.36 (s, 36H);

MALDI TOF MS: *m/z* calcd for $C_{84}H_{101}N_4O_8$ ([H_8**C4**+H]$^+$): 1293.76; found: 1293.78;

m.p.: over 280 °C;

Elemental analysis: calcd for $C_{84}H_{108}N_4O_{12}$ (H_8**C4**·4H_2O): C, 73.87; H, 7.97; N, 4.10. found: C, 73.83; H, 7.57; N, 4.11.

It was difficult to obtain a good ^{13}C NMR spectrum due to low solubility of H_8**C4**.

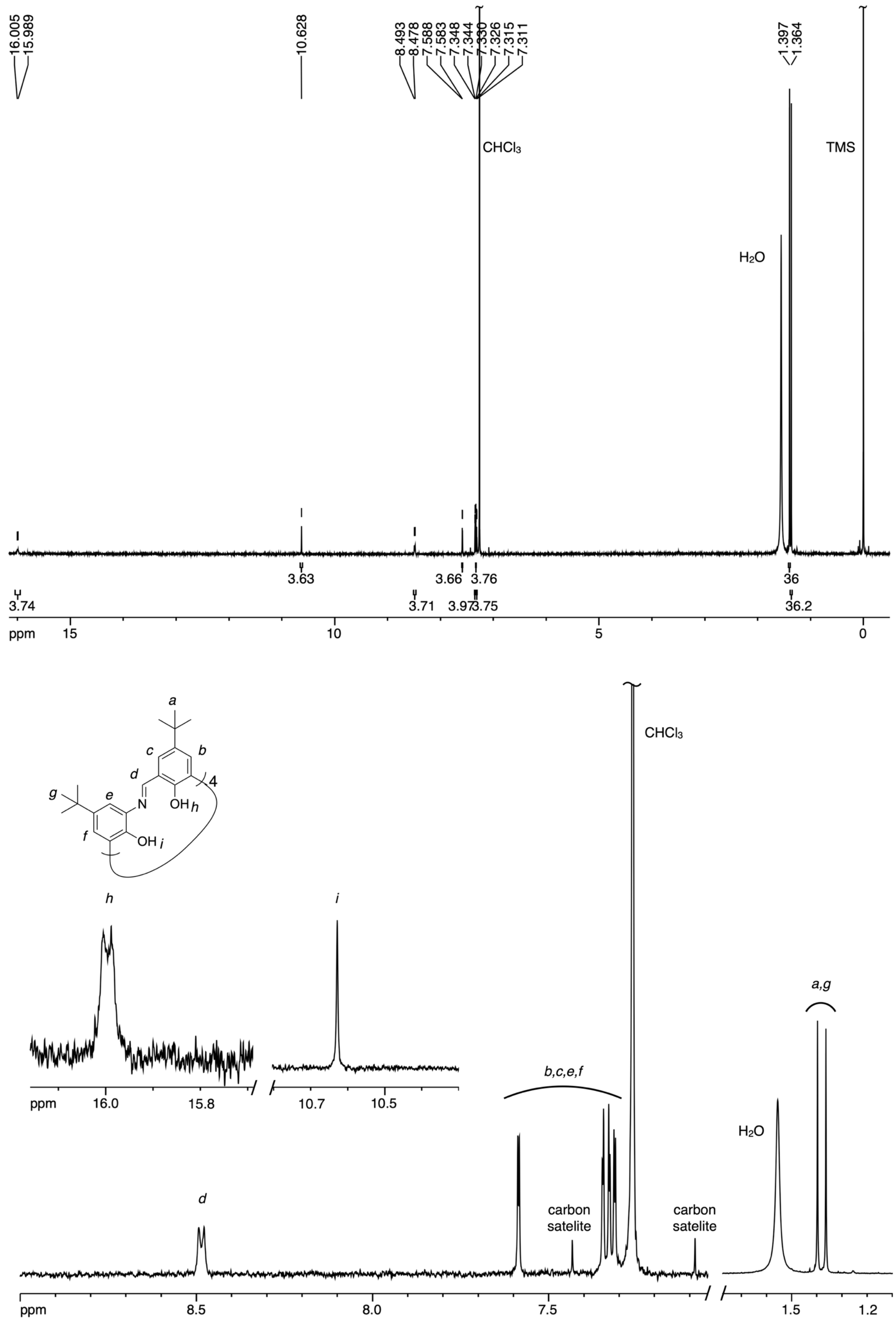

Figure S11. ^{1}H NMR spectrum of tetrasap H_8**C4** (600 MHz, $CDCl_3$).

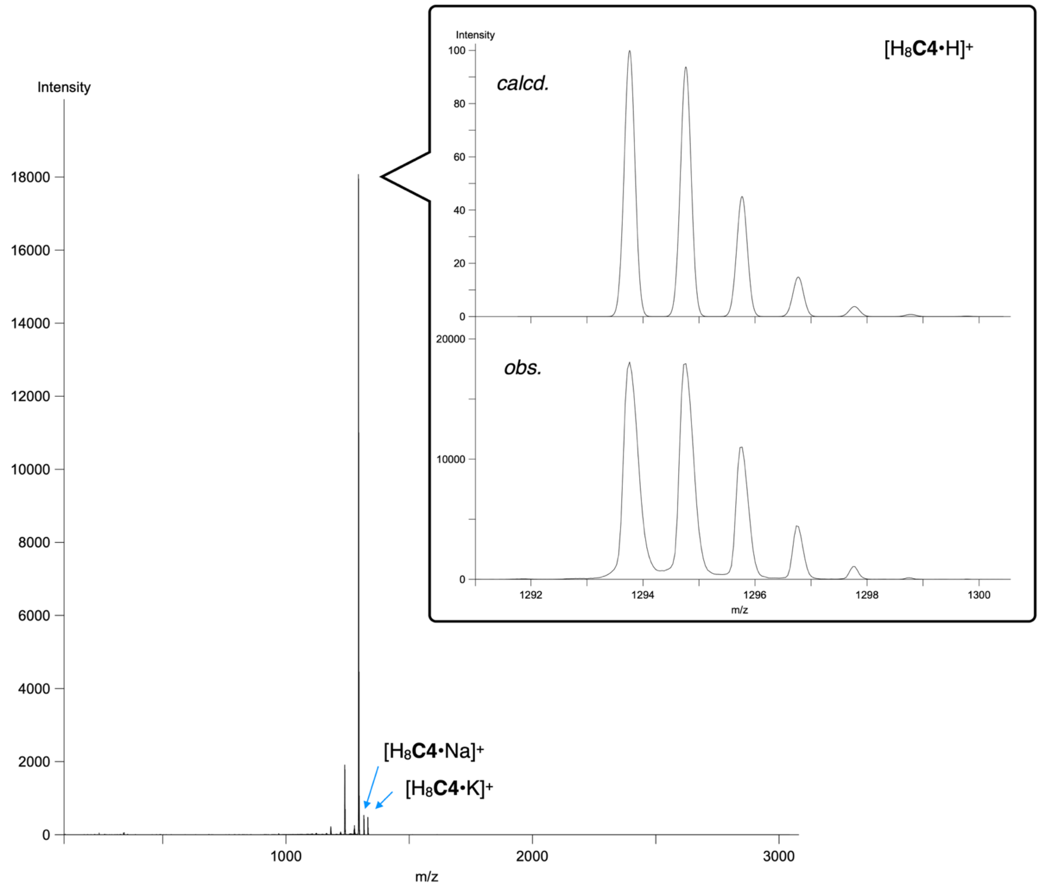

Figure S12. MALDI-TOF mass spectrum of tetrasap H_8**C4** (positive, matrix; dithranol).

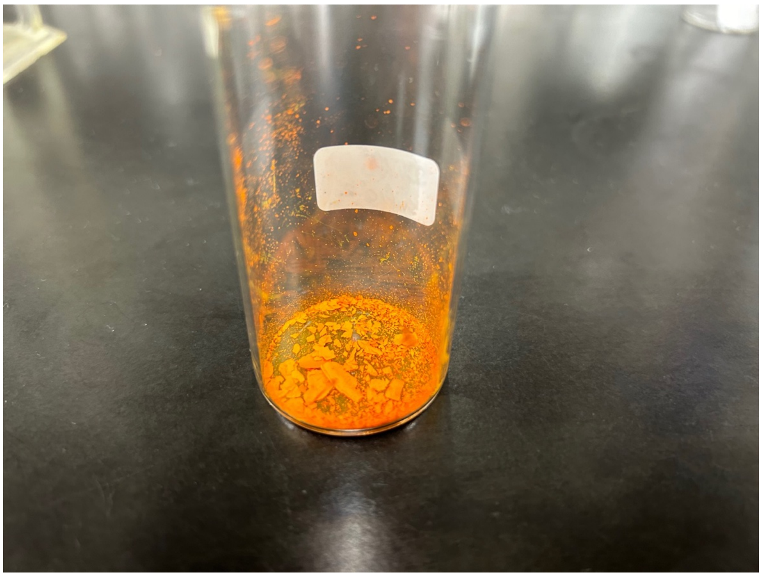

Figure S13. Appearance of tetrasap H_8**C4**.

3. Conversion from tetrasap H_8C4 to trisap H_6C3

3-1. Conversion from tetrasap H_8**C4** to trisap H_6**C3** by 1,1,2,2-tetrachloroethane

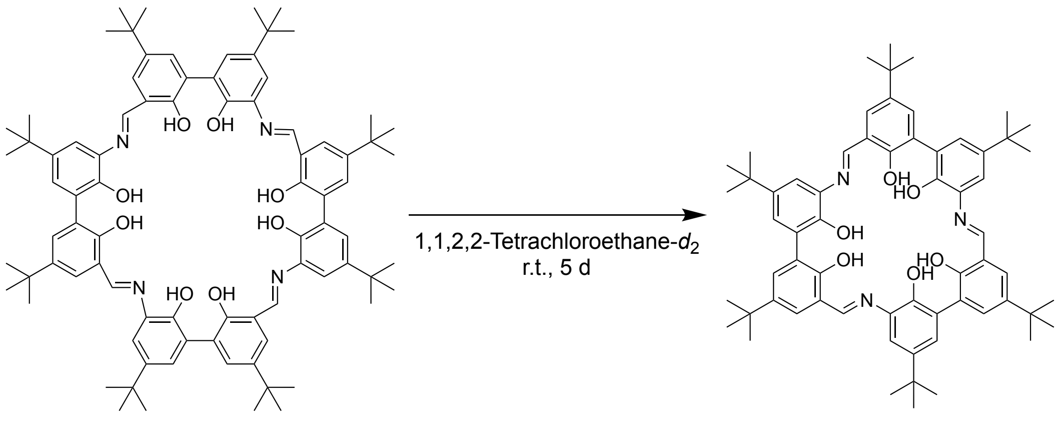

Tetrasap H_8**C4** Trisap H_6**C3**

In an NMR tube, 1,1,2,2-tetrachlotoethane-d_2 (500 μL) was added to H_8**C4** (1.14 mg, 0.881 μmol) and stood at r.t. for 5 d.

Large scale

In a 30 mL eggplant flask, a suspension of H_8**C4** 15.9 mg (12.3 μmol) in 1,1,2,2-tetrachloroethane 7.5 mL was stirred at 50 °C (bath temp.) for 4.5 h (the suspension changed into an orange solution). The solution was concentrated in vacuo to give H_6**C3** as a red solid (21.9 mg, quant.). H_6**C3** was measured by NMR in $CDCl_3$. The sample changed from an orange solution to an orange suspension over time, which suggested that some H_6**C3** converted to H_8**C4**. The suspension was concentrated in vacuo and same conversion experiment was carried out. The residue was purified by reprecipitation and decantation with 1,1,2,2-tetrachloroethane/hexane to give H_6**C3** as an orange solid (9.0 mg, 9.3 μmol, 56%).

Trisap H_6**C3**

^{1}H NMR (600 MHz, $CDCl_3$): δ 17.43 (d, J = 12.4 Hz, 3H), 12.57 (br, 3H), 8.65 (d, J = 12.4 Hz, 3H), 7.85 (d, J = 2.4 Hz, 3H), 7.52 (d, J = 2.0 Hz, 3H), 7.49 (d, J = 2.0 Hz, 3H), 7.39 (d, J = 2.4 Hz, 3H), 1.43 (s, 27H), 1.41 (s, 27H);

^{1}H NMR (600 MHz, 1,1,2,2-tetrachloroethane-d_2): δ 17.35 (d, J = 12.6 Hz, 3H), 12.77 (br, 3H), 8.68 (d, J = 12.6 Hz, 3H), 7.91 (d, J = 2.3 Hz, 3H), 7.57 (d, J = 1.8 Hz, 3H), 7.53 (d, J = 1.8 Hz, 3H), 7.46 (d, J = 2.3 Hz, 3H), 1.46 (s, 27H), 1.44 (s, 27H);

^{13}C NMR (151 MHz, $CDCl_3$): δ 171.0, 154.5, 148.2, 142.7, 138.2, 136.3, 131.7, 129.2, 128.9, 128.2, 127.2, 116.2, 111.3, 34.6, 34.1, 31.7, 31.4;

^{13}C NMR (151 MHz, 1,1,2,2-tetrachloroethane-d_2): δ 170.5, 154.7, 147.6, 143.0, 138.4, 136.4, 131.0, 129.1, 128.8, 127.8, 127.3, 116.0, 111.5, 34.4, 33.9, 31.5, 31.2;

MALDI TOF MS: m/z calcd for $C_{63}H_{76}N_3O_6$ ([H_6**C3**+H]$^+$): 970.57; found: 970.59.

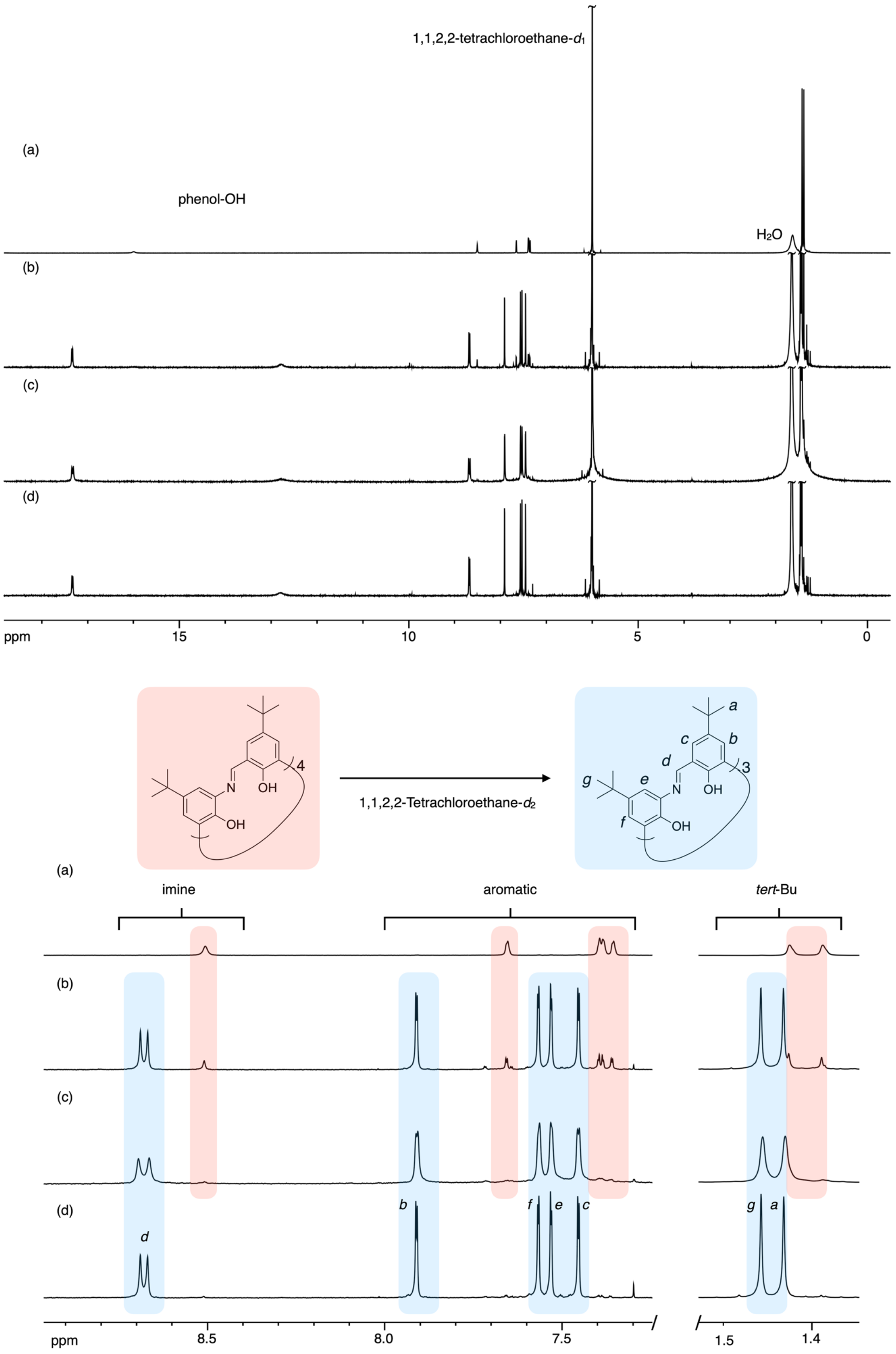

Figure S14. ^{1}H NMR spectra of conversion from tetrasap H_8**C4** to trisap H_6**C3** (1,1,2,2-tetracholoethane-d_2). (a) H_8**C4** (500 MHz). (b) Stood for 1 day (600 MHz). (c) Stood for 2 days (400 MHz). (d) Stood for 5 days (600 MHz). Appearance of the sample; (a) orange suspension, (b–d) orange solution.

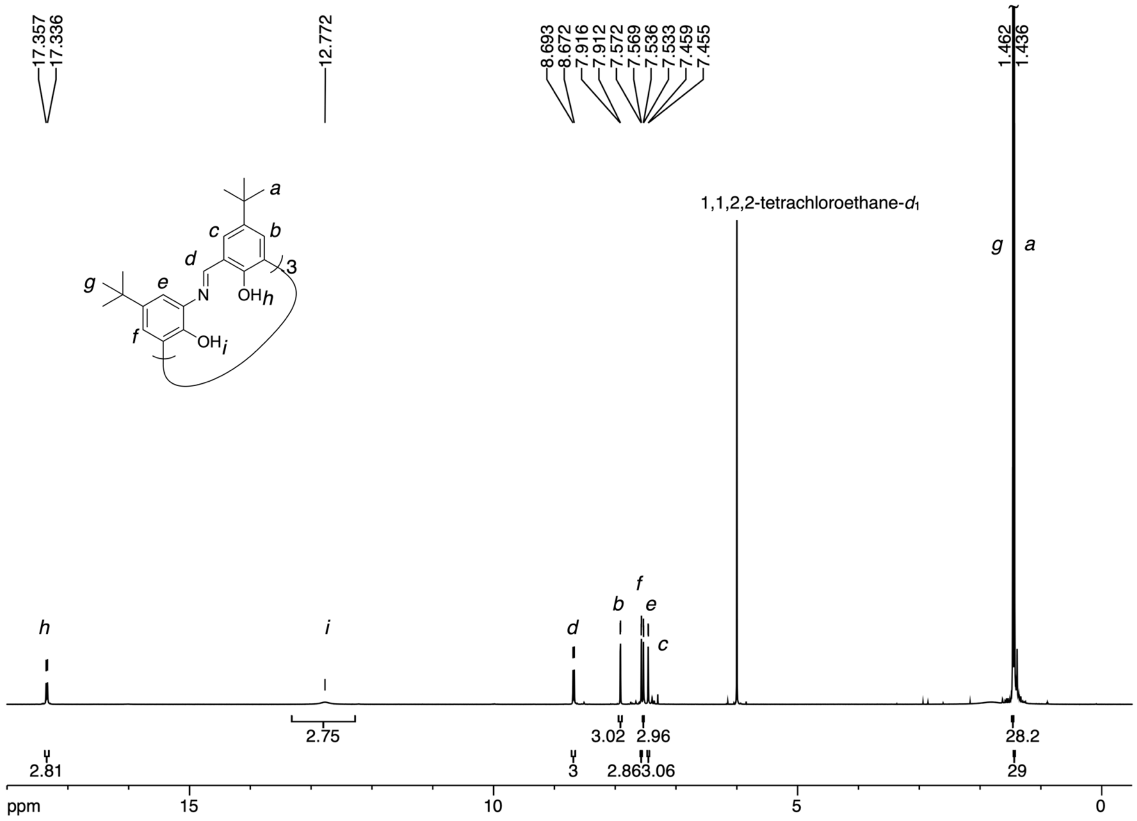

Figure S15. ^{1}H NMR spectrum of H_6**C3** (600 MHz, 1,1,2,2-tetracholoroethane-d_2).

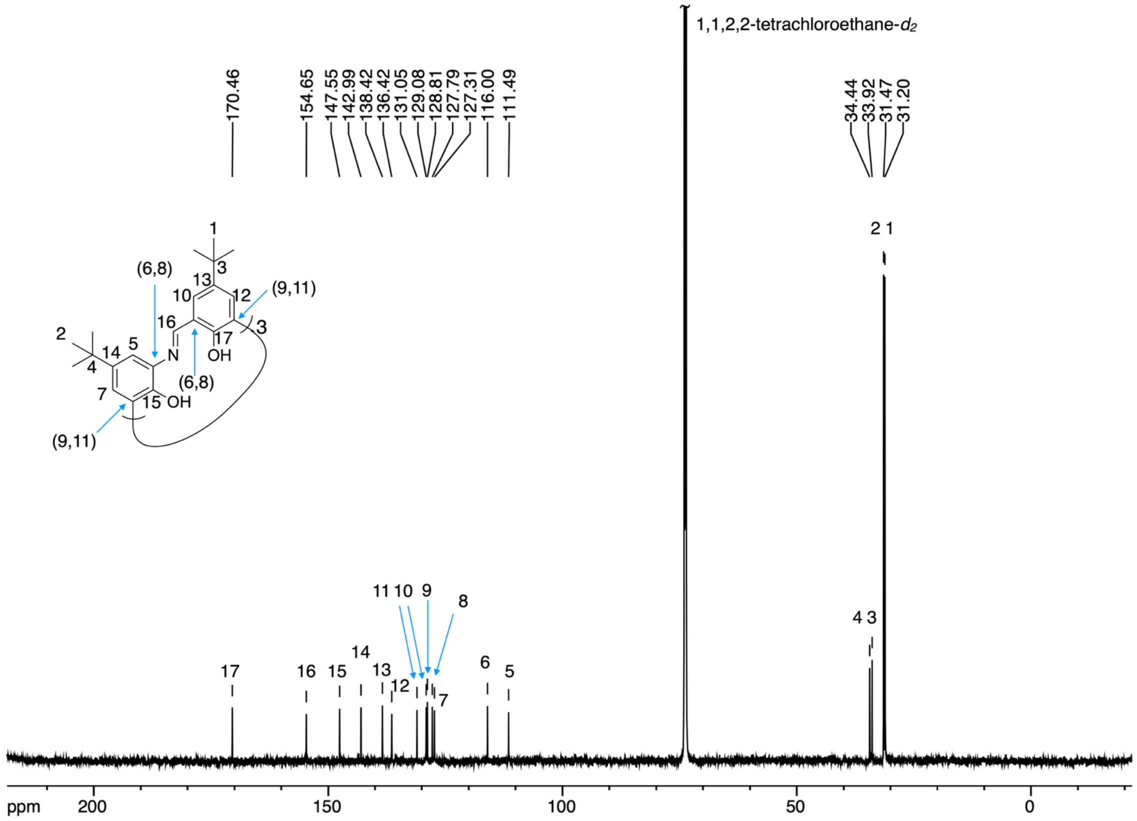

Figure S16. ^{13}C NMR spectrum of H_6**C3** (151 MHz, 1,1,2,2-tetracholoroethane-d_2).

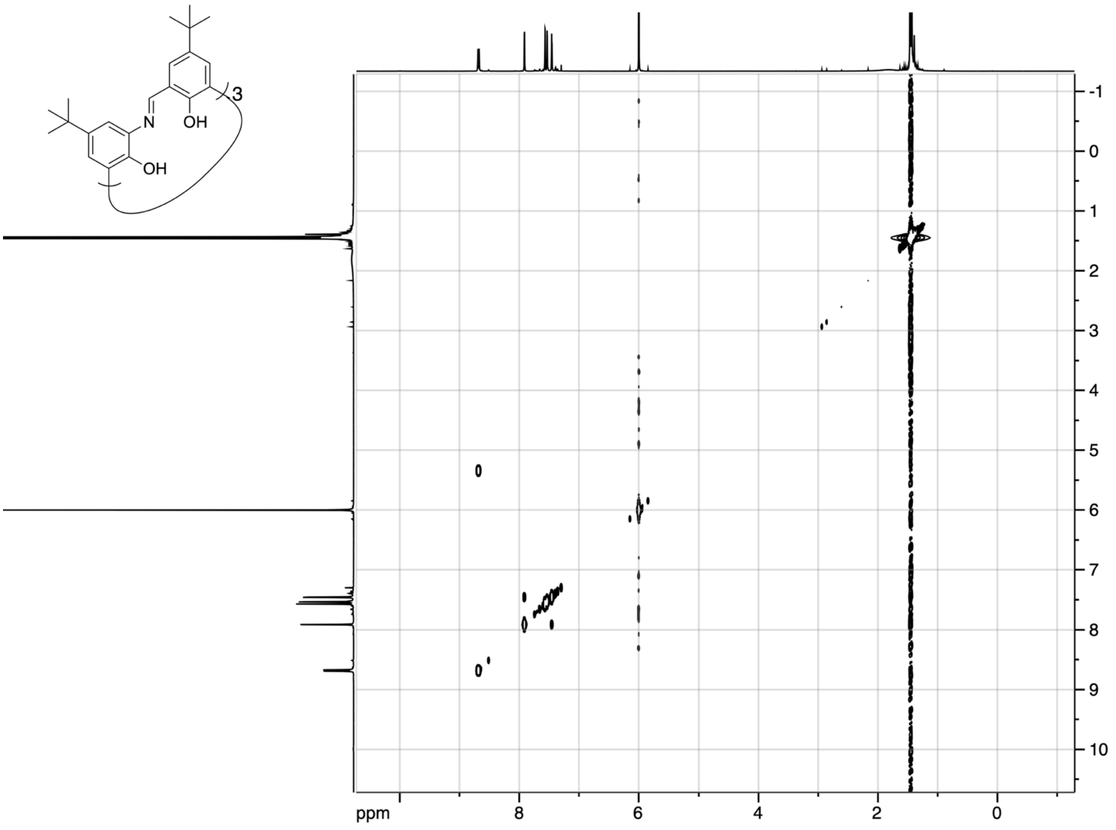

Figure S17. ^{1}H–^{1}H COSY spectrum of H_6**C3** (600 MHz, 1,1,2,2-tetracholoroethane-d_2).

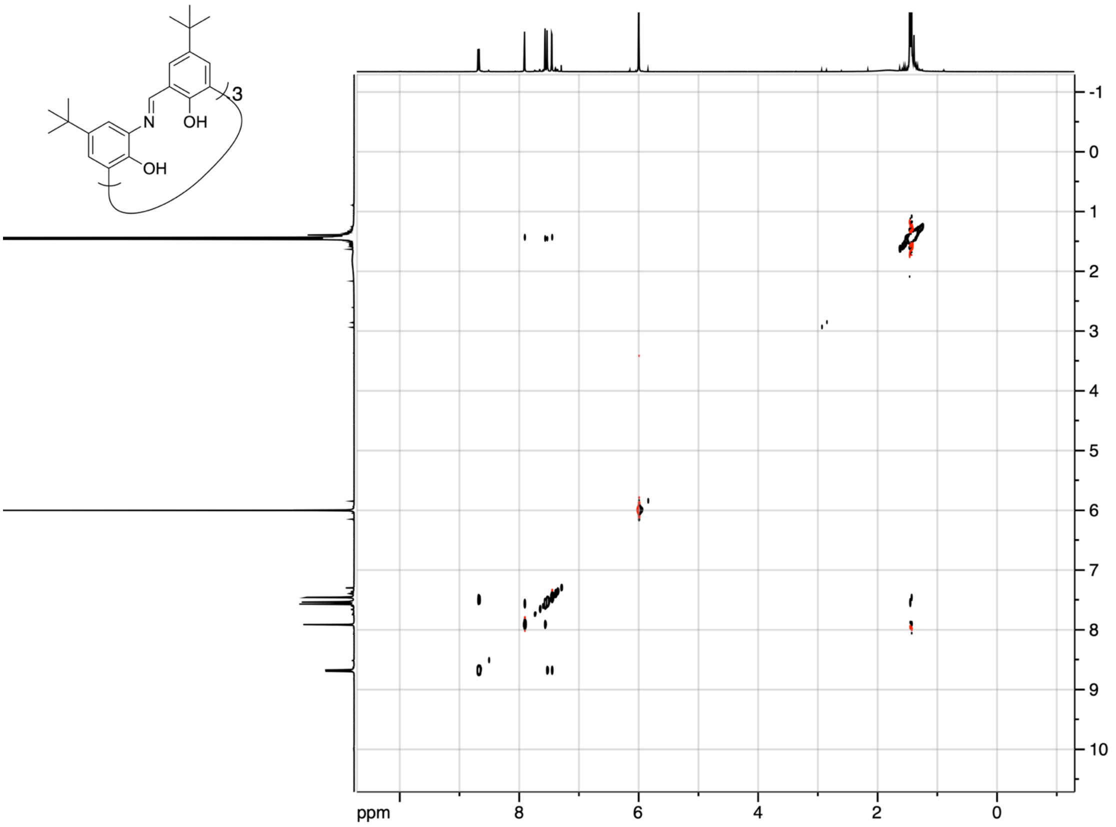

Figure S18. ^{1}H–^{1}H NOESY spectrum of H_6**C3** (600 MHz, 1,1,2,2-tetracholoroethane-d_2).

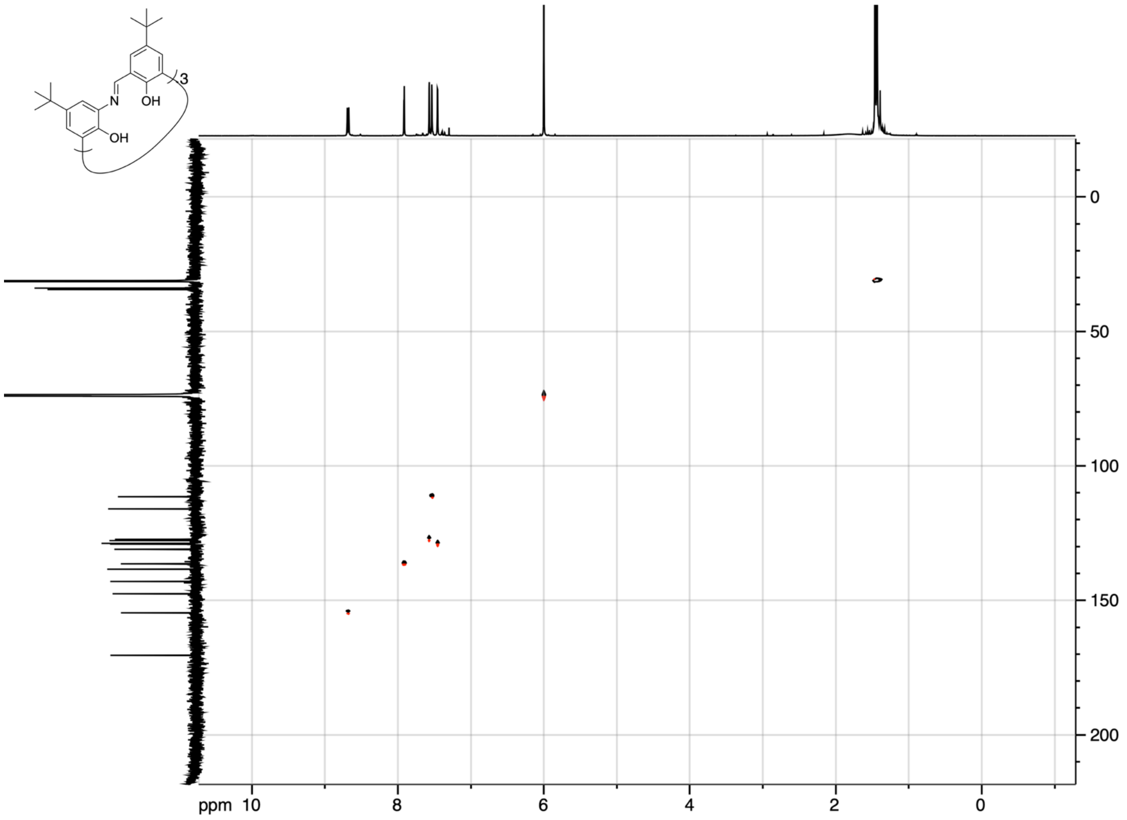

Figure S19. ^{1}H–^{13}C HSQC spectrum of H_6**C3** (600 MHz, 1,1,2,2-tetracholoroethane-d_2).

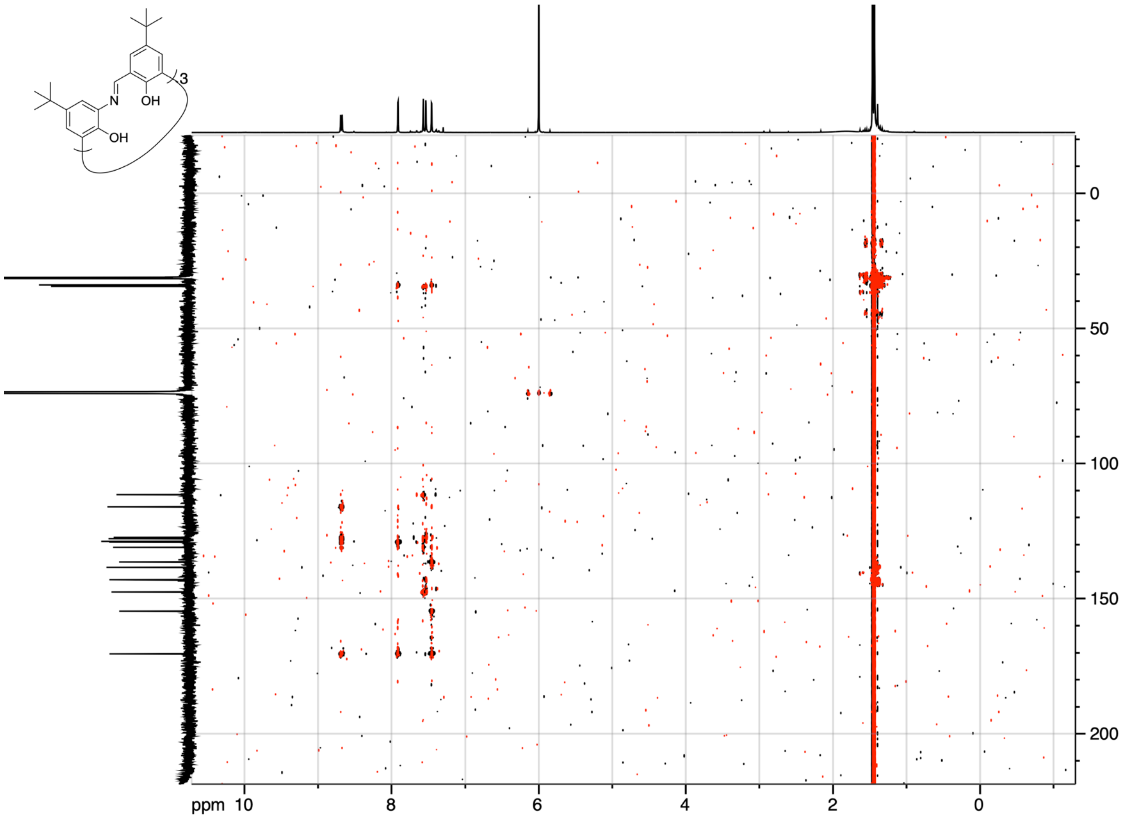

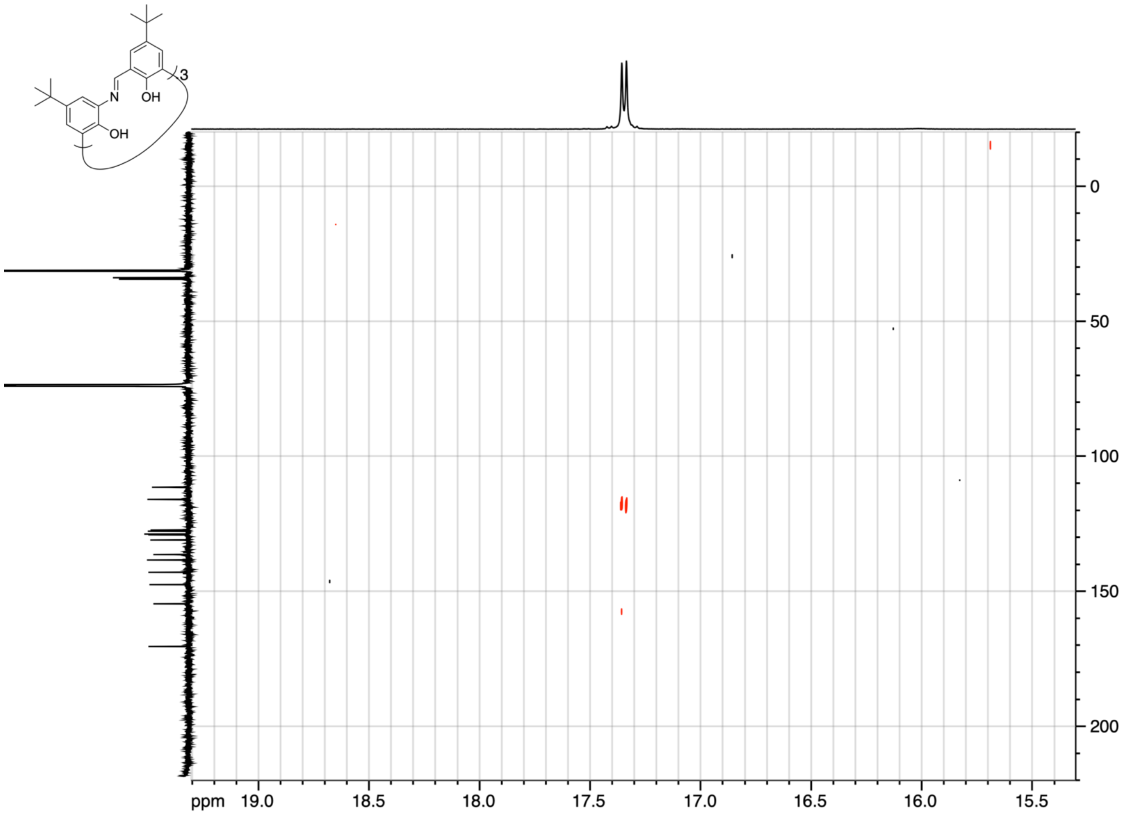

Figure S20. ^{1}H–^{13}C HMBC spectrum of H_6**C3** (600 MHz, 1,1,2,2-tetracholoroethane-d_2).

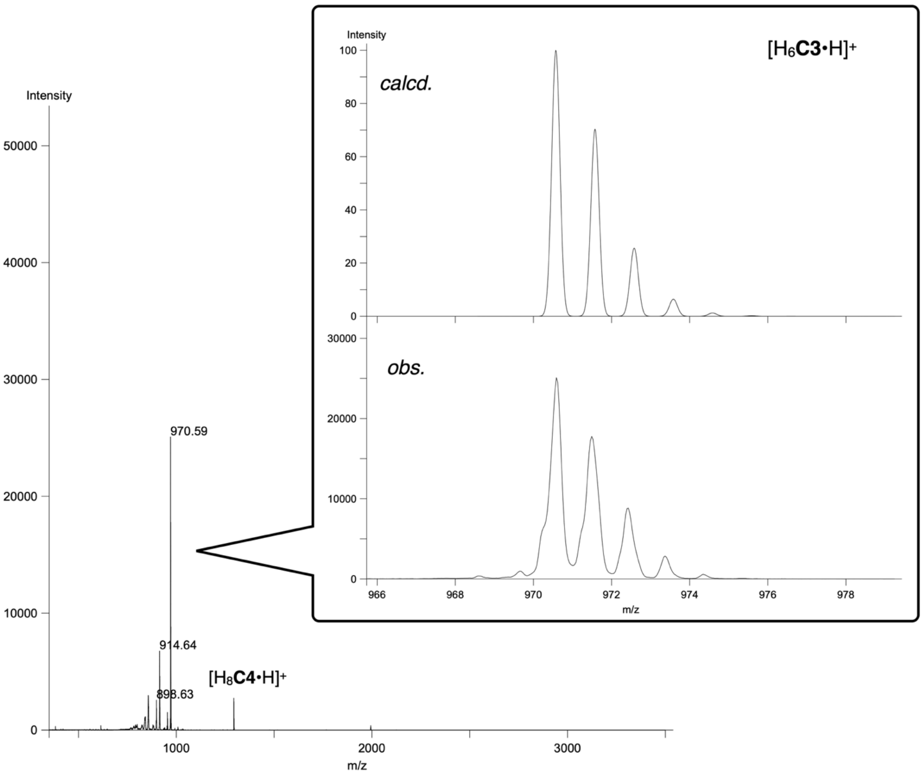

Figure S21. MALDI-TOF mass spectrum of H_6**C3** (positive, matrix; dithranol).

3-2. Behavior of tetrasap H_8**C4** in $CDCl_3$

The NMR sample of H_8**C4** in $CDCl_3$ (approximately 4.9 mg/500 μL) was prepared. ^{1}H NMR spectra were measured just after the addition of $CDCl_3$ and after 7 h. The appearances of both samples were orange suspension.

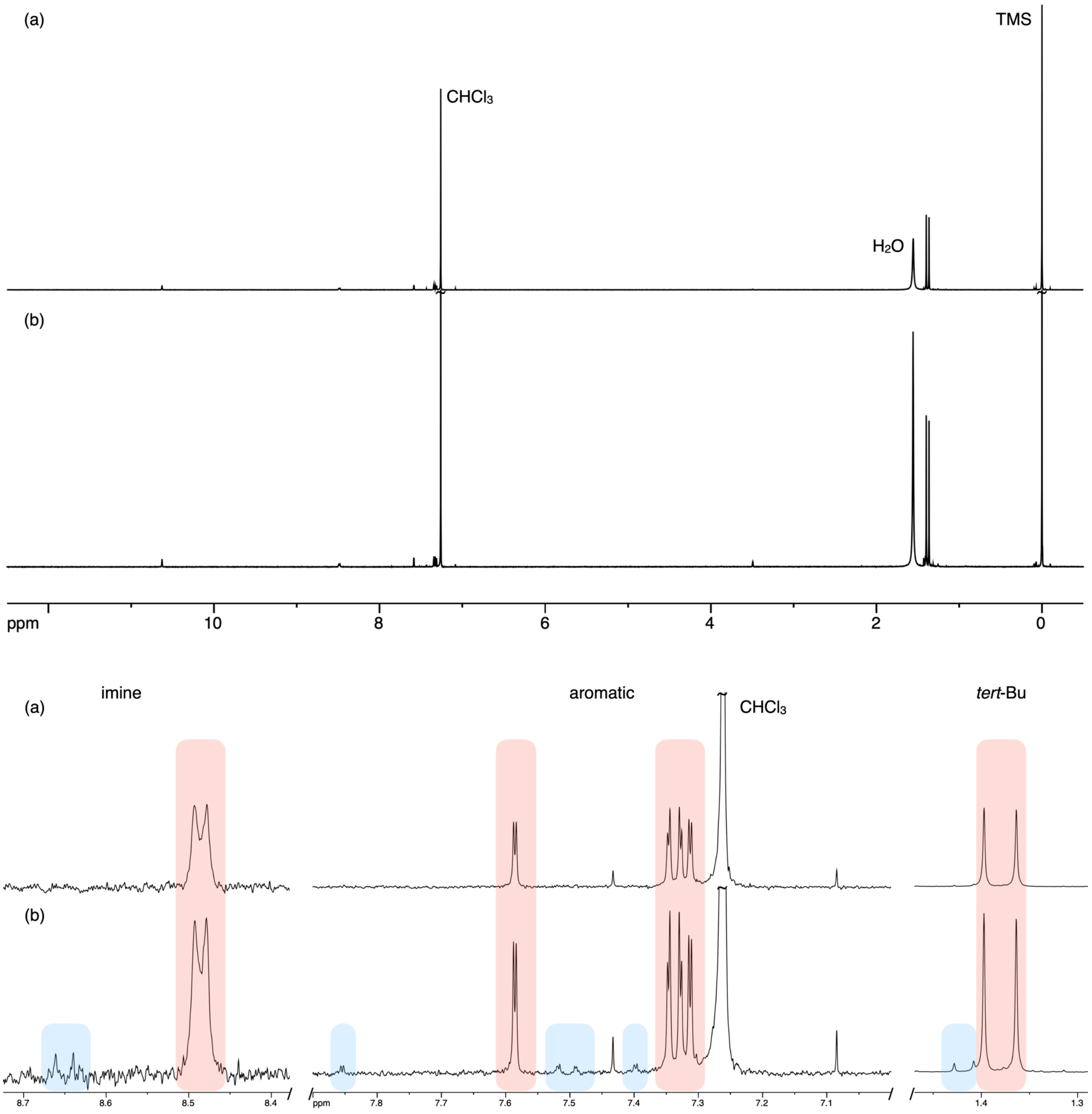

Figure S22. ^{1}H NMR spectra of behavior of tetrasap H_8**C4** in $CDCl_3$ (600 MHz). (a) After addition of $CDCl_3$. (b) Stood for 7 h. Highlight in red; H_8**C4**, in blue; H_6**C3**.

4. Formation of tetranuclear palladium complex [C4Pd4L4]

4-1. Synthesis of [**C4**Pd_4L_4]

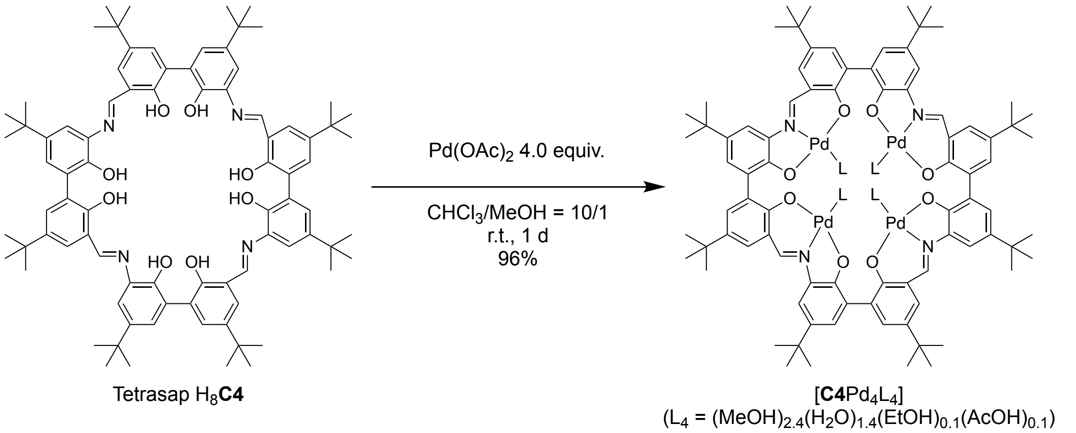

$Pd(OAc)_2$ (79.12 mg, 352.4 μmol, 4.0 equiv.) was dissolved in $CHCl_3$/MeOH = 10/1 (4 mL) in a sample tube, and the solution was added to a mixture of H_8**C4** (0.1140 g, 88.12 μmol, 1.0 equiv.) in $CHCl_3$/MeOH = 10/1 (45 mL) in a 100 mL eggplant flask. The sample tube was rinsed with $CHCl_3$/MeOH = 10/1 (1 mL×5), and the washings were added to the flask to ensure complete transfer. The mixture was stirred at r.t. for 1 d. The reaction mixture was concentrated in vacuo. MeOH (3 mL) was added to the residue, and the mixture was then concentrated in vacuo. This operation was carried out eight times to give [**C4**Pd_4L_4]•$8H_2O$ as an orange solid (0.1666 g, 84.6 μmol, 96%).

(L_4 = $(MeOH)_{2.4}(H_2O)_{1.4}(EtOH)_{0.1}(AcOH)_{0.1}$ determined by the integration of ^{1}H NMR and elemental analysis.)

(EtOH was a stabilizer of $CHCl_3$.)

^{1}H NMR (600 MHz, $CDCl_3/CD_3OD$ = 10/1): δ 8.18 (s, 4H), 7.65 (d, *J* = 2.1 Hz, 4H), 7.50 (d, *J* = 2.6 Hz, 4H), 7.44 (d, *J* = 2.6 Hz, 4H), 7.09 (d, *J* = 2.1 Hz, 4H), 1.39 (s, 36H), 1.36 (s, 36H);

^{13}C NMR (151 MHz, $CDCl_3/CD_3OD$ = 10/1): δ 160.8, 159.0, 146.5, 138.71, 138.69, 138.3, 134.5, 131.0, 130.8, 129.4, 128.6, 121.1, 110.4, 34.4, 34.0, 32.0, 31.5;

m.p.: 124 °C (decomp.);

Elemental analysis for $C_{86.8}H_{121.4}N_4O_{20.1}Pd_4$ ([**C4**$Pd_4(MeOH)_{2.4}(H_2O)_{1.4}(EtOH)_{0.1}(AcOH)_{0.1}$]•$8H_2O$) calcd.: C, 52.97; H, 6.22; N, 2.85. found: C, 52.76; H, 5.87; N, 3.06.

[**C4**$Pd_4(EtOH)_2(AcOH)_2$] was obtained with the same procedure except for the omission of evaporation from MeOH solution, and [**C4**$Pd_4(MeOH)_{3.0}(H_2O)_{0.9}(AcOH)_{0.1}$] was obtained by the same procedure.

Large scale

$Pd(OAc)_2$ (211.07 mg, 940.1 μmol, 4.0 equiv.) was dissolved in $CHCl_3$/MeOH = 10/1 (6 mL) in a sample tube, and the solution was added to a mixture of H_8**C4** (0.3039 g, 234.9 μmol, 1.0 equiv.) in $CHCl_3$/MeOH = 10/1 (100 mL) in a 500 mL eggplant flask. The sample tube was rinsed with $CHCl_3$/MeOH = 10/1 (2 mL×10), and the washings were added to the flask to ensure complete transfer. An additional $CHCl_3$/MeOH = 10/1 (30 mL) was added, and the mixture was stirred at r.t. for 1 d. The reaction mixture was filtered and the filtrate was then concentrated in vacuo. MeOH (6 mL) was added to the residue, and the mixture was concentrated in vacuo. This operation was carried out three times. The residue was washed by MeOH (8 mL) to give [**C4**Pd_4L_4] as an orange solid (0.3605 g, 197.5 μmol, 84%).
(L_4 = $(MeOH)_{2.5}(H_2O)_{1.3}(EtOH)_{0.1}(AcOH)_{0.1}$ determined by the integration of ^{1}H NMR.)
(EtOH was a stabilizer of $CHCl_3$.)

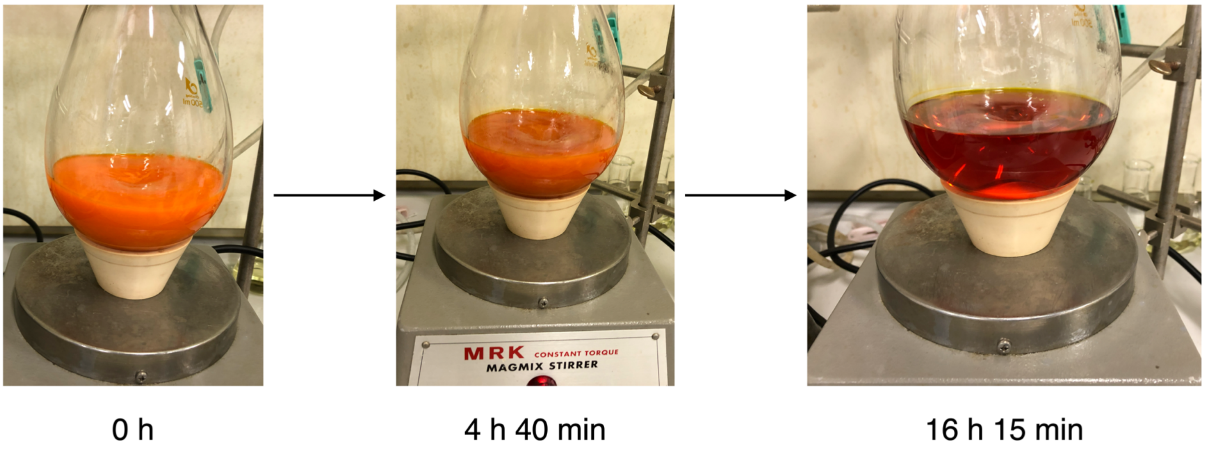

Figure S23. Appearance of the complexation reaction.

[**C4**$Pd_4(MeOH)_{2.0}(H_2O)_{2.0}$] was synthesized as follows.

<u>Synthesis of [**C4**$Pd_4(MeOH)_{2.0}(H_2O)_{2.0}$]</u>

$Pd(OAc)_2$ (0.2150 g, 957.6 μmol, 4.0 equiv.) was dissolved in $CHCl_3$/MeOH = 10/1 (6 mL) in a sample tube, and the solution was added to a mixture of H_8**C4** (0.3092 g, 239.0 μmol, 1.0 equiv.) in $CHCl_3$/MeOH = 10/1 (100 mL) in a 500 mL eggplant flask. The sample tube was rinsed with $CHCl_3$/MeOH = 10/1 (2 mL × 5), and the washings were added to the flask to ensure complete transfer. An additional $CHCl_3$/MeOH = 10/1 (34 mL) was added, and the mixture was stirred at r.t. for 1 d. and at 50 °C (bath temp.) for 13 h. The reaction mixture was concentrated in vacuo, then the residue was purified by reprecipitation with $CHCl_3$ and MeOH. $CHCl_3$ and MeOH were added to the solid, followed by several filtration to remove palladium black. A solid precipitated during the evaporation of the filtrate was filtered to give [**C4**$Pd_4(MeOH)_{2.0}(H_2O)_{2.0}$]•13$H_2O$ as an orange solid (0.1103 g, 53.9 μmol, 22%).
Elemental analysis for $C_{86}H_{130}N_4O_{25}Pd_4$ ([**C4**$Pd_4(MeOH)_{2.0}(H_2O)_{2.0}$]•13$H_2O$) calcd.: C, 50.49; H, 6.41; N, 2.74. found: C, 50.61; H, 6.21; N, 2.73.

4-2. NMR assignment of [**C4**Pd_4L_4]

For the assignment of ^{1}H NMR and ^{13}C NMR signals, [**C4**$Pd_4(EtOH)_2(AcOH)_2$] was used.

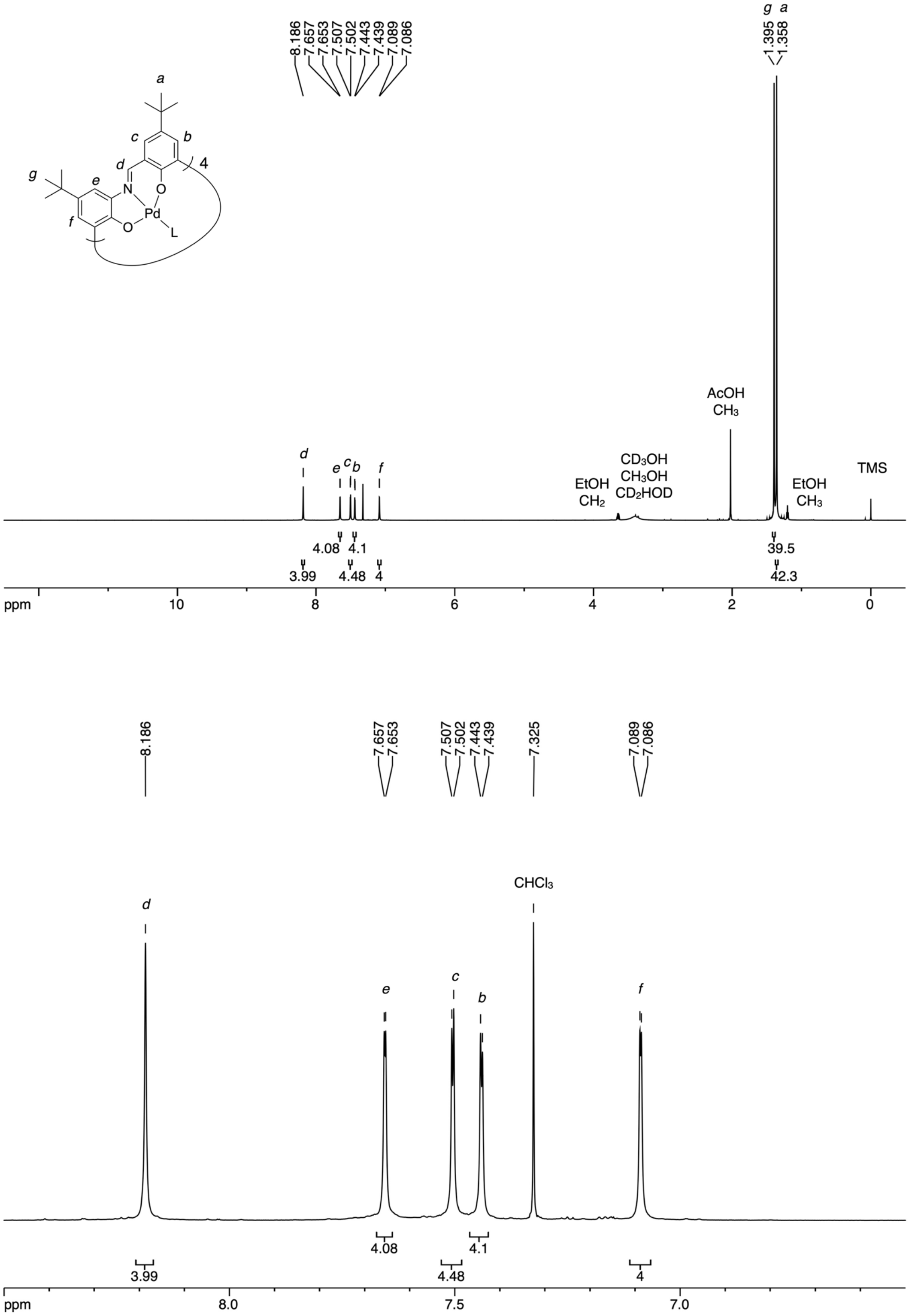

Figure S24. ^{1}H NMR spectrum of [**C4**Pd_4L_4] (600 MHz, $CDCl_3/CD_3OD$ = 10/1).

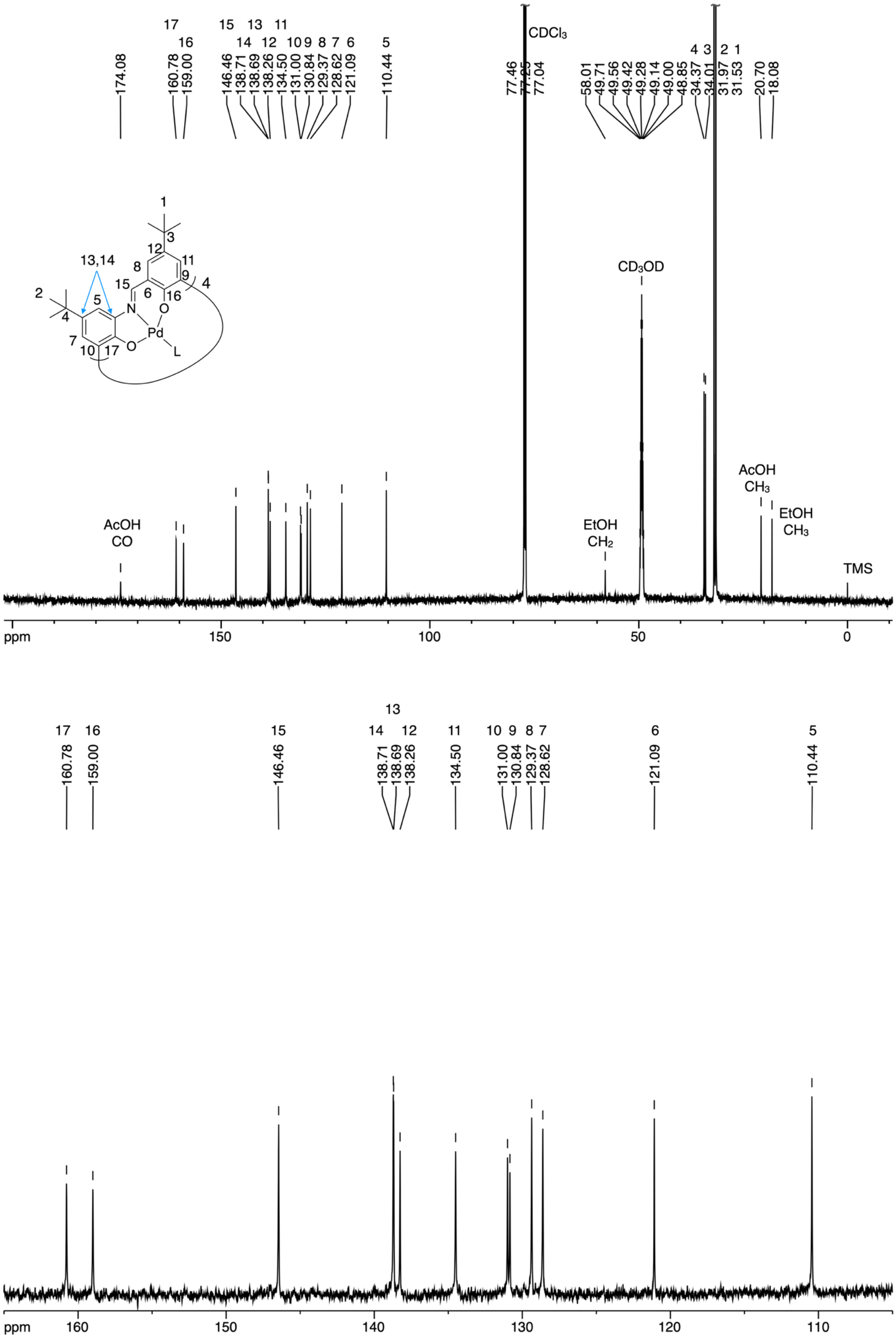

Figure S25. ^{13}C NMR spectrum of [**C4**Pd_4L_4] (151 MHz, $CDCl_3$/CD_3OD = 10/1).

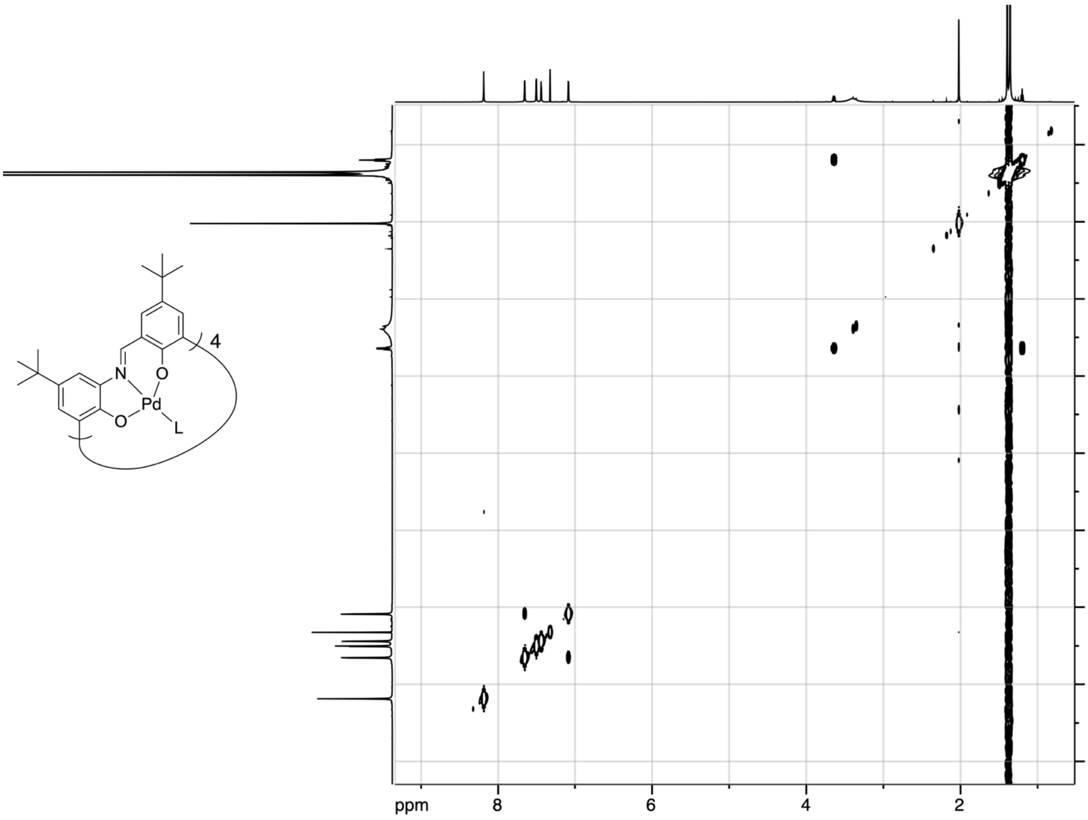

Figure S26. ^{1}H–^{1}H COSY NMR spectrum of [**C4**Pd_4L_4] (600 MHz, $CDCl_3/CD_3OD$ = 10/1).

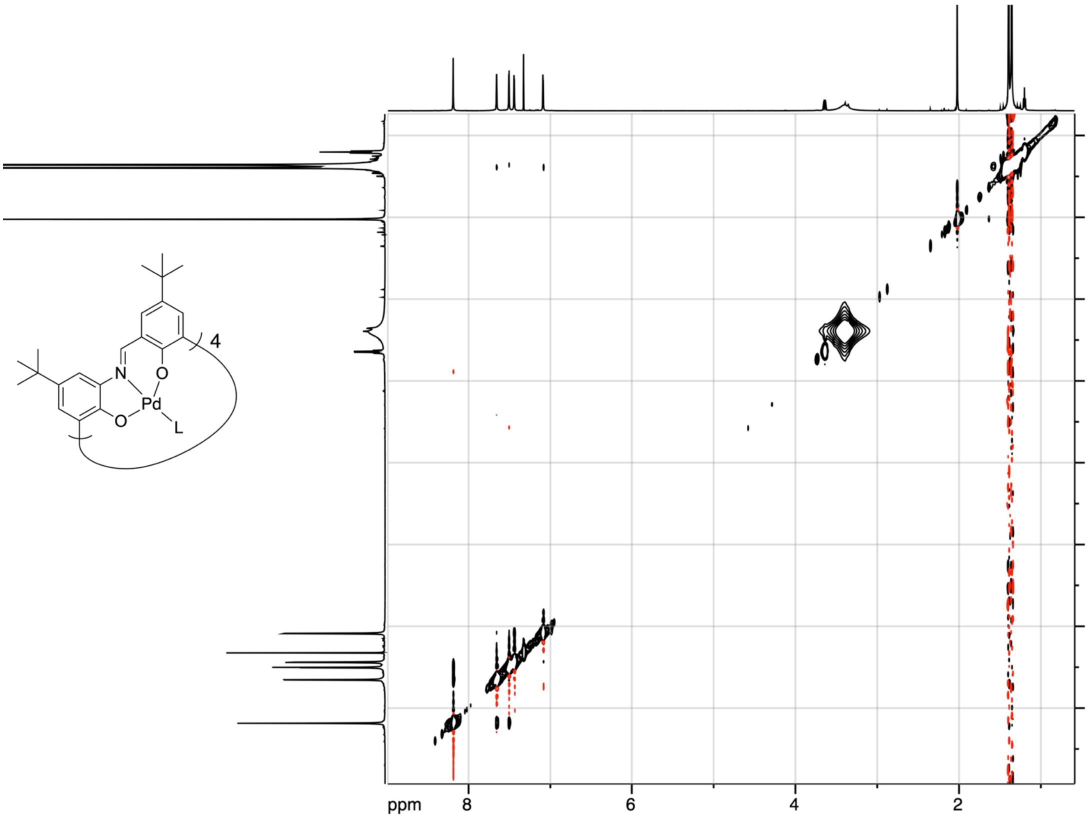

Figure S27. ^{1}H–^{1}H NOESY NMR spectrum of [**C4**Pd_4L_4] (600 MHz, $CDCl_3/CD_3OD$ = 10/1).

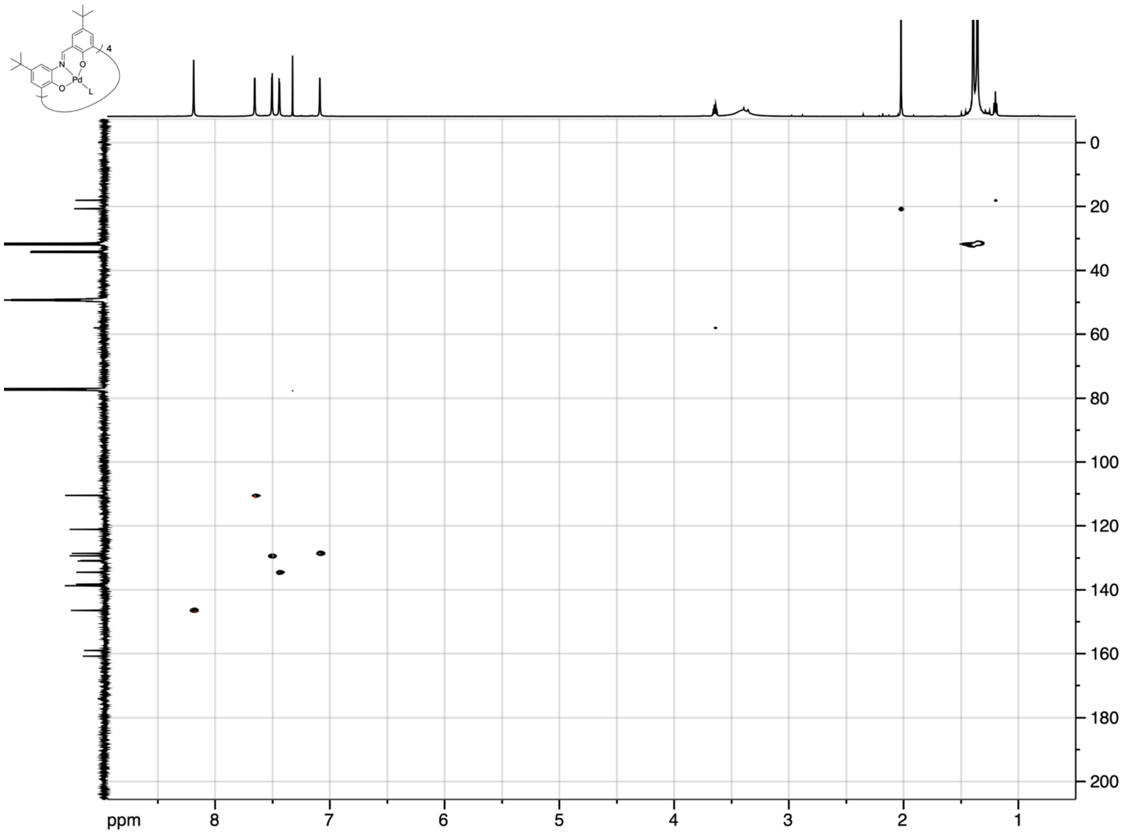

Figure S28. ^{1}H–^{13}C HSQC NMR spectrum of [**C4**Pd_4L_4] (600 MHz, $CDCl_3/CD_3OD$ = 10/1).

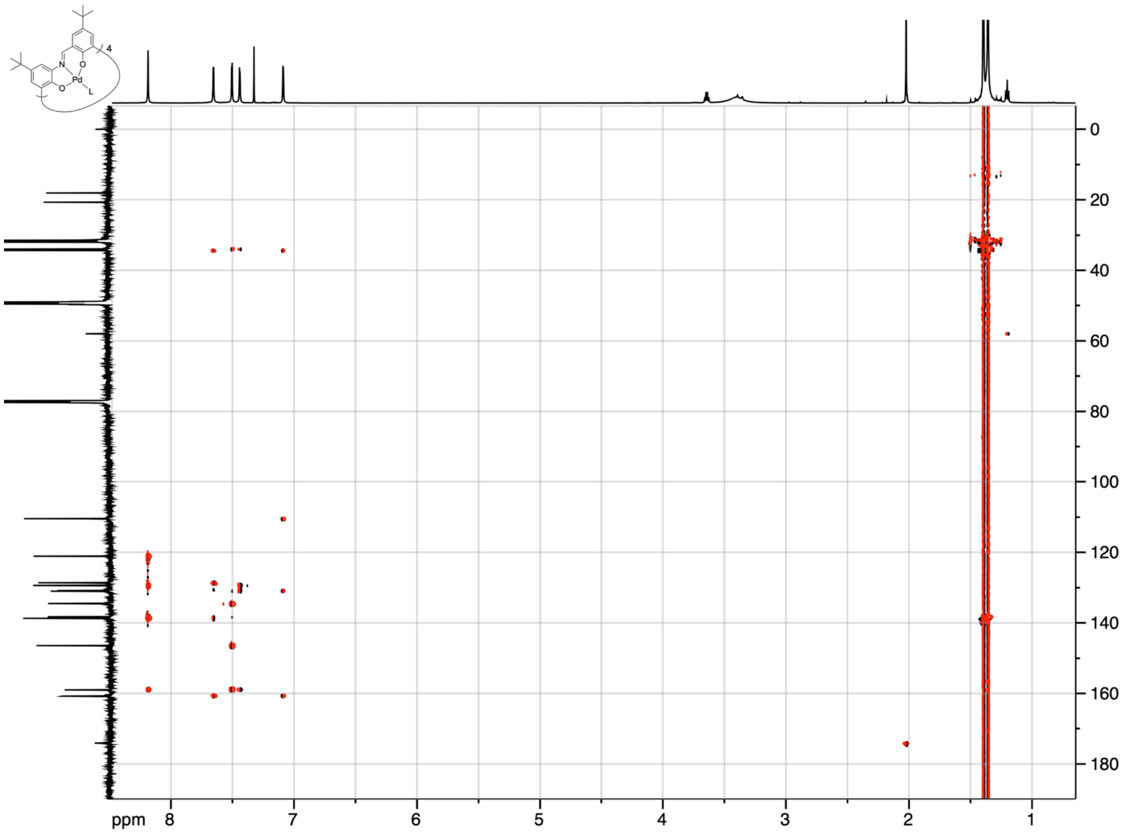

Figure S29. ^{1}H–^{13}C HMBC NMR spectrum of [**C4**Pd_4L_4] (600 MHz, $CDCl_3/CD_3OD$ = 10/1).

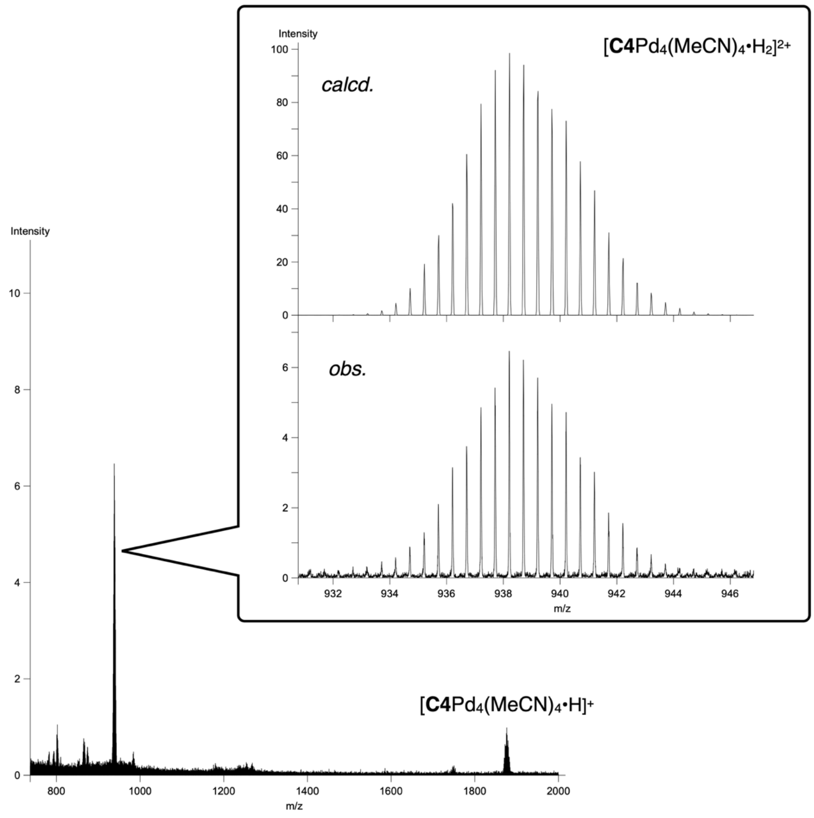

Figure S30. ESI-TOF mass spectrum of [**C4**Pd_4L_4] (positive, MeCN, 5 μM).

4-3. Comparison of ^{1}H NMR spectra in different solvents

In an NMR tube, [**C4**Pd_4L_4] was dissolved in $CDCl_3$, C_6D_6, toluene-d_8, THF-d_8 or $CDCl_3/CD_3OD$ = 10/1, then ^{1}H NMR spectra were measured. [**C4**Pd_4L_4] dissolved in each solvent and gave brown ~ orange solutions.

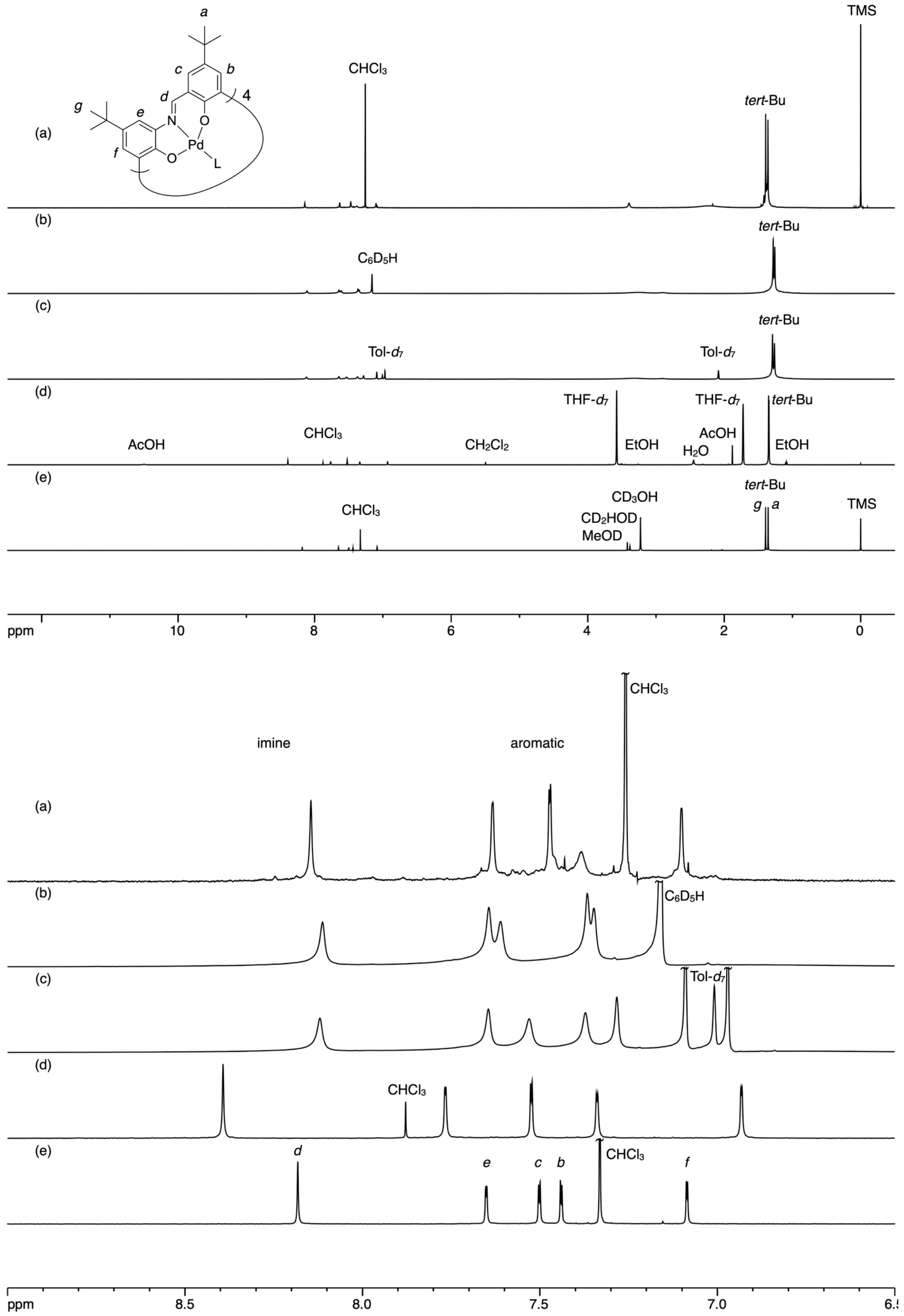

Figure S31. ^{1}H NMR spectra of [**C4**Pd_4L_4] (600 MHz). (a) $CDCl_3$, (b) C_6D_6, (c) toluene-d_8, (d) THF-d_8, (e) $CDCl_3/CD_3OD$ = 10/1. (a,e) L_4 = $(MeOH)_{2.4}(H_2O)_{1.4}(EtOH)_{0.1}(AcOH)_{0.1}$. (b,c) L_4 = $(MeOH)_{3.0}(H_2O)_{0.9}(AcOH)_{0.1}$. (d) L_4 = $(EtOH)_2(AcOH)_2$.

4-4. Comparison of ^{1}H NMR spectra for different coordination solvents L

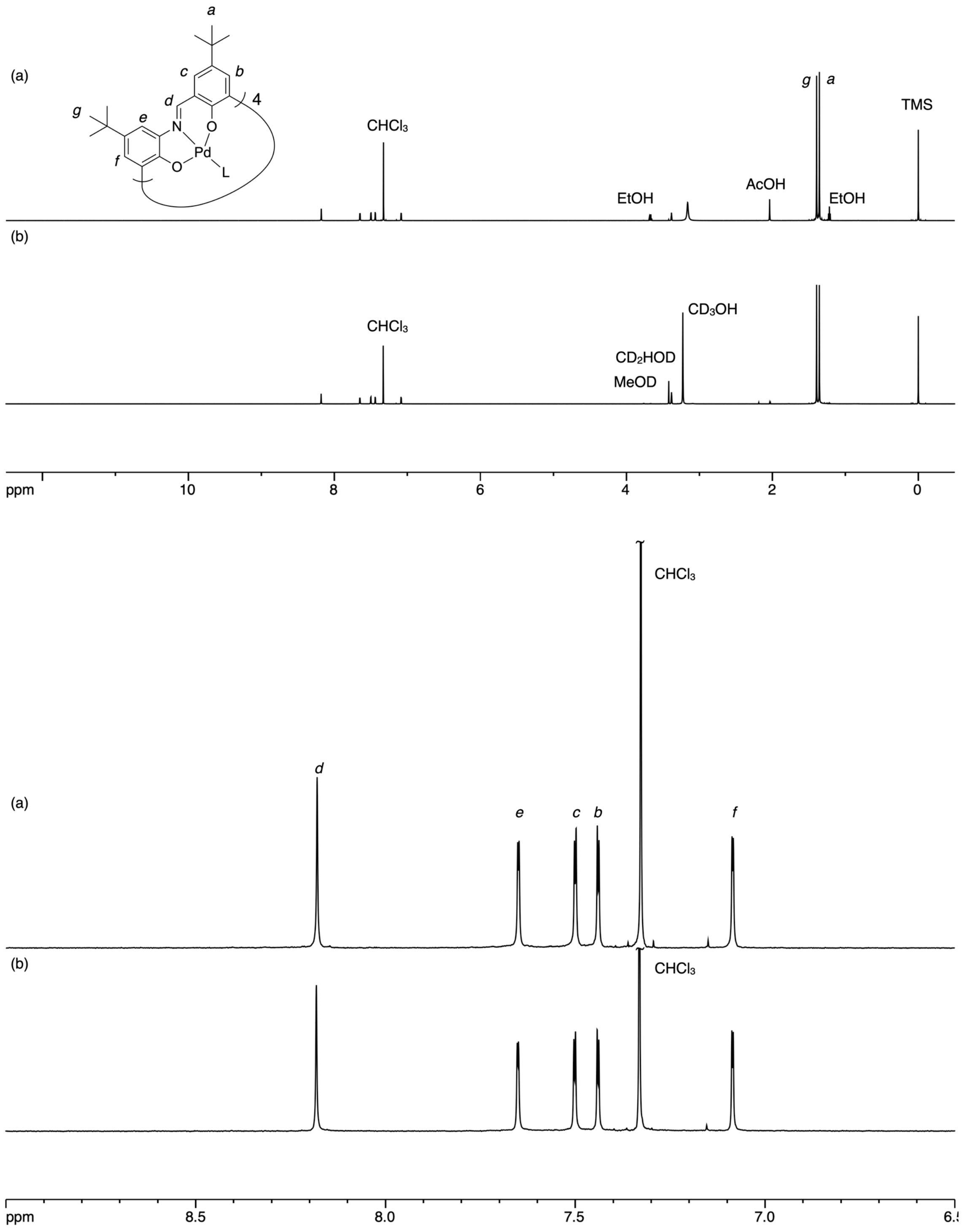

Figure S32. ^{1}H NMR spectra of [**C4**Pd_4L_4] (600 MHz, $CDCl_3/CD_3OD$ = 10/1).
(a) $L_4 = (EtOH)_2(AcOH)_2$. (b) $L_4 = (MeOH)_{2.4}(H_2O)_{1.4}(EtOH)_{0.1}(AcOH)_{0.1}$.

The same signals were observed for the complexes [**C4**Pd_4L_4] with different L in $CDCl_3/CD_3OD$ = 10/1 solvent.

4-5. X-ray diffraction analysis of [**C4**$Pd_4(MeOH)_4$]

A single crystal of [**C4**$Pd_4(MeOH)_4$] suitable for the X-ray diffraction analysis was obtained by slow evaporation from CH_2Cl_2/MeOH = 1/4 solution of [**C4**Pd_4L_4] (13 mM).

SADI, RIGU and ISOR restraints were applied for disordered *t*Bu groups. DFIX and RIGU restraints were applied for MeOH solvent molecules. Some hydrogens of MeOH were not generated by AFIX 137 and AFIX 147 because the structure was not converged. Hydrogens of MeOH coordinating to Pd were generated by AFIX 43.

Crystallographic data for $C_{98}H_{148}N_4O_{22}Pd_4$ ([**C4**$Pd_4(MeOH)_4$]•10MeOH), Fw = 2159.80, orange block, 0.24 × 0.24 × 0.14 mm^3, triclinic, space group P-1 (No. 2), a = 15.492(3) Å, b = 16.522(4) Å, c = 22.426(5) Å, α = 95.079(2)°, β = 91.591(2)°, γ = 106.167(2)°, V = 5483(2) $Å^3$, Z = 2, T = 100 K, λ = 0.71073 Å, θ_{max} = 26.501°, R_1 = 0.0902 ($I > 2\sigma$), wR_2 = 0.2948 (total), GOF = 1.117. CCDC 2412785.

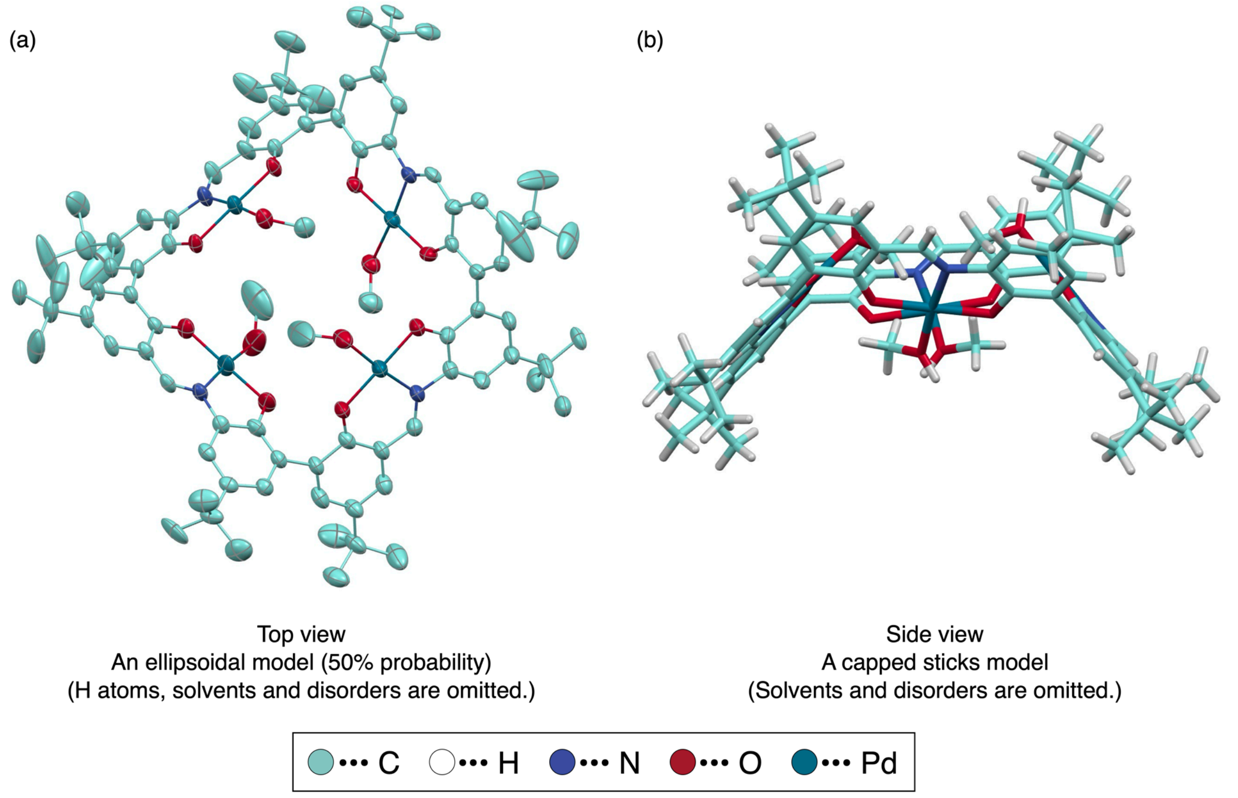

Figure S33. The molecular structure of [**C4**$Pd_4(MeOH)_4$] determined by X-ray diffraction analysis. (a) An ellipsoidal model (50% probability). (b) A capped sticks model.

5. Coordination binding experiments of amylene

5-1. Coordination binding of amylene as a stabilizer in chloroform

[**C4**$Pd_4(EtOH)_2(AcOH)_2$] (32.7 mg, 17.2 μmol) was dissolved in chloroform stabilized by amylene (about 150 ppm) (20 mL) and washed with water (20 mL) twice. The organic layer was concentrated in vacuo. The residue was measured by ^{1}H NMR as $CDCl_3$ and $CDCl_3/CD_3OD$ = 10/1 solution ($CDCl_3$ did not contain amylene.). Also, MeOH was added to the residue, and the mixture was concentrated in vacuo, then measured ^{1}H NMR as $CDCl_3$ and $CDCl_3/CD_3OD$ = 10/1 solution again.

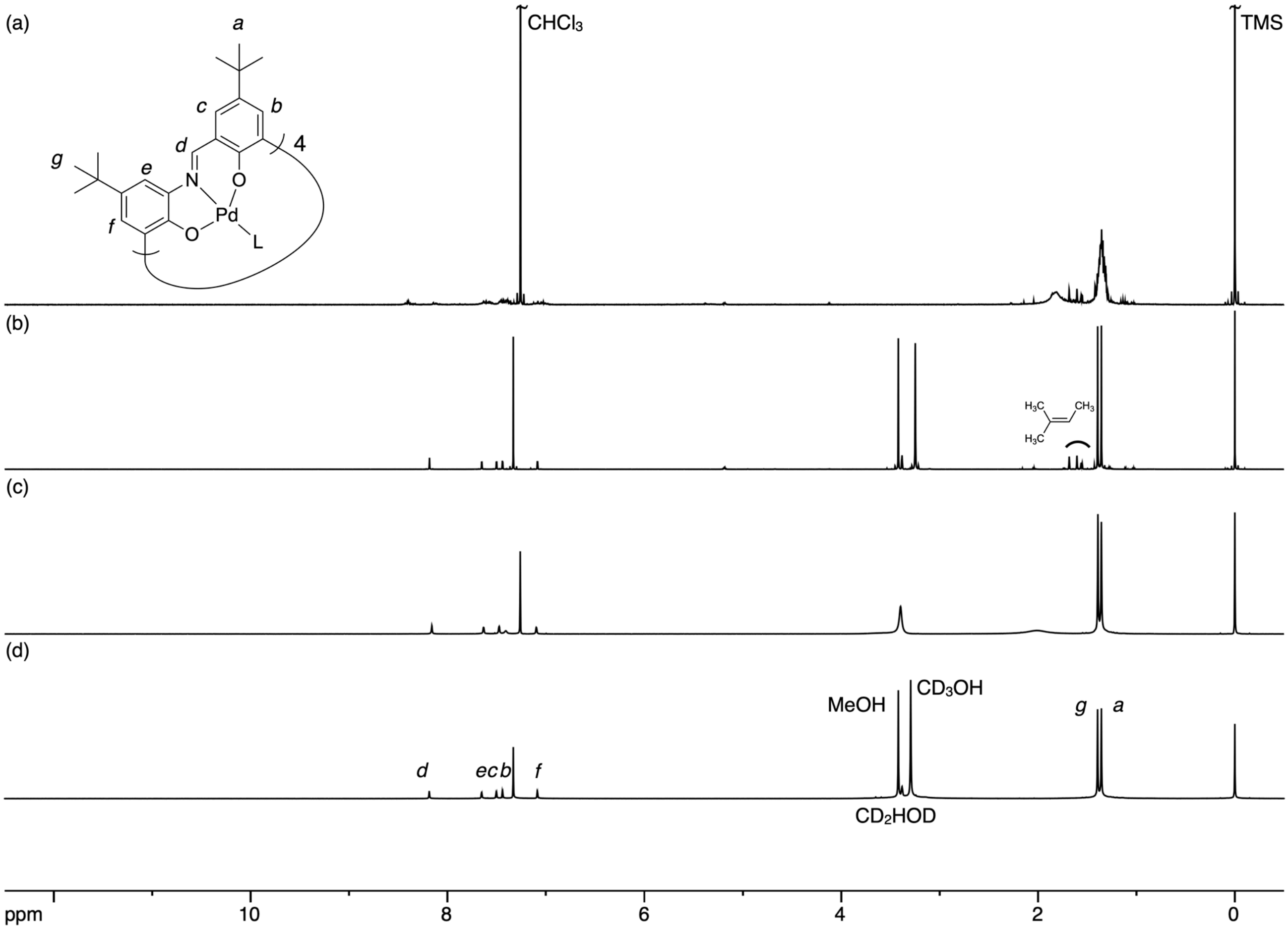

Figure S34. ^{1}H NMR spectra of each residue. (a,b) The residue from the chloroform stabilized by amylene solution (600 MHz). (c,d) The residue from the MeOH mixture (400 MHz). (a,c) $CDCl_3$. (b,d) $CDCl_3/CD_3OD$ = 10/1.

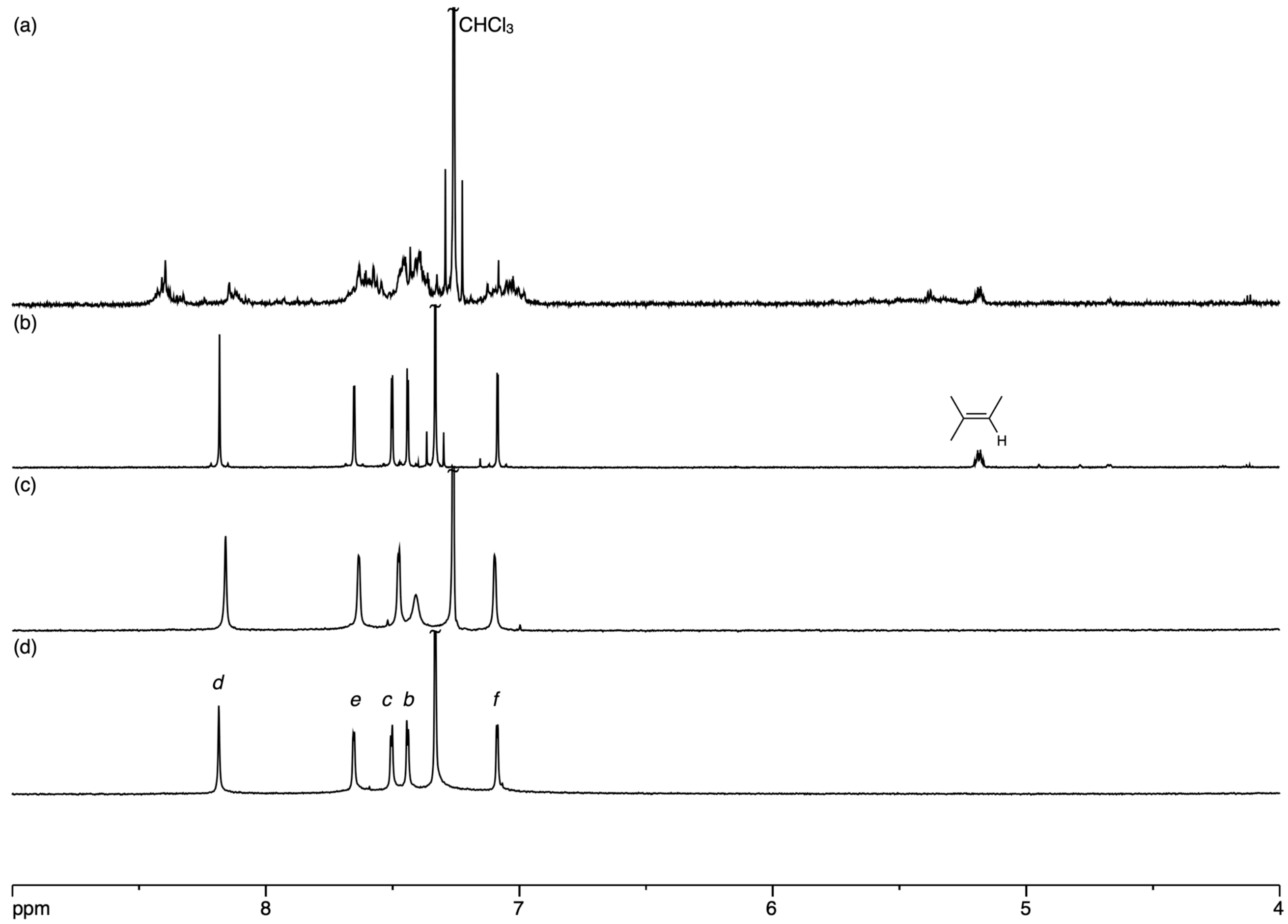

Figure S35. Enlarged ^{1}H NMR spectra of Figure S34.

5-2. Titration of amylene against [**C4**Pd$_4$L$_4$]

[**C4**Pd$_4$(MeOH)$_{2.4}$(H$_2$O)$_{1.4}$(EtOH)$_{0.1}$(AcOH)$_{0.1}$]•8H$_2$O (1.36 mg, 0.691 μmol, 1.0 equiv.) was added to an NMR tube and dissolved in CDCl$_3$ (450 μL, 1.5 mM) as the host solution. Amylene (1.62 mg, 23.1 μmol) was added to a microtube and dissolved in CDCl$_3$ (465 μL) as the guest solution. Each 15.0 μL (0.745 μmol, 1.1 equiv.) aliquot of the guest solution was titrated into the host solution until 4.3 equiv. Also, amylene (5.46 mg, 77.8 μmol) was added to a microtube and dissolved in CDCl$_3$ (100 μL) as the guest solution. 25 μL (19.46 μmol, 28 equiv.) aliquot of the guest solution was added to the solution (total 32 equiv. of amylene was added.). The ^{1}H NMR measurements (600 MHz, r.t.) were carried out during the titration.

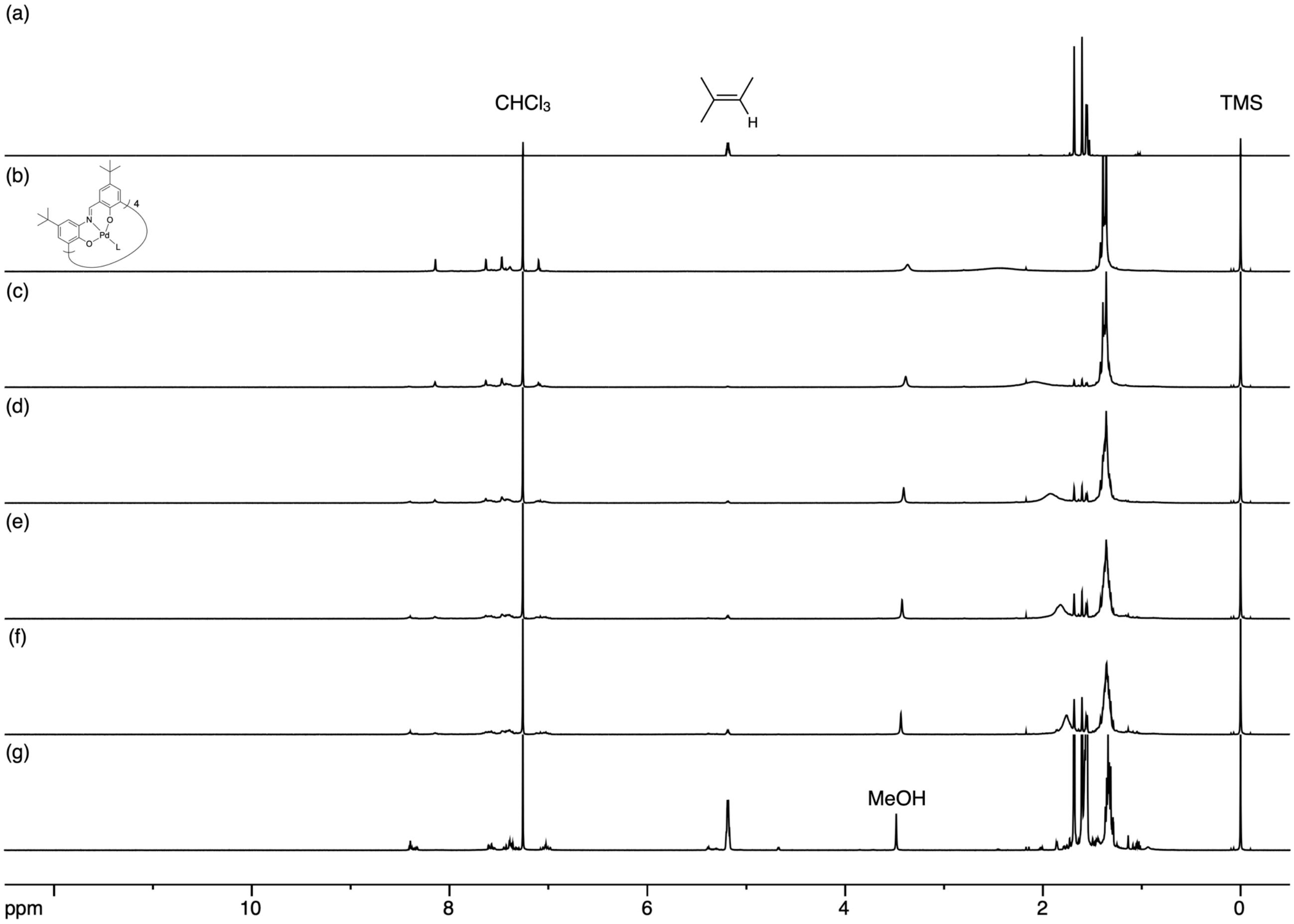

Figure S36. Titration of amylene to [**C4**Pd$_4$L$_4$] in CDCl$_3$ investigated by ^{1}H NMR measurements. (a–g) ^{1}H NMR spectra (600 MHz, CDCl$_3$). (a) Amylene. (b) [**C4**Pd$_4$L$_4$]. (c) [**C4**Pd$_4$L$_4$] + amylene 1.1 equiv. (d) [**C4**Pd$_4$L$_4$] + amylene 2.2 equiv. (e) [**C4**Pd$_4$L$_4$] + amylene 3.2 equiv. (f) [**C4**Pd$_4$L$_4$] + amylene 4.3 equiv. (g) [**C4**Pd$_4$L$_4$] + amylene 32 equiv. L$_4$ = (MeOH)$_{2.4}$(H$_2$O)$_{1.4}$(EtOH)$_{0.1}$(AcOH)$_{0.1}$.

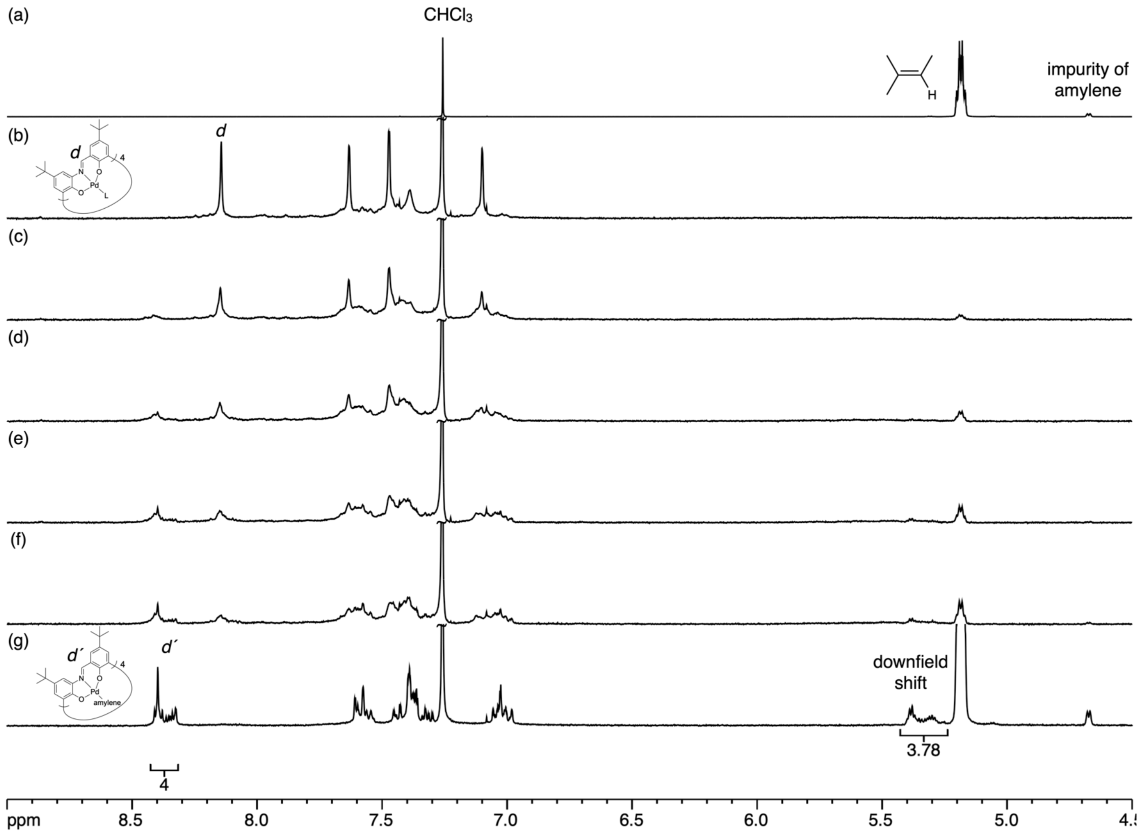

Figure S37. Enlarged ^{1}H NMR spectra of Figure S36.

The integral value of the olefin proton signals of amylene and that of the imine proton signals of Pd-tetrasap suggested that four amylene molecules coordinated to Pd-tetrasap. [**C4**Pd_4(amylene)$_4$] has many possible isomers due to orientation of the coordinating amylene (Figure S38), which resulted in multiple ^{1}H NMR signals.

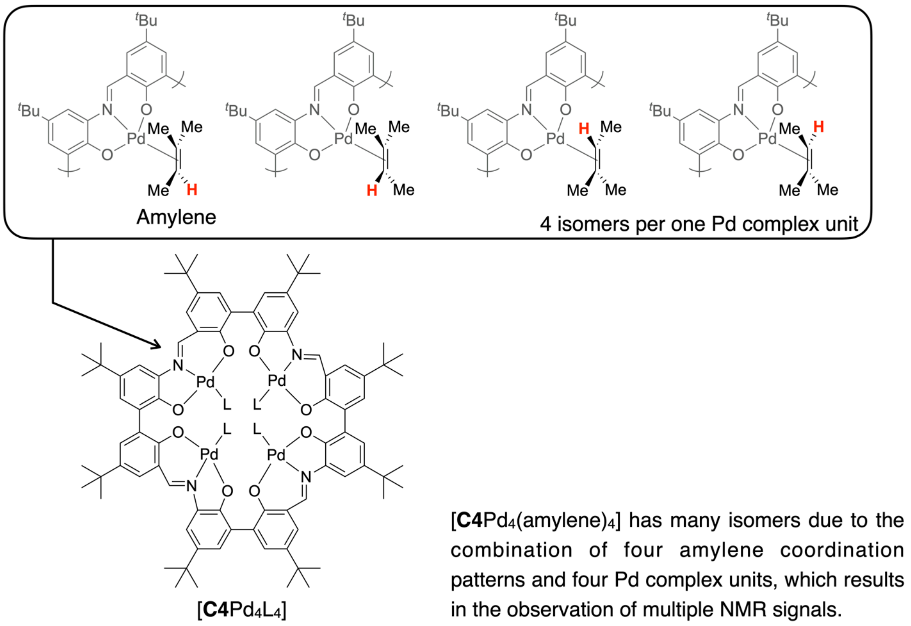

Figure S38. Possible isomers of [**C4**Pd_4(amylene)$_4$].

6. Coordination binding experiments of squalene

6-1. Titration of squalene against [**C4**Pd_4L_4]

A representative procedure:

[**C4**$Pd_4(MeOH)_{2.0}(H_2O)_{2.0}$]•$13H_2O$ (2.14 mg, 1.05 μmol, 1.0 equiv.) was added to an NMR tube and dissolved in $CDCl_3$ (450 μL, 2.3 mM) as the host solution. Squalene (1.54 mg, 3.75 μmol) was added to a microtube and dissolved in $CDCl_3$ (190 μL) as the guest solution. Each 15.0 μL (0.296 μmol, 0.28 equiv.) aliquot of the guest solution was titrated to the host solution. The ^{1}H NMR measurements (600 MHz, r.t.) were carried out during the titration.

Titration experiments in C_6D_6 and THF-d_8 were conducted in similar manner.

Titration in $CDCl_3$

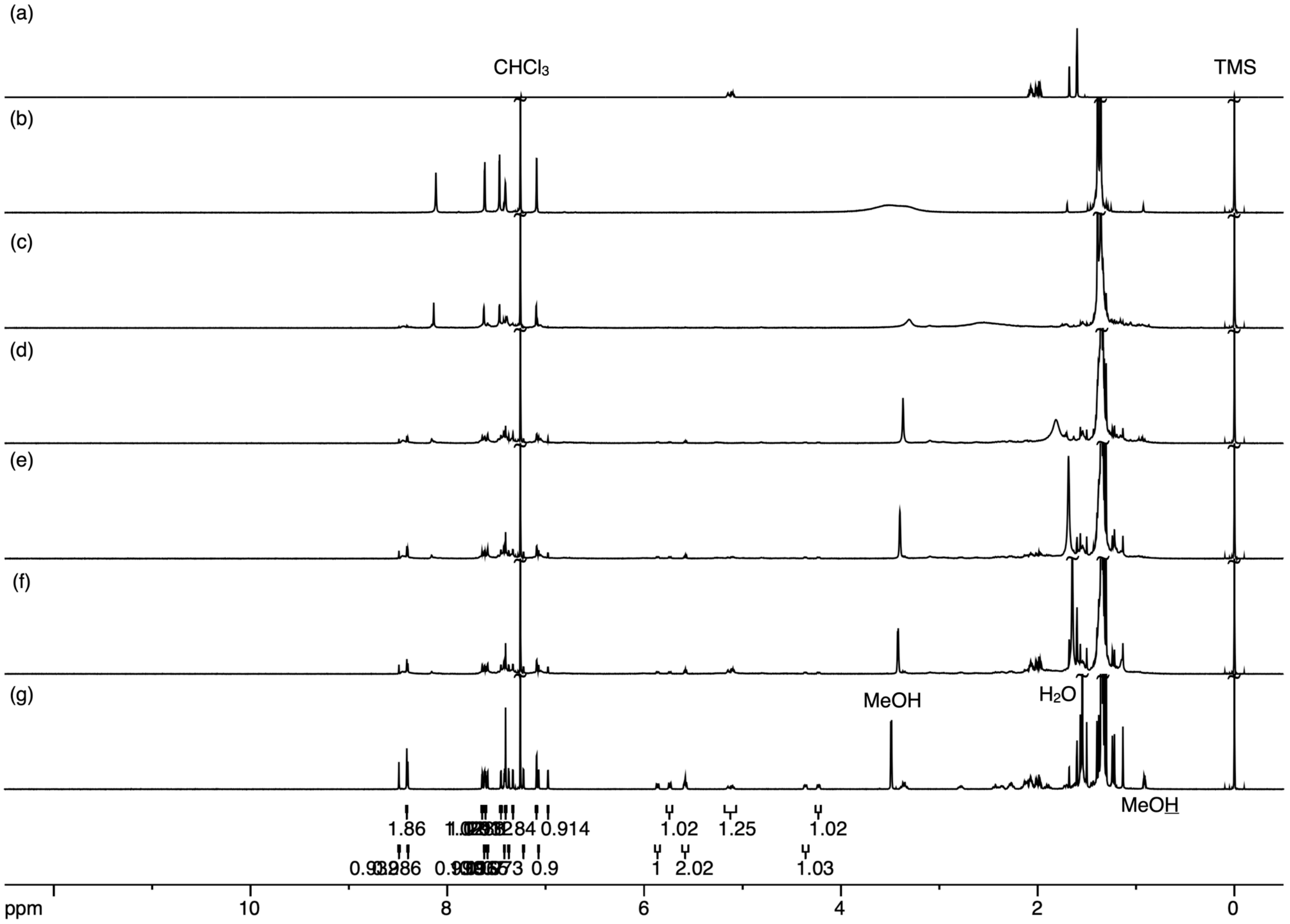

Figure S39. Titration of squalene to [**C4**Pd_4L_4] in $CDCl_3$ investigated by ^{1}H NMR measurements. (a–f) ^{1}H NMR spectra (600 MHz, $CDCl_3$). (a) Squalene. (b) [**C4**Pd_4L_4]. (c) [**C4**Pd_4L_4] + squalene 0.28 equiv. (d) [**C4**Pd_4L_4] + squalene 0.56 equiv. (e) [**C4**Pd_4L_4] + squalene 0.85 equiv. (f) [**C4**Pd_4L_4] + squalene 1.1 equiv. (g) left to stand at r.t. for 1 d. $L_4 = (MeOH)_{2.0}(H_2O)_{2.0}$.

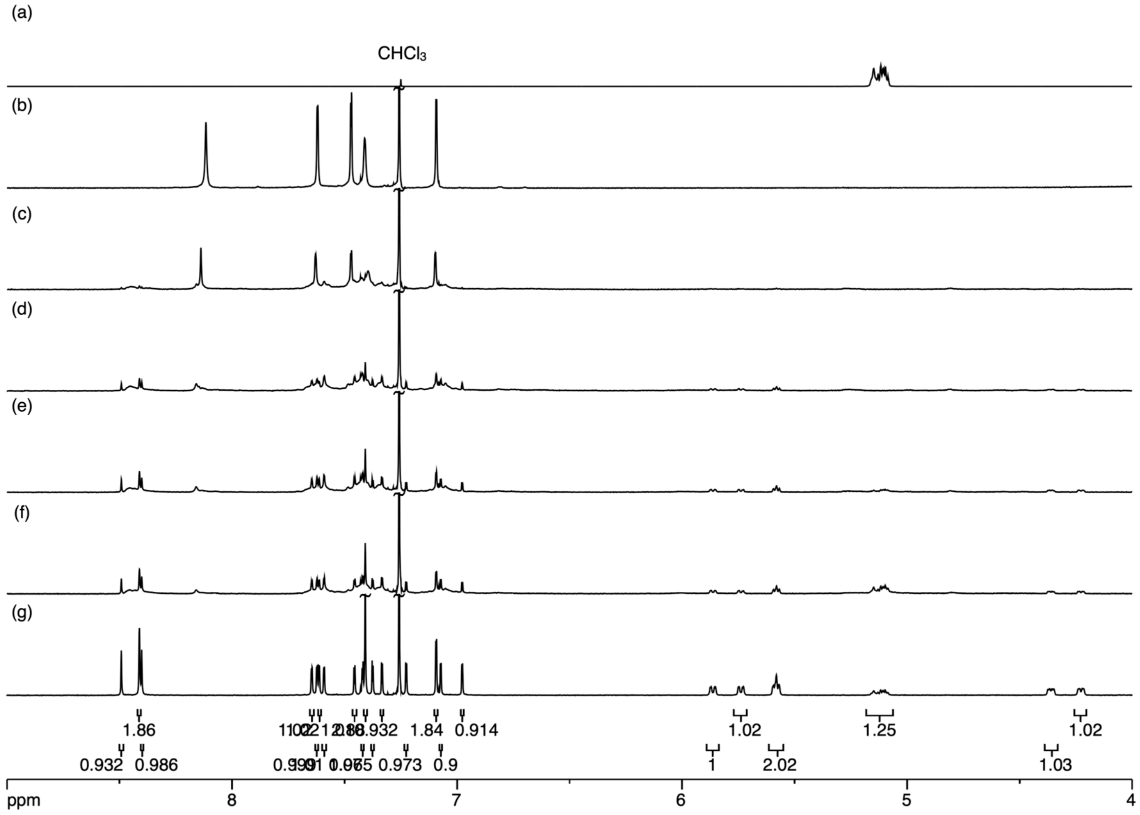

Figure S40. Enlarged ^{1}H NMR spectra of Figure S39.

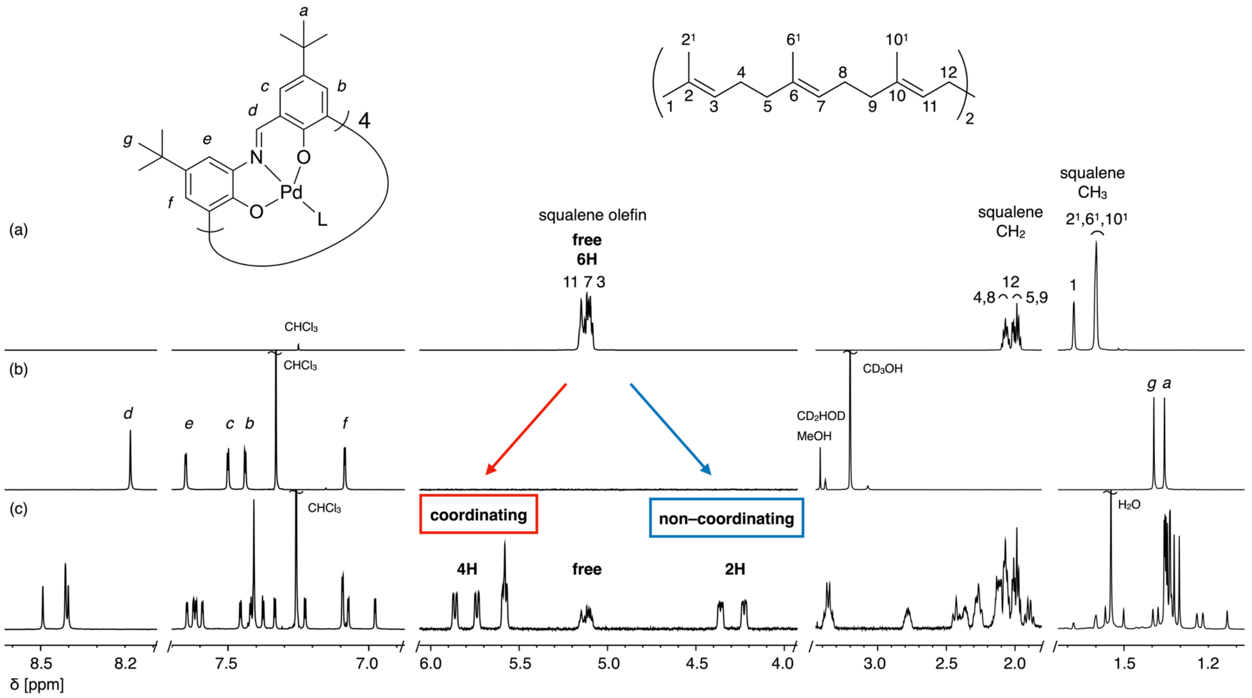

Figure S41. Comparison of squalene, [**C4**Pd_4L_4], and [**C4**Pd_4(squalene)]. (a–c) ^{1}H NMR spectra (600 MHz). (a) Squalene ($CDCl_3$). (b) [**C4**Pd_4L_4] ($CDCl_3/CD_3OD$ = 10/1). (c) [**C4**Pd_4L_4] + squalene 1.1 equiv. then left to stand at r.t. for 1 d. $L_4 = (MeOH)_{2.0}(H_2O)_{2.0}$.

Titration in C_6D_6

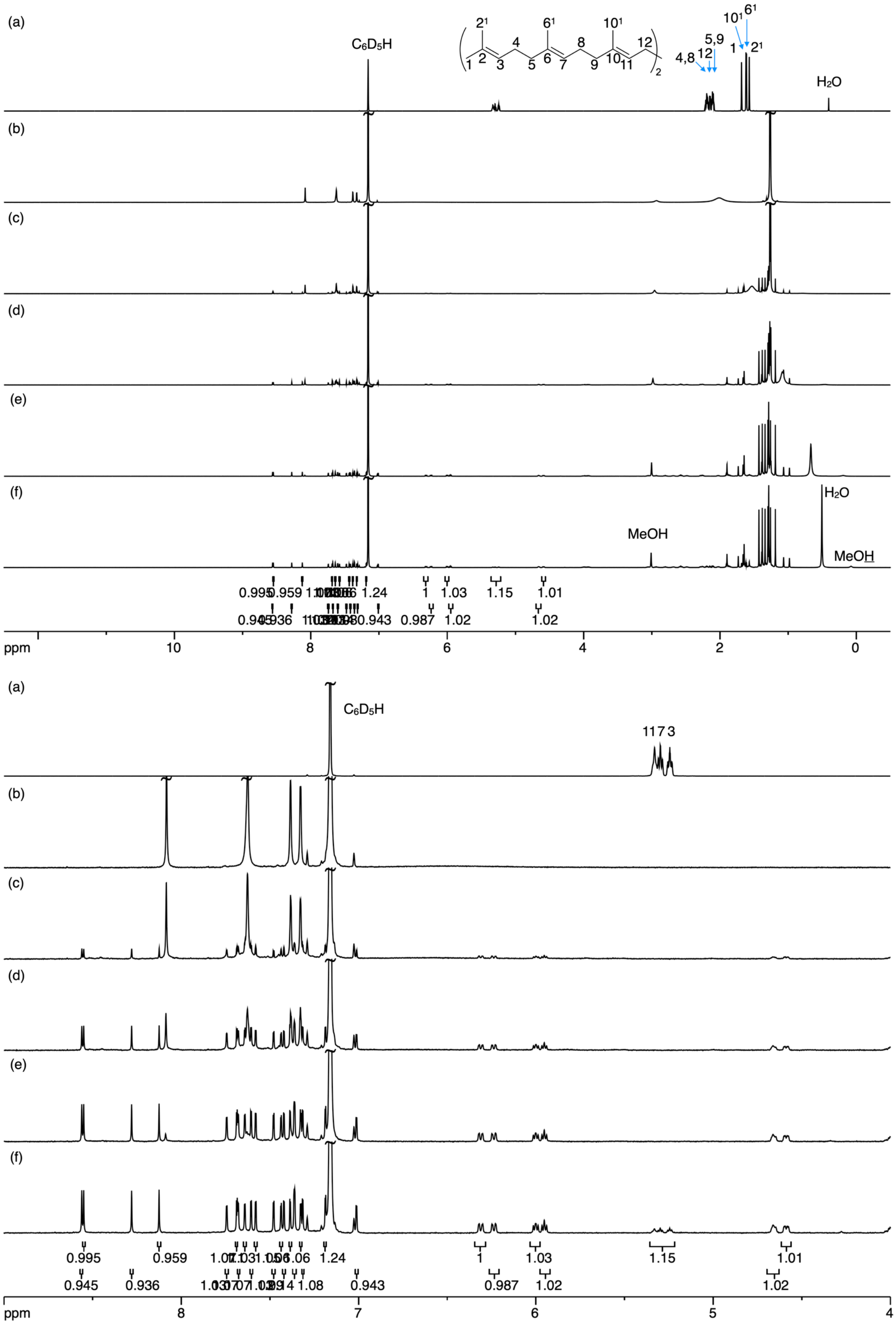

Figure S42. Titration of squalene to [**C4**Pd_4L_4] in C_6D_6 investigated by ^{1}H NMR measurements. (a–f) ^{1}H NMR spectra (600 MHz, C_6D_6). (a) Squalene. (b) [**C4**Pd_4L_4]. (c) [**C4**Pd_4L_4] + squalene 0.28 equiv. (d) [**C4**Pd_4L_4] + squalene 0.56 equiv. (e) [**C4**Pd_4L_4] + squalene 0.85 equiv. (f) [**C4**Pd_4L_4] + squalene 1.1 equiv. L_4 = $(MeOH)_{2.0}(H_2O)_{2.0}$.

Titration in THF-d_8

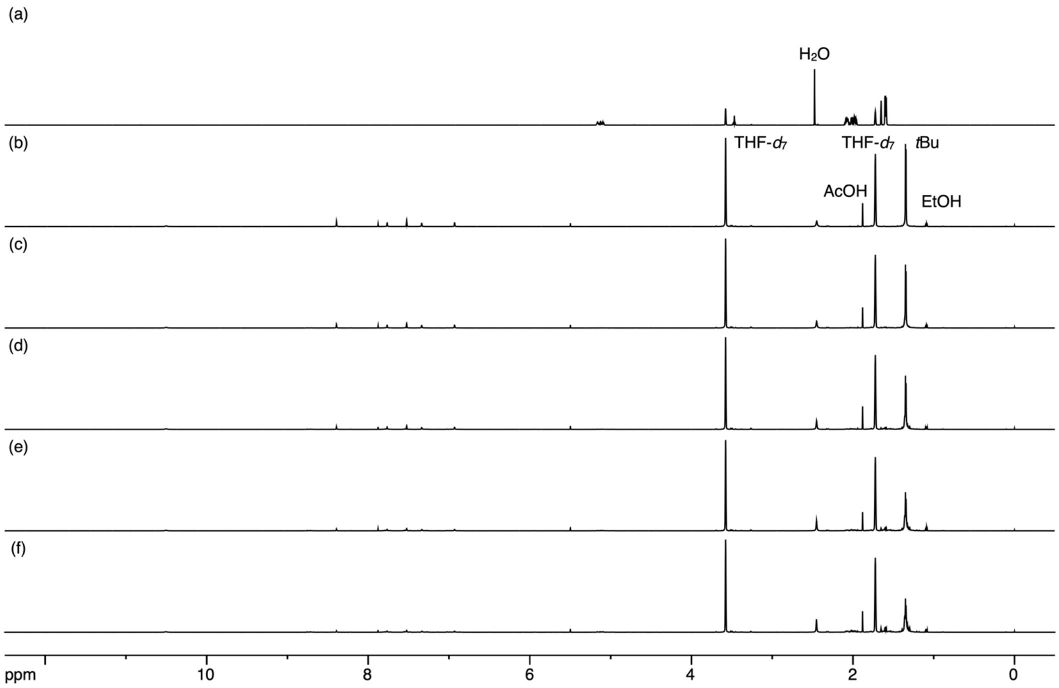

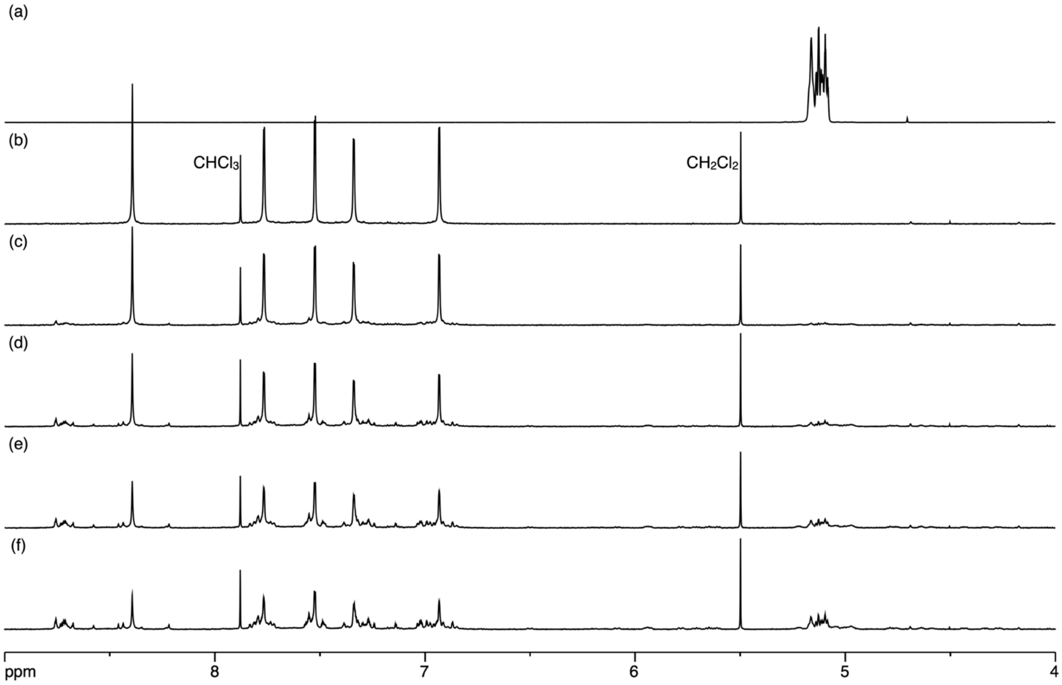

Figure S43. Titration of squalene to [**C4**Pd_4L_4] in THF-d_8 investigated by ^{1}H NMR measurements. (a–f) ^{1}H NMR spectra (600 MHz, THF-d_8). (a) Squalene. (b) [**C4**Pd_4L_4]. (c) [**C4**Pd_4L_4] + squalene 0.25 equiv. (d) [**C4**Pd_4L_4] + squalene 0.50 equiv. (e) [**C4**Pd_4L_4] + squalene 0.75 equiv. (f) [**C4**Pd_4L_4] + squalene 1.0 equiv. L_4 = $(EtOH)_2(AcOH)_2$.

Excess amount of squalene

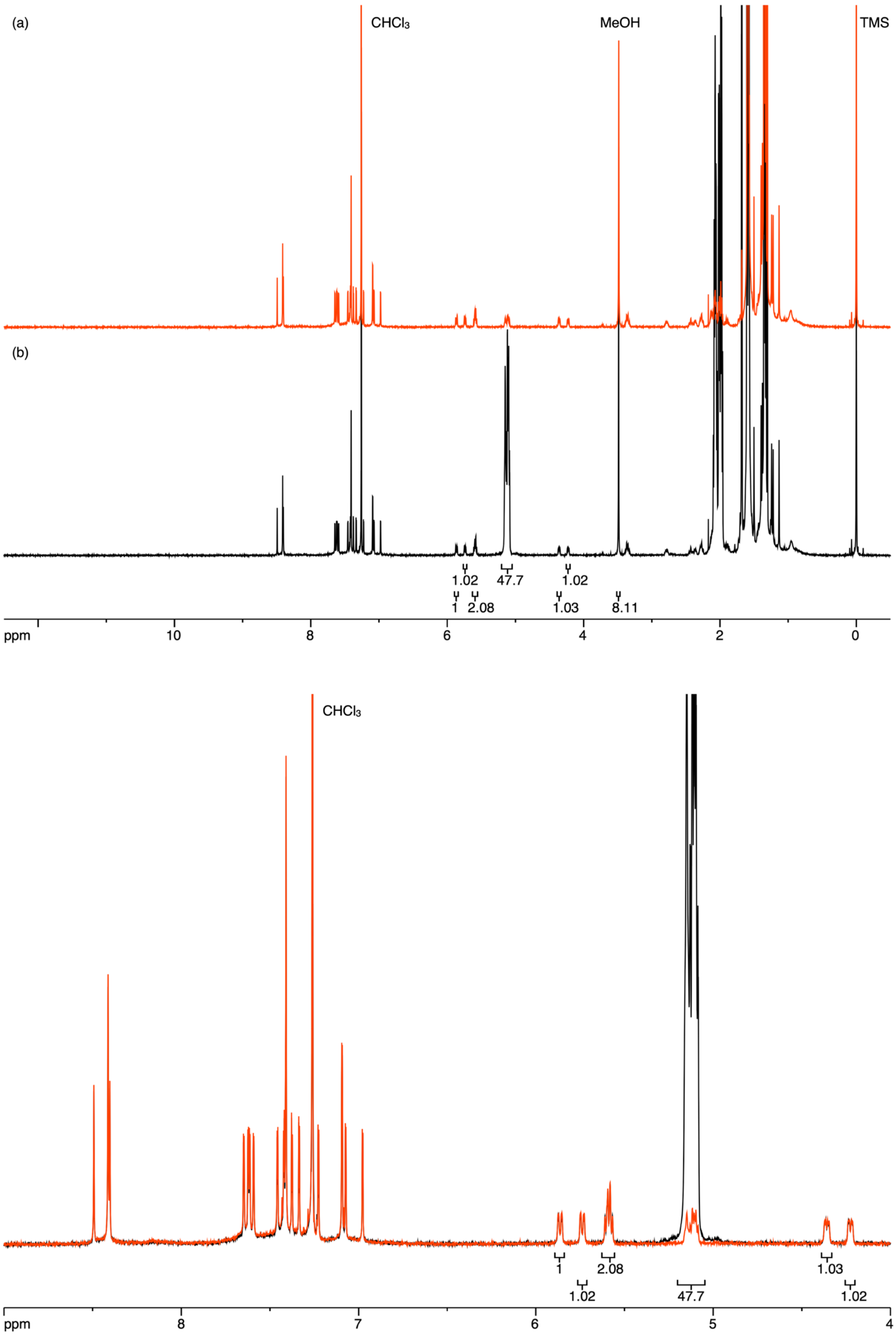

Figure S44. ^{1}H NMR spectra of using excess amount of squalene (600 MHz, $CDCl_3$). (a) [**C4**Pd_4L_4] + squalene 1.0 equiv. (red). (b) [**C4**Pd_4L_4] + squalene 6.0 equiv. (black). $L_4 = (MeOH)_{2.5}(H_2O)_{1.3}(EtOH)_{0.1}(AcOH)_{0.1}$.

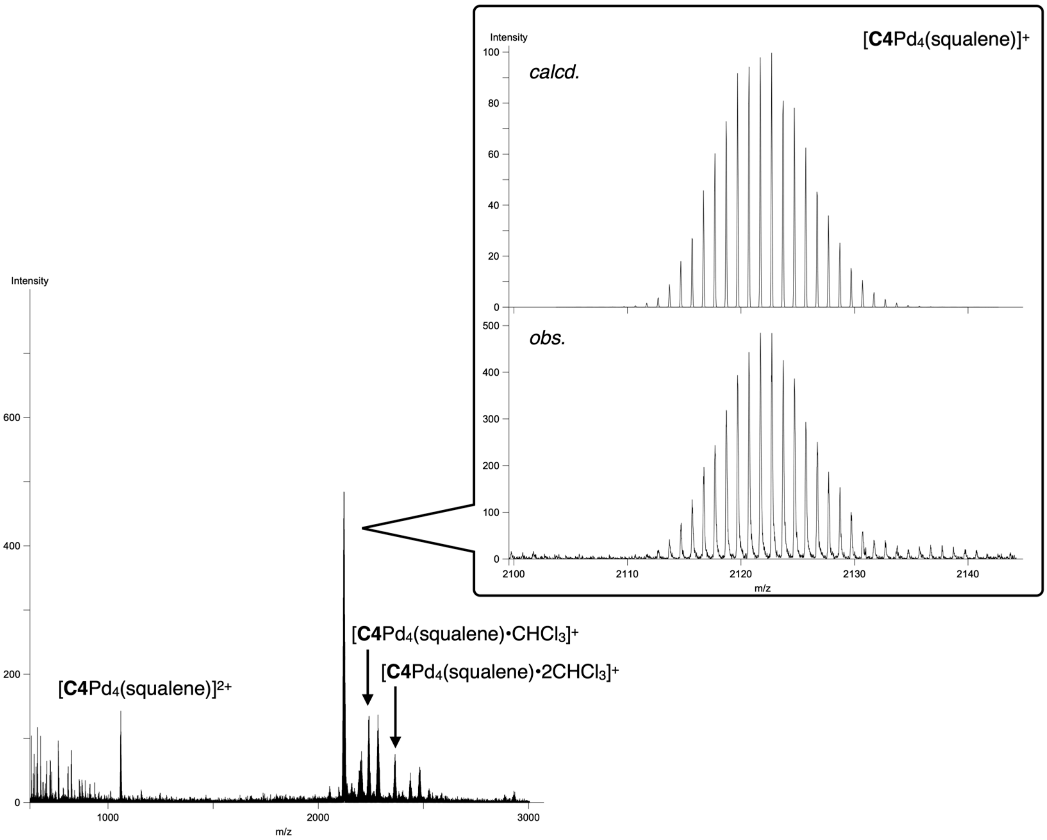

Figure S45. ESI-TOF mass spectrum of [**C4**Pd$_4$(squalene)] (positive, $CHCl_3$, 2.7 μM).

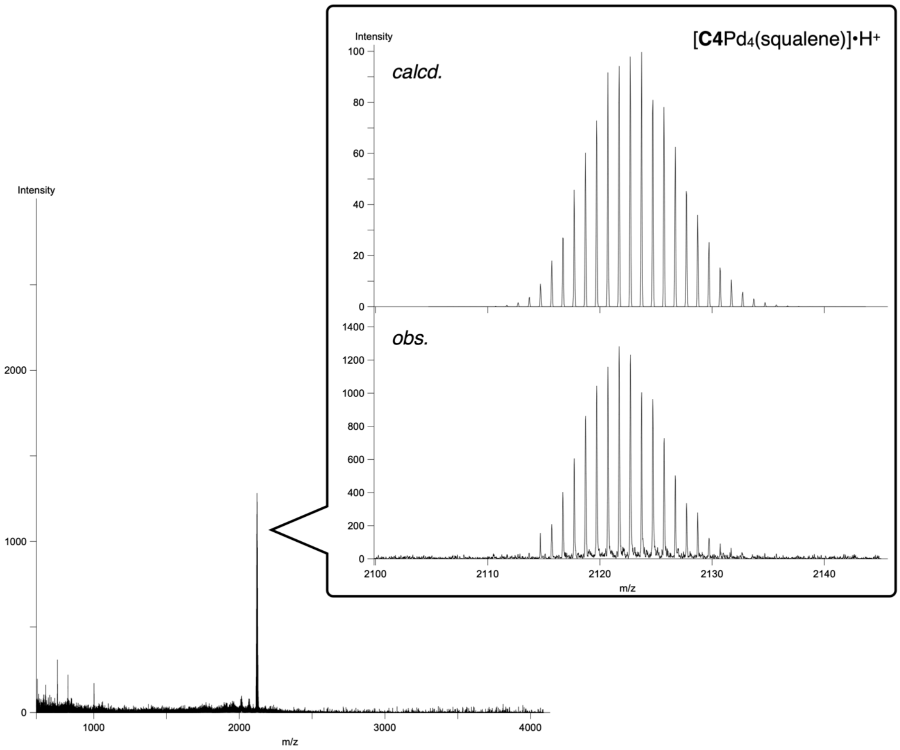

Figure S46. MALDI-TOF mass spectrum of [**C4**Pd$_4$(squalene)] (positive, matrix; DCTB (*trans*-2-[3-(4-*tert*-Butylphenyl)-2-methyl-2-propenylidene]malononitrile)).

The ^{1}H NMR spectra obtained during the titration experiments squalene against Pd-tetrasap [**C4**$Pd_4(MeOH)_{2.0}(H_2O)_{2.0}$] in $CDCl_3$ and C_6D_6 (2 mM) indicated that the ratio of the free host and the free guest were less the 10% compared to the 1:1 host-guest complex at the titration point of around 1 equiv. of guest (Figure S39g, Figure S42e,f). This confirmed that the association constant K [M^{-1}] is greater than 10^4 (i.e. log $K > 4$) in both solvents. Due to the strong binding, it was difficult to determine a reliable value from the ^{1}H NMR titration experiments.

6-2. NMR assignment of [**C4**Pd_4(squalene)]

In an NMR tube, C_6D_6 (475 µL) was added to [**C4**$Pd_4(MeOH)_{2.4}(H_2O)_{1.4}(EtOH)_{0.1}(AcOH)_{0.1}$]•$8H_2O$ (19.88 mg, 10.10 µmol, 1.0 equiv.), then squalene C_6D_6 solution (60 µL, 10.92 µmol, 1.1 equiv.) was added. The sample was left at r.t. for 1 d, then the ^{1}H, ^{13}C and 2D NMR measurements were carried out at r.t. for 1.5 d.

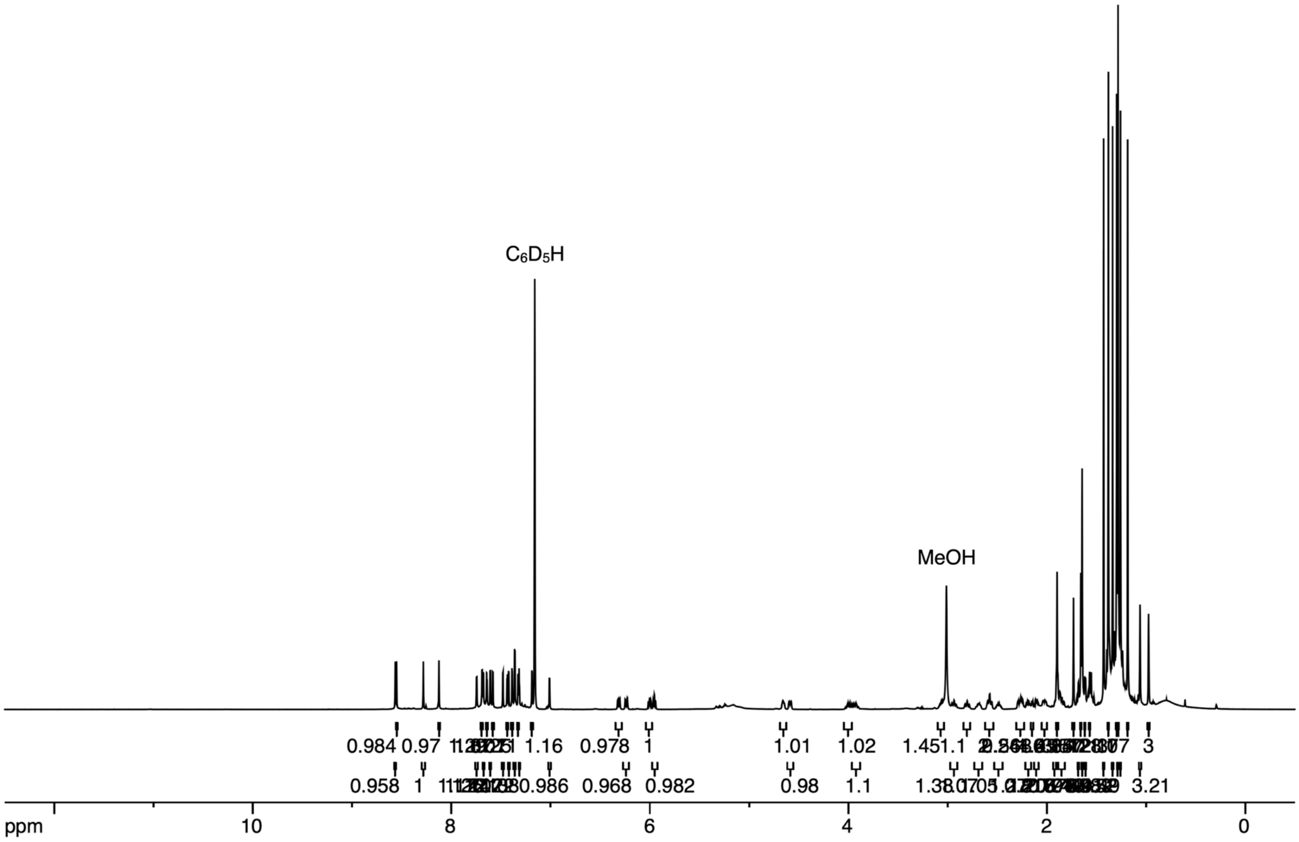

Figure S47. ^{1}H NMR spectrum of [**C4**Pd_4(squalene)] (600 MHz, C_6D_6).

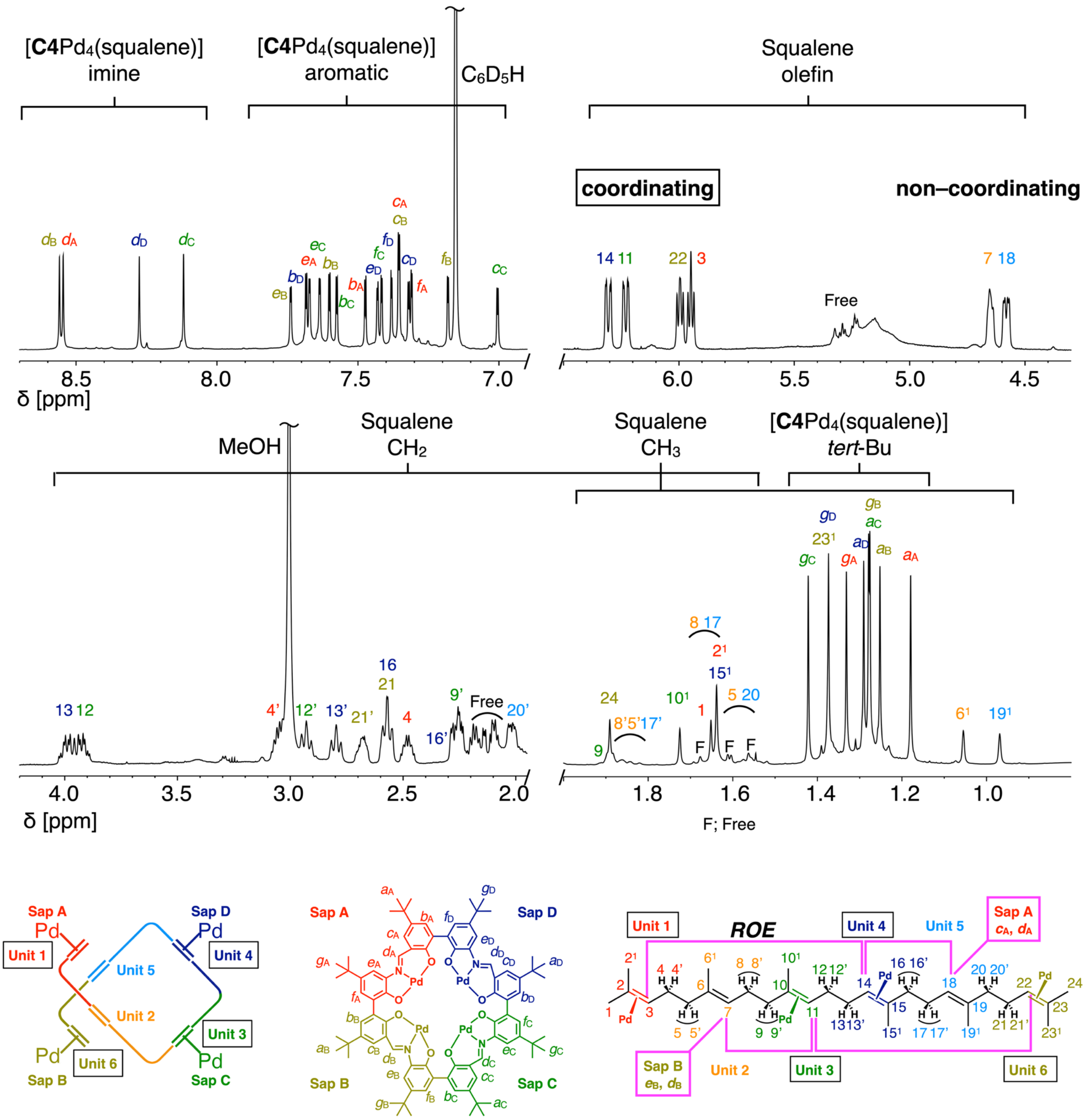

Figure S48. ^{1}H NMR assignment of [**C4**Pd_4(squalene)] (600 MHz, C_6D_6).

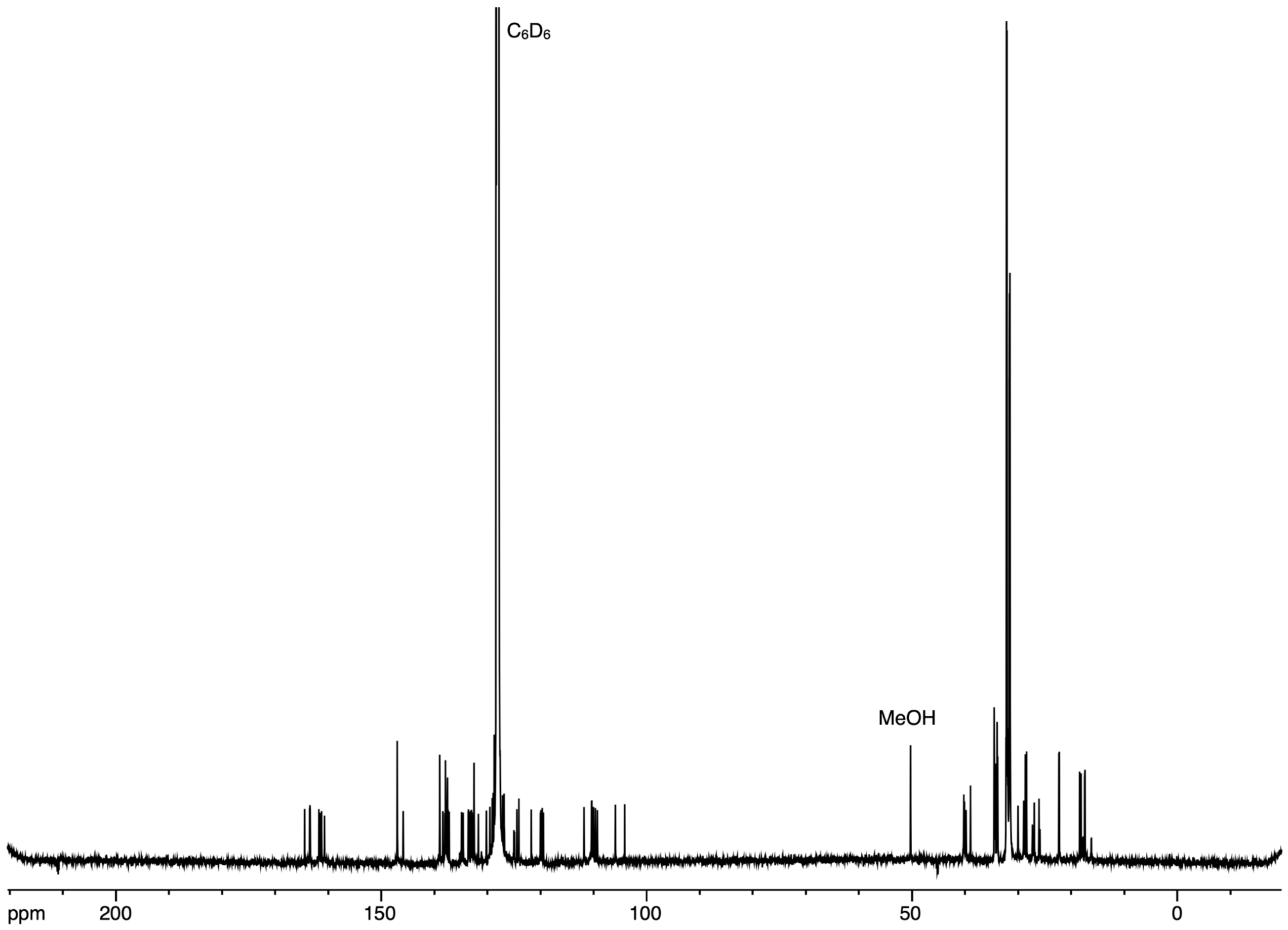

Figure S49. ^{13}C NMR spectrum of [**C4**Pd_4(squalene)] (151 MHz, C_6D_6).

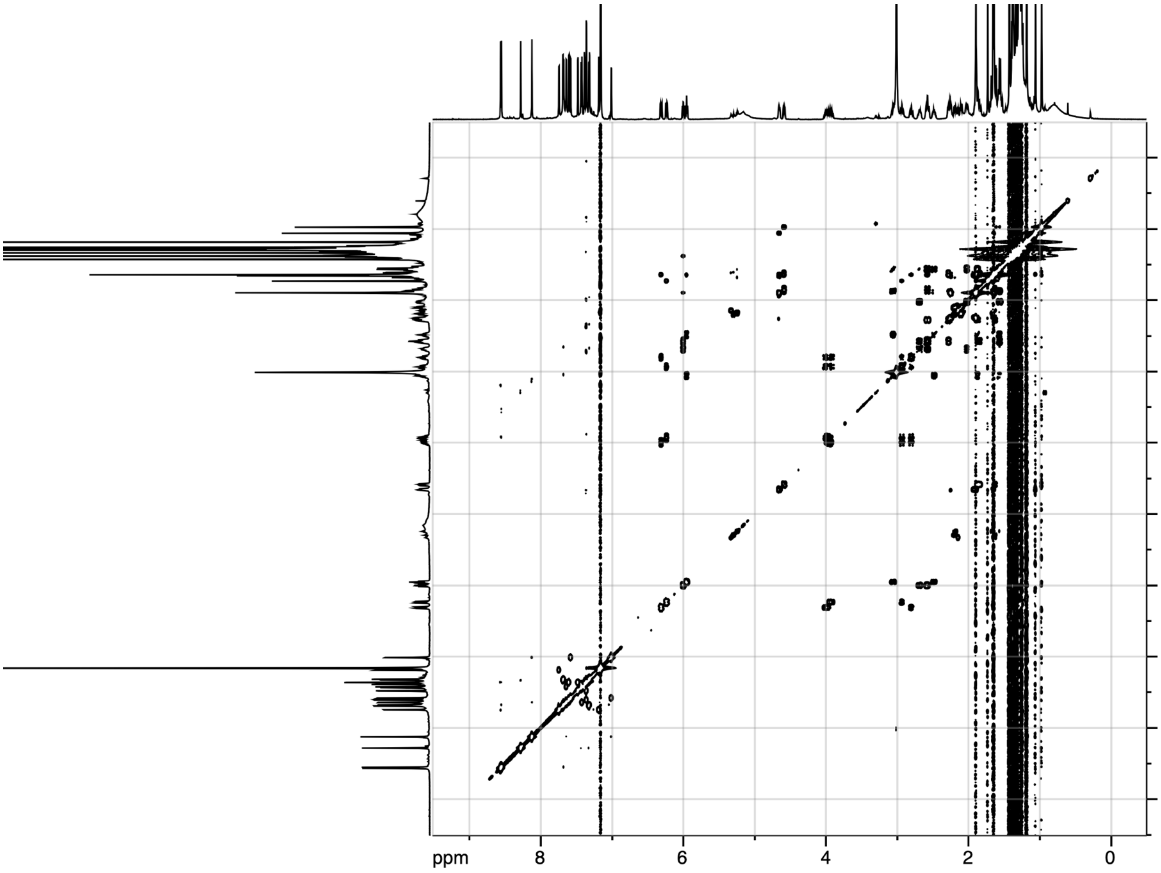

Figure S50. ^{1}H–^{1}H COSY NMR spectrum of [**C4**Pd_4(squalene)] (600 MHz, C_6D_6).

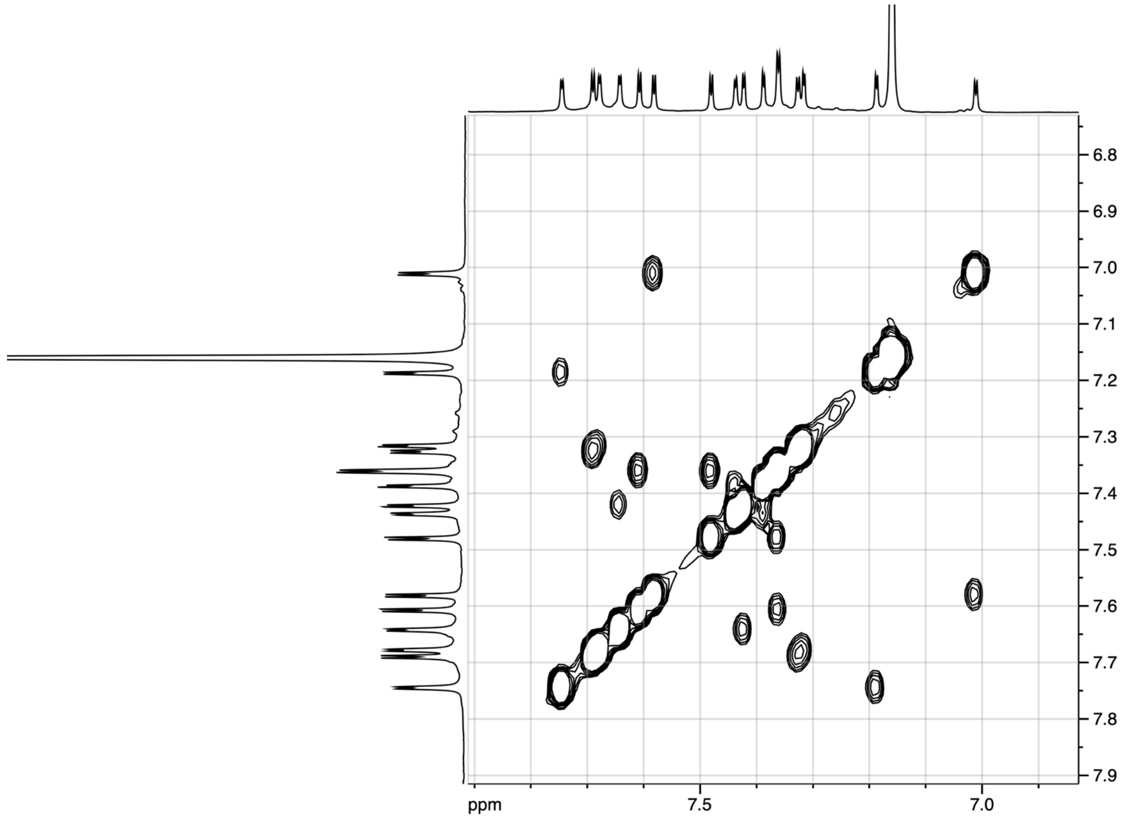

Figure S51. ^{1}H–^{1}H COSY NMR spectrum of [**C4**Pd_4(squalene)] (600 MHz, C_6D_6) (aromatic region).

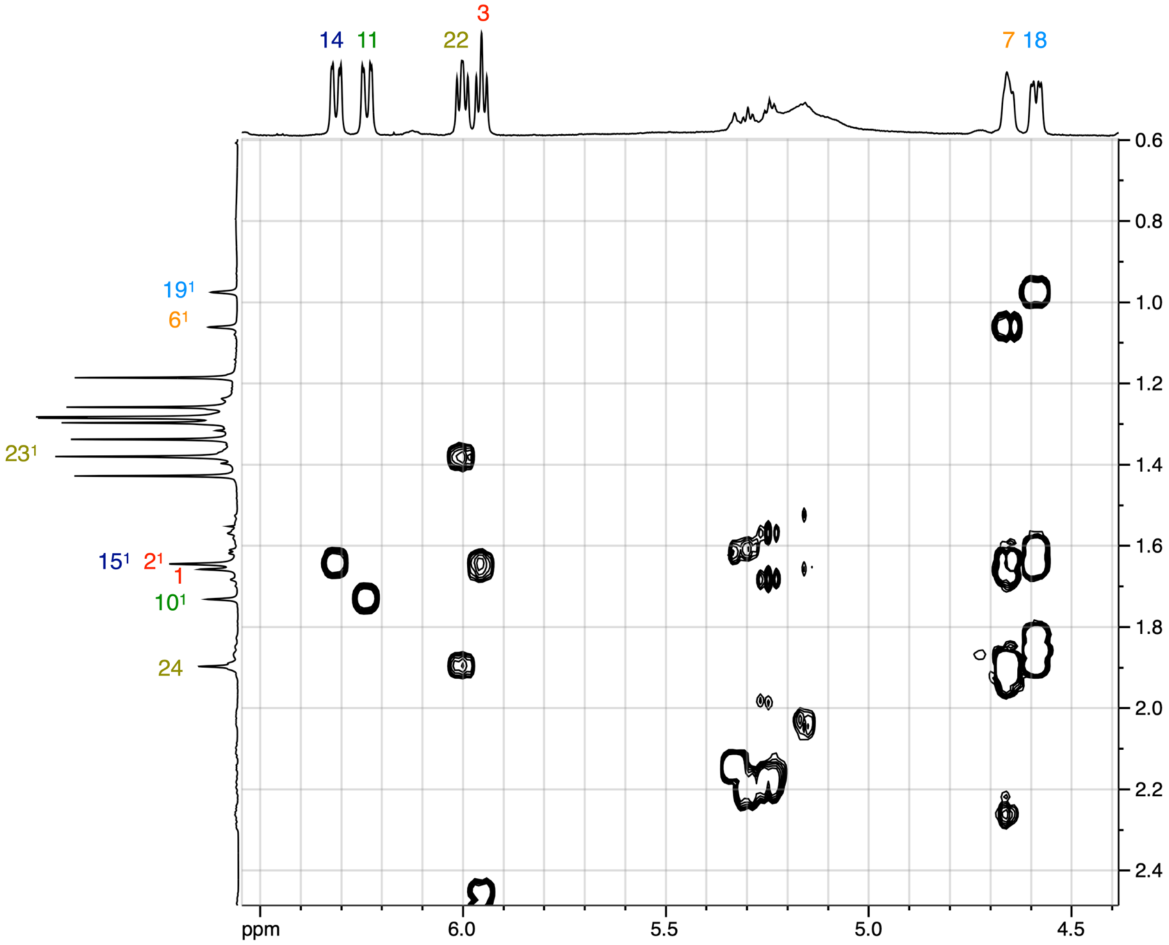

Figure S52. ^{1}H–^{1}H COSY NMR spectrum of [**C4**Pd_4(squalene)] (600 MHz, C_6D_6) (olefin–methyl cross peaks).

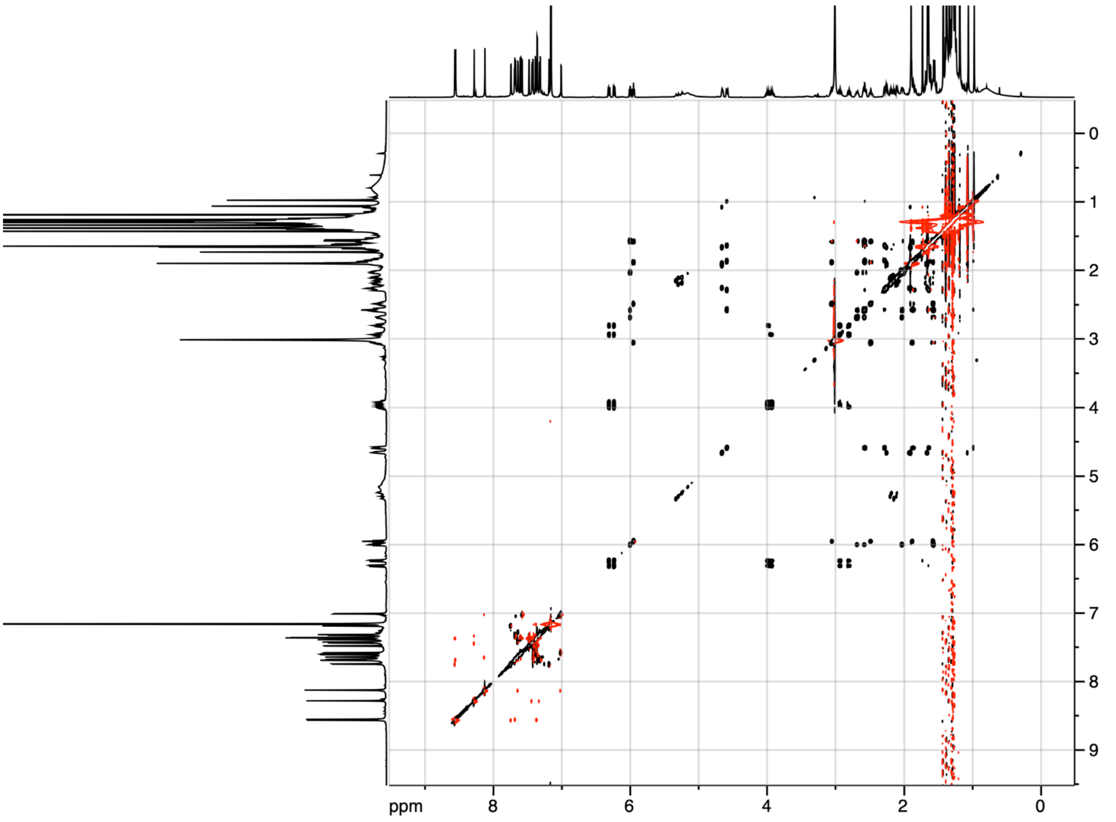

Figure S53. ^{1}H–^{1}H TOCSY NMR spectrum of [**C4**Pd_4(squalene)] (600 MHz, C_6D_6).

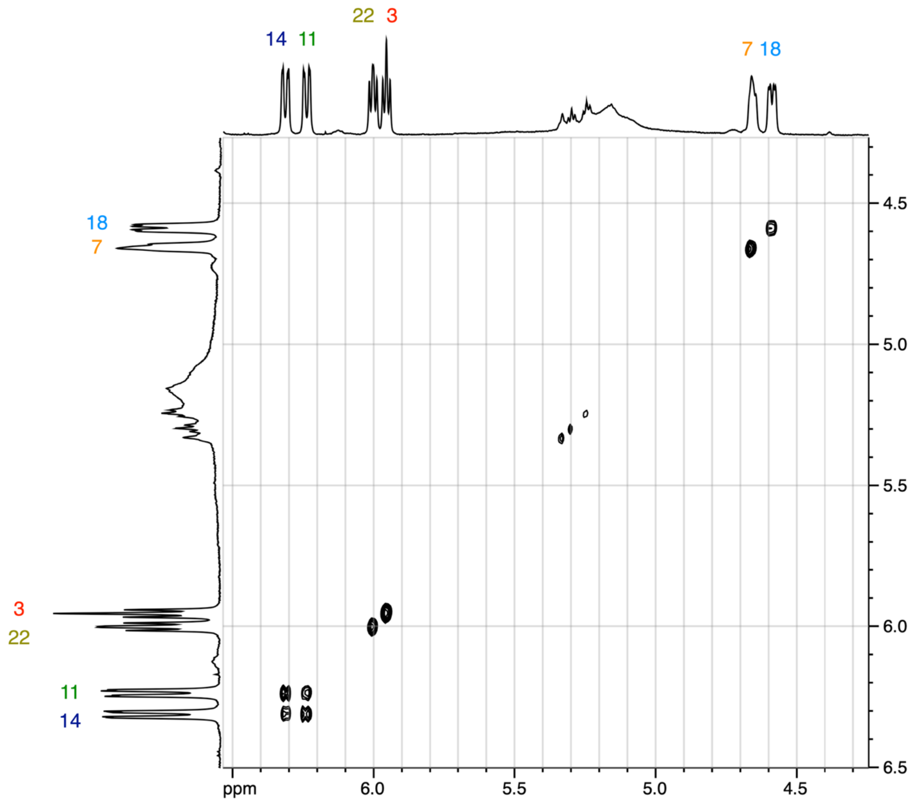

Figure S54. ^{1}H–^{1}H TOCSY NMR spectrum of [**C4**Pd_4(squalene)] (600 MHz, C_6D_6) (olefin region).

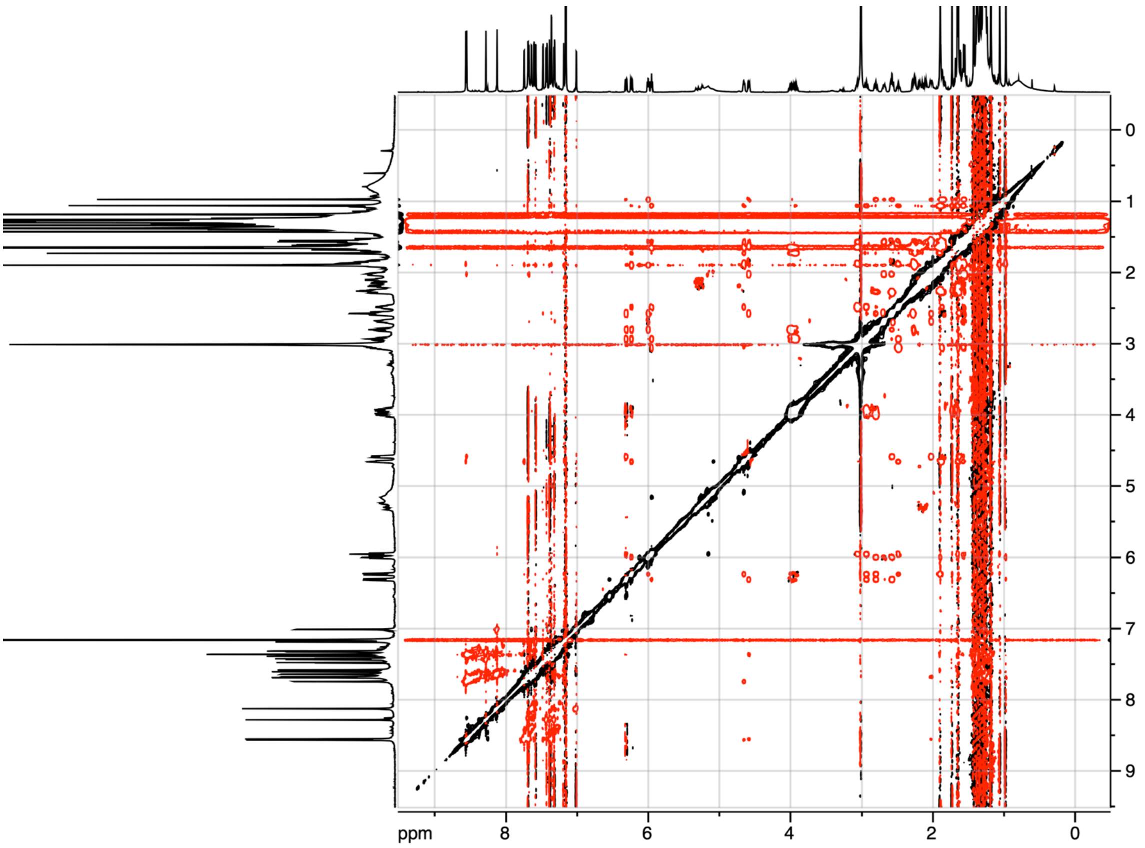

Figure S55. ^{1}H–^{1}H ROESY NMR spectrum of [**C4**Pd$_4$(squalene)] (600 MHz, C_6D_6).

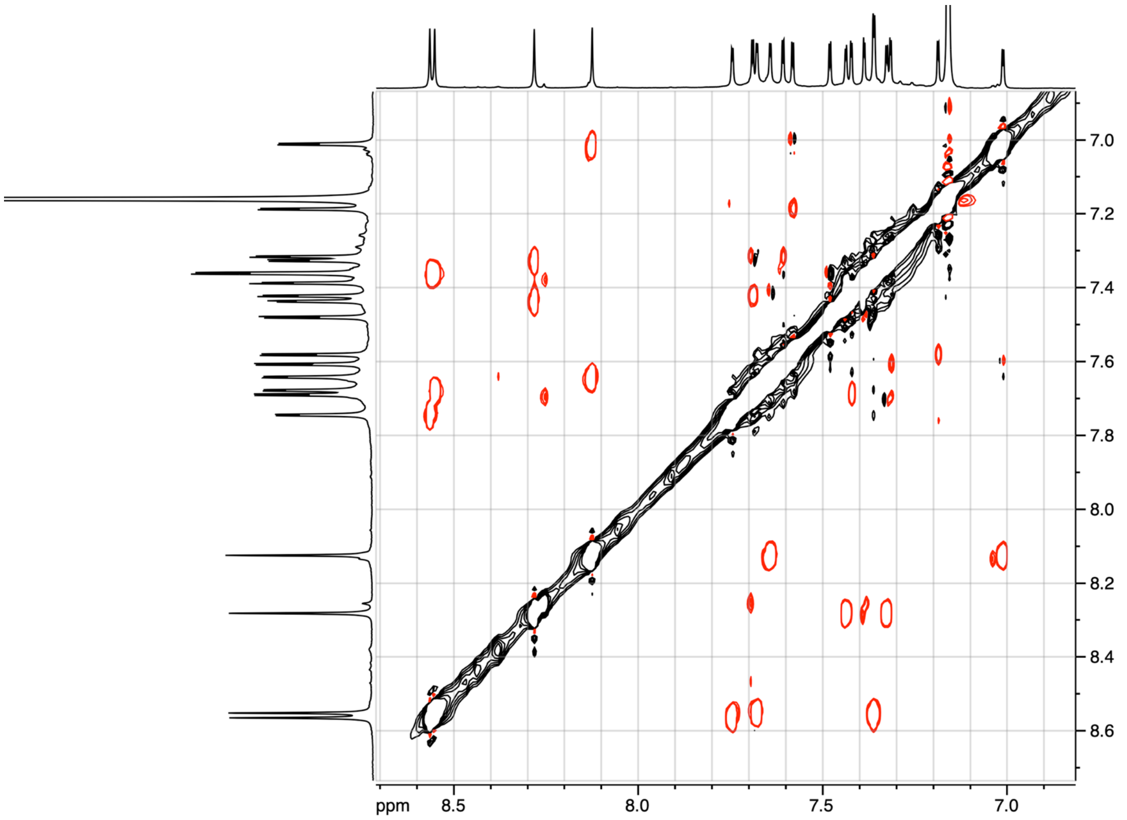

Figure S56. ^{1}H–^{1}H ROESY NMR spectrum of [**C4**Pd$_4$(squalene)] (600 MHz, C_6D_6) (aromatic and imine region).

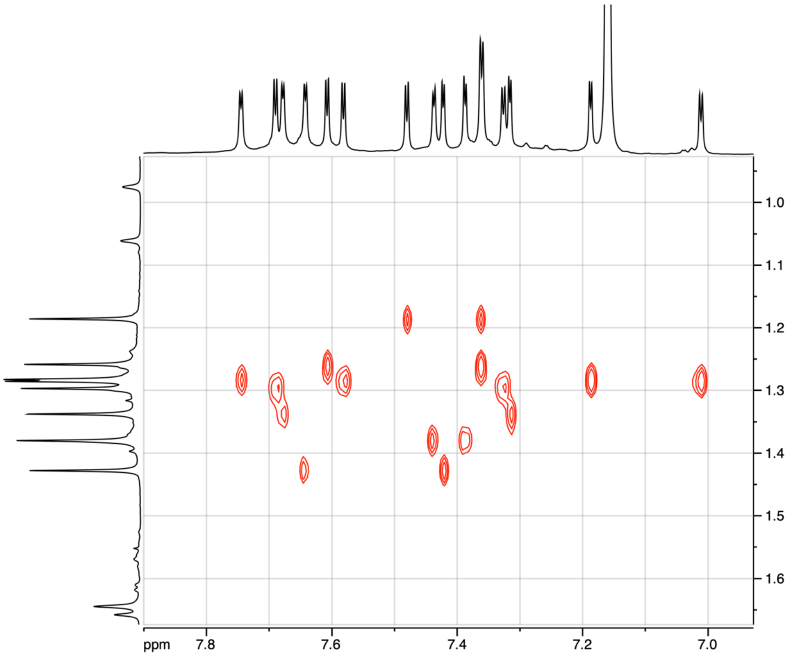

Figure S57. $^{1}H–^{1}H$ ROESY NMR spectrum of [**C4**Pd_4(squalene)] (600 MHz, C_6D_6) (aromatic–*tert*-butyl cross peaks).

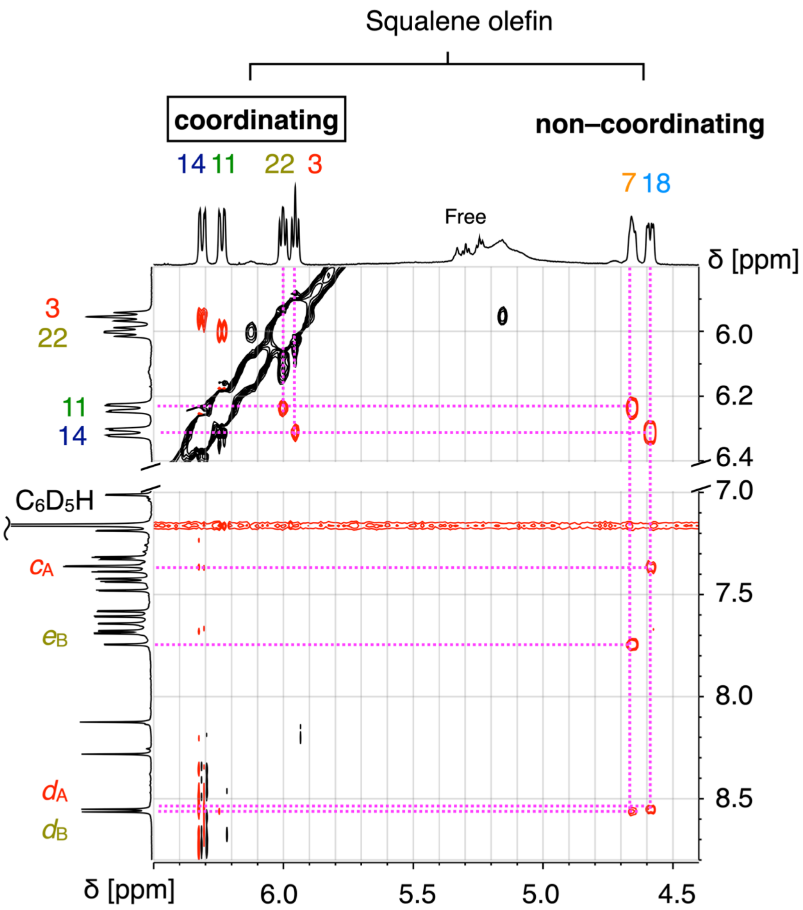

Figure S58. $^{1}H–^{1}H$ ROESY NMR spectrum of [**C4**Pd_4(squalene)] (600 MHz, C_6D_6) (olefin–olefin and olefin–host complex cross peaks).

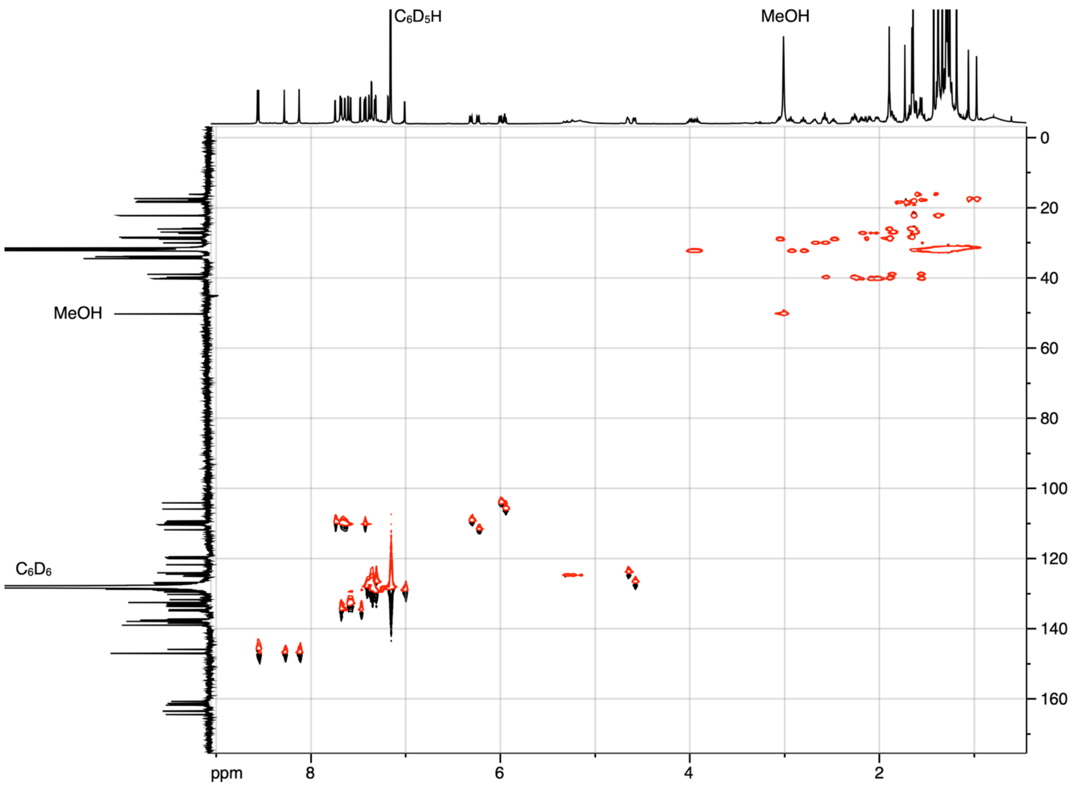

Figure S59. ^{1}H–^{13}C HSQC NMR spectrum of [**C4**Pd$_4$(squalene)] (600 MHz, C_6D_6).

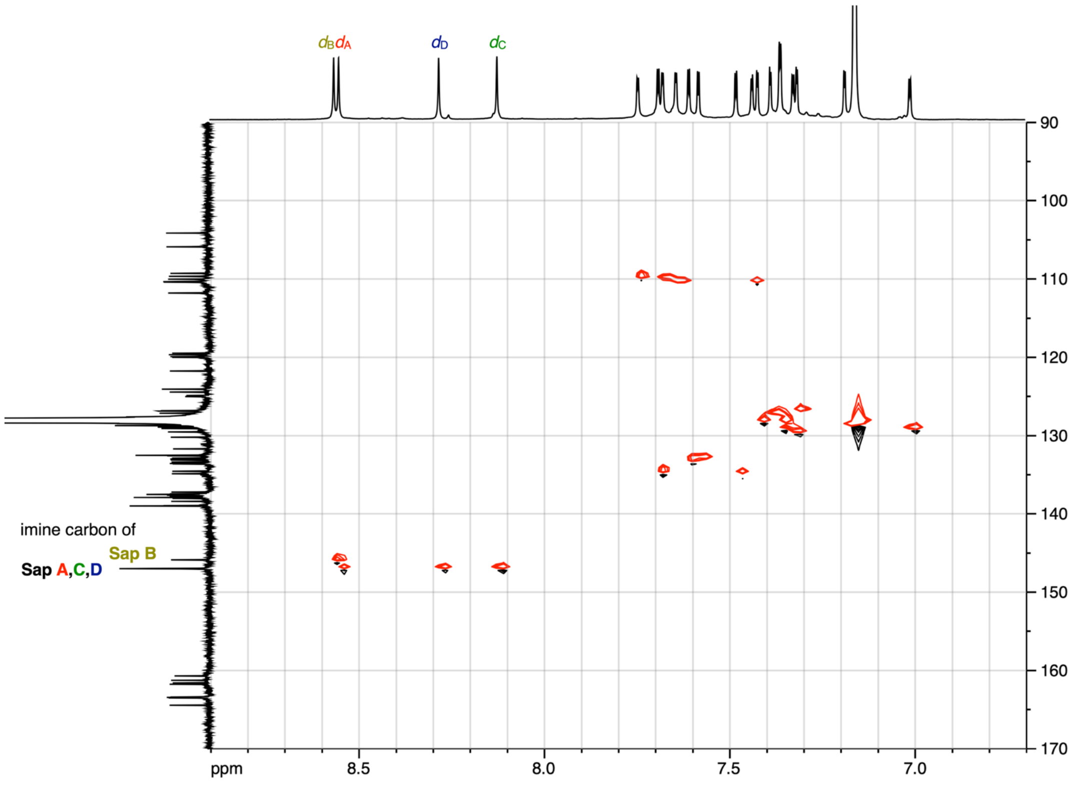

Figure S60. ^{1}H–^{13}C HSQC NMR spectrum of [**C4**Pd$_4$(squalene)] (600 MHz, C_6D_6) (aromatic and imine region).

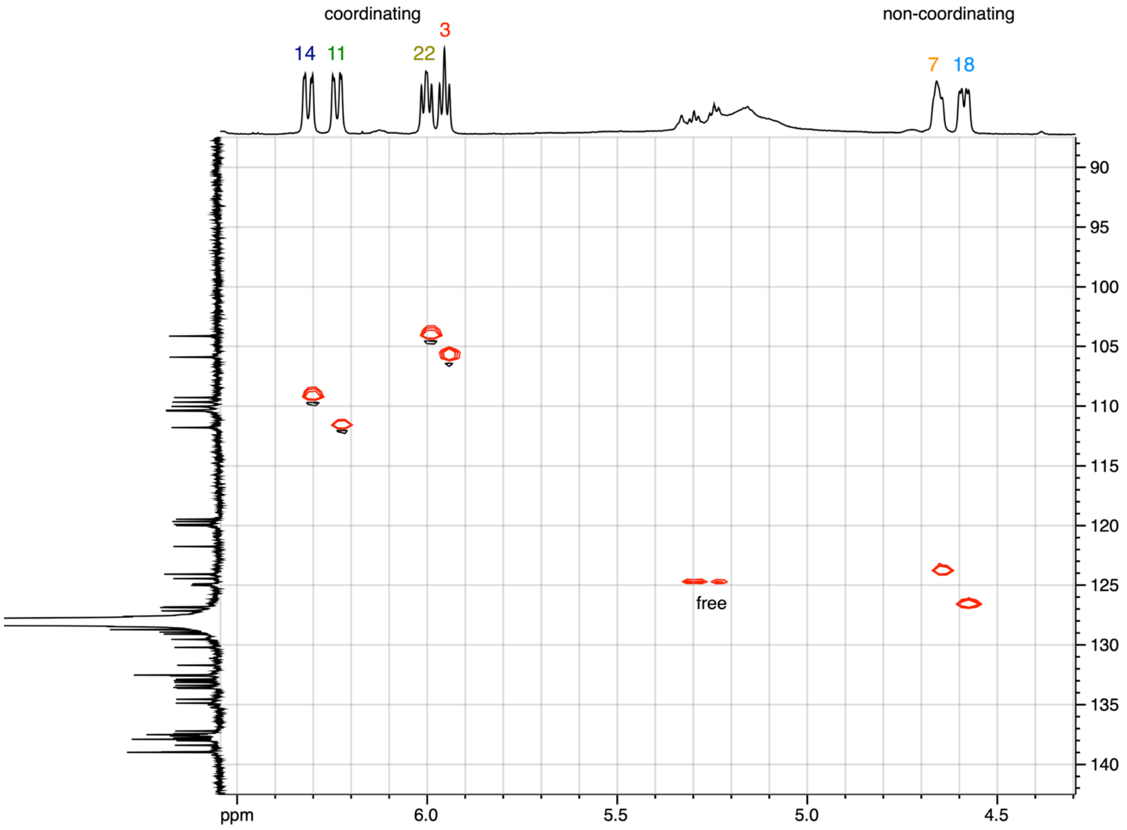

Figure S61. ^{1}H–^{13}C HSQC NMR spectrum of [**C4**Pd_4(squalene)] (600 MHz, C_6D_6) (olefin region).

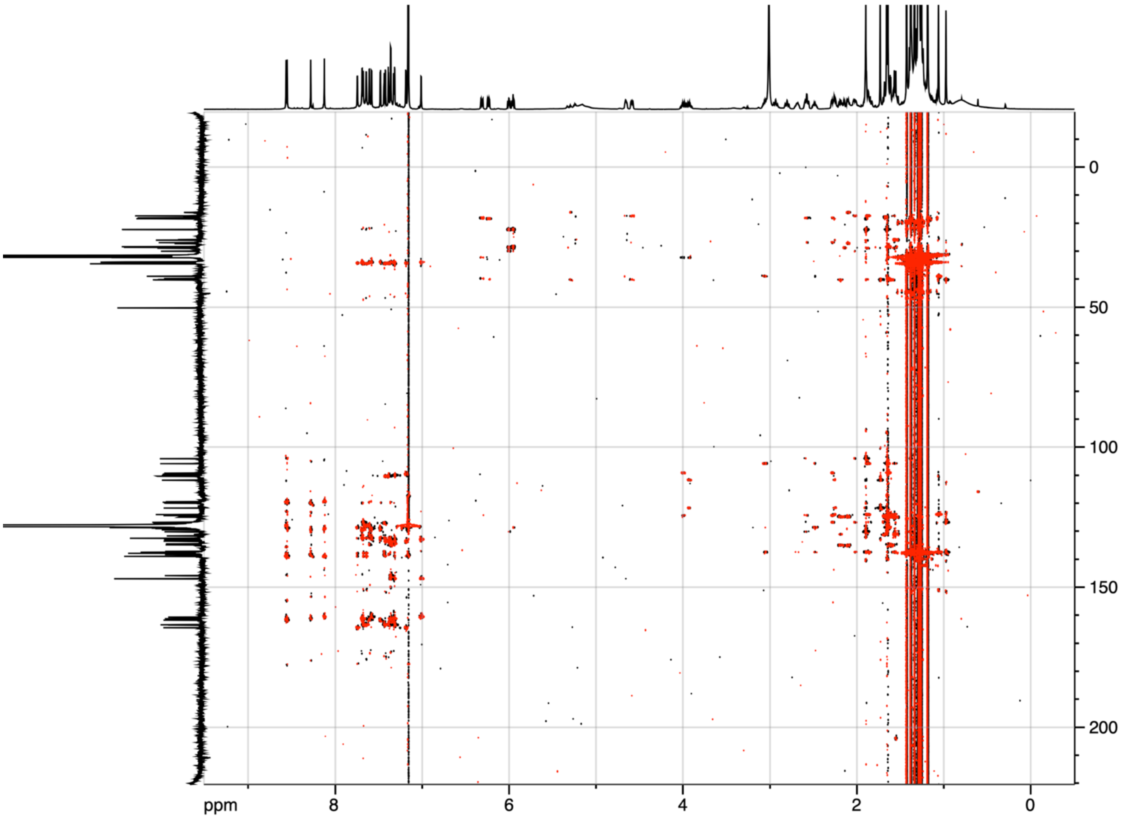

Figure S62. $^{1}H–^{13}C$ HMBC NMR spectrum of [**C4**Pd$_4$(squalene)] (600 MHz, C_6D_6).

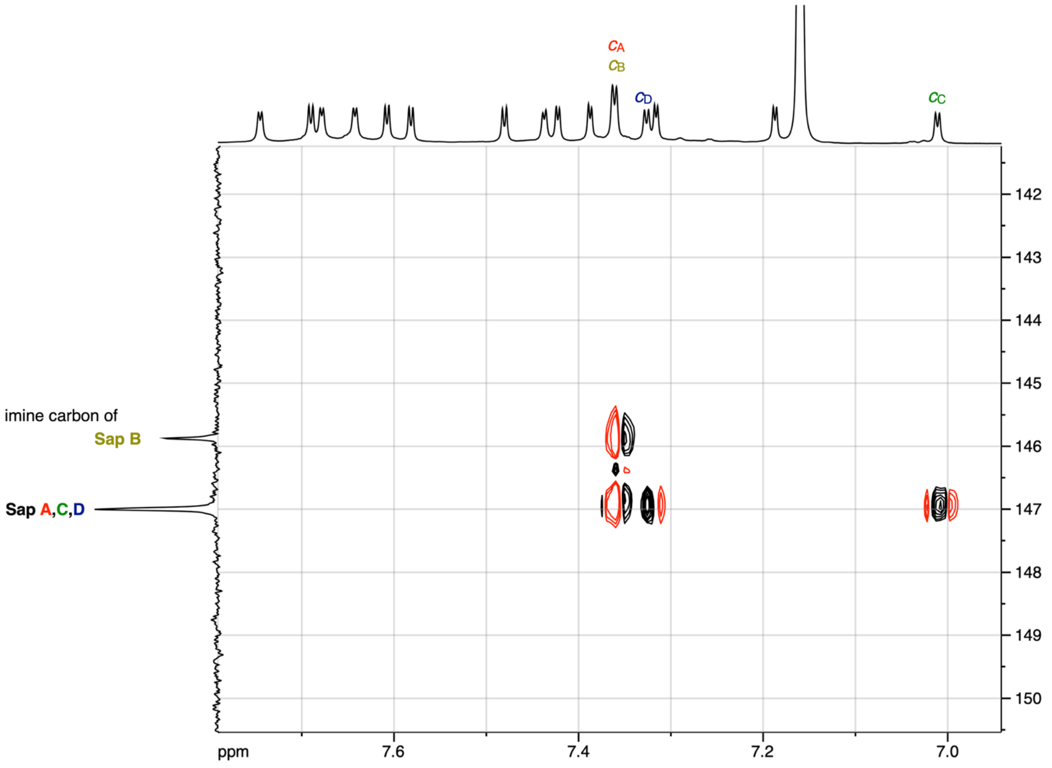

Figure S63. $^{1}H–^{13}C$ HMBC NMR spectrum of [**C4**Pd$_4$(squalene)] (600 MHz, C_6D_6) (aromatic proton–imine carbon cross peak).

Assignment of the squalene olefin ^{1}H

Cross peaks were observed between the internal olefin protons **11** and **14** in the ^{1}H–^{1}H TOCSY NMR spectrum (Figure S54). Cross peaks were observed between the terminal olefin protons **3**, **22** and the corresponding terminal two methyl protons in the ^{1}H–^{1}H COSY NMR spectrum (Figure S52). The relationships between each squalene unit were determined by cross peaks between the olefin protons and methylene protons in the ^{1}H–^{1}H ROESY NMR spectrum. Diastereotopic methylene protons were assigned through cross peaks of the folded squalene protons in the ^{1}H–^{1}H ROESY NMR spectrum.

Assignment of the host molecule, Pd-tetrasap complex

Regarding the aromatic protons, the *c* and *e* protons were assigned based on cross peaks with imine protons in the ^{1}H–^{1}H ROESY NMR spectrum (Figure S56), and the *c* protons were confirmed by cross peaks with imine carbons in the ^{1}H–^{13}C HMBC NMR spectrum (Figure S63). The pairs of (*b*,*c*) and (*e*,*f*) were determined by the ^{1}H–^{1}H COSY NMR spectrum (Figure S51). The relationships between the sap units were determined by ROE correlations of the biphenyl units (Figure S56). *tert*-Butyl groups were assigned through ROE correlations with the aromatic protons (Figure S57).

Assignment of the connection of host-guest complex

The assignment of the host-guest complex was determined by ROE correlations between squalene and Pd-tetrasap (Figure S55), and further confirmed by the structure obtained from the X-ray diffraction analysis (Figure S64). The host-guest complex exhibits helicity derived from the folding orientation of squalene (see next section), and the structure shown in Figure S48 corresponds to *M*-[**C4**Pd_4(squalene)] chirality. All ROE correlations were consistent with the structure determined by X-ray (e.g., Figure S58 and Figure S67), supporting that the structure of [**C4**Pd_4(squalene)] observed in the crystal state is also present in solution.

6-3. X-ray diffraction analysis of [**C4**Pd_4(squalene)]

A single crystal of [**C4**Pd_4(squalene)] suitable for the X-ray diffraction analysis was obtained by slow evaporation from recrystallization of acetone vapor diffusion to $CDCl_3$ solution of [**C4**$Pd_4(MeOH)_{3.0}(H_2O)_{0.9}(AcOH)_{0.1}$] + squalene 1.0 equiv. (20 mM).

Refinements were performed using reflection data of 1.18 Å since the values of mean $F_0^2/\sigma(F_0^2)$ in the resolution shell between 1.19 and 1.13 Å was 1.71. The low resolution was due to the small size and insufficient quality of the crystal. Solvent accessible voids of 5922 Å^3 (38.9 % of the cell volume, calculated by SQUEEZE) were found, where solvents ($CDCl_3$ or acetone) were heavily disordered. The residual electron density was treated with SQUEEZE[63]. Global RIGU and SIMU restraints were applied. SADI and ISOR restraints were applied for disordered *t*Bu groups. DFIX and DANG restraints were applied for imine moiety due to small disorder of imine C=N directions. DFIX and DANG restraints were applied for parts of squalene C-C single bonds and ISOR restraints was applied for some atoms of squalene.

Crystallographic data for $C_{114}H_{142}N_4O_8Pd_4$, *Fw* = 2121.91, orange block, 0.10 × 0.04 × 0.04 mm^3, monoclinic, space group $P2_1/c$ (No. 14), a = 12.496(2) Å, b = 28.419(6) Å, c = 42.924(8) Å, β = 93.888(2)°, V = 15209(5) Å^3, Z = 4, T = 100 K, λ = 0.71073 Å, θ_{max} = 17.499°, R_1 = 0.0598 ($I > 2\sigma$, after SQUEEZE), wR_2 = 0.1716 (total, after SQUEEZE), GOF = 1.032. CCDC 2412786.

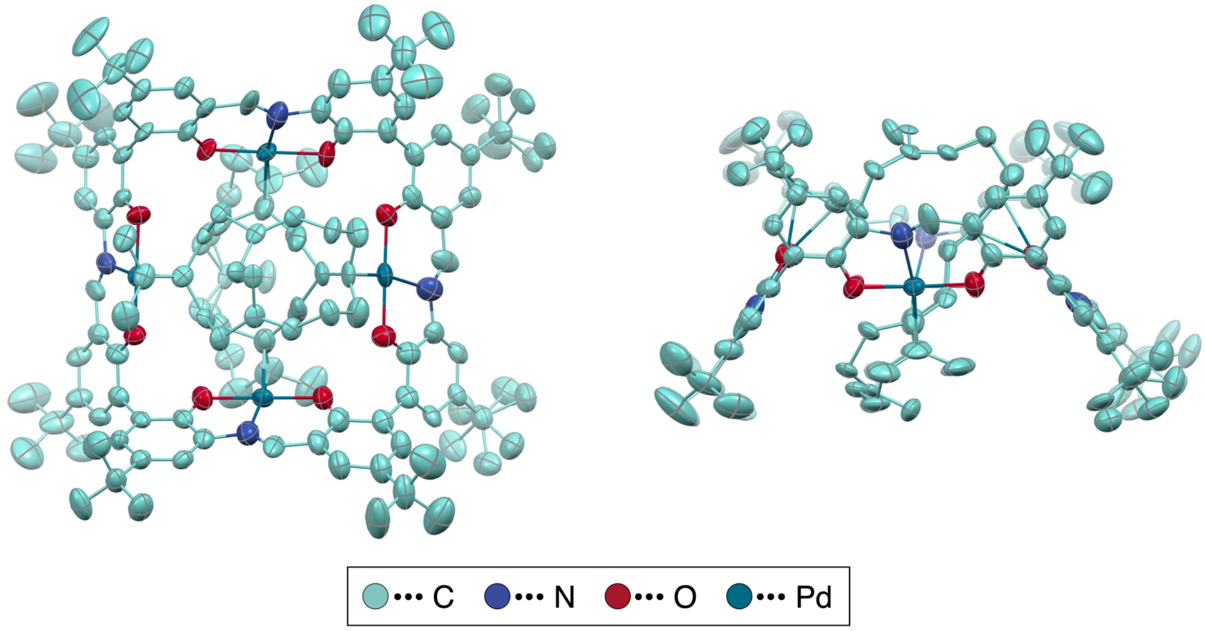

Figure S64. The structure of palladium-tetrasap squalene complex determined by X-ray diffraction analysis. An ellipsoidal model (30% probability). H atoms are not shown.

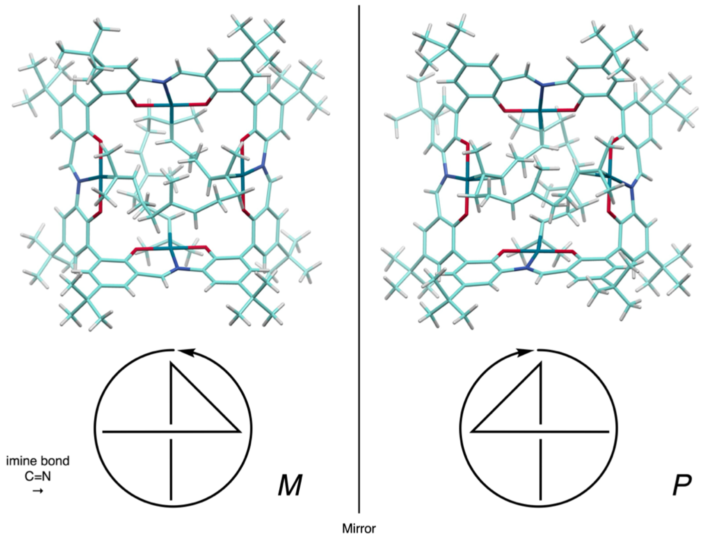

Figure S65. Helicity of [**C4**Pd$_4$(squalene)] (Left: *M*-[**C4**Pd$_4$(squalene)], Right: *P*-[**C4**Pd$_4$(squalene)]).

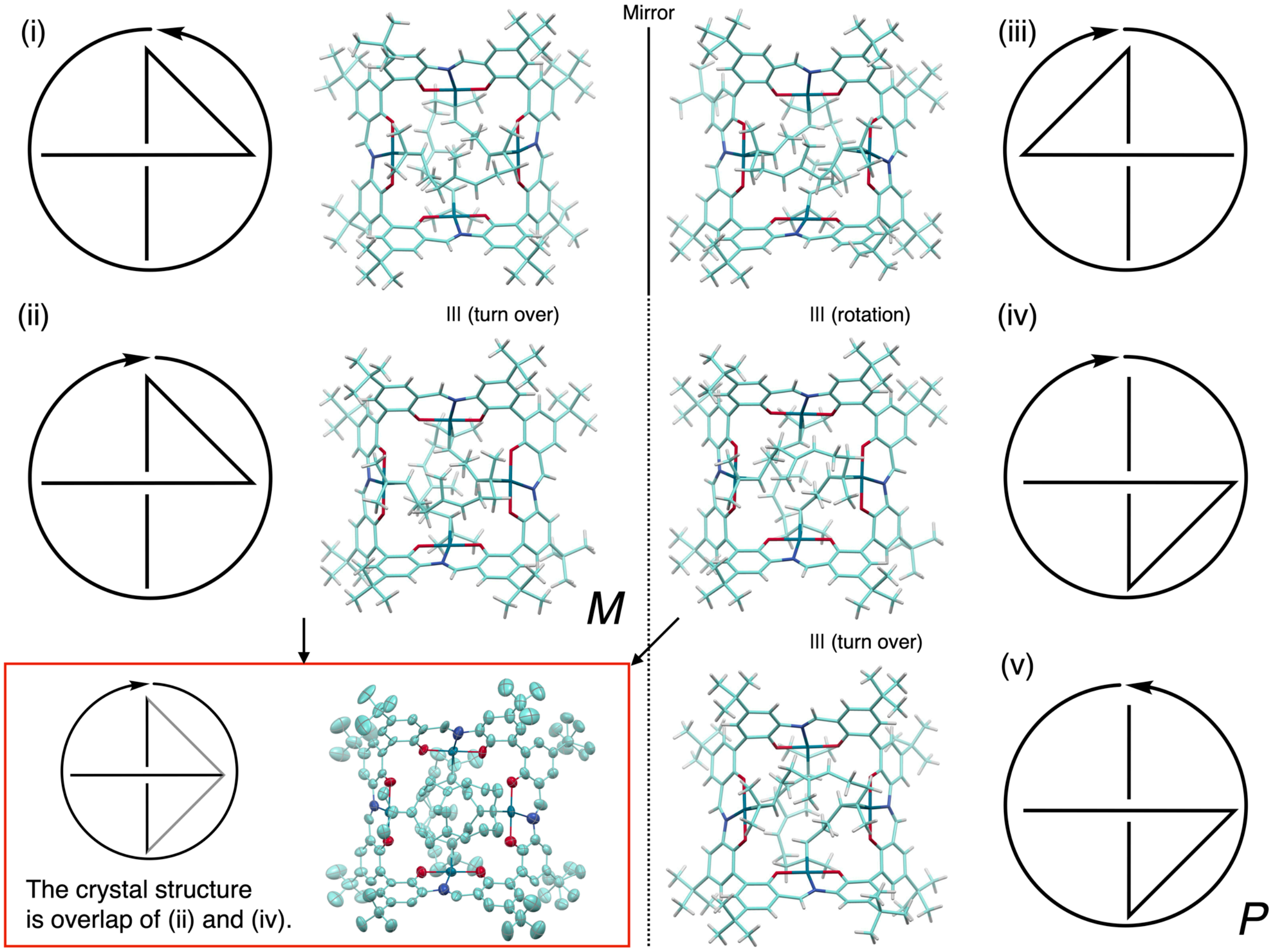

Figure S66. Helicity of [**C4**Pd$_4$(squalene)].

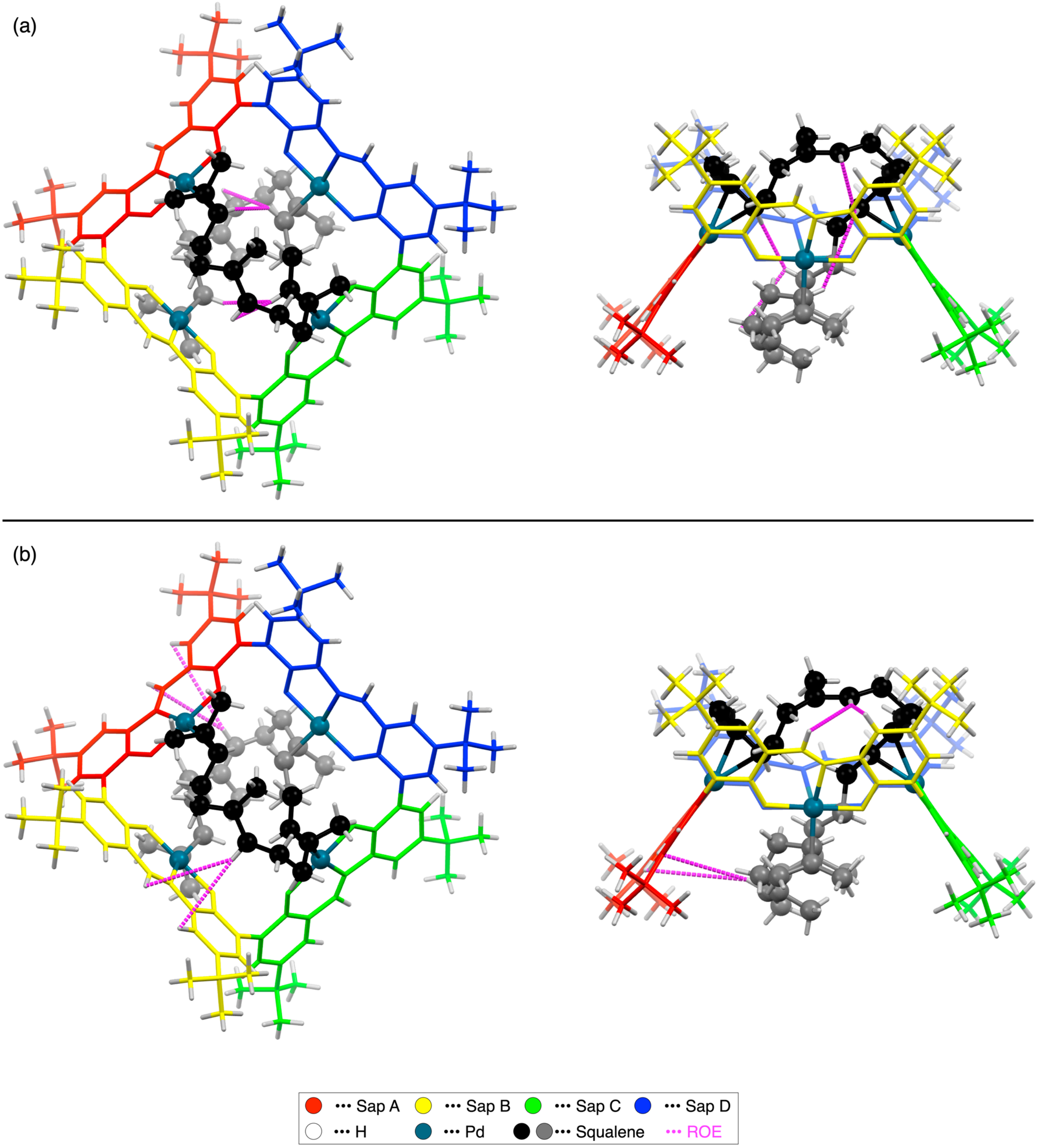

Figure S67. The ball and stick model of palladium-tetrasap squalene complex determined by X-ray diffraction analysis. Dashed lines in magenta show the ROE correlations in Figure S58. (a) Correlations between squalene olefin – squalene olefin. (b) Correlations between squalene olefin – Pd-tetrasap imine and aromatic protons.

6-4. Addition of squalene against Pd-hexapap

The coordination binding ability of squalene was investigated for Pd-hexapap[27], which was previously developed in our group.

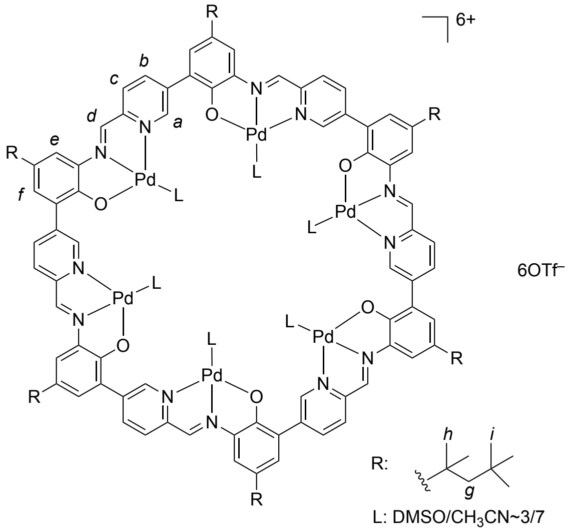

Figure S68. The structure of Pd-hexapap.

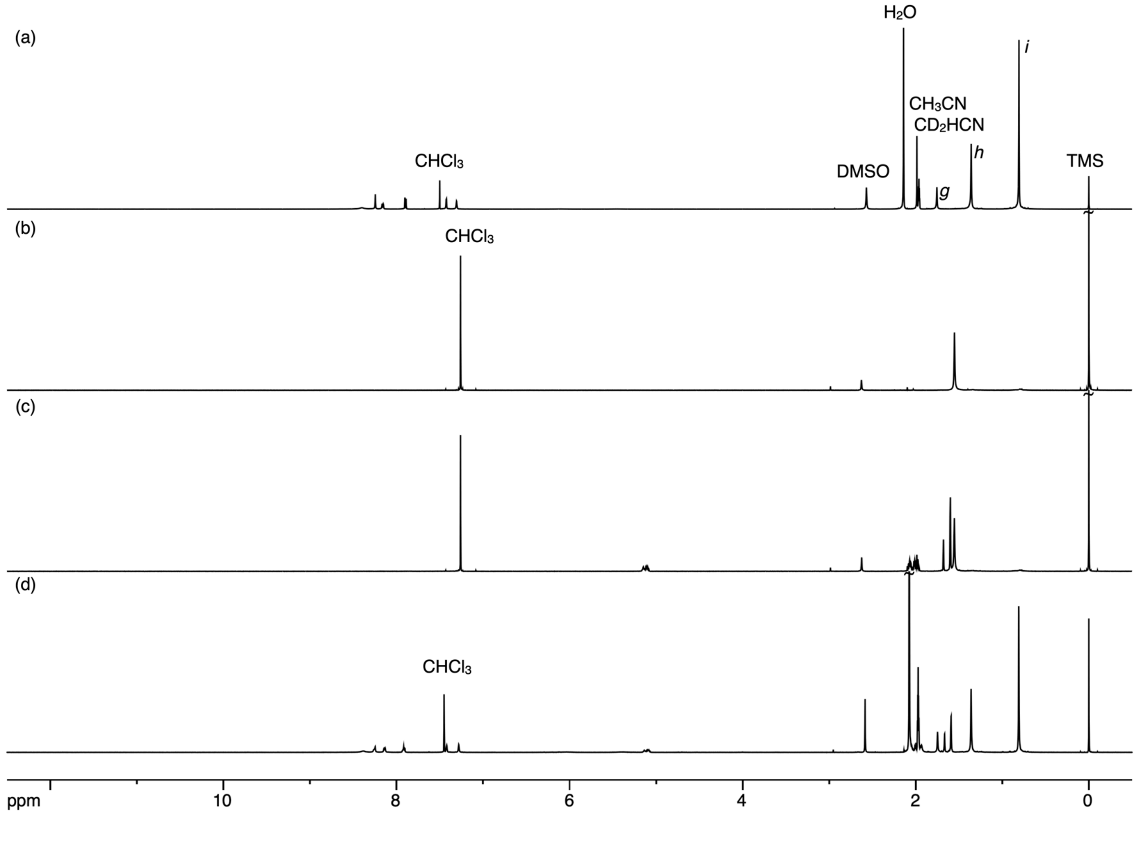

Figure S69. A coordinative binding trial of squalene to Pd-hexapap (^{1}H NMR, 600 MHz). (a) Pd-hexapap ($CDCl_3/CD_3CN$ = 1/1). (b) Pd-hexapap ($CDCl_3$). (c) Pd-hexapap + squalene 1.0 equiv. ($CDCl_3$). (d) Pd-hexapap + squalene 1.0 equiv. ($CDCl_3/CD_3CN$ = 2/1).

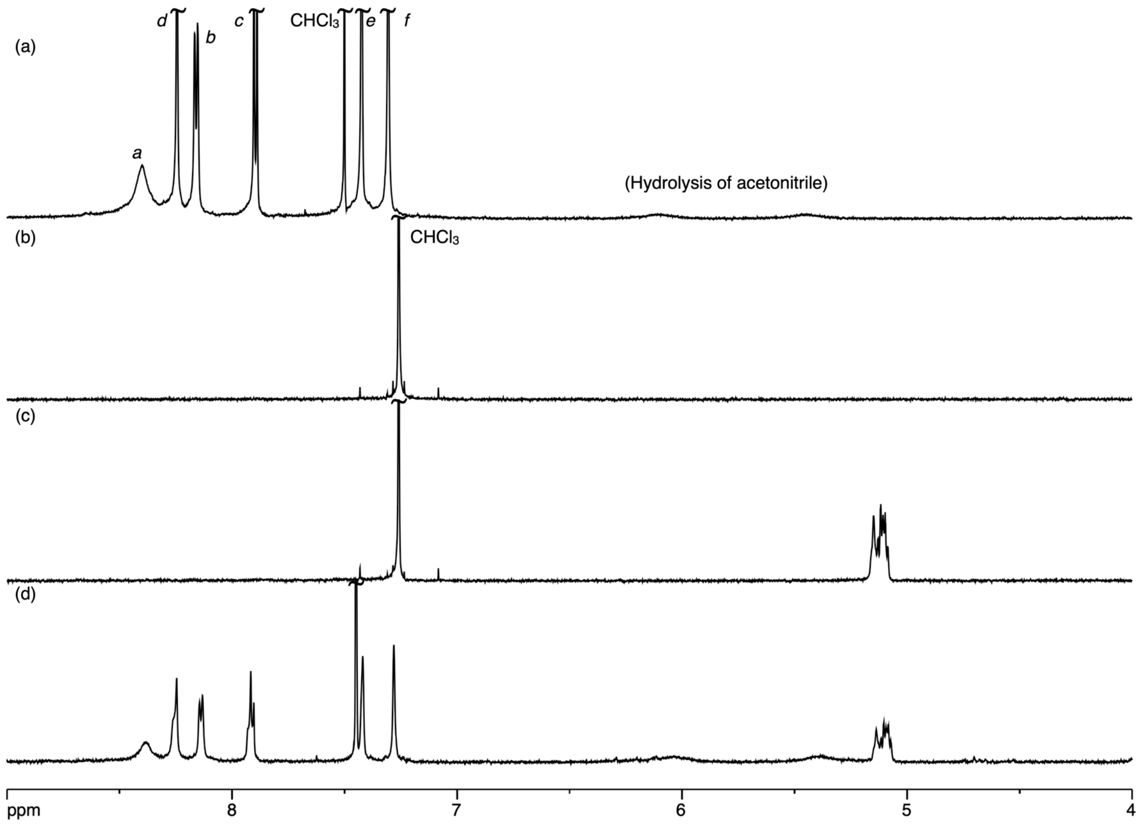

Figure S70. Enlarged ^{1}H NMR spectra of Figure S69.

Pd-hexapap was not soluble in chloroform and, therefore, did not react with squalene in $CDCl_3$. While Pd-hexapap was soluble in $CDCl_3/CD_3CN$ = 2/1, acetonitrile coordinated to the palladium, preventing the coordination of squalene.

7. Coordination binding experiments of other isoprenoids

^{1}H NMR titration experiments of other isoprenoids were conducted in $CDCl_3$ solution in a manner similar to that of the squalene titration.

7-1. Geraniol

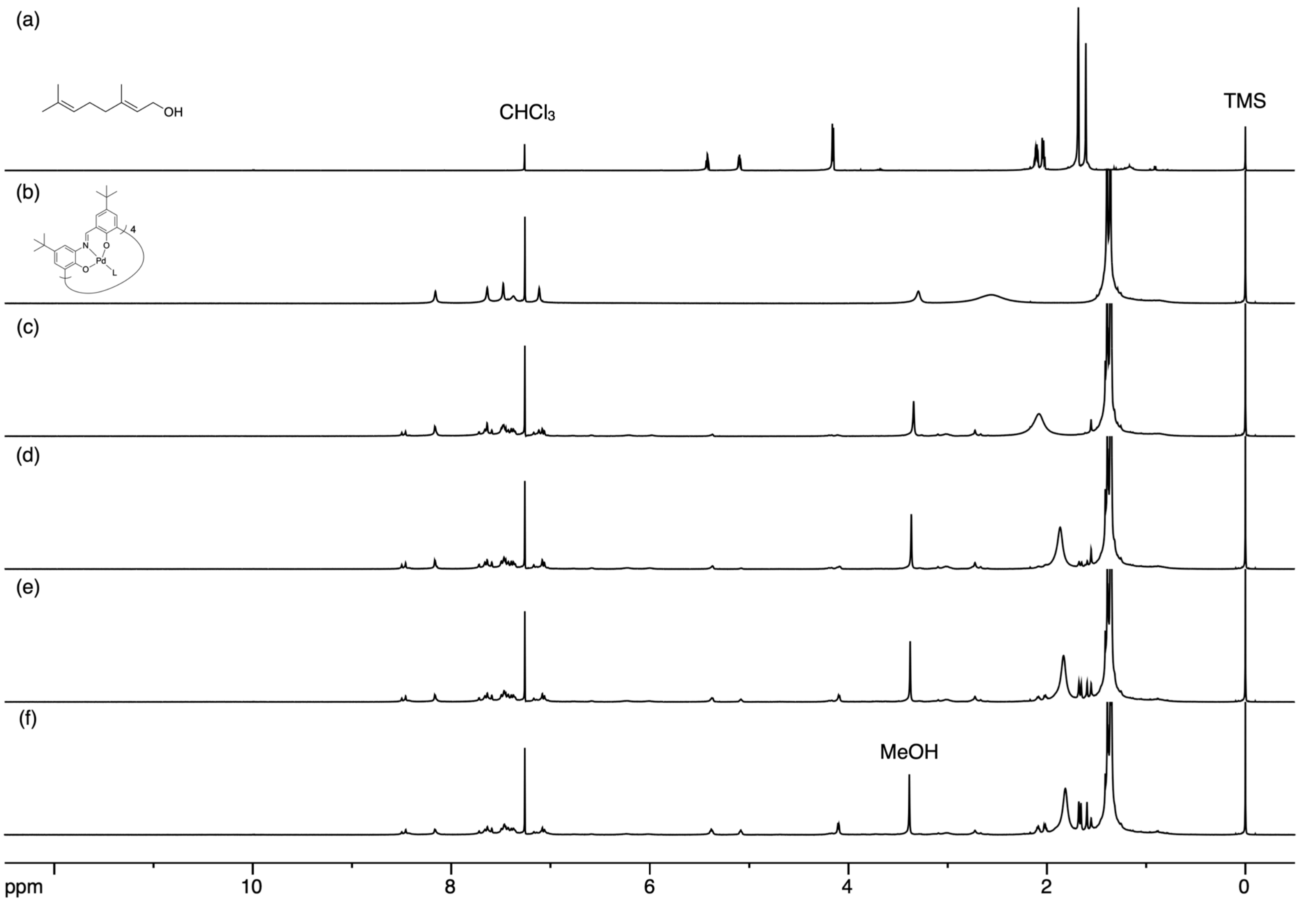

Figure S71. Titration of geraniol to [**C4**Pd_4L_4] in $CDCl_3$ investigated by ^{1}H NMR measurements. (a–f) ^{1}H NMR spectra (600 MHz, $CDCl_3$). (a) Geraniol. (b) [**C4**Pd_4L_4]. (c) [**C4**Pd_4L_4] + geraniol 0.50 equiv. (d) [**C4**Pd_4L_4] + geraniol 1.0 equiv. (e) [**C4**Pd_4L_4] + geraniol 1.5 equiv. (f) [**C4**Pd_4L_4] + geraniol 2.0 equiv. L_4 = $(MeOH)_{3.0}(H_2O)_{0.9}(AcOH)_{0.1}$.

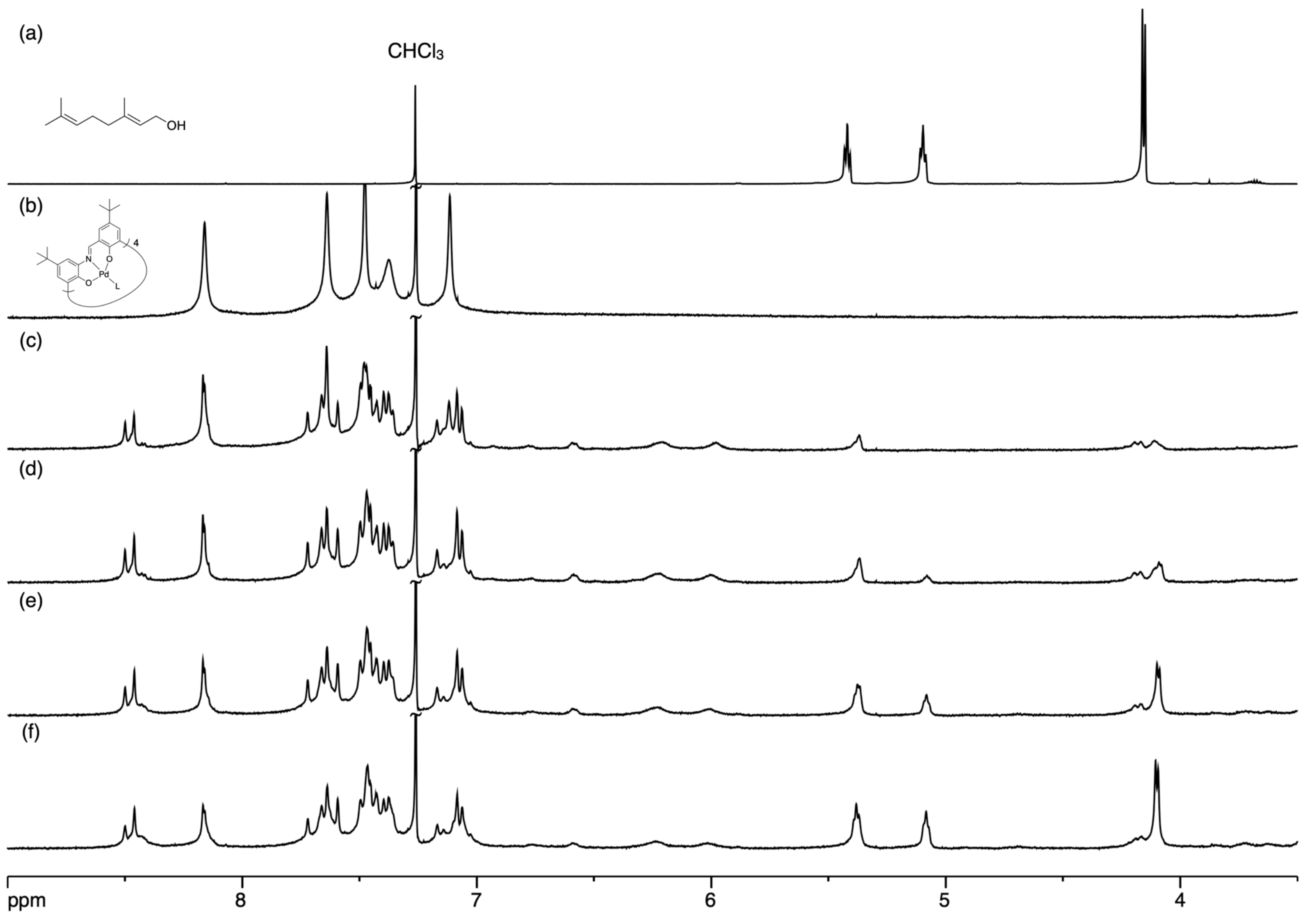

Figure S72. Enlarged ^{1}H NMR spectra of Figure S71.

7-2. Farnesol (mixture of isomers)

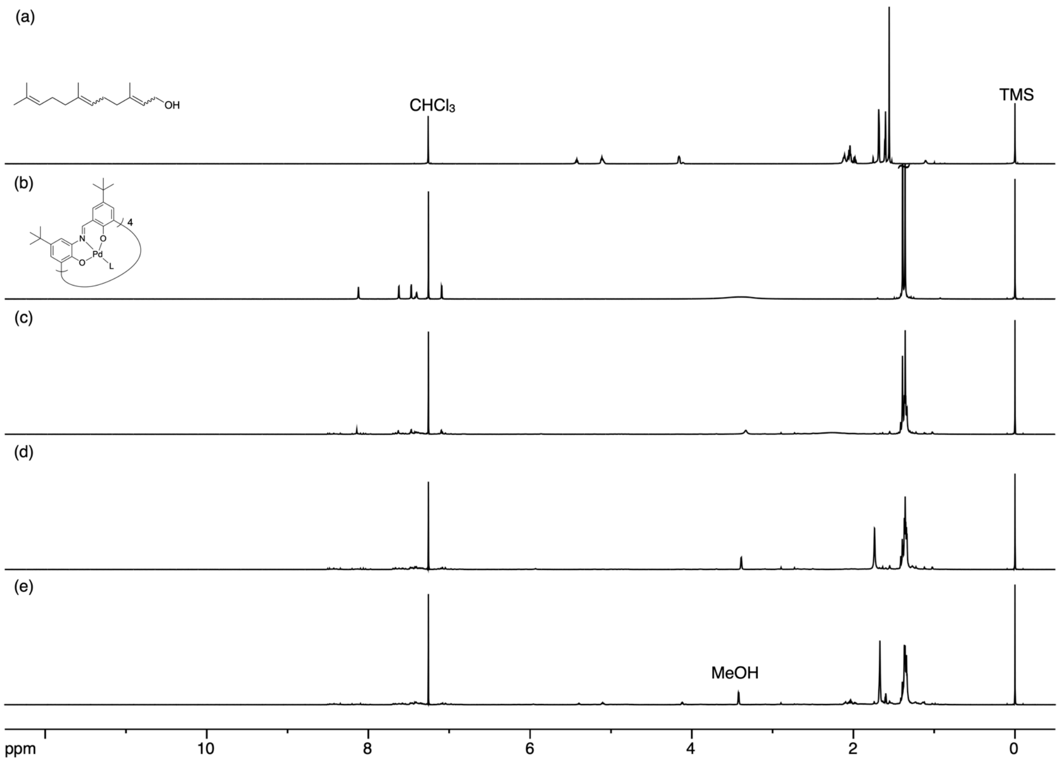

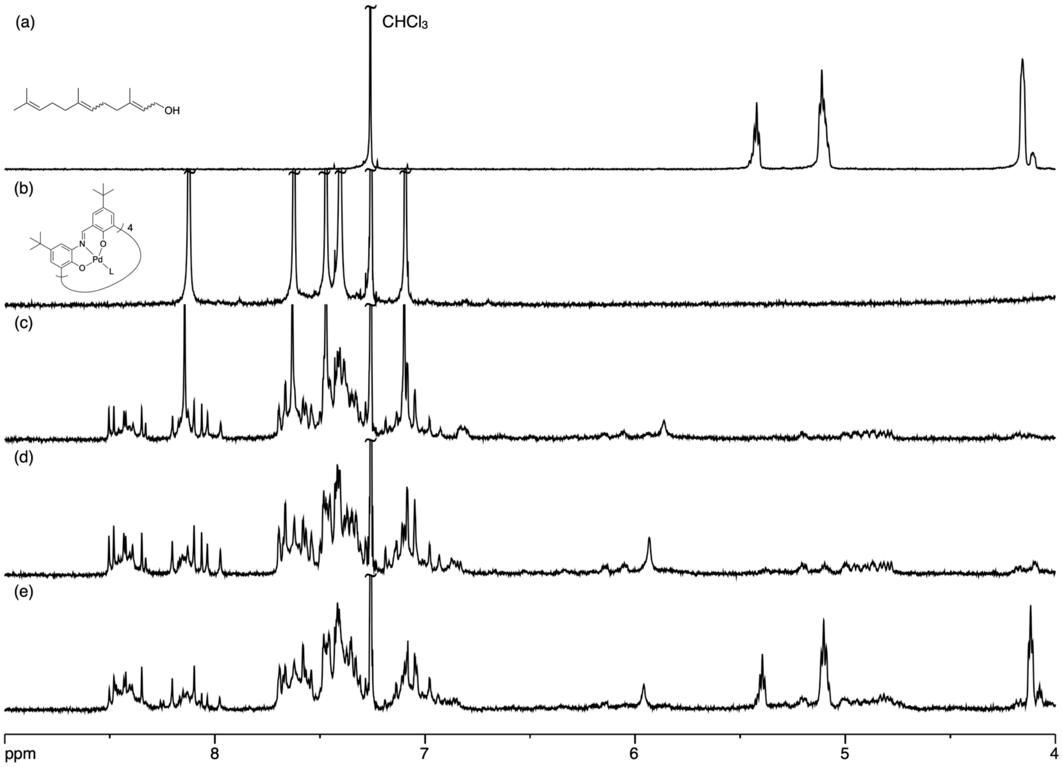

Figure S73. Titration of farnesol to [**C4**Pd_4L_4] in $CDCl_3$ investigated by ^{1}H NMR measurements. (a–e) ^{1}H NMR spectra (600 MHz, $CDCl_3$). (a) Farnesol. (b) [**C4**Pd_4L_4]. (c) [**C4**Pd_4L_4] + farnesol 0.56 equiv. (d) [**C4**Pd_4L_4] + farnesol 1.1 equiv. (e) [**C4**Pd_4L_4] + farnesol 2.3 equiv. $L_4 = (MeOH)_{2.0}(H_2O)_{2.0}$.

7-3. Solanesol

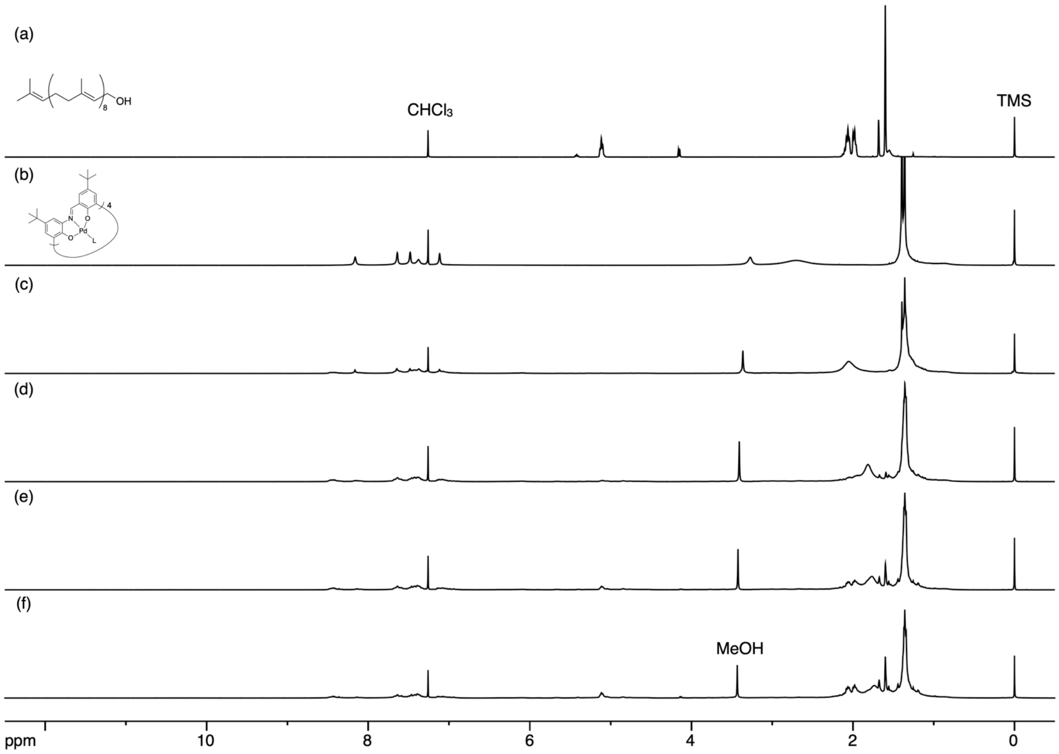

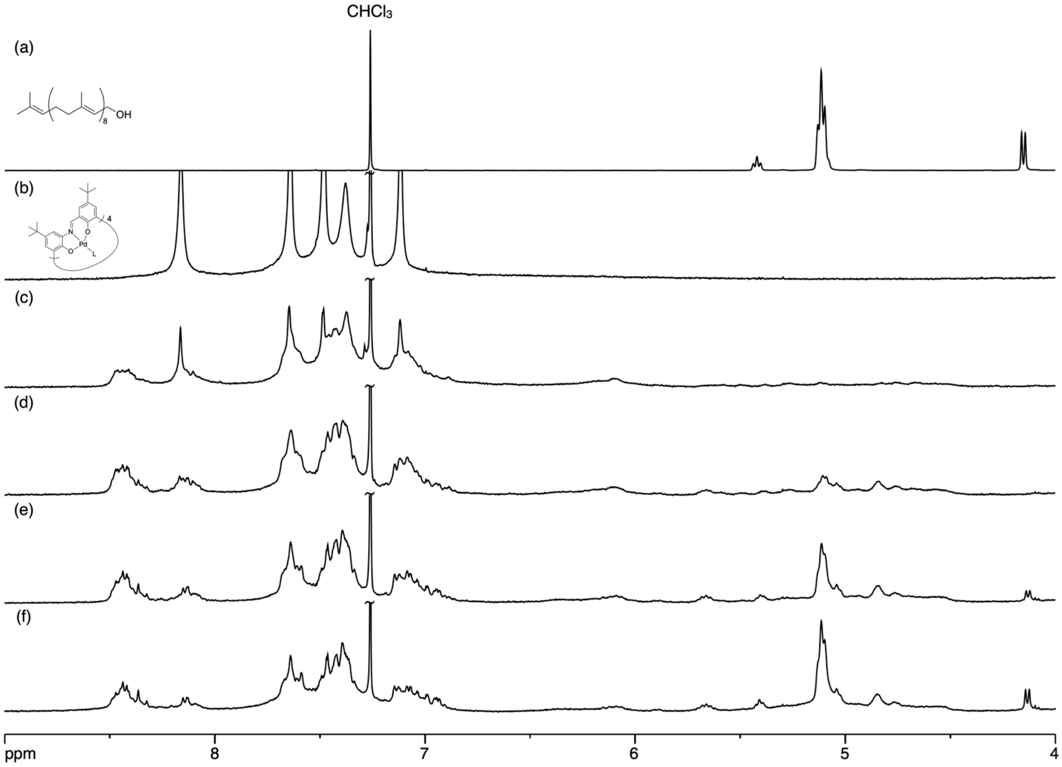

Figure S74. Titration of solanesol to [**C4**Pd_4L_4] in $CDCl_3$ investigated by ^1H NMR measurements. (a–f) ^1H NMR spectra (400 MHz, $CDCl_3$). (a) Solanesol. (b) [**C4**Pd_4L_4]. (c) [**C4**Pd_4L_4] + solanesol 0.25 equiv. (d) [**C4**Pd_4L_4] + solanesol 0.50 equiv. (e) [**C4**Pd_4L_4] + solanesol 0.75 equiv. (f) [**C4**Pd_4L_4] + solanesol 1.0 equiv. L_4 = $(MeOH)_{3.0}(H_2O)_{0.9}$ $(AcOH)_{0.1}$.

7-4. Coenzyme Q_{10} (ubiquinone 10)

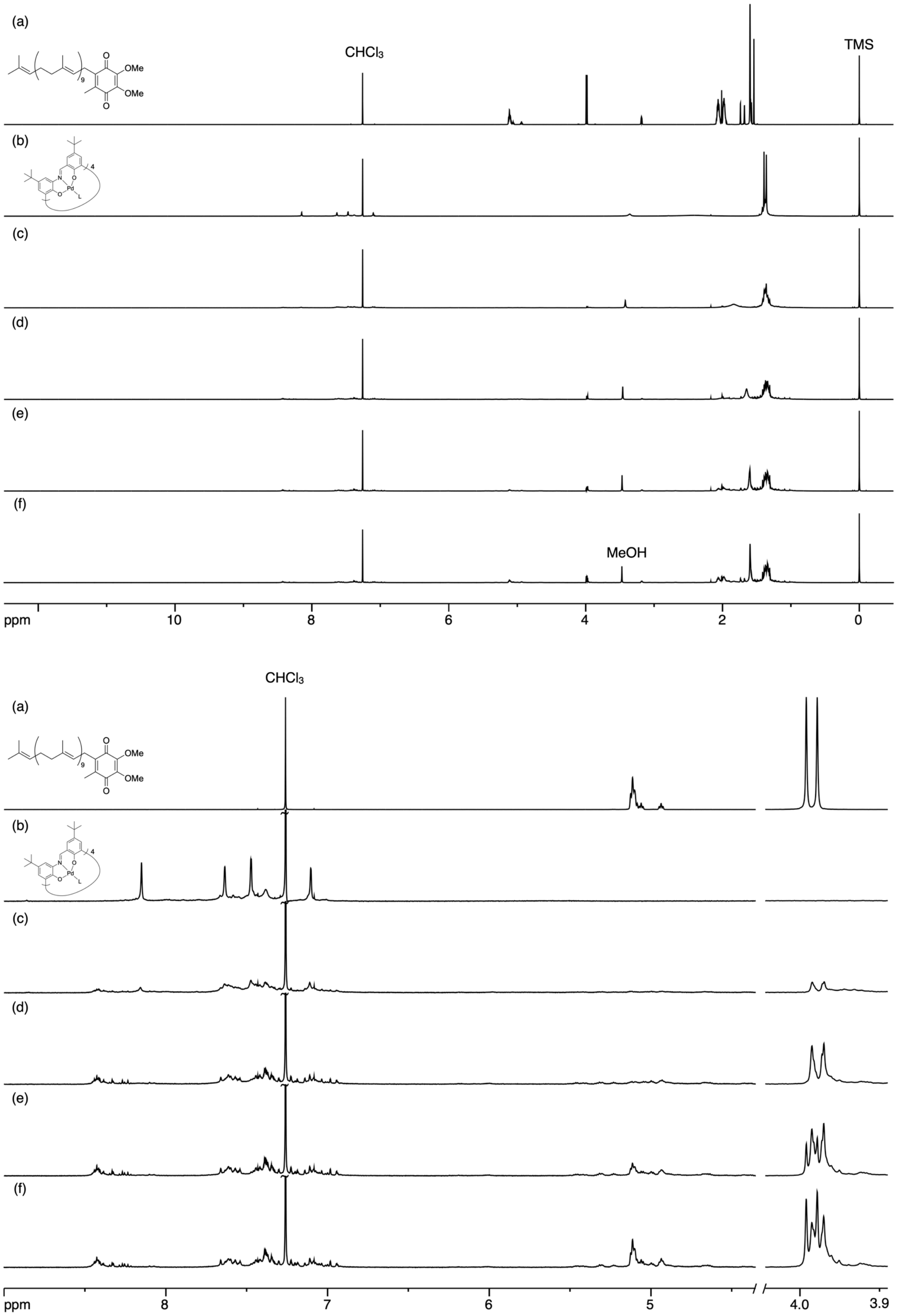

Figure S75. Titration of coenzyme Q_{10} to [**C4**Pd_4L_4] in $CDCl_3$ investigated by ^1H NMR measurements. (a–f) ^1H NMR spectra (600 MHz, $CDCl_3$). (a) Coenzyme Q_{10}. (b) [**C4**Pd_4L_4]. (c) [**C4**Pd_4L_4] + coenzyme Q_{10} 0.25 equiv. (d) [**C4**Pd_4L_4] + coenzyme Q_{10} 0.50 equiv. (e) [**C4**Pd_4L_4] + coenzyme Q_{10} 0.75 equiv. (f) [**C4**Pd_4L_4] + coenzyme Q_{10} 1.0 equiv. $L_4 = (MeOH)_{2.4}(H_2O)_{1.4}(EtOH)_{0.1}(AcOH)_{0.1}$.

7-5. 2,3-Oxidosqualene

2,3-oxidosqualene (racemic) was synthesized according to the literatures.[63]–[65]

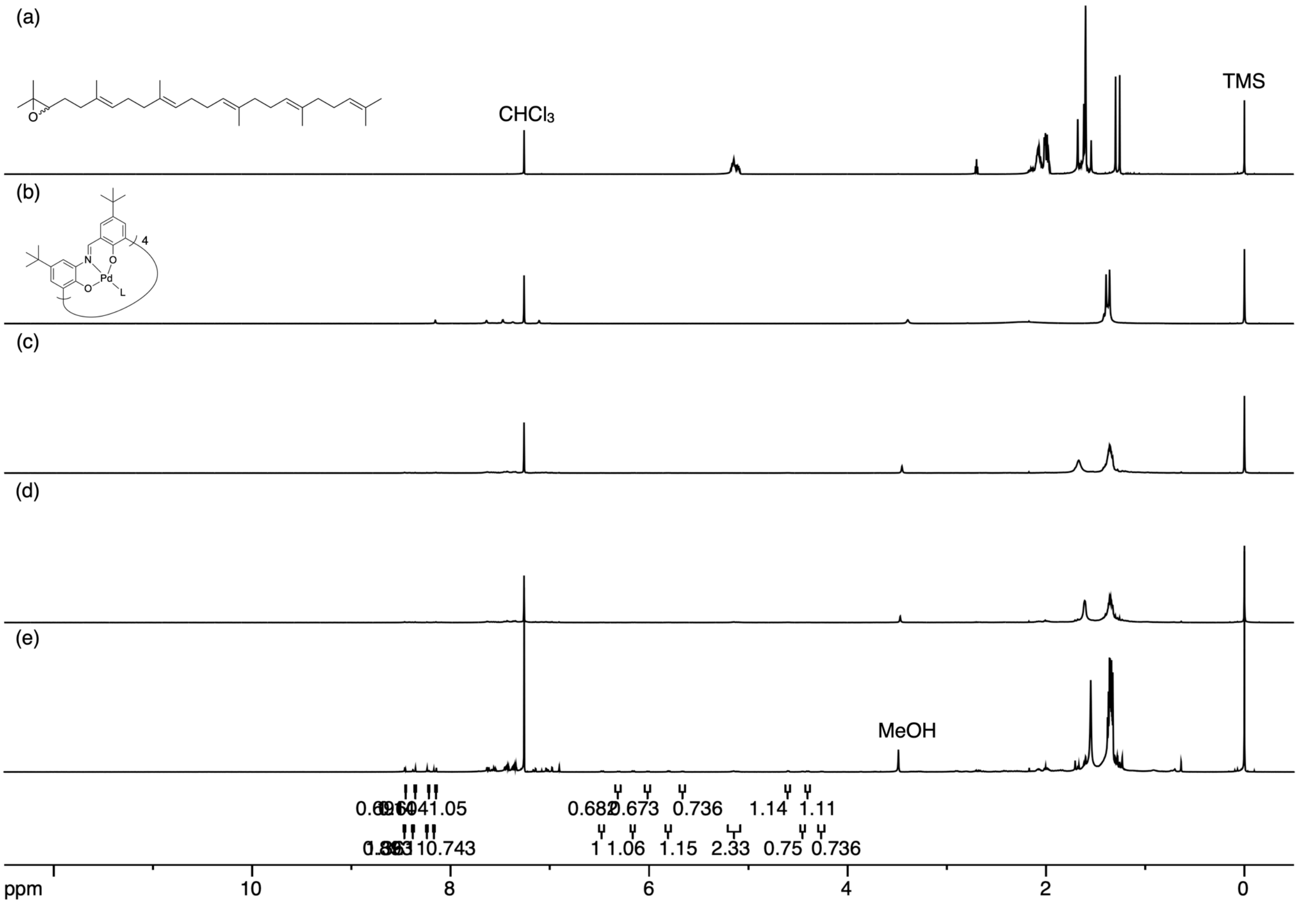

Figure S76. Titration of 2,3-oxidosqualene to [**C4**Pd_4L_4] in $CDCl_3$ investigated by ^1H NMR measurements. (a,e) ^1H NMR spectra (600 MHz, $CDCl_3$). (b–d) ^1H NMR spectra (400 MHz, $CDCl_3$). (a) 2,3-Oxidosqualene. (b) [**C4**Pd_4L_4]. (c) [**C4**Pd_4L_4] + 2,3-oxidosqualene 0.50 equiv. (d) [**C4**Pd_4L_4] + 2,3-oxidosqualene 1.0 equiv. (e) The sample (d) was heated at 50 °C for 5 h. $L_4 = (MeOH)_{2.4}(H_2O)_{1.4}(EtOH)_{0.1}(AcOH)_{0.1}$.

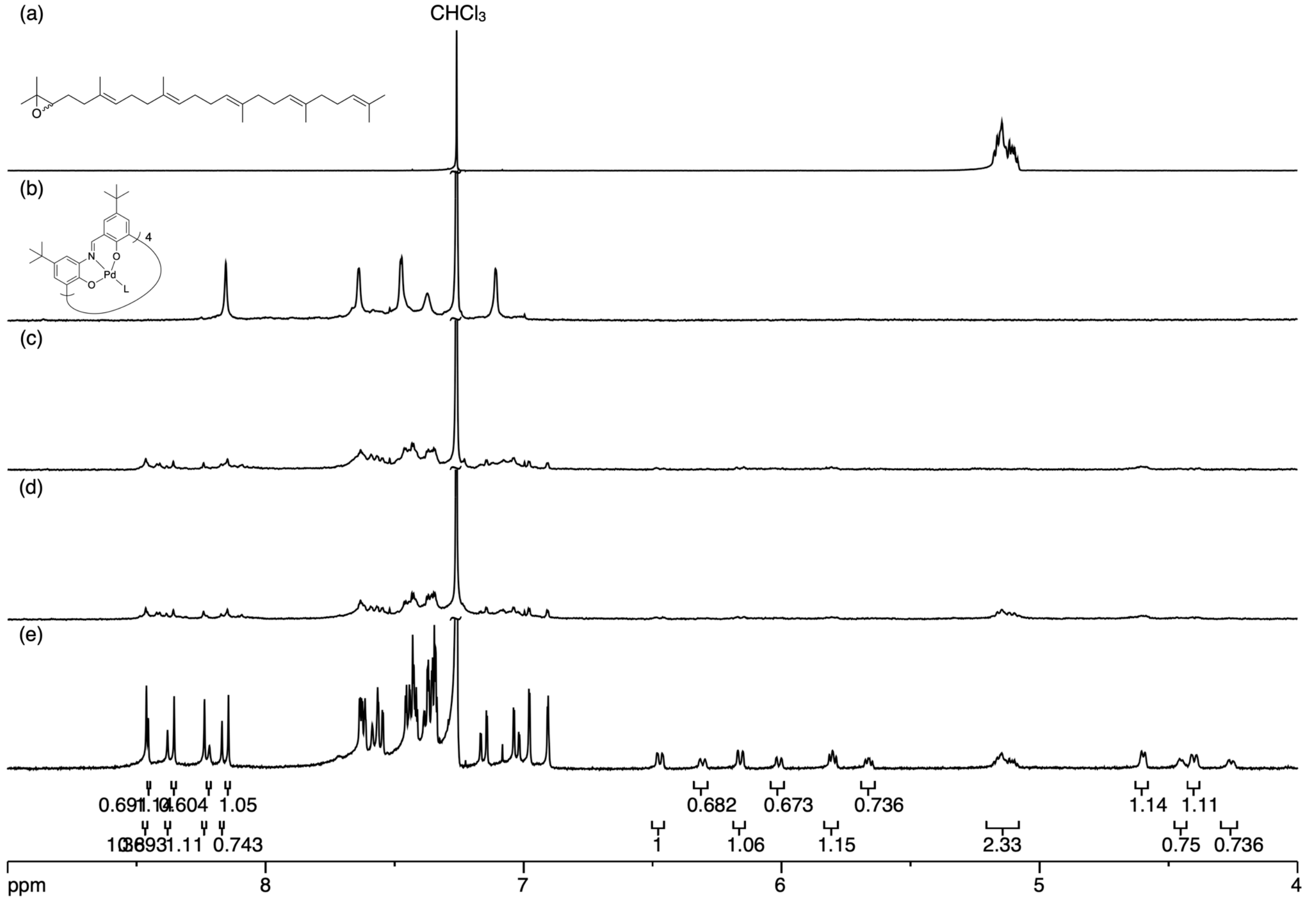

Figure S77. Enlarged ^{1}H NMR spectra of Figure S76.

The NMR spectra suggested that [**C4**Pd_4(2,3-oxidosqualene)] exists as two diastereomers. This results from the combination of the chirality of the epoxide in 2,3-oxidosqualene and the helical folding of the guest molecule.

8. Coordination binding experiments of unsaturated fatty acid methyl esters

^{1}H NMR titration experiments of unsaturated fatty acid methyl esters were conducted in $CDCl_3$ solution in a manner similar to the squalene titration.

8-1. Methyl oleate

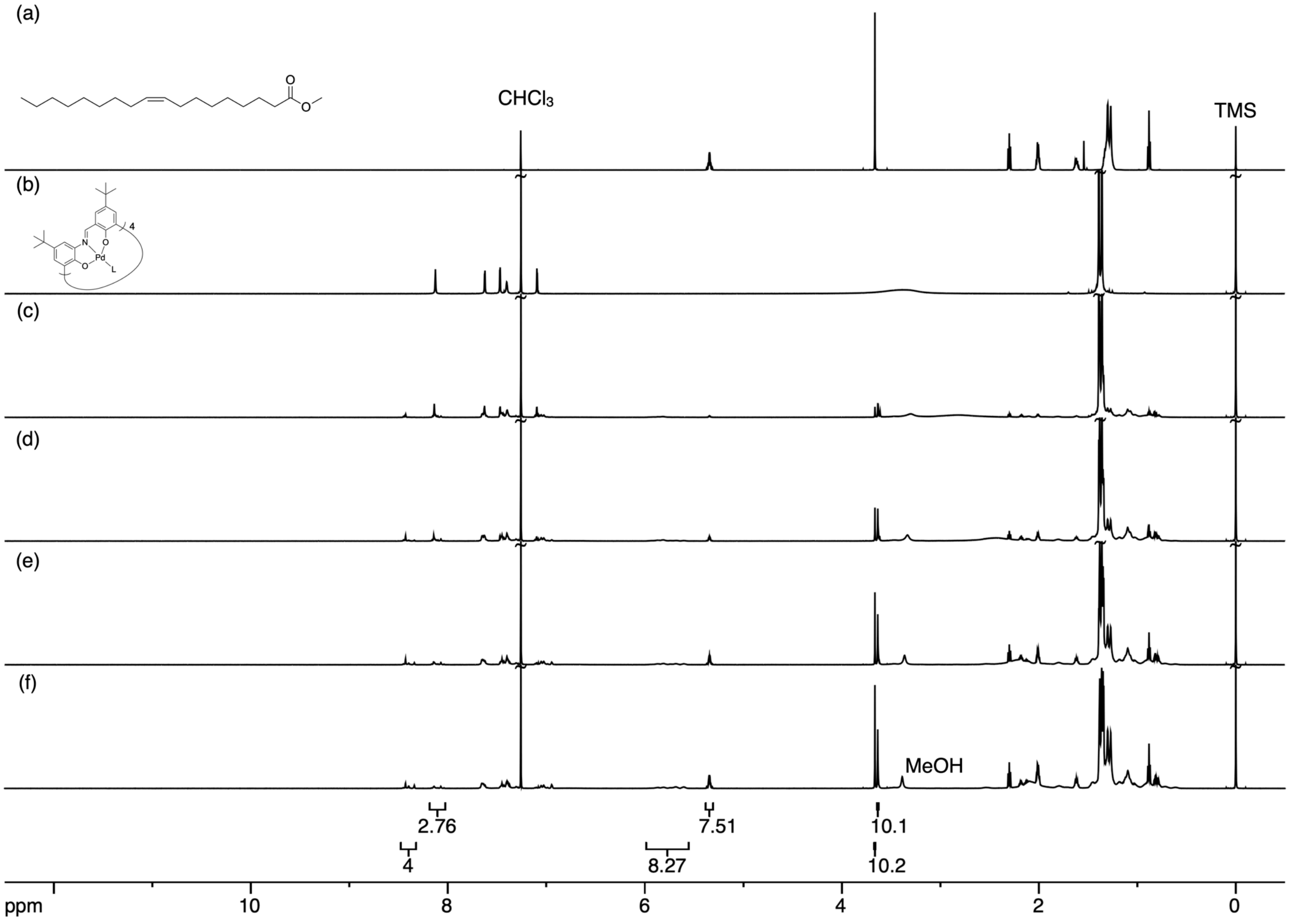

Figure S78. Titration of methyl oleate to [**C4**Pd_4L_4] in $CDCl_3$ investigated by ^{1}H NMR measurements. (a–f) ^{1}H NMR spectra (600 MHz, $CDCl_3$). (a) Methyl oleate. (b) [**C4**Pd_4L_4]. (c) [**C4**Pd_4L_4] + methyl oleate 1.1 equiv. (d) [**C4**Pd_4L_4] + methyl oleate 2.3 equiv. (e) [**C4**Pd_4L_4] + methyl oleate 3.4 equiv. (f) [**C4**Pd_4L_4] + methyl oleate 4.5 equiv. $L_4 = (MeOH)_{2.0}(H_2O)_{2.0}$.

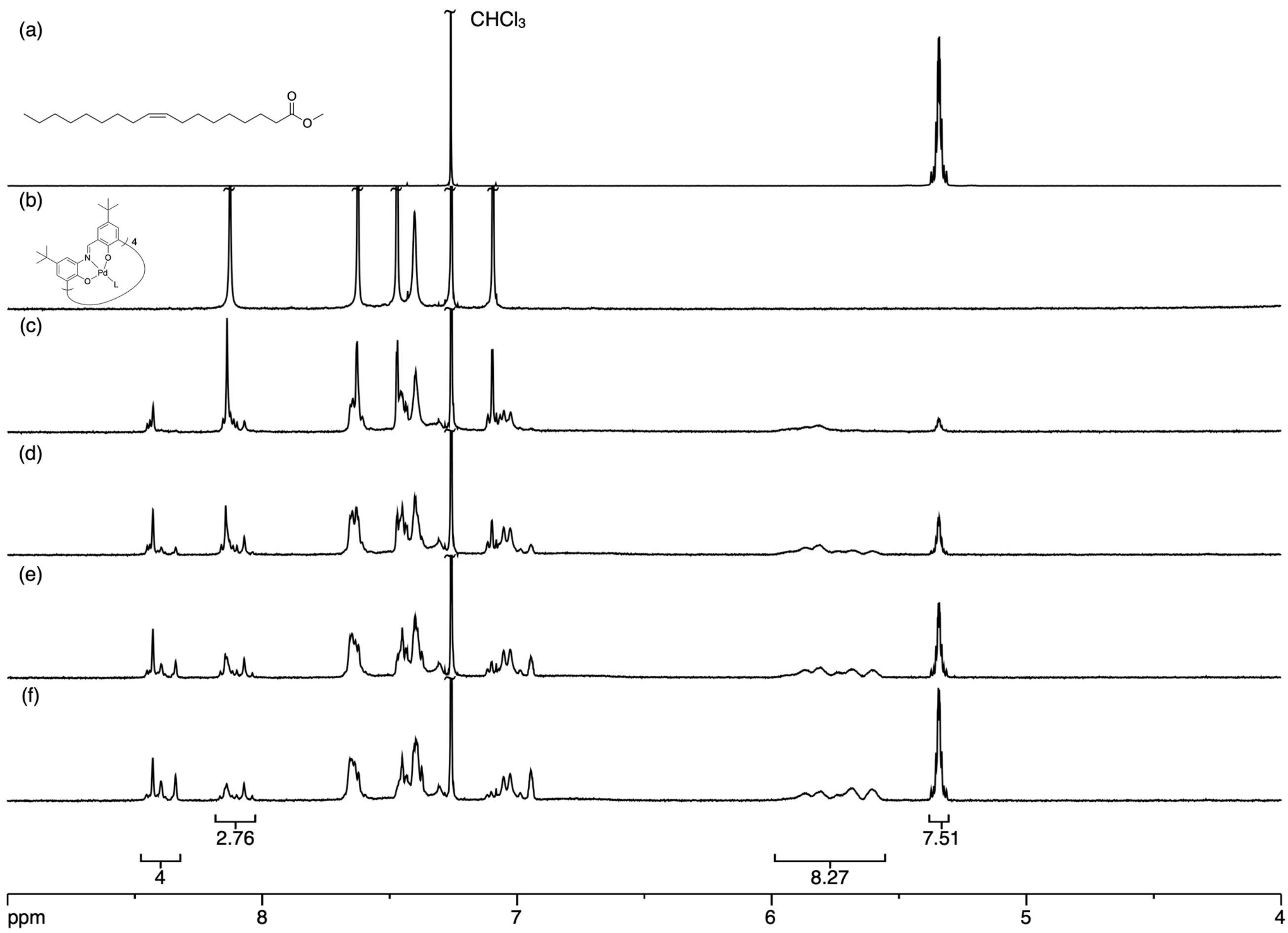

Figure S79. Enlarged ^{1}H NMR spectra of Figure S78.

8-2. Methyl linoleate

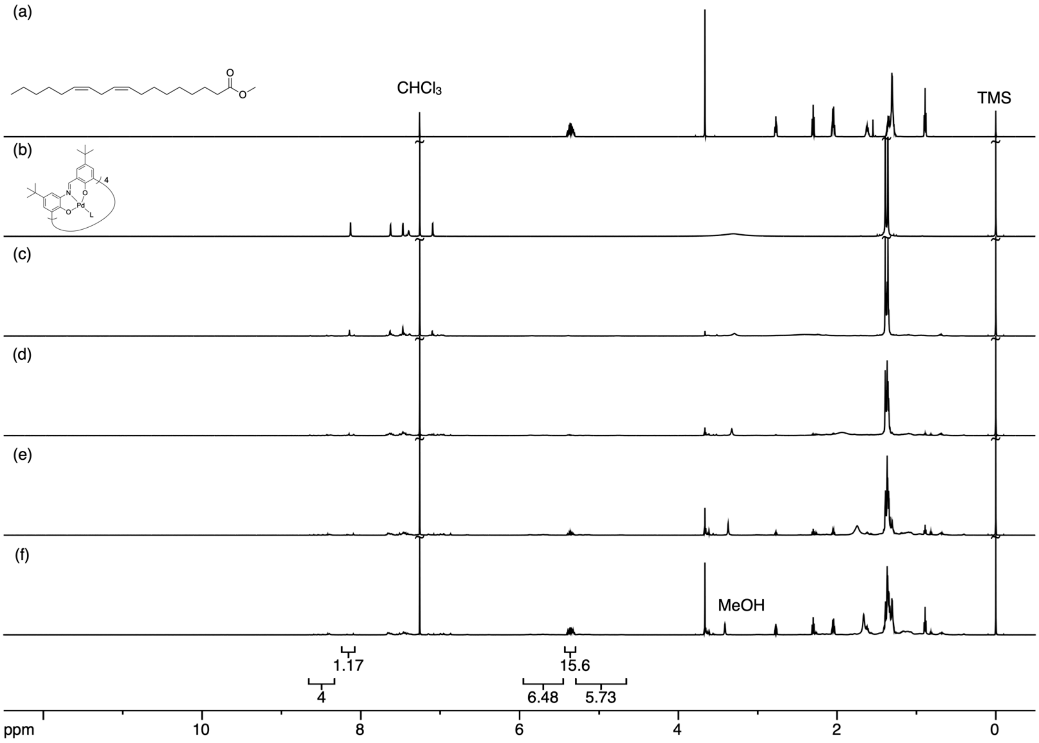

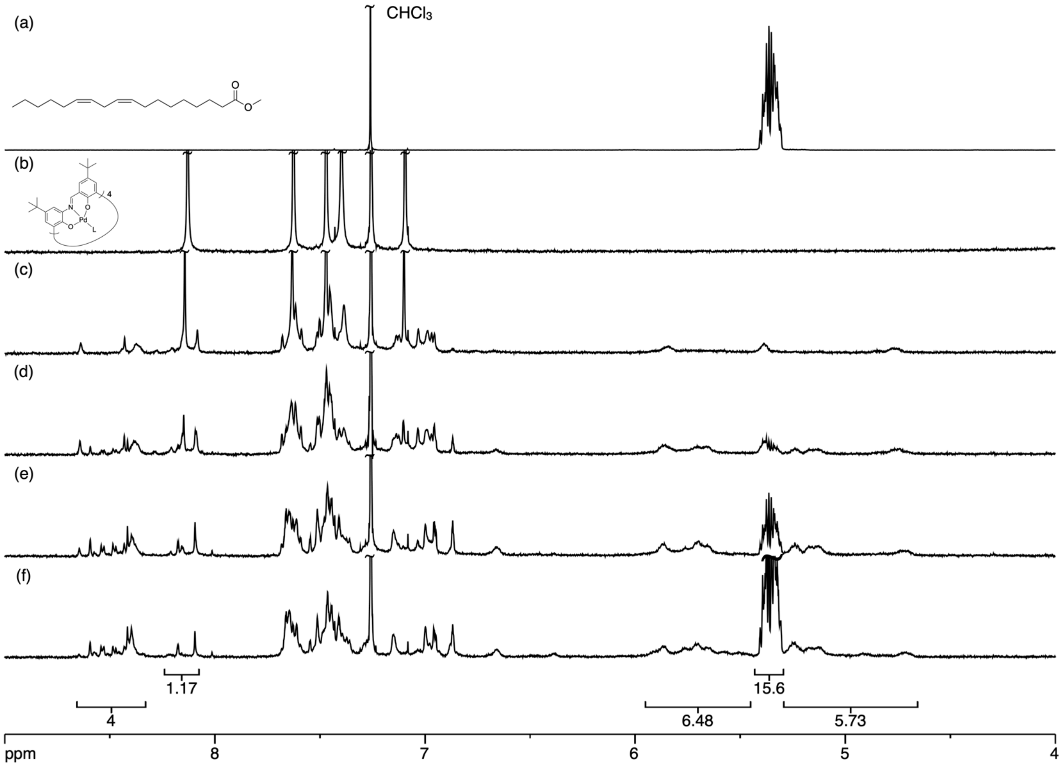

Figure S80. Titration of methyl linoleate to [**C4**Pd_4L_4] in $CDCl_3$ investigated by ^1H NMR measurements. (a–f) ^1H NMR spectra (600 MHz, $CDCl_3$). (a) Methyl linoleate. (b) [**C4**Pd_4L_4]. (c) [**C4**Pd_4L_4] + methyl linoleate 0.56 equiv. (d) [**C4**Pd_4L_4] + methyl linoleate 1.1 equiv. (e) [**C4**Pd_4L_4] + methyl linoleate 2.3 equiv. (f) [**C4**Pd_4L_4] + methyl linoleate 4.5 equiv. $L_4 = (MeOH)_{2.0}(H_2O)_{2.0}$.

8-3. Methyl linolenate

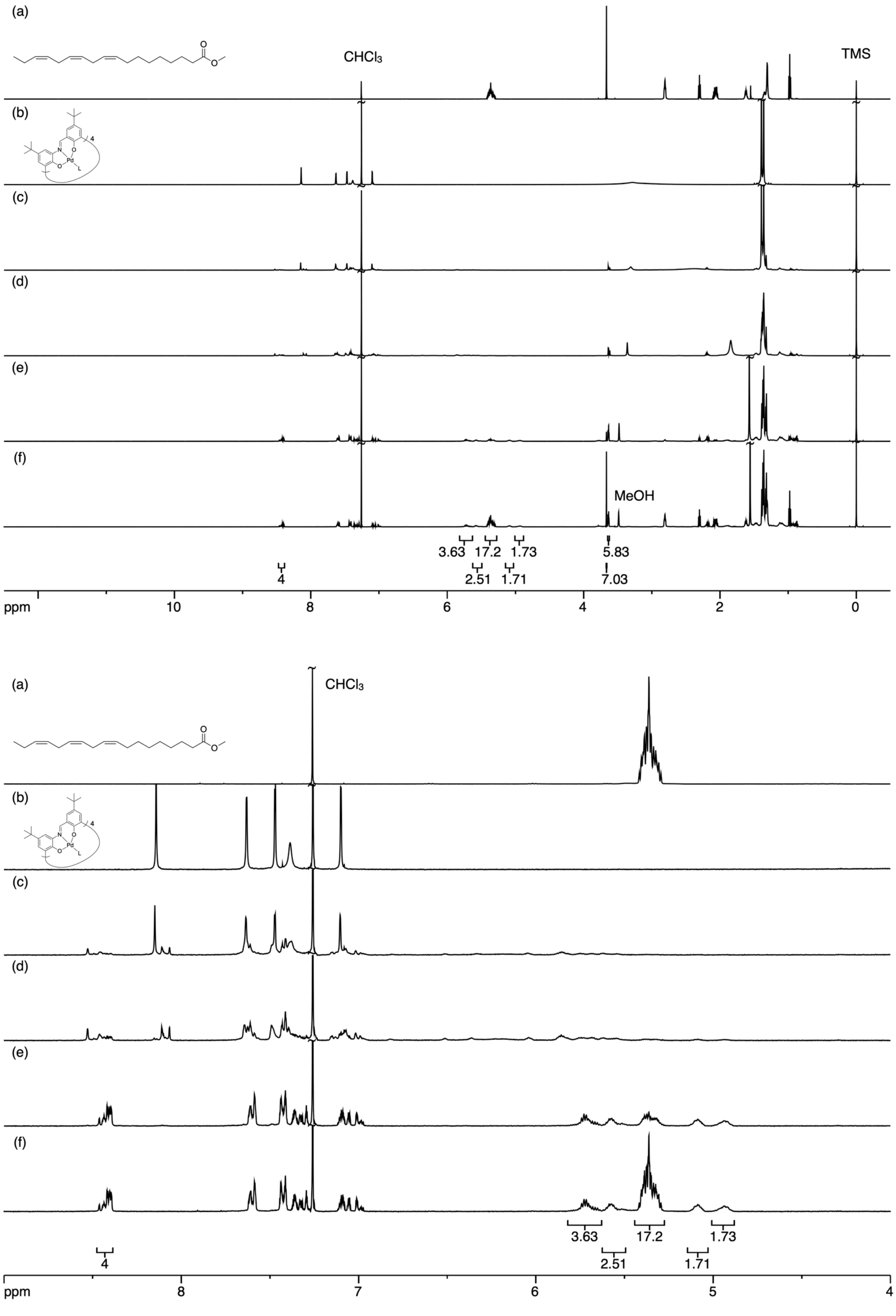

Figure S81. Titration of methyl linolenate to [**C4**Pd_4L_4] in $CDCl_3$ investigated by ^{1}H NMR measurements. (a–f) ^{1}H NMR spectra (600 MHz, $CDCl_3$). (a) Methyl linolenate. (b) [**C4**Pd_4L_4]. (c) [**C4**Pd_4L_4] + methyl linolenate 0.56 equiv. (d) [**C4**Pd_4L_4] + methyl linolenate 1.1 equiv. (e) [**C4**Pd_4L_4] + methyl linolenate 2.3 equiv. (f) [**C4**Pd_4L_4] + methyl linolenate 4.5 equiv. $L_4 = (MeOH)_{2.0}(H_2O)_{2.0}$.

^{1}H NMR assignment of [**C4**Pd_4(methyl linolenate)$_2$]

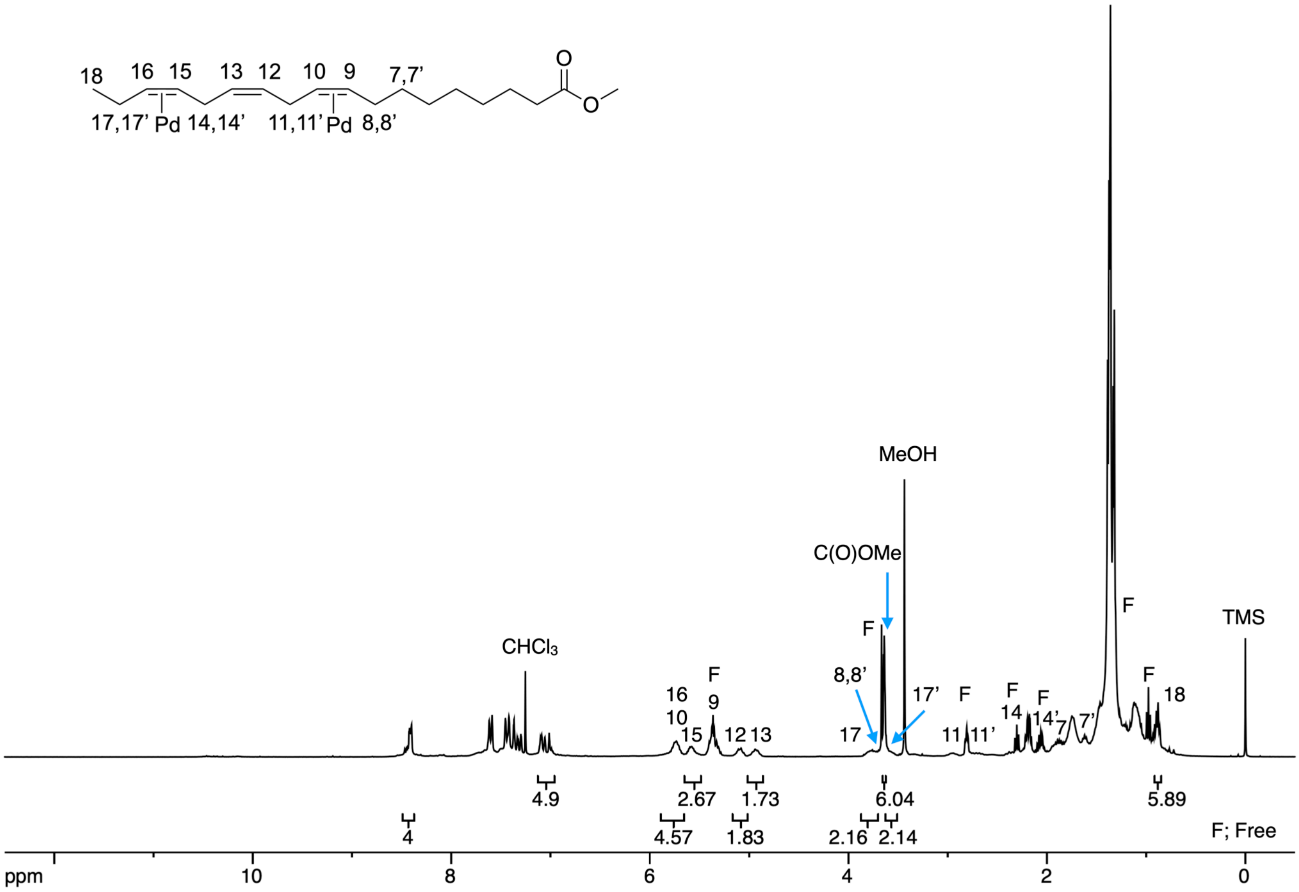

Figure S82. ^{1}H NMR spectrum of [**C4**Pd_4(methyl linolenate)$_2$] ([**C4**Pd_4L_4] (21 mM) + methyl linolenate 2.0 equiv.) (400 MHz, $CDCl_3$). $L_4 = (MeOH)_{2.5}(H_2O)_{1.3}(EtOH)_{0.1}(AcOH)_{0.1}$.

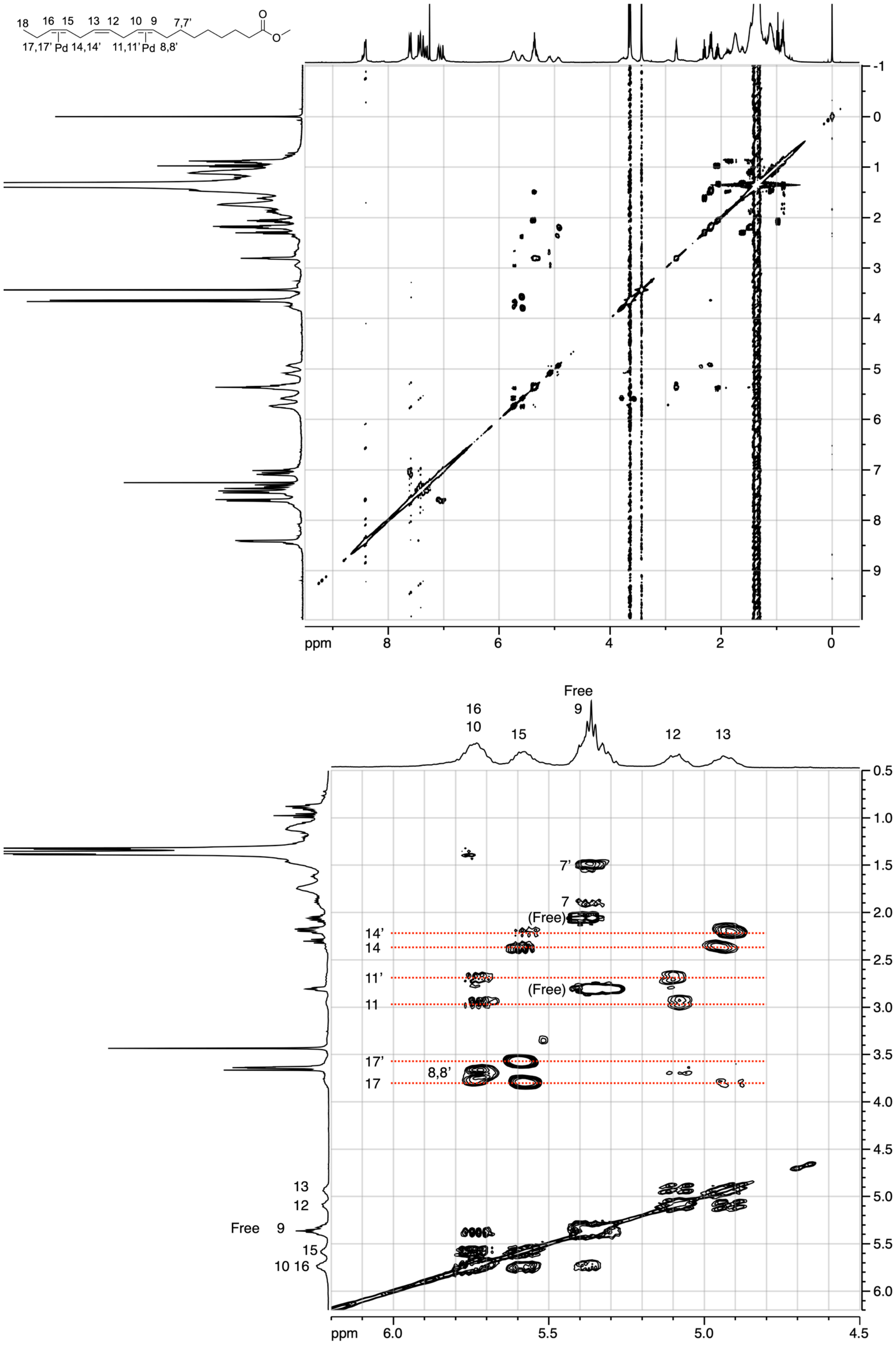

Figure S83. ^{1}H–^{1}H COSY NMR spectrum of [**C4**Pd_4(methyl linolenate)$_2$] (400 MHz, $CDCl_3$).

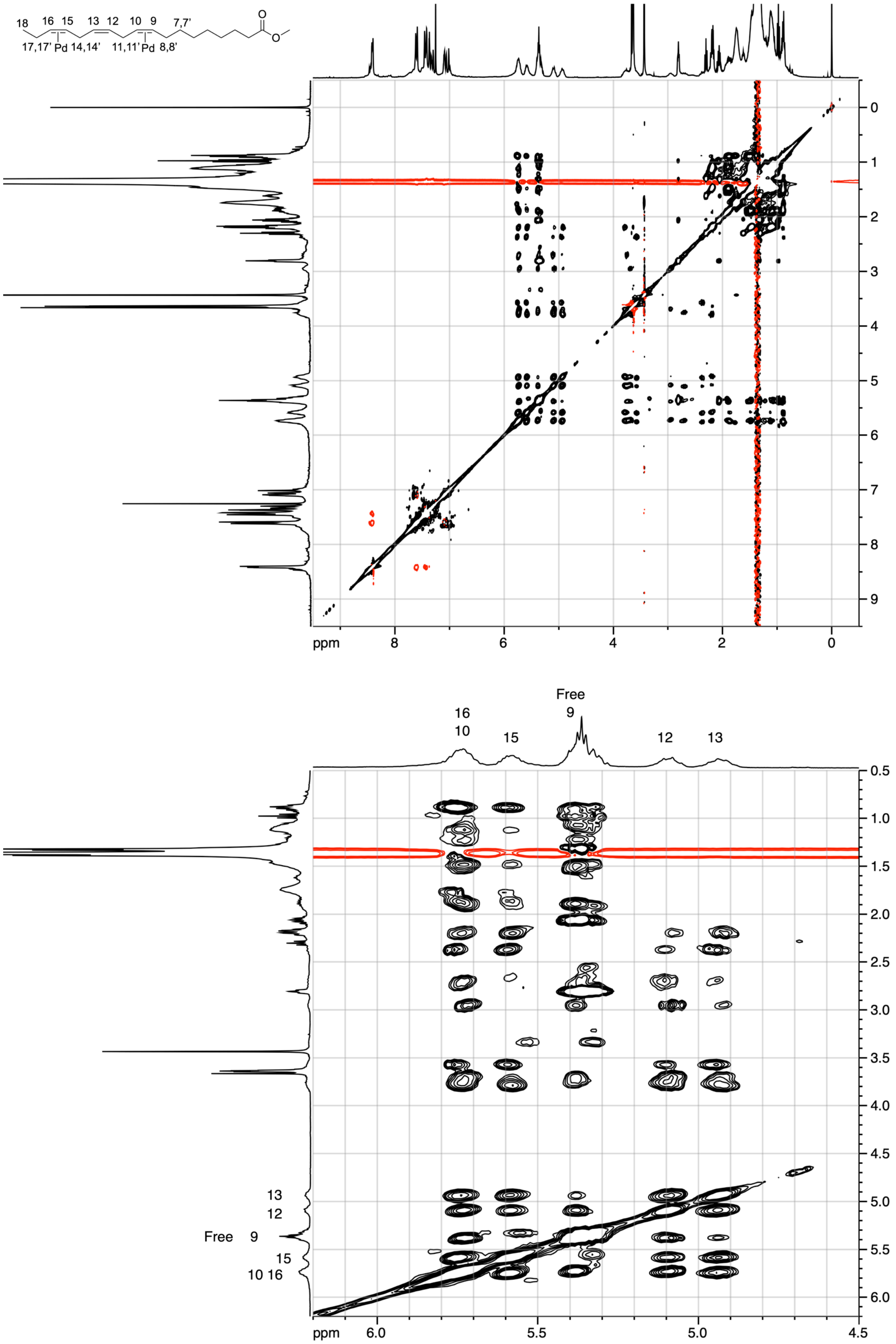

Figure S84. ^{1}H–^{1}H TOCSY NMR spectrum of [**C4**Pd_4(methyl linolenate)$_2$] (400 MHz, $CDCl_3$).

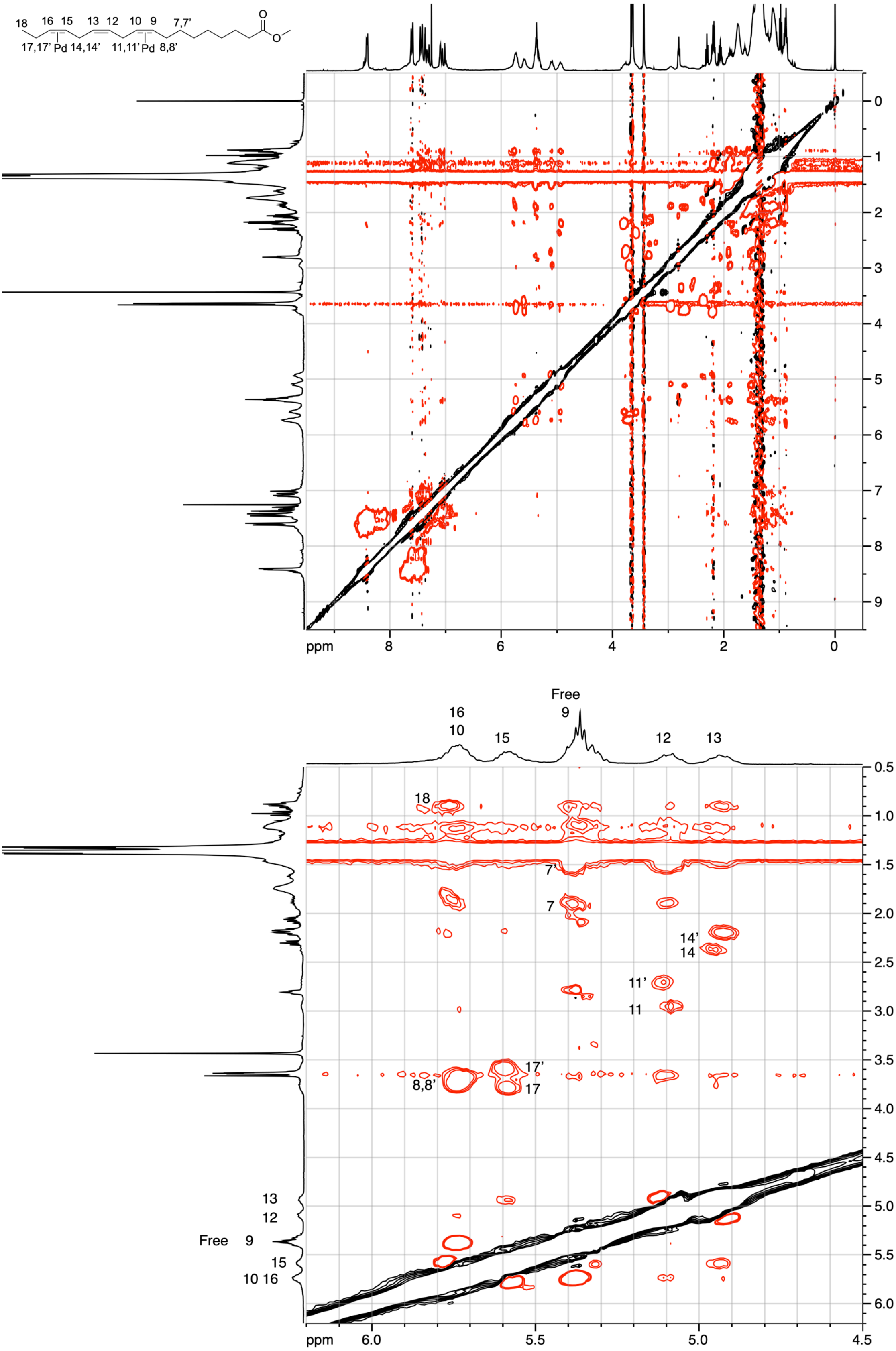

Figure S85. ^{1}H–^{1}H ROESY NMR spectrum of [**C4**Pd$_4$(methyl linolenate)$_2$] (400 MHz, $CDCl_3$).

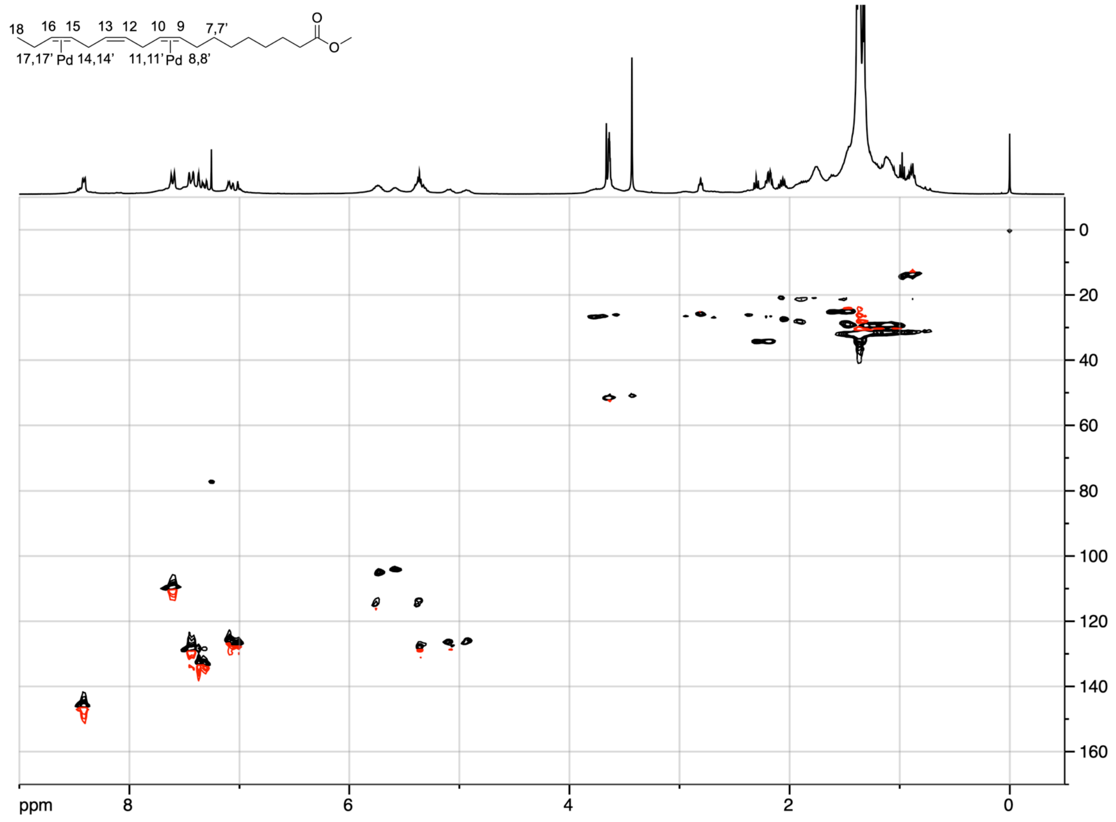

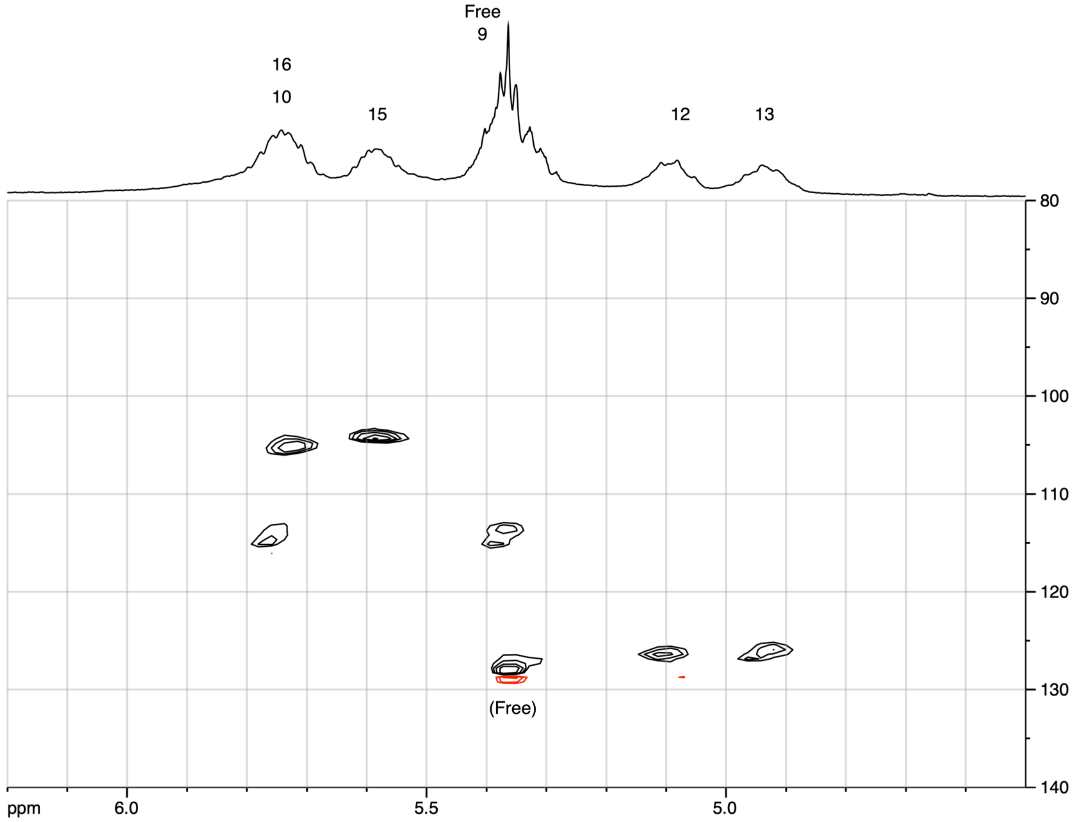

Figure S86. ^{1}H–^{13}C HSQC NMR spectrum of [**C4**Pd_4(methyl linolenate)$_2$] (400 MHz, $CDCl_3$).

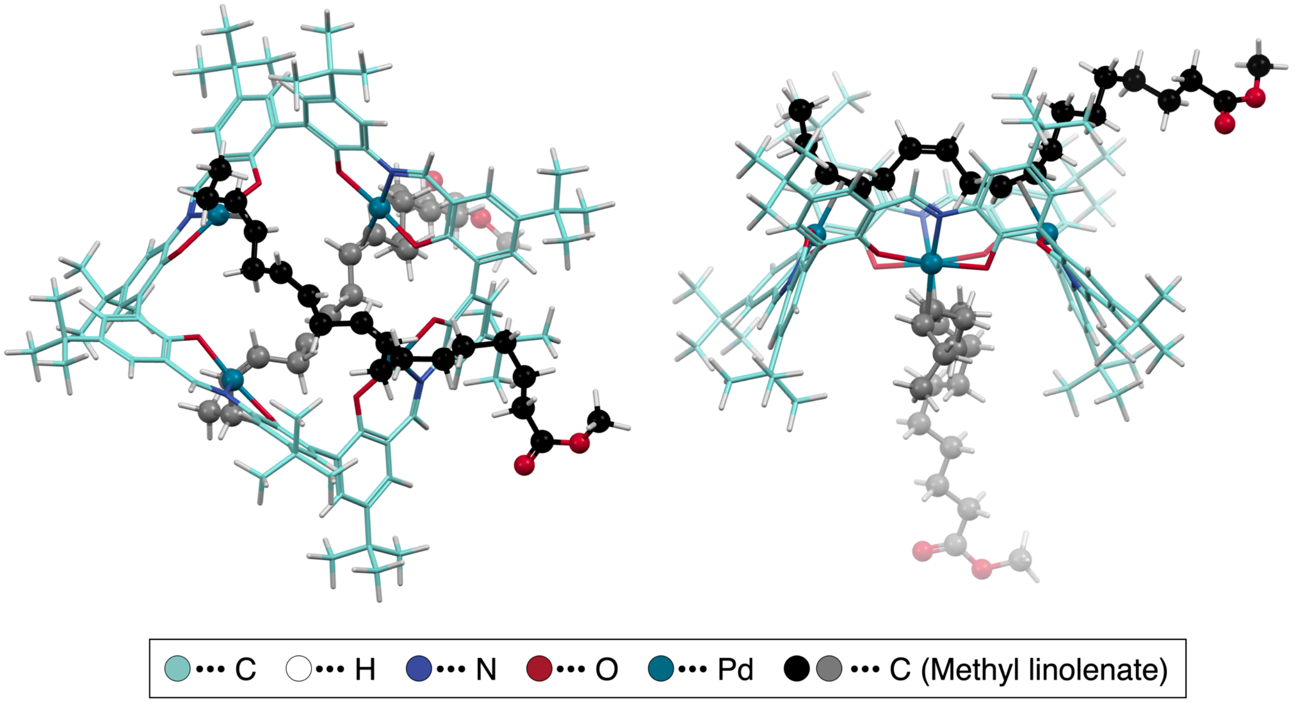

Figure S87. The proposed structure of one isomer of [**C4**Pd_4(methyl linolenate)$_2$].

The structure of [**C4**Pd_4(methyl linolenate)$_2$] shown in Figure S87 was optimized by molecular mechanics calculations (force field: MMFF, calculated by a Spartan'20 software (Wavefunction Inc., *ver* 1.1.2 (2021)).

The integral ratio of the proton signals indicated that two molecules of methyl linolenate bound to Pd-tetrasap. The chemical shift of the olefinic protons of the free molecule appeared at δ 5.46–5.27 ppm. In contrast, the olefinic protons of methyl linolenate in the host-guest complex were observed at 5.83–5.66 (2H), 5.66–5.48 (1H), 5.46–5.27 (1H), 5.16–5.02 (1H), and 5.02–4.86 (1H) ppm. Notably, 4H signals appeared at downfield or similar chemical shift regions, while 2H signals were observed in the upfield region. These olefinic protons were assigned as shown in Figure S82–Figure S86. Based on the discussion of [**C4**Pd_4(amylene)$_4$] and [**C4**Pd_4(squalene)], two C=C double bonds (9,10 and 15,16) coordinated to Pd, while the remaining double bond (12,13) was non-coordinating. Considering the molecular geometry, methyl linolenate coordinates to two palladium centers of the Pd-tetrasap from the same side (Figure S87). [**C4**Pd_4(methyl linolenate)$_2$] is expected to have many possible isomers, resulting from the direction of the fatty acid ester (head, tail) and the coordination mode of the C=C double bonds (*re*-face, *si*-face), and so on. This structural diversity likely explains the observation of many proton signals at similar chemical shifts.

9. Ratio of guest molecules captured by Pd-tetrasap

When olefinic substrates were added to the host solution, the host-guest complex was typically obtained as a mixture of isomers, making it difficult to determine accurate binding constants from ^{1}H NMR measurements. Table S1 summarizes the ratio of guest molecules captured by Pd-tetrasap as a quantitative measure of guest binding strength, calculated using the follow equation.

$$\mathrm{Ratio} = \frac{\text{Integral values of olefinic proton signals of catpured species}}{\text{Integral values of all olefinic proton signals}}$$

Table S1. Ratio of guest molecules captured by Pd-tetrasap (^{1}H NMR, $CDCl_3$).

Guest	Number of captured guest molecules per one host molecule [a]	Guest equivalent	Host concentration / mM	Ratio of captured guest
Squalene	1	1.1	2.1	86% [b]
Amylene	4	4.3	1.4	58%
Geraniol	2–3	2.0	6.1	56%
Farnesol (isomers)	1–2	2.3	2.1	65%
Solanesol	0.5–1	1.0	7.1	51%
Coenzyme Q10	0.5	0.5	2.1	~100%
2,3-Oxidosqualene	1	1.0	1.3	80%
Methyl oleate	2-4	4.5	2.0	52%
Methyl linoleate	2–4	2.3	2.1	65%
Methyl linolenate	2	2.3	2.2	79%

a …The number is estimated from the integral ratio of host/guest proton signals.

b …Average of two runs.

10. Competition experiments of guest molecules

Competition experiments were conducted between squalene and other guests, as well as between methyl linolenate and other unsaturated fatty acid methyl esters. Since both squalene and methyl linolenate were quantitatively captured, the ratios of the complex with squalene/methyl linolenate and that with the competitive guests were calculated based on the corresponding imine signal intensities. The imine signals used for the calculations were not overlapped for the complex of squalene/methyl linolenate and that of the competitive guests. When the imine signal intensity of the competitive guest were below the noise level, the noise intensity was used as the possible maximum intensity.

10-1. Competition of squalene and amylene

As competition experiments of squalene and amylene, the following experiments (1)–(3) were conducted.

(1) Addition of a mixture of squalene and amylene to Pd-tetrasap.

[**C4**$Pd_4(MeOH)_{2.5}(H_2O)_{1.3}(EtOH)_{0.1}(AcOH)_{0.1}$] (3.19 mg, 1.75 µmol, 1.0 equiv.) was added to an NMR tube and dissolved in $CDCl_3$ (450 µL, 3.9 mM) to prepare the host solution. Squalene (9.26 mg, 22.5 µmol) was added to a microtube and dissolved in $CDCl_3$ solution of amylene (32.7 mg/mL (467 mM), 192.5 µL) to prepare the guest solution. Each 15.0 µL (squalene 1.0 equiv. and amylene 4.0 equiv.) aliquot of the guest solution was titrated into the host solution. The ^{1}H NMR measurements (400 MHz, r.t.) were performed during the titration process.

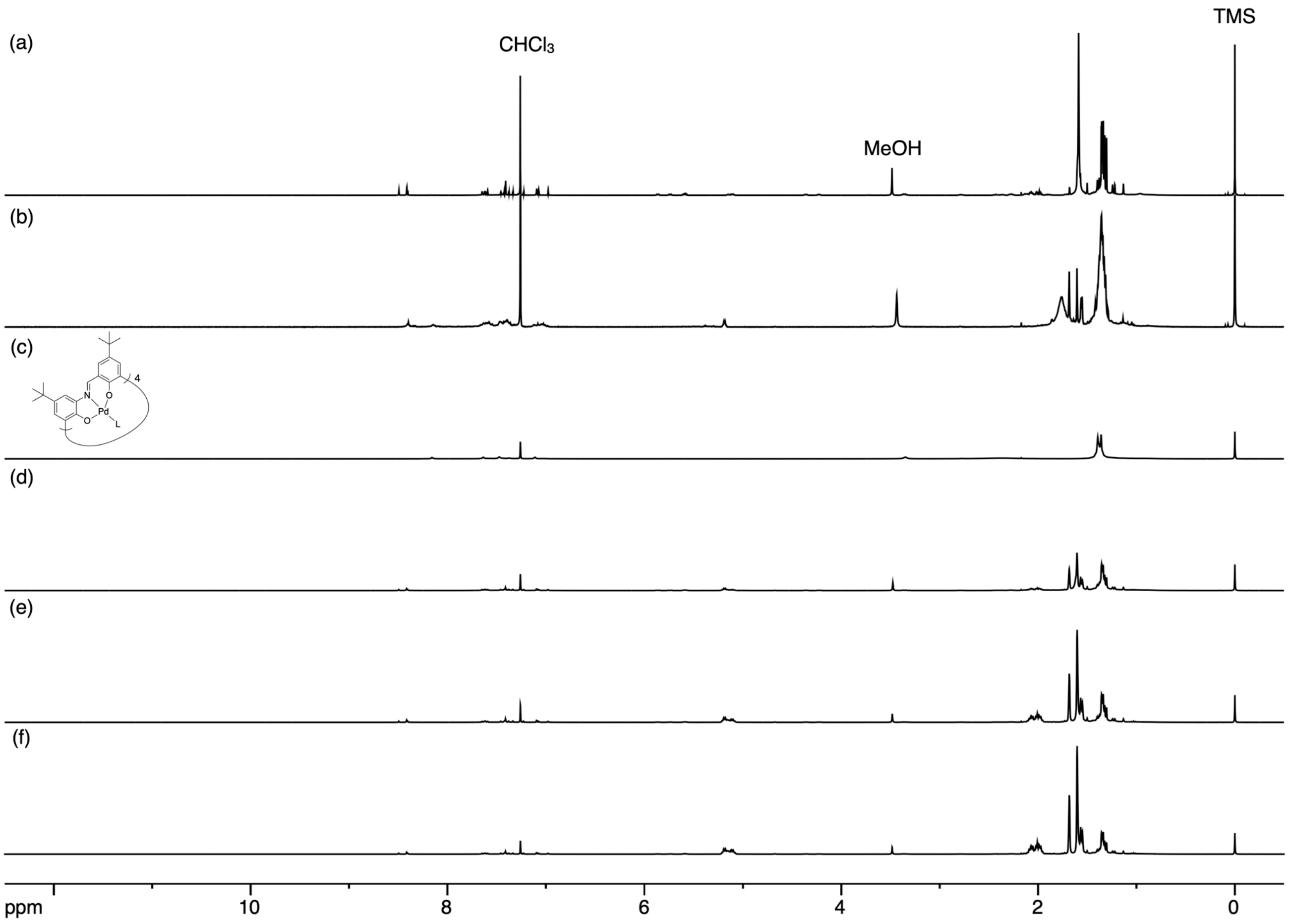

Figure S88. Titration of squalene and amylene to [**C4**Pd_4L_4]. (a,b) ^{1}H NMR spectra (600 MHz, $CDCl_3$). (c–f) ^{1}H NMR spectra (400 MHz, $CDCl_3$). (a) [**C4**Pd_4L_4] + squalene 1.0 equiv. (b) [**C4**Pd_4L_4] + amylene 4.0 equiv. (c) [**C4**Pd_4L_4]. (d) [**C4**Pd_4L_4] + (squalene 1.0 equiv. + amylene 4.0 equiv.). (e) [**C4**Pd_4L_4] + (squalene 2.0 equiv. + amylene 8.0 equiv.). (f) [**C4**Pd_4L_4] + (squalene 3.0 equiv. + amylene 12 equiv.). L_4 = $(MeOH)_{2.5}(H_2O)_{1.3}(EtOH)_{0.1}(AcOH)_{0.1}$.

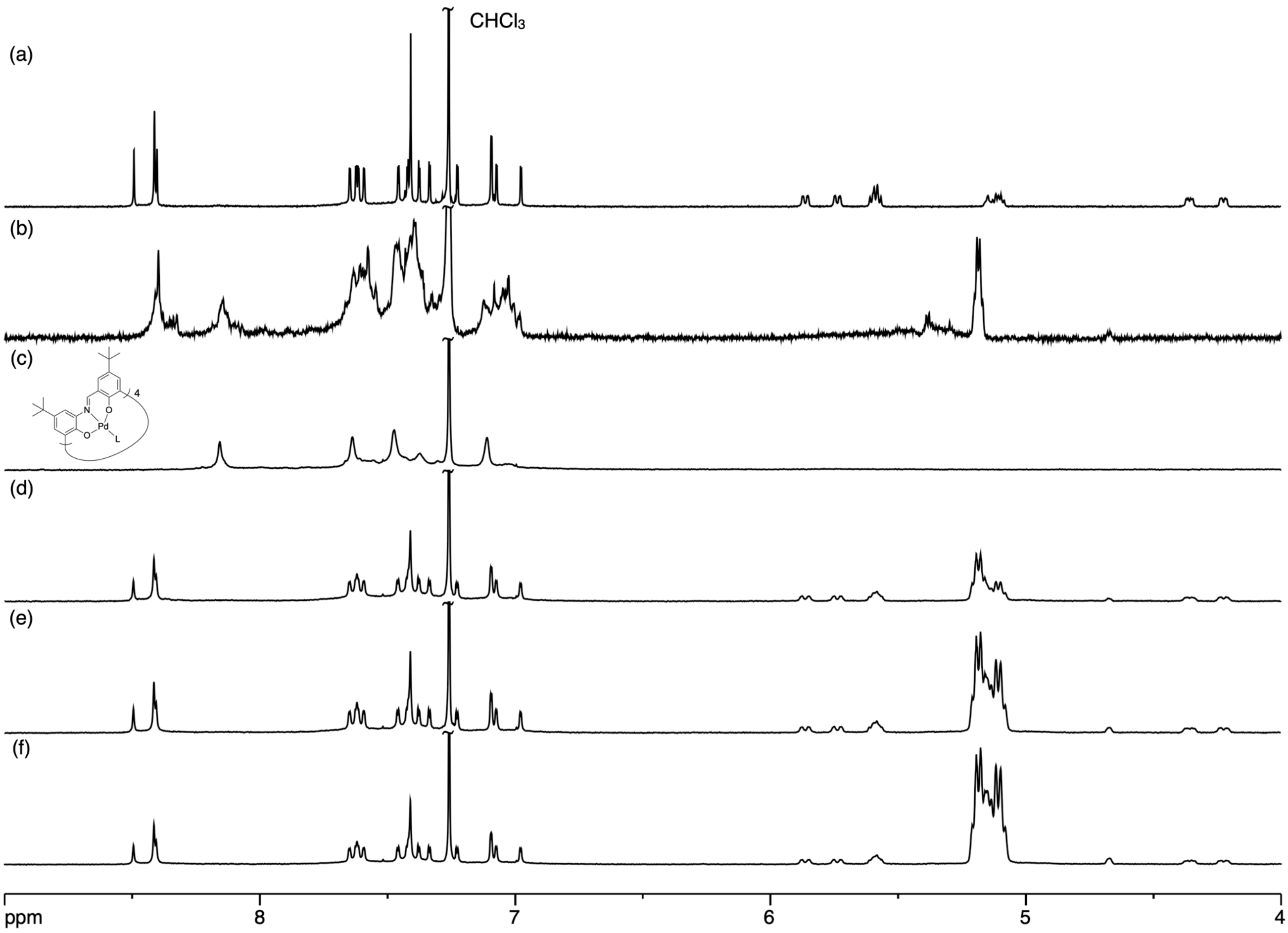

Figure S89. Enlarged ^{1}H NMR spectra of Figure S88.

Table S2. Ratio of [**C4**Pd_4(amylene)$_4$] in the section 10-1 (1) experiment.

Entry	Condition		Ratio of	
	Squalene	Amylene	[**C4**Pd_4(amylene)$_4$]	$\frac{[\mathbf{C4}\mathrm{Pd}_4(\mathrm{squalene})]}{[\mathbf{C4}\mathrm{Pd}_4(\mathrm{amylene})_4]}$
(d)	1.0 equiv.	4.0 equiv.	<13%	>6
(e)	2.0 equiv.	8.0 equiv.	<9%	>9
(f)	3.0 equiv.	12 equiv.	<7%	>13

(2) Addition of amylene after the addition of squalene to Pd-tetrasap.

[**C4**$Pd_4(MeOH)_{2.5}(H_2O)_{1.3}(EtOH)_{0.1}(AcOH)_{0.1}$] (2.20 mg, 1.21 μmol, 1.0 equiv.) was dissolved in $CDCl_3$ (400 μL) in an NMR tube, then $CDCl_3$ solution of squalene (4.95 mg/mL (12.0 mM), 100 μL, 1.0 equiv.) was added. Amylene (5.05 mg, 72.0 μmol) was added to a microtube and dissolved in $CDCl_3$ (150 μL) to prepare the guest solution. Aliquots of 10.0 μL (amylene 4.0 equiv.), 30.0 μL, and 50.0 μL of the guest solution were titrated into the host-squalene solution. The ^{1}H NMR measurements (400 MHz, r.t.) were conducted during the titration process.

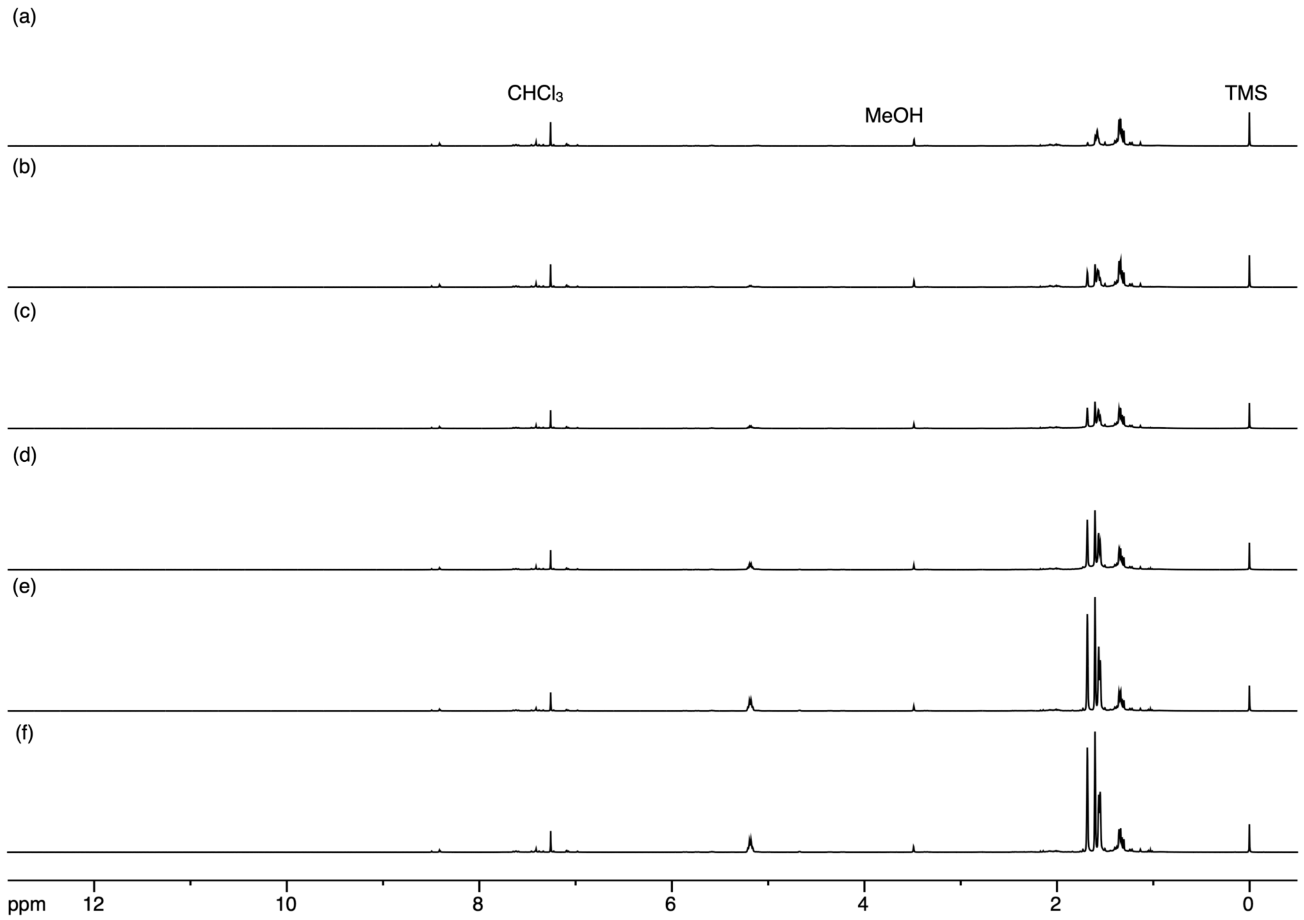

Figure S90. Titration of amylene to [**C4**Pd_4L_4] + squalene 1.0 equiv. (a–f) ^{1}H NMR spectra (400 MHz, $CDCl_3$). (a) [**C4**Pd_4L_4] + squalene 1.0 equiv. (b) ([**C4**Pd_4L_4] + squalene 1.0 equiv.) + amylene 4.0 equiv. (c) ([**C4**Pd_4L_4] + squalene 1.0 equiv.) + amylene 8.0 equiv. (d) ([**C4**Pd_4L_4] + squalene 1.0 equiv.) + amylene 20 equiv. (e) ([**C4**Pd_4L_4] + squalene 1.0 equiv.) + amylene 40 equiv. (f) The sample (e) left to stand at r.t. for 12 d. L_4 = $(MeOH)_{2.5}(H_2O)_{1.3}(EtOH)_{0.1}(AcOH)_{0.1}$.

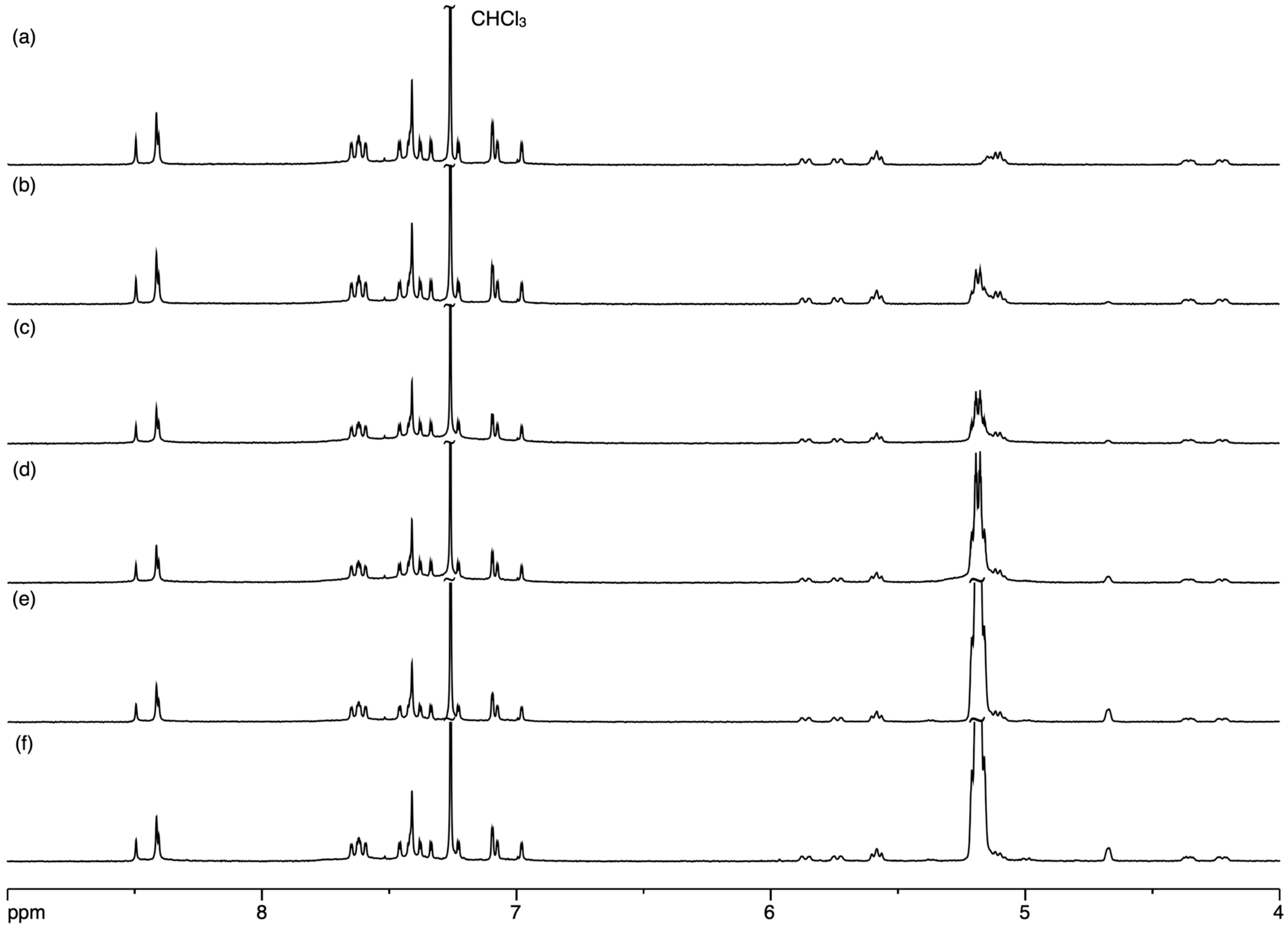

Figure S91. Enlarged ^{1}H NMR spectra of Figure S90.

Table S3. Ratio of [**C4**Pd_4(amylene)$_4$] in the section 10-1 (2) experiment.

Entry	Condition		Ratio of	$\frac{[\mathbf{C4}Pd_4(\text{squalene})]}{[\mathbf{C4}Pd_4(\text{amylene})_4]}$
	Squalene	Amylene	[**C4**Pd_4(amylene)$_4$]	
(b)	1.0 equiv.	4.0 equiv.	<10%	>9
(c)		8.0 equiv.	<12%	>7
(d)		20 equiv.	<12%	>7
(e)		40 equiv.	<12%	>7
(f)	r.t., 12 d		<13%	>6

(3) Addition of squalene after addition of amylene to Pd-tetrasap.

[**C4**$Pd_4(MeOH)_{2.0}(H_2O)_{2.0}$]•$13H_2O$ (1.84 mg, 0.899 μmol, 1.0 equiv.) was dissolved in $CDCl_3$ (450 μL) in an NMR tube, then $CDCl_3$ solution of amylene (56.90 mg/mL (0.811 M), 50 μL, 45 equiv.) was added. Squalene (3.42 mg, 8.33 μmol) was added to a microtube and dissolved in $CDCl_3$ (410 μL) to prepare the guest solution. 50 μL aliquot of the guest solution (squalene 1.02 μmol, 1.1 equiv.) was added to the host-amylene solution. The ^1H NMR measurements (600 MHz, r.t.) were conducted during the titration.

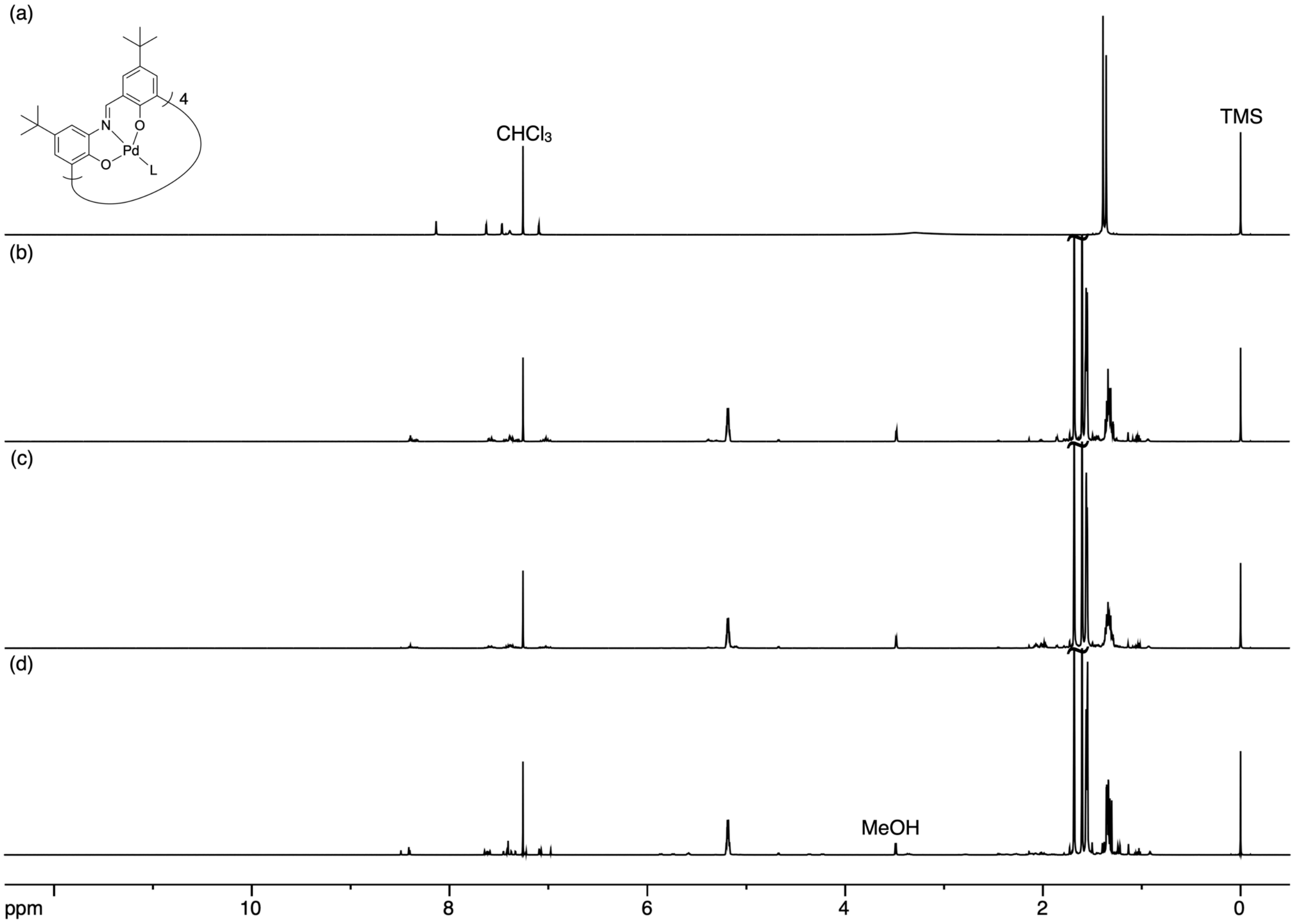

Figure S92. Addition of squalene to [**C4**Pd_4L_4] + amylene 45 equiv. (a–d) ^1H NMR spectra (600 MHz, $CDCl_3$). (a) [**C4**Pd_4L_4]. (b) [**C4**Pd_4L_4] + amylene 45 equiv. (c) ([**C4**Pd_4L_4] + amylene 45 equiv.) + squalene 1.1 equiv. (d) The sample (c) left to stand at r.t. for 9 h. $L_4 = (MeOH)_{2.0}(H_2O)_{2.0}$.

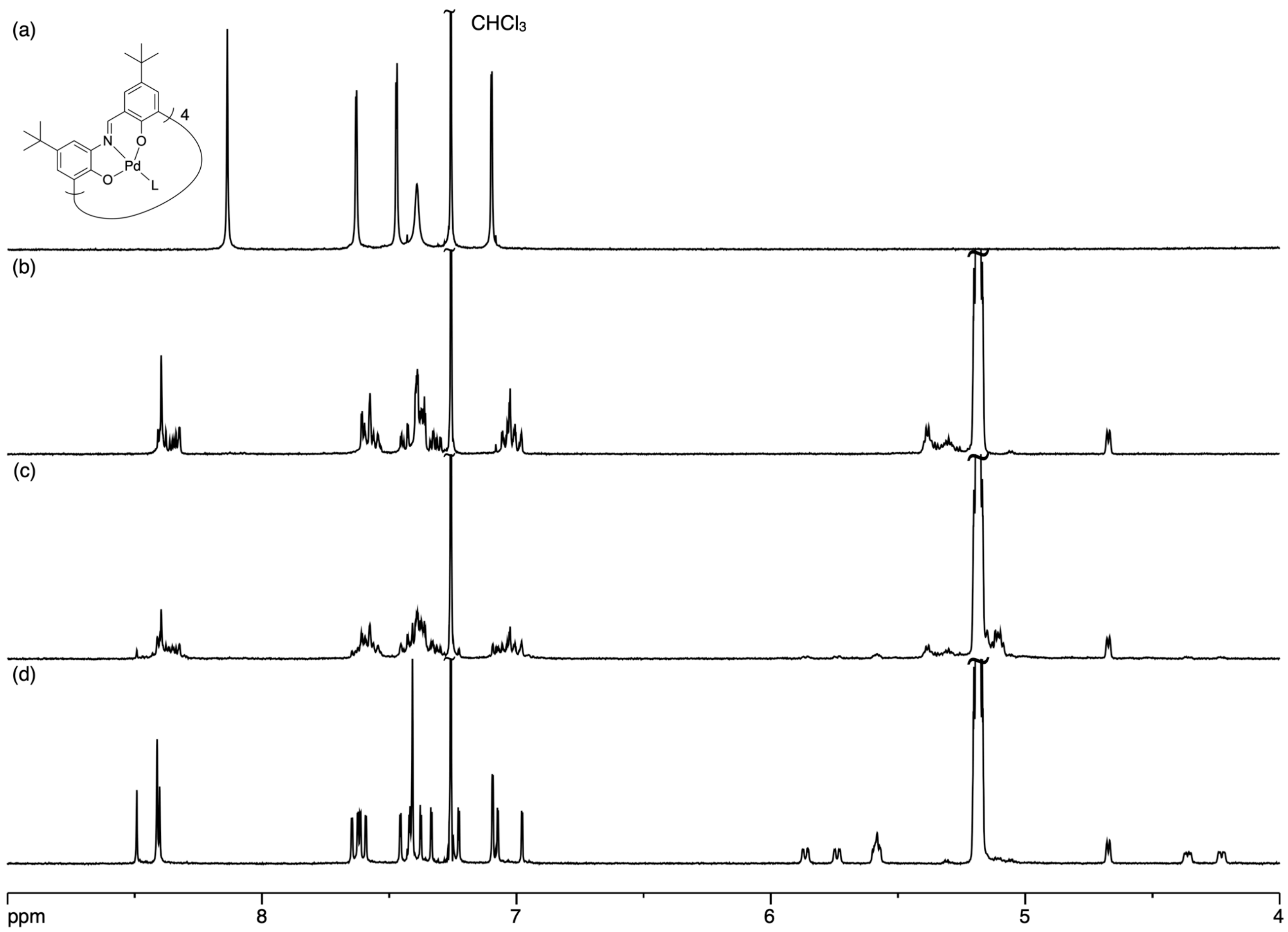

Figure S93. Enlarged ^{1}H NMR spectra of Figure S92.

Table S4. Ratio of [**C4**Pd_4(amylene)$_4$] in the section 10-1 (3) experiment.

Entry	Condition		Ratio of	$\frac{[\mathbf{C4}\mathrm{Pd}_4(\mathrm{squalene})]}{[\mathbf{C4}\mathrm{Pd}_4(\mathrm{amylene})_4]}$
	Squalene	Amylene	[**C4**Pd_4(amylene)$_4$]	
(c)	1.1 equiv.	45 equiv.	>81%	<0.3
(d)	r.t., 9 h		<9%	>11

The ligand exchange from amylene to squalene did not occur immediately (approximately 10 min. was not sufficient). However, the equilibrium was achieved after at least 9 h at r.t.

10-2. Competition of squalene and geraniol or solanesol

^{1}H NMR competition experiments of squalene and geraniol or solanesol were conducted in $CDCl_3$ solution in a similar manner to section 10-1 (2).

Competition of squalene and geraniol

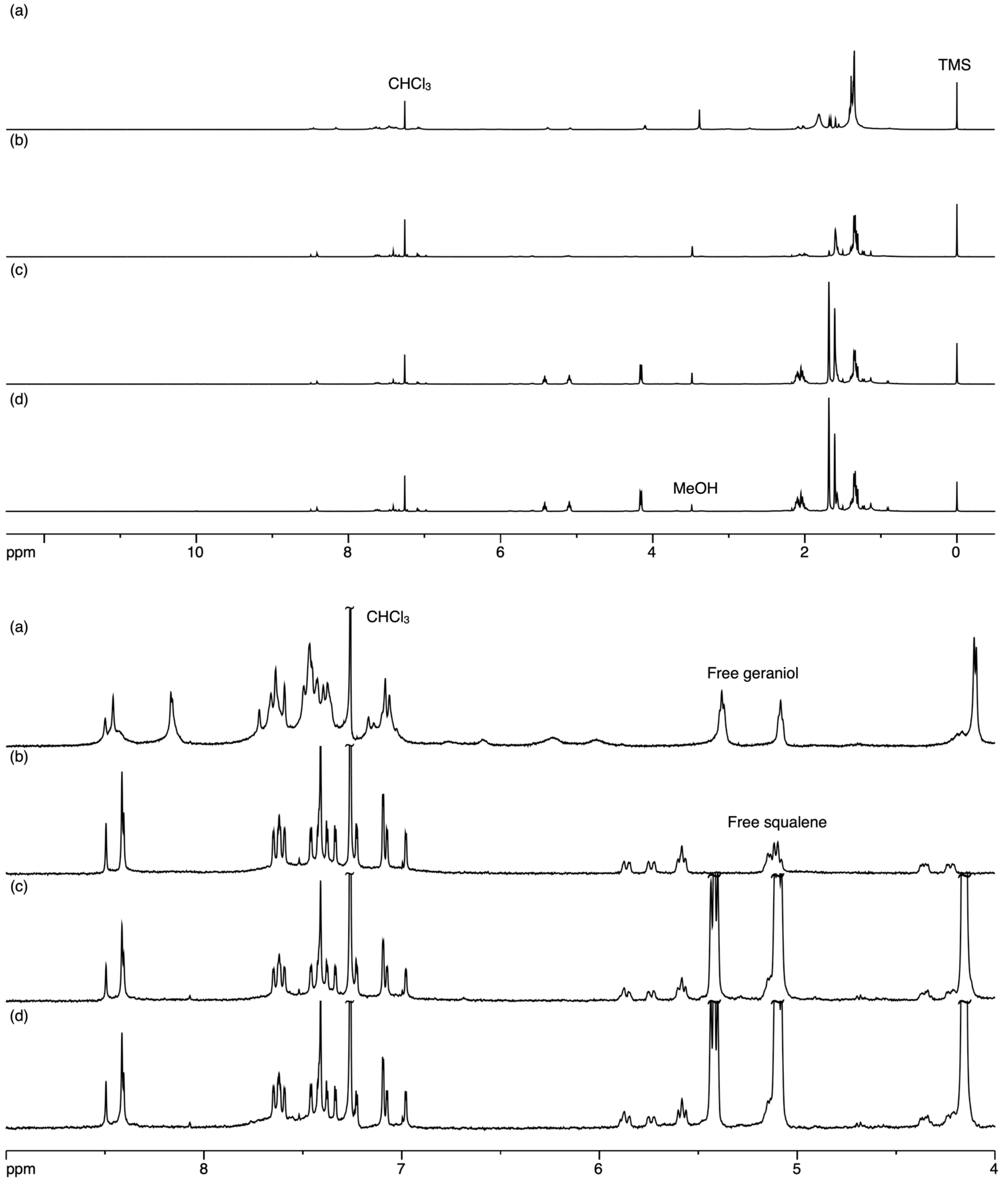

Figure S94. Competition experiment of squalene and geraniol. (a) ^{1}H NMR spectrum (600 MHz, $CDCl_3$). (b–d) ^{1}H NMR spectra (400 MHz, $CDCl_3$). (a) [**C4**Pd_4L_4] + geraniol 2.0 equiv. (b) [**C4**Pd_4L_4] + squalene 1.0 equiv. (c) ([**C4**Pd_4L_4] + squalene 1.0 equiv.) + geraniol 10 equiv. (d) The sample (c) left to stand at r.t. for 12 d. L_4 = $(MeOH)_{2.5}(H_2O)_{1.3}(EtOH)_{0.1}(AcOH)_{0.1}$.

Competition of squalene and solanesol

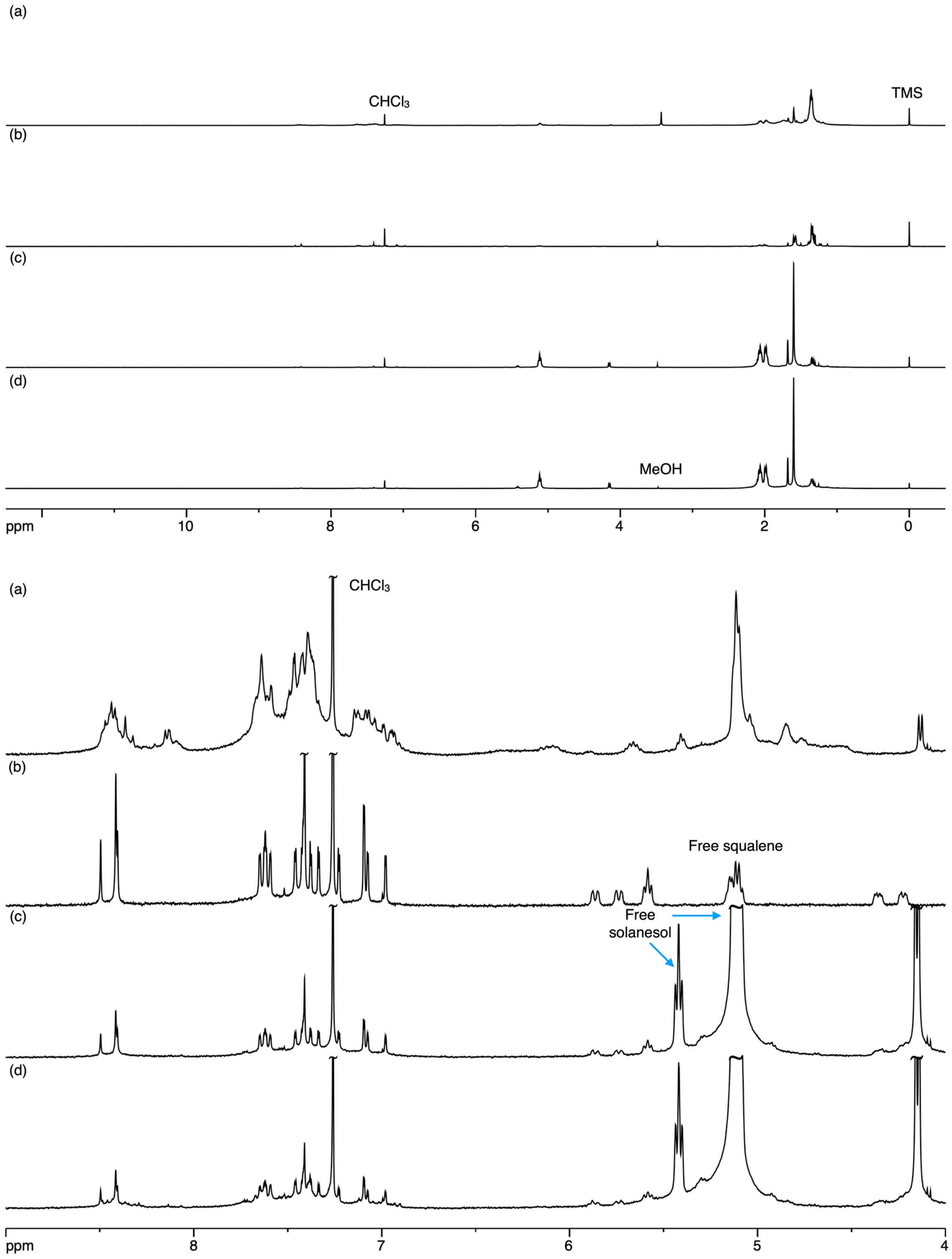

Figure S95. Competition experiment of squalene and solanesol. (a) ^{1}H NMR spectrum (600 MHz, $CDCl_3$). (b–d) ^{1}H NMR spectra (400 MHz, $CDCl_3$). (a) [**C4**Pd_4L_4] + solanesol 1.0 equiv. (b) [**C4**Pd_4L_4] + squalene 1.0 equiv. (c) ([**C4**Pd_4L_4] + squalene 1.0 equiv.) + solanesol 10 equiv. (d) The sample (c) left to stand at r.t. for 12 d. L_4 = $(MeOH)_{2.5}(H_2O)_{1.3}(EtOH)_{0.1}(AcOH)_{0.1}$.

Table S5. Ratio of [**C4**Pd$_4$(competitive guest)$_n$] for the experiments in the section 10-1(2) and 10-2.

Competitive Guest	Condition[a]	Ratio of [**C4**Pd$_4$(guest)$_n$]	$\frac{[\mathbf{C4}\mathrm{Pd}_4(\mathrm{squalene})]}{[\mathbf{C4}\mathrm{Pd}_4(\mathrm{guest})_n]}$
Amylene	40 equiv.	<13%	>6
Geraniol	10 equiv.	<4%	>22
Solanesol	10 equiv.	<22%	>3

a … Pd-tetrasap (2.0 mM), squalene 1.0 equiv., stood at r.t. for 12 d.

10-3. Competition of squalene and methanol

[**C4**$Pd_4(MeOH)_{2.5}(H_2O)_{1.3}(EtOH)_{0.1}(AcOH)_{0.1}$] (2.20 mg, 1.21 μmol, 1.0 equiv.) was dissolved in $CDCl_3$ (400 μL) in an NMR tube, then $CDCl_3$ solution of squalene (4.95 mg/mL (12.0 mM), 100 μL, 1.0 equiv.) was added. CD_3OD was added to the host-guest solution. The ^1H NMR measurements (400 MHz, r.t.) were conducted during the titration process.

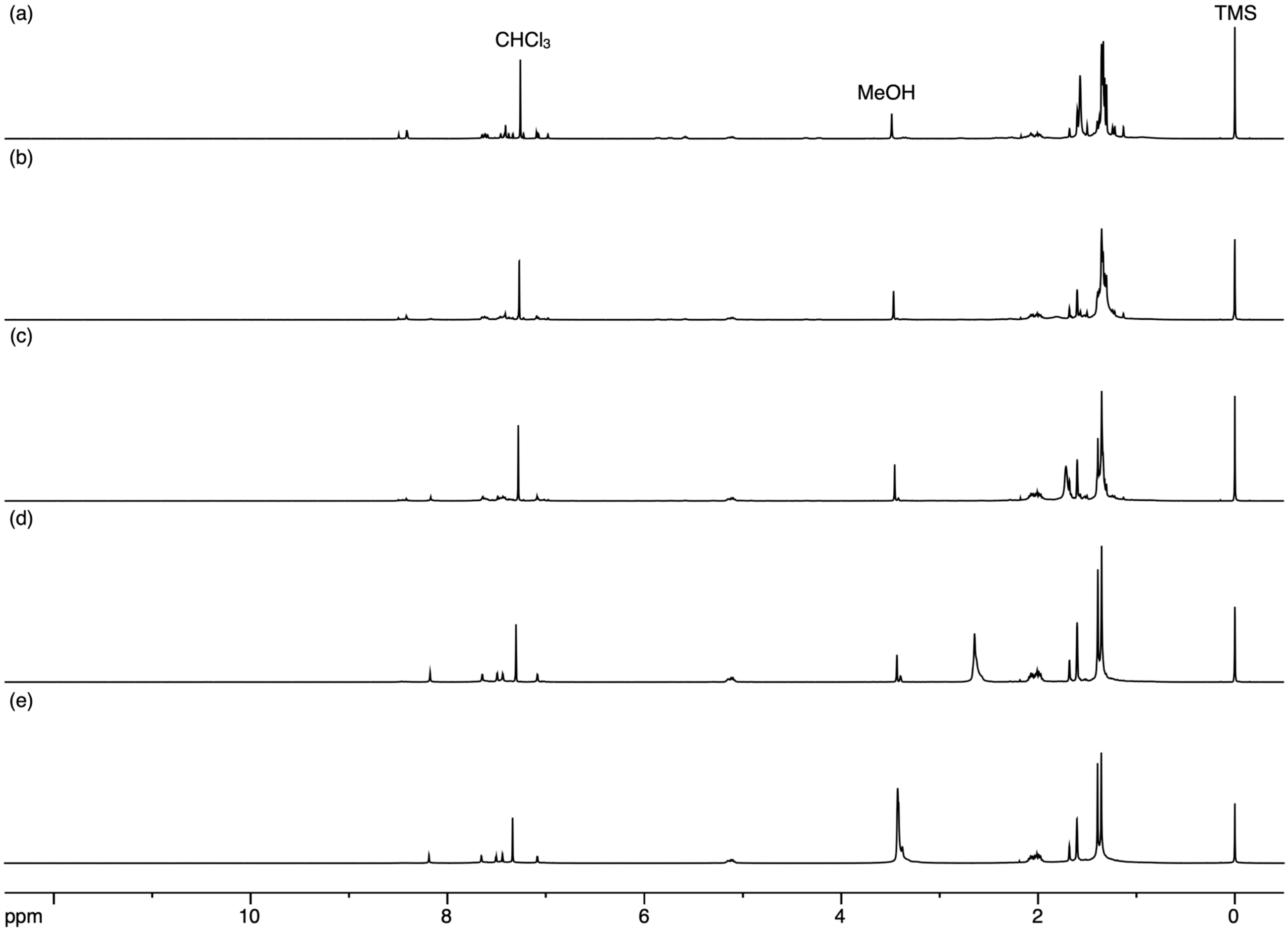

Figure S96. Addition of CD_3OD to [**C4**Pd_4L_4] + squalene 1.0 equiv. in $CDCl_3$. (a–e) ^1H NMR spectra (400 MHz). $CDCl_3/CD_3OD$ (v/v) = (a) 100/0. (b) 100/1 (CD_3OD: 100 equiv. [/squalene]). (c) 50/1. (d) 20/1. (e) 10/1. $L_4 = (MeOH)_{2.5}(H_2O)_{1.3}(EtOH)_{0.1}(AcOH)_{0.1}$.

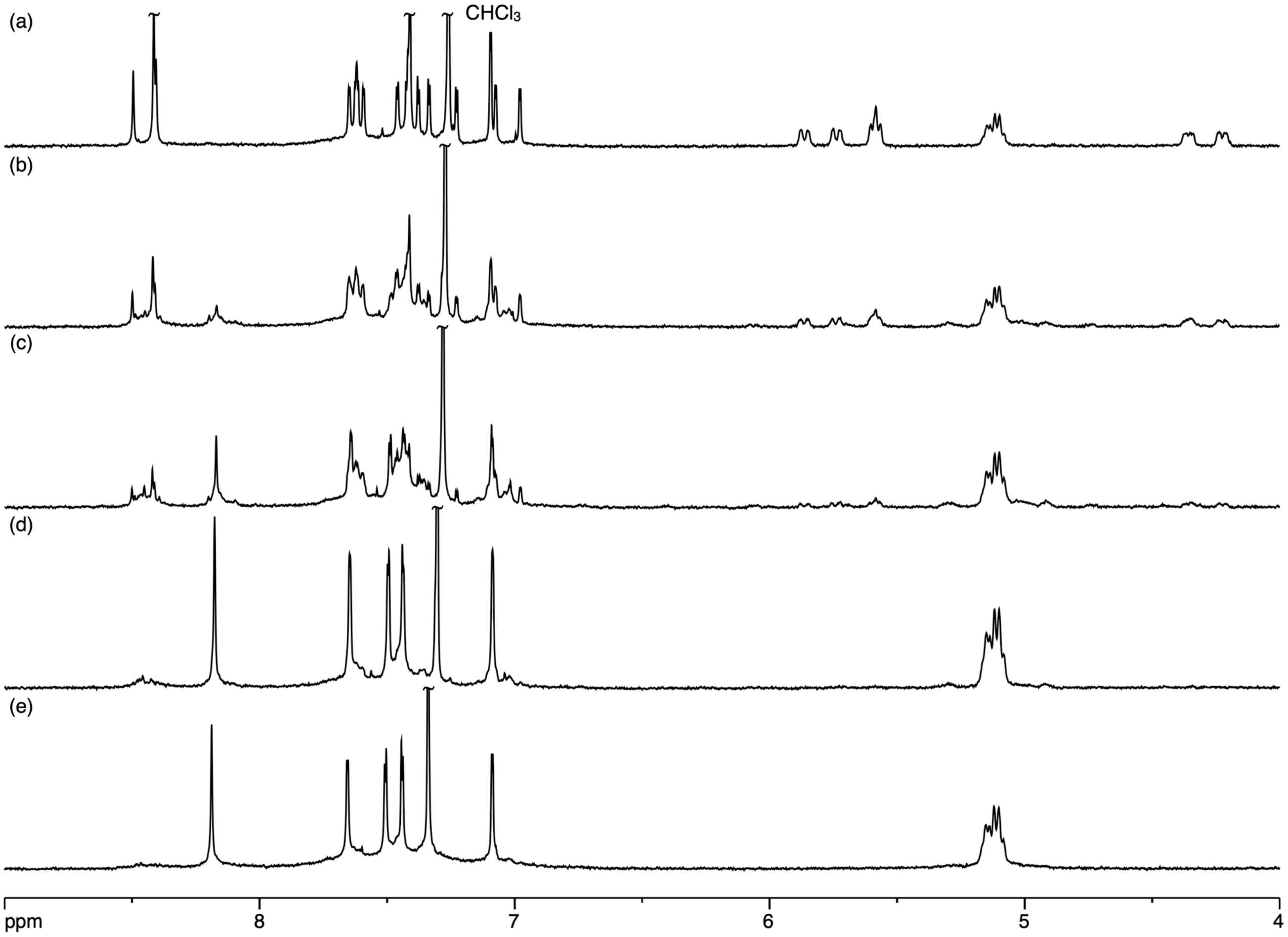

Figure S97. Enlarged ^{1}H NMR spectra of Figure S96.

Table S6. Ratio of [**C4**Pd_4(squalene)].

Entry	Condition $CDCl_3/CD_3OD$ (v/v)	Ratio of [**C4**Pd_4(squalene)]
(a)	100/0	68%
(b)	100/1	70%
(c)	50/1	52%
(d)	20/1	28%
(e)	10/1	21%

The ratio of [**C4**Pd_4(squalene)] was determined from the integral values of (a) the olefin protons, (b–e) the imine protons.

10-4. Competition of methyl linolenate and methyl oleate or methyl linoleate

^{1}H NMR competition experiments of unsaturated fatty acid methyl esters were conducted in $CDCl_3$ solution in a similar manner to section 10-1 (1).

Competition of methyl linolenate and methyl oleate

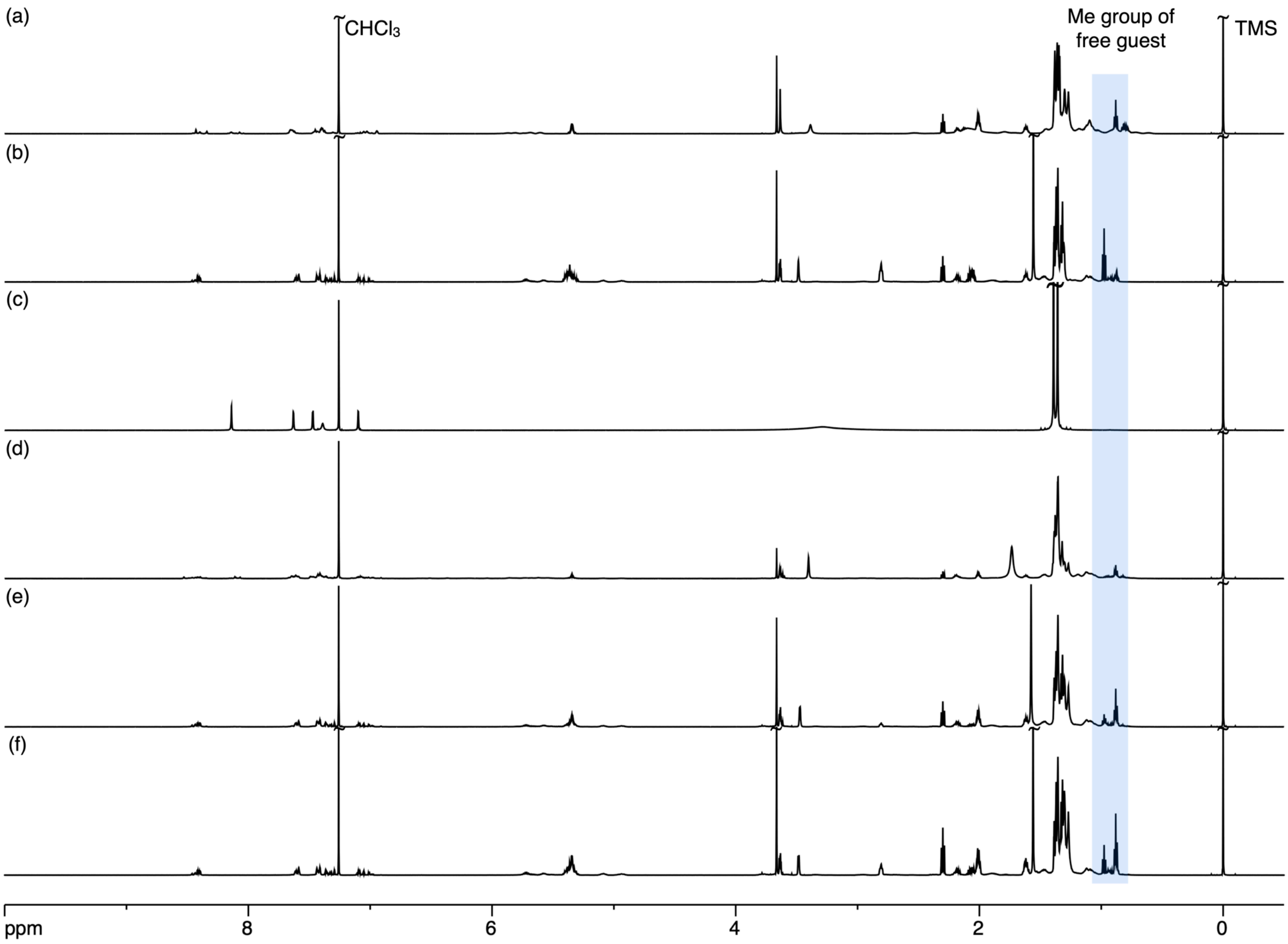

Figure S98. Titration of methyl linolenate and methyl oleate to [**C4**Pd_4L_4]. (a–f) ^{1}H NMR spectra (600 MHz, $CDCl_3$). (a) [**C4**Pd_4L_4] + methyl oleate 4.5 equiv. (b) [**C4**Pd_4L_4] + methyl linolenate 4.5 equiv. (c) [**C4**Pd_4L_4]. (d) [**C4**Pd_4L_4] + (methyl linolenate 1.1 equiv. + methyl oleate 1.1 equiv.). (e) [**C4**Pd_4L_4] + (methyl linolenate 2.3 equiv. + methyl oleate 2.3 equiv.). (f) [**C4**Pd_4L_4] + (methyl linolenate 3.4 equiv. + methyl oleate 3.4 equiv.). $L_4 = (MeOH)_{2.0}(H_2O)_{2.0}$.

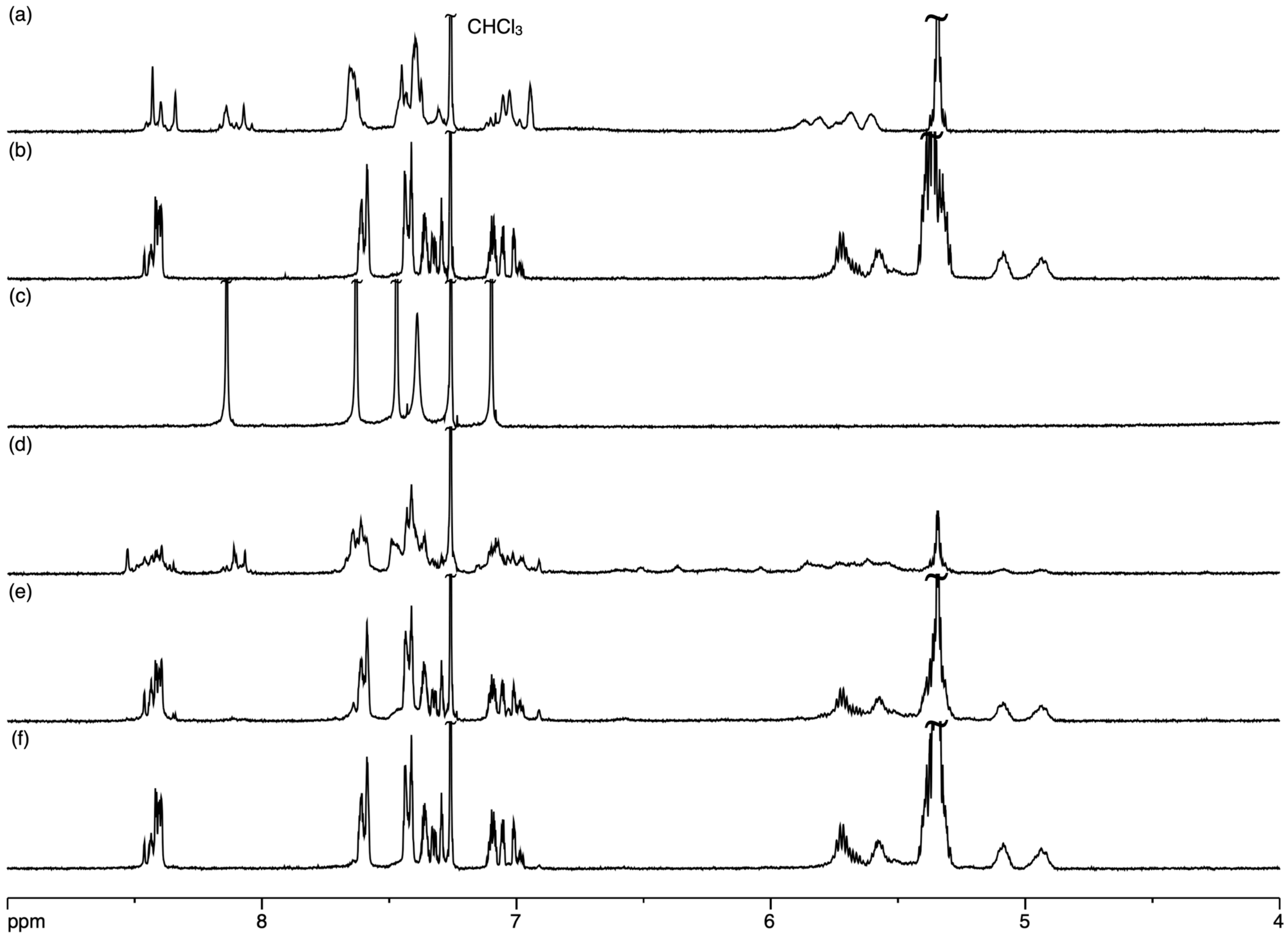

Figure S99. Enlarged ^{1}H NMR spectra of Figure S98.

Table S7. Ratio of [**C4**Pd_4(methyl oleate)$_n$].

Entry	Condition[a]		Ratio of	
	Methyl linolenate	Methyl oleate	[**C4**Pd_4(methyl oleate)$_n$]	$\frac{[\mathbf{C4}Pd_4(\text{methyl linolenate})_2]}{[\mathbf{C4}Pd_4(\text{methyl oleate})_n]}$
(e)	2.3 equiv.	2.3 equiv.	<10%	>9
(f)	3.4 equiv.	3.4 equiv.	<3%	>37

a … Pd-tetrasap (2.2 mM).

Competition of methyl linolenate and methyl linoleate

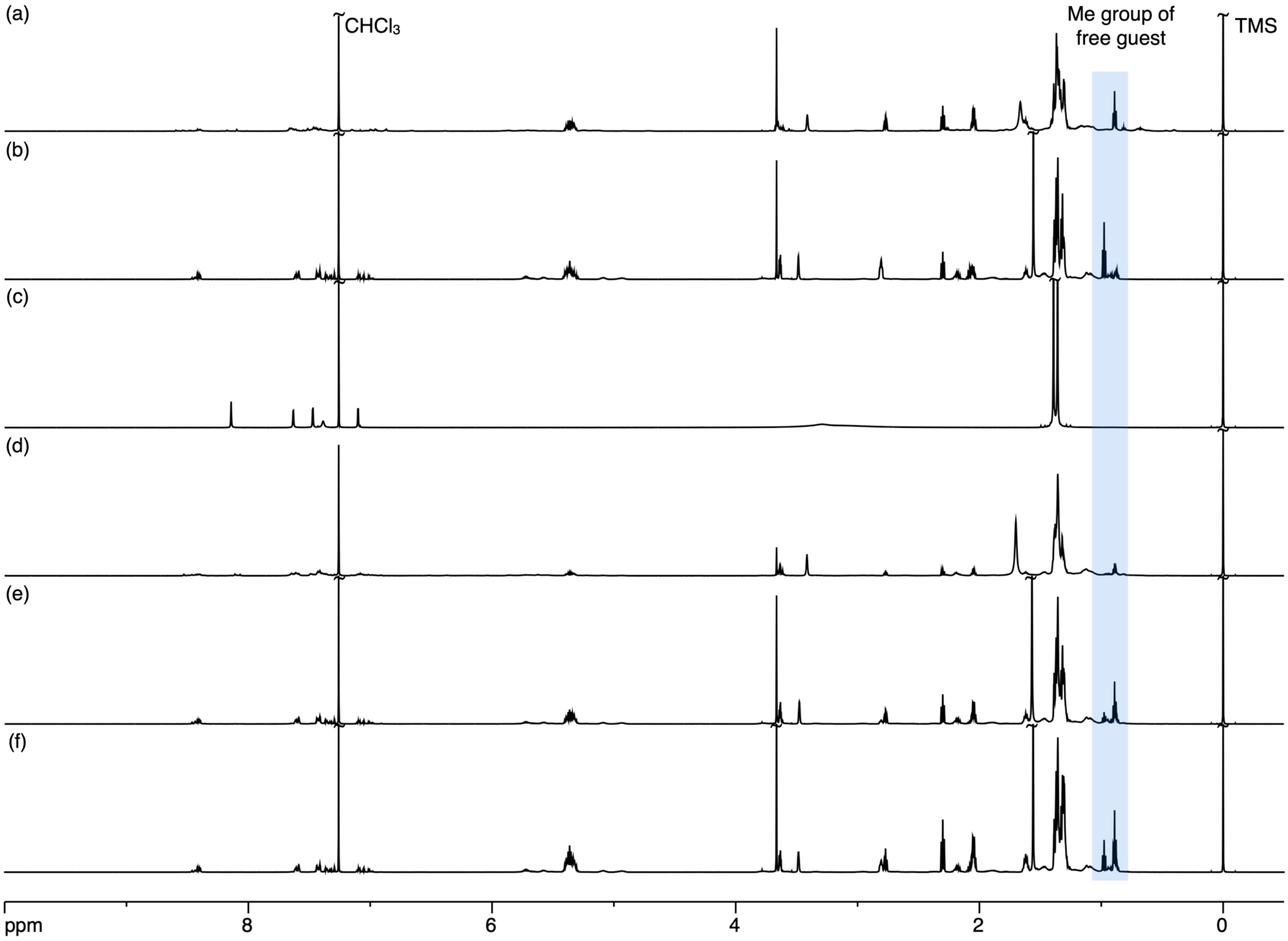

Figure S100. Titration of methyl linolenate and methyl linoleate to [**C4**Pd_4L_4]. (a–f) ^1H NMR spectra (600 MHz, $CDCl_3$). (a) [**C4**Pd_4L_4] + methyl linoleate 4.5 equiv. (b) [**C4**Pd_4L_4] + methyl linolenate 4.5 equiv. (c) [**C4**Pd_4L_4]. (d) [**C4**Pd_4L_4] + (methyl linolenate 1.1 equiv. + methyl linoleate 1.1 equiv.). (e) [**C4**Pd_4L_4] + (methyl linolenate 2.3 equiv. + methyl linoleate 2.3 equiv.). (f) [**C4**Pd_4L_4] + (methyl linolenate 3.4 equiv. + methyl linoleate 3.4 equiv.). L_4 = $(MeOH)_{2.0}(H_2O)_{2.0}$.

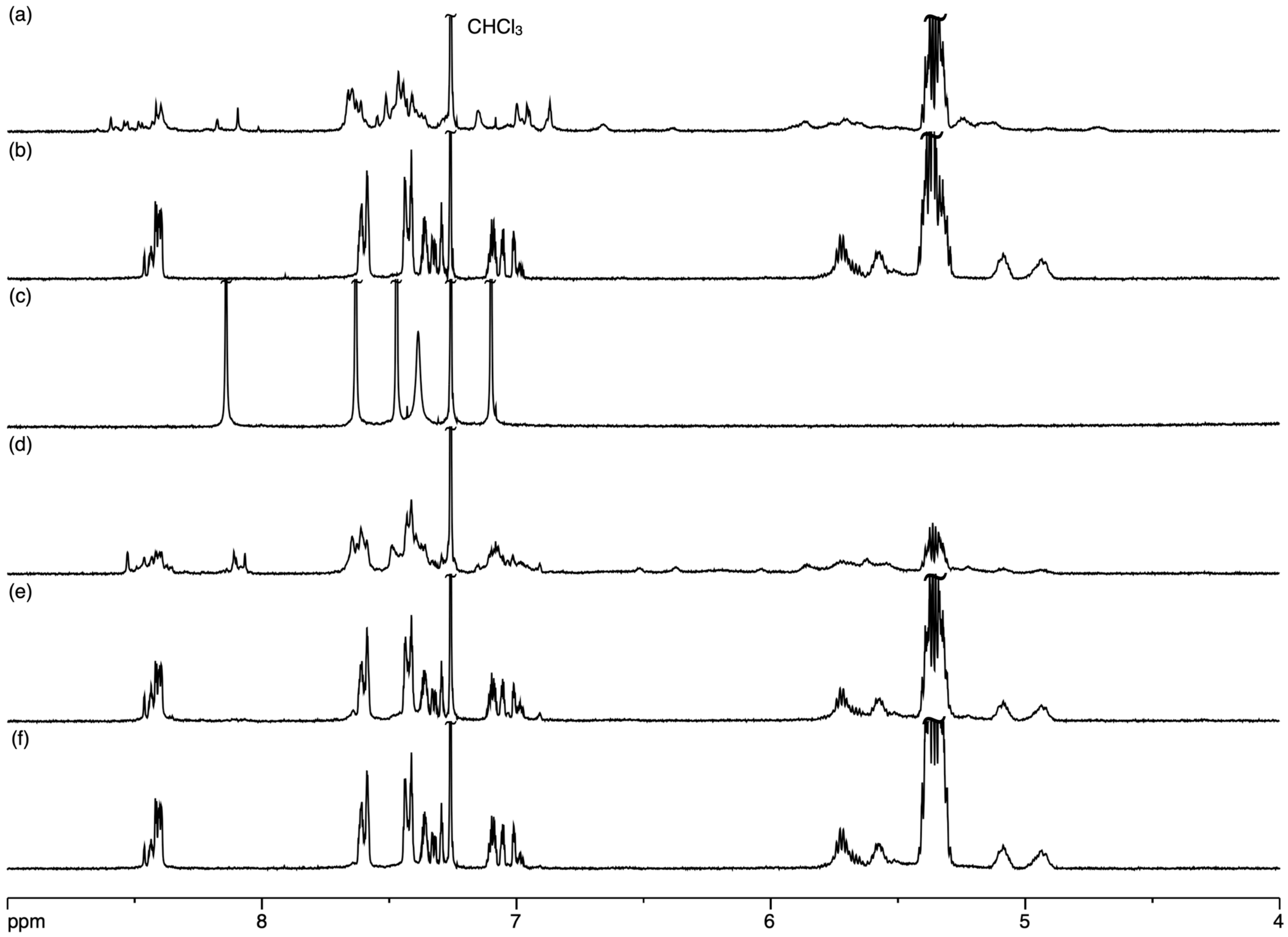

Figure S101. Enlarged ^{1}H NMR spectra of Figure S100.

Table S8. Ratio of [**C4**Pd_4(methyl linoleate)$_n$].

Entry	Condition[a]		Ratio of	$\frac{[\mathbf{C4}Pd_4(\text{methyl linolenate})_2]}{[\mathbf{C4}Pd_4(\text{methyl linoleate})_n]}$
	Methyl linolenate	Methyl linoleate	[**C4**Pd_4(methyl linoleate)$_n$]	
(e)	2.3 equiv.	2.3 equiv.	<13%	>7
(f)	3.4 equiv.	3.4 equiv.	<10%	>9

a … Pd-tetrasap (2.1 mM).
